# Structure‐Guided Design of G‐Protein‐Coupled Receptor Polypharmacology

**DOI:** 10.1002/anie.202101478

**Published:** 2021-07-16

**Authors:** Stefanie Kampen, Duc Duy Vo, Xiaoqun Zhang, Nicolas Panel, Yunting Yang, Mariama Jaiteh, Pierre Matricon, Per Svenningsson, Jose Brea, Maria Isabel Loza, Jan Kihlberg, Jens Carlsson

**Affiliations:** ^1^ Science for Life Laboratory Department of Cell and Molecular Biology Uppsala University 75124 Uppsala Sweden; ^2^ Department of Clinical Neuroscience Karolinska Institute 17177 Stockholm Sweden; ^3^ USEF Screening Platform-BioFarma Research Group Centre for Research in Molecular Medicine and Chronic Diseases University of Santiago de Compostela 15706 Santiago de Compostela Spain; ^4^ Department of Chemistry-BMC Uppsala University 75123 Uppsala Sweden

**Keywords:** drug design, Parkinson's disease, polypharmacology, receptors, virtual screening

## Abstract

Many diseases are polygenic and can only be treated efficiently with drugs that modulate multiple targets. However, rational design of compounds with multi‐target profiles is rarely pursued because it is considered too difficult, in particular if the drug must enter the central nervous system. Here, a structure‐based strategy to identify dual‐target ligands of G‐protein‐coupled receptors is presented. We use this approach to design compounds that both antagonize the A_2A_ adenosine receptor and activate the D_2_ dopamine receptor, which have excellent potential as antiparkinson drugs. Atomic resolution models of the receptors guided generation of a chemical library with compounds designed to occupy orthosteric and secondary binding pockets in both targets. Structure‐based virtual screens identified ten compounds, of which three had affinity for both targets. One of these scaffolds was optimized to nanomolar dual‐target activity and showed the predicted pharmacodynamic effect in a rat model of Parkinsonism.

## Graphical abstract

Structure‐based modelling was used to design a single compound with the ability to modulate the activity of two G‐protein‐coupled receptors relevant for Parkinson's disease. The most potent scaffold displayed nanomolar binding affinities for both targets and was active in a rat model of parkinsonism.

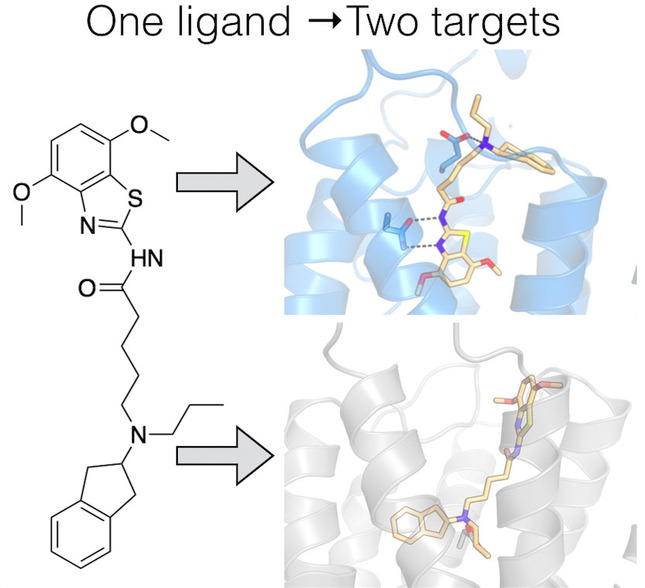

## Introduction

Despite major efforts from the pharmaceutical industry, effective therapies for many central nervous system (CNS) diseases are still lacking.[^1^, ^2^] A common property of CNS drugs (e.g. antipsychotics) is that these compounds interact with multiple targets and that this is essential for their therapeutic effect.[^3^, ^4^] The fact that multi‐target profiles may be required for treatment of complex diseases contrasts with the philosophy of modern drug discovery, which focuses on ligands with selectivity for a single target. However, drugs that modulate several nodes in a network of targets often provide synergistic therapeutic effects, fewer side effects, and are more cost‐effective compared to combination therapy based on single‐target compounds.[^5^, ^6^, ^7^, ^8^, ^9^] The potential of polypharmacology has been recognized for more than a decade, but further progress is limited by difficulties to rationally design such compounds.[^4^, ^6^, ^8^, ^9^, ^10^]

We undertook the challenge to design ligand polypharmacology relevant for Parkinson's disease, a neurological disorder that has proven very difficult for traditional drug development.[^1^, ^11^] In Parkinson's disease, progressive degeneration of dopaminergic neurons leads to motor dysfunction that initially is treated effectively with the dopamine precursor l‐DOPA. However, long‐term use of l‐DOPA leads to a gradual loss of drug efficacy and side effects such as motor fluctuations and dyskinesia.^[1]^ An attractive alternative to targeting only the dopamine receptors is to consider the network of G‐protein‐coupled receptors (GPCRs) in the basal ganglia controlling movement, which includes the A_2A_ adenosine receptor.^[12]^ A compound with the ability to interact with both the A_2A_ adenosine receptor (A_2A_AR) and the D_2_ dopamine receptor (D_2_R) could delay progression of the disease and treat the symptoms. Antagonism of the A_2A_AR is not only symptomatic, but also neuroprotective in animal models of Parkinsonism.[^13^, ^14^] This complements the strictly symptomatic benefits of D_2_ agonists. Moreover, A_2A_AR antagonists alleviate dyskinetic side effects of long‐term l‐DOPA treatment.^[15]^ The dual‐target approach is supported by the fact that combined treatment with a D_2_ agonist and A_2A_ antagonist has synergistic therapeutic effects.^[16]^

Rational design of drugs targeting GPCRs is currently being accelerated by breakthroughs in structural biology,^[17]^ providing opportunities to design drugs with novel properties.^[18]^ In this study, we developed a structure‐based approach to design GPCR polypharmacology, which was employed to identify a compound that antagonizes the A_2A_AR and activates the D_2_R. Structure‐based virtual screening was used to predict dual‐target compounds that were synthesized and evaluated experimentally. One scaffold with affinity for both targets was optimized, leading to potent dual‐target ligands with functional activity tailored for treatment of Parkinson's disease. Our results suggest a general strategy to design dual‐target ligands of GPCRs.

## Results and Discussion

### Structure‐Guided Design of a Virtual Library

**Figure 1:**
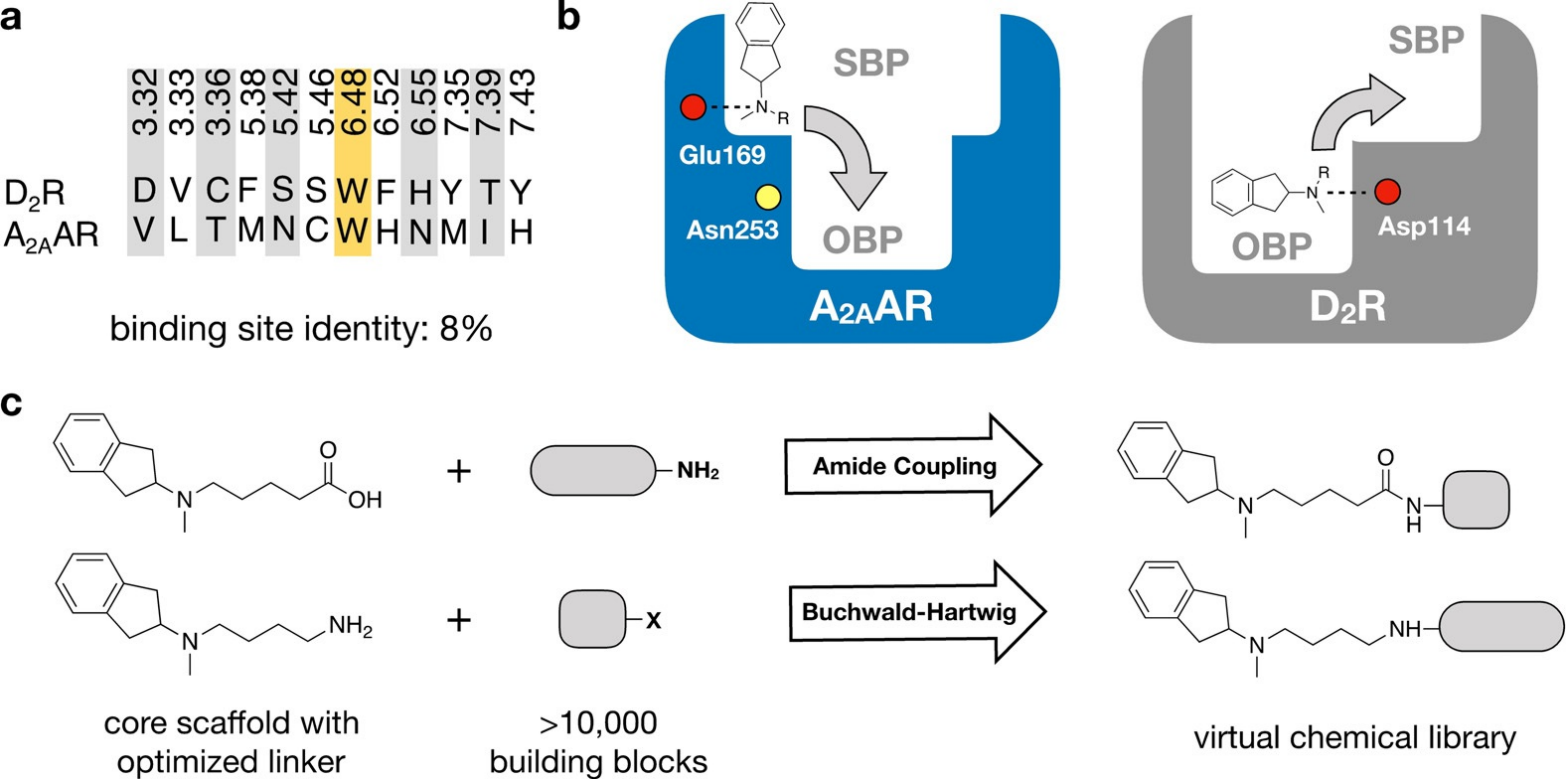
Design of virtual chemical library. a) Sequence alignment of the orthosteric binding sites of the A_2A_AR and D_2_R. Only one out of 12 residues (marked yellow) is the same in both pockets. b) A virtual chemical library was constructed guided by the receptor binding sites. Compounds were designed to target both the orthosteric binding pocket (OBP) and a secondary binding pocket (SBP). Key binding site residues are shown as circles (negatively charged Glu169 and Asp114 in red, and Asn253 in yellow). c) An N‐methyl‐2‐aminoindane (**1**) scaffold was used as the core fragment of the virtual library, which was connected to building blocks using two reactions (amide coupling and Buchwald‐Hartwig amination).

The binding sites of the A_2A_AR and D_2_R were first inspected to assess strategies to design dual‐target ligands of these two GPCRs. Several crystal structures of the A_2A_AR in the inactive conformation, which is the relevant state for development of antiparkinson drugs, were analyzed.^[19]^ No experimental structure of the D_2_R was available and therefore a homology model of the agonist‐bound receptor was used. The recently determined structures of the D_2_R confirmed the accuracy of the binding site model.[^20^, ^21^] Structural alignment of the targets revealed large differences between the orthosteric sites that recognize the endogenous ligands adenosine and dopamine. This was evident both based on the overall shapes of the orthosteric sites and the lack of sequence identity in this region (Figure 1 a). Only one out of 12 residues, the GPCR family‐conserved Trp^6.48^ (Ballesteros‐Weinstein residue numbering scheme is shown as superscripts^[22]^), was the same in both sites and key residues for ligand recognition (A_2A_AR/Asn253^6.55^ and D_2_R/Asp114^3.32^) were different.[^19^, ^23^] The disparate nature of the targets was further supported by comparing known A_2A_AR and D_2_R ligands from the ChEMBL bioactivity database.^[24]^ Whereas the vast majority of D_2_R ligands are cations, compounds that bind to the A_2A_AR are neutral (Supplementary Table 1). Identifying compounds with dual A_2A_/D_2_ activity hence appeared very challenging.

Many class A GPCRs have secondary binding pockets, which are formed by the extracellular entrance to the orthosteric site. Secondary pockets are targets of allosteric modulators and subtype selective ligands can be obtained by forming interactions in these less conserved regions.[^25^, ^26^] We hypothesized that the secondary binding pockets could also be targeted to achieve polypharmacology and analysed these sites in both receptors. We identified that Glu169^EL2^ in a secondary binding pocket at the extracellular interface of the A_2A_AR could potentially act as counter ion to a positive charge, which is one of the main characteristics of D_2_R ligands. This was supported by crystal structures of the A_2A_AR in complex with compounds that extended towards this region.[^27^, ^28^] Based on the observation that the secondary binding pocket of A_2A_AR and the orthosteric site of D_2_R could both accommodate cations, we searched for a starting point for ligand design among D_2_R agonists. Among the few privileged structures that activate the D_2_R, N‐methyl‐2‐aminoindane was selected (compound **1**).^[29]^ Molecular docking calculations positioned N‐methyl‐2‐aminoindane in the orthosteric site of the D_2_R model and its charged nitrogen formed a salt bridge to Asp114^3.32^, an interaction that is conserved among biogenic amine GPCRs (Supplementary Figure 1).^[23]^ The predicted binding mode suggested that the core scaffold could be expanded into a secondary pocket in the D_2_R by linking a building block to the amino moiety (Figure 1 b). Similarly, N‐methyl‐2‐aminoindane docked to a secondary binding pocket in the A_2A_AR crystal structure revealed that a building block fused to the core scaffold could access the orthosteric binding site (Figure 1 b and Supplementary Figure 1). Structural analysis hence supported that dual‐target ligands of the A_2A_AR and D_2_R could be obtained by targeting the orthosteric and secondary pockets.

In the next step, a virtual chemical library with potential dual‐target ligands was designed. The library was generated by linking building blocks to the core scaffold with robust reactions that enabled rapid synthesis. The receptor binding sites guided identification of two linkers that connected the N‐methyl‐2‐aminoindane to building blocks. Linkers of optimal length to access both the orthosteric and secondary binding pockets with either a terminal carboxyl or amino group were selected, which allowed facile connection of building blocks by amide coupling or Buchwald‐Hartwig amination (Figure 1 c). Importantly, the resulting amide and amine moieties from these reactions would be positioned within hydrogen bond distance of Asn253^6.55^ in the A_2A_AR, which fulfilled a key interaction for antagonists in the orthosteric site. The final virtual library was created by connecting the linker groups to building blocks from two sources. The first set of building blocks was based on utilizing known single‐target A_2A_AR ligands from the ChEMBL bioactivity database.^[24]^ As direct connection of potent A_2A_AR ligands led to compounds that lacked drug‐like properties (e.g. due to high molecular weight), we developed an alternative approach. In silico retrosynthesis was used to deconstruct A_2A_AR ligands into building blocks that could be used as starting material for synthesis. Reacting the resulting building blocks with the two linkers resulted in a library with 87 compounds. A second library was created by identifying commercial building blocks in the ZINC15 database^[30]^ that could be fused to the same linkers, yielding an additional 10 448 products (Figure 1 c). The compounds in the virtual library had drug‐like properties with median molecular weight and *c*Log*D* of 368 Da and 1.1, respectively (Supplementary Figure 2).

### Structure‐Based Virtual Screening for Dual‐Target Ligands

Structure‐based docking screens were used to identify the most promising compounds in the virtual library. Each compound was first docked to a crystal structure of the A_2A_AR using the molecular docking program DOCK3.6. Thousands of conformations and orientations of the compounds were explored in the receptor binding site, followed by ranking of these using the binding energy scores.^[31]^ The 500 top‐ranked compounds from the library based on commercial building blocks and the compounds based on the ChEMBL database were inspected visually. Ten compounds (**2**–**11**) were selected for synthesis based on favourable interactions with key residue Asn253^6.55^ in the orthosteric binding site of the A_2A_AR and positioning of the aminoindane moiety in the extracellular vestibule. Docking to the D_2_R supported that the N‐methyl‐2‐aminoindane moiety could maintain the interaction with Asp114^3.32^ in the orthosteric binding site and that the linker allowed the building block to reach into the secondary binding pockets. Six of the selected compounds originated from the library based on commercial building blocks and the remaining four were derived from A_2A_AR ligands using the retrosynthesis approach (Table 1 and Supplementary Table 2).

**Table 1:** Binding affinities of dual‐target ligands discovered from the virtual library.

		Binding affinity [μm] or *% displacement at 10 μm* ^[b]^	
Cmpd (rank^[a]^)	Structure	A_2A_AR	D_2_R
**2** ^[c]^ (**167**)	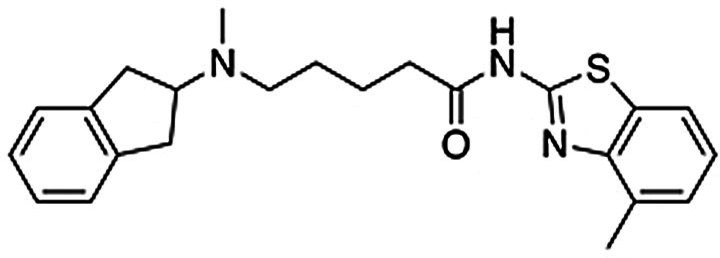	1.2±0.1	0.90±0.08
**3** ^[c]^ (**147**)	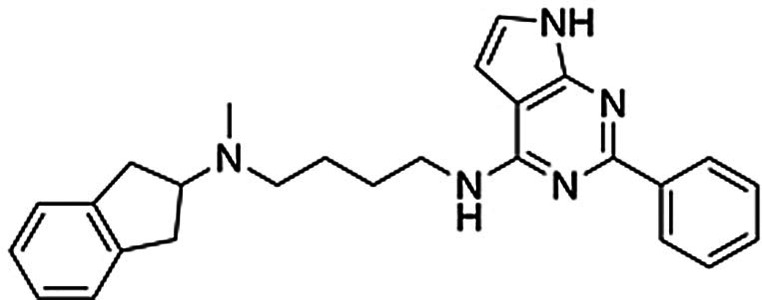	5.6±0.9	0.29±0.03
**4** ^[d]^ (**241**)	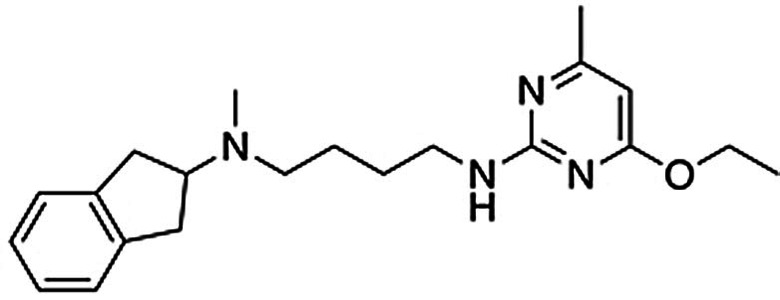	8.4±1.3	0.33±0.03
**12**	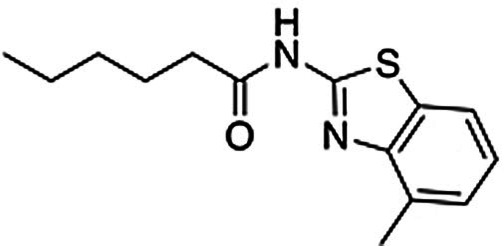	8.1±3.8	*1±2 %*
**13**	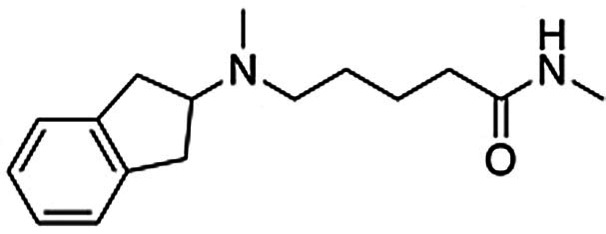	*5±2 %*	5.0±1.3

[a] Ranking in the structure‐based virtual screen of the chemical library. [b] Binding affinities were determined from radioligand displacement assays. Data represent mean values±SEM of three individual experiments performed in duplicate. [c] Building block for synthesis of dual‐target compound identified based on known A_2A_AR ligands from the ChEMBL database. [d] Building block for synthesis of dual‐target compound identified based on commercial chemical library.

### Compound Synthesis

**Scheme 1:**
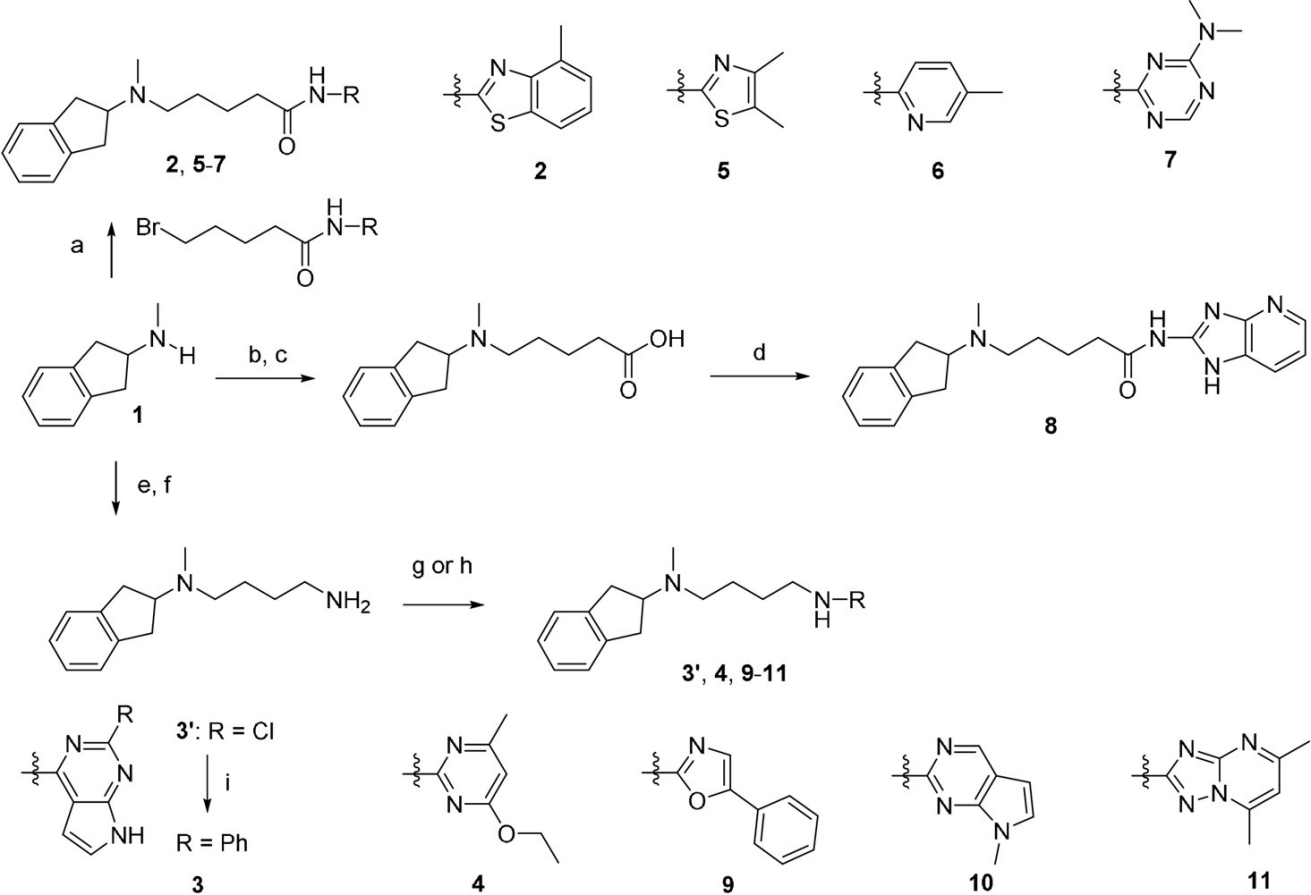
Synthesis of compounds **2**–**11**. Reagents and conditions: a) N‐aryl‐5‐bromopentanamides, K_2_CO_3_, DMF, RT, overnight, 6–65 % (HPLC); b) ethyl 5‐bromopentanoate, K_2_CO_3_, DMF, RT, overnight, 70 %; c) KOH, MeOH, 85 %; d) 1H‐imidazo[4,5‐b]pyridin‐2‐amine, HATU, DIEA, DMF/DCM, RT, overnight, 22 % (HPLC); e) 4‐bromobutanenitrile, K_2_CO_3_, CH_3_CN, RT, overnight, 24 %; f) LiAlH_4_, Et_2_O, 1 h, 82 % (HPLC); g) aryl chlorides, K_2_CO_3_, CH_3_CN, 70–160 °C, 1–4 h, 40–51 % (HPLC) for **3′**, **4**, and **9**; h) aryl chloride or bromide, CuI, 1,10‐phenantroline, K_2_CO_3_, DMF, 120 °C, 48 h, 2–28 % (HPLC) for **10** and **11**; i) phenyl boronic acid, Pd(PPh_3_)_4_, K_2_CO_3_, dioxane/H_2_O, 100 °C, overnight, 20 % (HPLC, over 2 steps).

As planned in the design of the virtual library, the syntheses of compounds **2**–**11** was achieved by short routes of two to four steps (Scheme 1). In brief, compounds **2** and **5**–**7** were obtained by alkylation of N‐methyl‐2‐aminoindane with preformed derivatives of the N‐pentanamide linker and the second building block. Compound **8** was prepared by a three‐step version of this route (Supplementary Scheme 1). The five compounds based on an N‐butylamine linker (**3**–**4** and **9**–**11**) were obtained through routes in which the key steps consisted of alkylation of N‐methyl‐2‐aminoindane by the linker, followed by attachment of the second building block by N‐alkylation or Buchwald‐Hartwig amination (Supplementary Scheme 2). Detailed synthesis procedures are available in the Supplementary Information.

### Biological Assays for Dual‐Target Ligands

**Figure 2:**
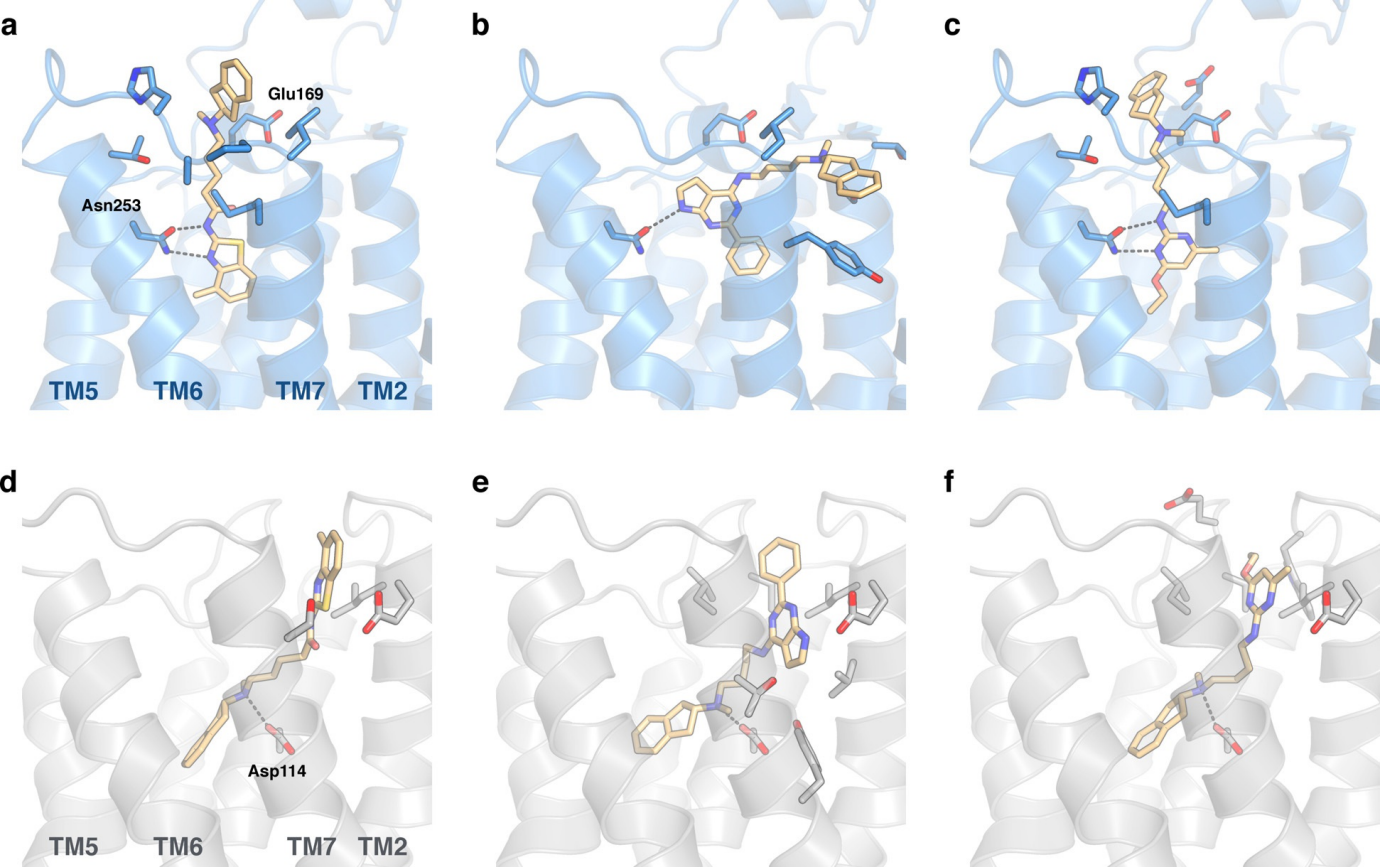
Binding modes of dual‐target ligands. Predicted binding poses of compounds a,d) **2**, b,e) **3**, and c,f) **4**. The A_2A_AR (PDB code: 3PWH^[32]^) and D_2_R (homology model) are shown as blue and grey cartoons, respectively. Key binding site residues and ligands are shown as sticks.

Compounds **2**–**11** were evaluated in competition binding assays (Table 1 and Supplementary Table 2). *K*_i_ values were determined for the three compounds (**2**, **3** and **4**) that showed significant radioligand displacement at 10 μm for both the A_2A_AR and D_2_R. The three hits were ranked in the top 250 of the docking‐ranked library with >10 000 compounds. Compounds **2** and **3** originated from the library based on ChEMBL‐derived building blocks and compound **4** from the set based on a commercial library. The dual‐target ligands had *K*_i_ values between 1.2 and 8.4 μm at the A_2A_AR whereas the affinities for the D_2_R were higher and ranged from 0.29 to 0.90 μm (Table 1). In the predicted binding modes, the discovered ligands formed interactions with the key binding site residues Asn253^6.55^ (A_2A_AR) and Asp114^3.32^ (D_2_R) and explored secondary pockets in the extracellular vestibule (Figure 2). In the A_2A_AR, the linker moieties of compounds **2** and **4** extended towards the extracellular loops and transmembrane helix (TM) 6/7, and the aminoindane moiety was in the vicinity of Glu169^EL2^. The aminoindane moiety of compound **3** interacted primarily with a secondary pocket formed by TM2/7 and was predicted to hydrogen bond with Ser67^2.65^. All compounds formed a salt bridge to Asp114^3.32^ in the D_2_R and extended towards a common secondary binding pocket formed by extracellular loop 2 and TM2/7.

Compound **2** was considered to be the most promising starting point for hit‐to‐lead optimization as it showed the best affinity for the A_2A_AR and submicromolar affinity for the D_2_R (Supplementary Figure 3). Functional experiments measuring G‐protein‐mediated changes in intracellular cAMP also confirmed that compound **2** activated the D_2_R (EC_50_=9.7 μm, E_max_=93 %). Retrospective analysis of how this virtual library compound was generated demonstrated the power of using in silico retrosynthesis to identify building blocks and obtain drug‐like compounds. Whereas the A_2A_AR ligand that it was based on was drug‐like (molecular weight of 434 Da),^[34]^ the retrosynthesis approach deconstructed it into a smaller 2‐amino‐4‐methyl‐benzothiazole building block. This commercially available compound could be connected to the aminoindane scaffold in a single chemical reaction, yielding a dual‐target ligand with lower molecular weight (393 Da) than the original A_2A_AR ligand. In contrast, direct linking of the A_2A_AR ligand would have resulted in a compound with a molecular weight of 635 Da, less favorable physicochemical properties, and a more elaborate synthetic route (Supplementary Figure 4).

To assess if binding to the secondary pockets improved affinity, compounds representing the moieties that anchored the ligands in the orthosteric sites (**12** and **13**) were tested in binding assays. As anticipated, the benzothiazole‐based scaffold **12** showed binding to the A_2A_AR (*K*_i_=8.1 μm), but not to the D_2_R. Conversely, the aminoindane‐based scaffold **13** was a D_2_R ligand (*K*_i_=5.0 μm), but showed no activity for the A_2A_AR. The interactions with the secondary pocket hence improved binding to the A_2A_AR and D_2_R by 7‐ and 6‐fold, respectively. We also noted that benzothiazole is a substructure of the compound Tozadenant, which was evaluated as an antiparkinson drug.^[35]^ A recent crystal structure of the A_2A_AR in complex with this drug candidate (PDB code: 5OLO^33^), which was released after the discovery of compound **2**, confirmed our predicted binding mode of the scaffold (Supplementary Figure 5). Molecular dynamics (MD) simulation refinement of the predicted complex with compound **2** also showed that the interaction with key residue Asn253^6.55^ and a strong salt bridge between the positively charged aminoindane moiety and Glu169^EL2^ were formed (Supplementary Figure 6).

### Optimization of Dual‐Target Activity

Structure‐guided design of analogs to compound **2** was performed to further improve affinity and optimize functional potency. By focusing on commercially available building blocks, analogs could rapidly be synthesized to obtain structure–activity relationships. Detailed synthetic procedures are described in the Supplementary Information.

**Figure 3:**
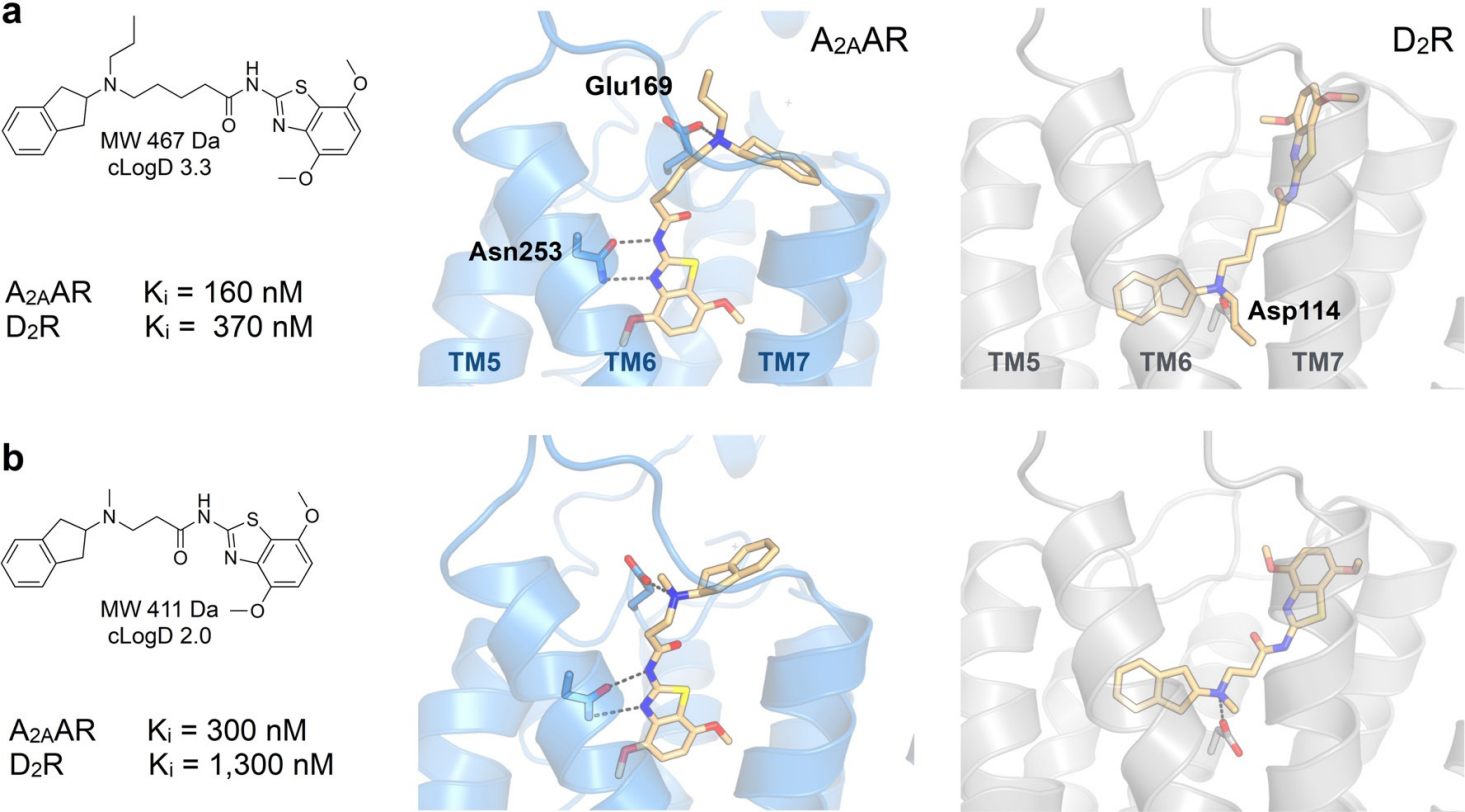
Predicted binding modes of dual‐target ligands. Experimental data and predicted binding modes of compounds a) **30** and b) **37**. The A_2A_AR (PDB code: 5OLO^[33]^, MD‐refined binding modes) and D_2_R (homology model) are shown as blue and grey cartoons, respectively. Key binding site residues and the ligands are shown as sticks.

A series of analogs with modifications on the benzothiazole moiety were first synthesized (**14**–**20**, Table 2 and Supplementary Table 3). Whereas substituents in the 5‐position led to loss of activity (**14**–**17**, Supplementary Table 3), compounds with small substituents in the 4‐position of the benzothiazole moiety had improved affinities for the A_2A_AR (**18**–**20**, Table 2). Compound **20** showed an affinity of 190 nm at the A_2A_AR, corresponding to a >6‐fold improvement over **2**, and a *K*_i_ value of 340 nm at the D_2_R (Supplementary Figure 3). Although compounds **18**–**20** had submicromolar affinities for both targets, functional assays revealed that they were weak D_2_R agonists. The aminoindane group was modified to improve potency and efficacy at the D_2_R. Replacement of the N‐methyl with ethyl or propyl substituents (**21**–**27**) maintained D_2_R affinity and increased functional potency. The benzothiazole moiety was then further optimized and we identified substituents at the 7‐position that resulted in high A_2A_AR affinity (**28**–**30**). Compound **30** had an affinity of 160 nm for the A_2A_AR with nanomolar inhibitor potency in functional assays (*K*_b_=720 nm) and was also a potent D_2_R agonist (*K*_i_=370 nm, EC_50_=180 nm with E_max_=77 %) (Supplementary Figure 3 and Supplementary Figure 7). Models of compound **30** bound to the A_2A_AR and D_2_R showed that hydrogen bonds with the key residues Asn253^6.55^ and Asp114^3.32^, as well as interactions with the secondary binding site, were maintained (Figure 3 a). Physicochemical properties relevant for CNS drugs were calculated for the dual‐target ligands and compared to reference A_2A_AR and D_2_R compounds (Supplementary Table 4). Notably, the most potent dual‐target compounds have properties similar to some approved drugs and are within the recommended property ranges for blood–brain barrier permeability (molecular weight <500, polar surface area <90 Å^2^, number of hydrogen bond donors <3, and *c*Log*D*=2–4).^[36]^

**Table 2:** Experimental data for optimized dual‐target ligands. 
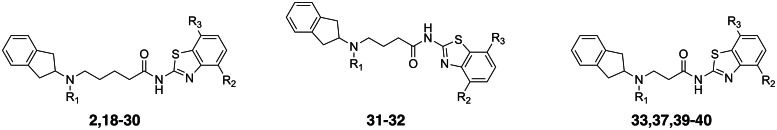

Cmpd	Structure			Binding affinity^[a]^		Functional activity^[a]^		
	R_1_	R_2_	R_3_	A_2A_AR	D_2_R	A_2A_AR	D_2_R	
				*K*_i_ [μm]	*K*_i_ [μm]	*K*_b_ [μm]	EC_50_ [μm]	E_max_ [%]
**2**	CH_3_	CH_3_	H	1.2±0.1	0.90±0.08	–^[b]^	9.7±3.8	93±12
**18**	CH_3_	OCH_3_	H	0.37±0.05	0.39±0.03	–	26±14	89±18
**19**	CH_3_	Cl	H	0.42±0.04	0.51±0.05	–	23±9	82±22
**20**	CH_3_	Br	H	0.19±0.03	0.34±0.03	0.32±0.03	1.5±0.02	62±9
**21**	CH_2_CH_3_	OCH_3_	H	0.63±0.08	0.33±0.02	–	–	–
**22**	CH_2_CH_3_	Br	H	0.34±0.05	0.34±0.04	–	2.5±0.9	119±6
**23**	CH_2_CH_2_CH_3_	F	H	2.9±0.6	0.20±0.02	–	–	–
**24**	CH_2_CH_2_CH_3_	CH_2_CH_3_	H	11±3	0.82±0.18	–	–	–
**25**	CH_2_CH_2_CH_3_	CH_3_	H	0.99±0.24	0.34±004	–	0.75±0.07	81±5
**26**	CH_2_CH_2_CH_3_	OCH_3_	H	0.61±0.09	0.23±0.02	3.6±2.0	0.99±0.38	94±3
**27**	CH_2_CH_2_CH_3_	Br	H	0.39±0.05	0.53±0.18	0.51±0.24	0.18±0.03	89±4
**28**	CH_2_CH_2_CH_3_	OCH_3_	Cl	0.47±0.11	0.90±0.10	–	1.2±0.2	104±6
**29**	CH_2_CH_2_CH_3_	OCH_3_	CH_3_	0.46±0.04	0.67±0.07	1.9±0.7	0.98±0.01	105±5
**30**	CH_2_CH_2_CH_3_	OCH_3_	OCH_3_	0.16±0.03	0.37±0.03	0.72±0.25	0.18±0.04	77±5
**31**	CH_3_	CH_3_	H	1.6±0.3	0.63±0.05	–	5.2±2.6	88±13
**32**	CH_3_	Br	H	0.67±0.11	0.88±0.11	–	1.2±0.3	51±4
**33**	CH_3_	Br	H	1.1±0.2	2.2±0.3	–	5.0±2.8	81±6
**37**	CH_3_	OCH_3_	OCH_3_	0.30±0.05	1.3±0.2	–	31±12	103±3
**39**	CH_2_CH_3_	OCH_3_	CH_3_	1.3±0.2	2.0±0.3	–	28±9	107±6
**40**	CH_2_CH_3_	OCH_3_	OCH_3_	0.72±0.07	1.9±0.3	–	8.9±2.7	105±5

[a] Data represent mean values±SEM of three individual experiments each performed in duplicate. E_max_ values are relative (%) to the maximal effect of dopamine. [b] Not determined.

Modification of the linker was explored to test if it could be shortened and thereby reduce the size and lipophilicity of the scaffold. N‐butanamide and N‐propanamide linkers generally reduced affinity, but several compounds in the series maintained submicromolar K_i_ values for at least one of the targets and low micromolar for the other (**31**–**41,** Table 2 and Supplementary Table 5). For example, compound **37** had affinities of 300 and 1300 nm for the A_2A_AR and D_2_R, respectively, and had lower molecular weight and cLogD values (Table 2 and Figure 3 b). Several of the compounds were D_2_R agonists, but functional potency was reduced to between 1.2 and 31 μm. These results supported that the optimal linker length had been selected in the library design and that physicochemical properties could be further optimized with maintained dual‐target activity.

### Selectivity of Dual‐Target Compounds

A potential concern in development of ligands with polypharmacological profiles is that such compounds may be promiscuous rather than interacting specifically with the targets.^[6]^ To assess the selectivity properties of the dual‐target ligands, we tested six compounds (**18**, **20**, **22**, **26**, **27**, and **30**) in binding assays at the A_1_ adenosine receptor (A_1_AR), D_3_ dopamine receptor (D_3_R), D_4_ dopamine receptor (D_4_R), and H_1_ histamine receptor (H_1_R) (Supplementary Table 6). The compounds generally showed high affinity for the D_3_R, which was expected considering the high sequence similarity to the D_2_R. Compared to the two targets, comparable or substantially weaker ligand binding affinities were observed for the A_1_AR, D_4_R and H_1_R. Notably, compound **30** showed high affinity for the A_2A_AR and D_2_R, but displayed weak or no significant binding to the A_1_AR, D_4_R and H_1_R (*K*_i_>10 μm).

### Blood‐Brain Barrier Penetration

**Figure 4:**
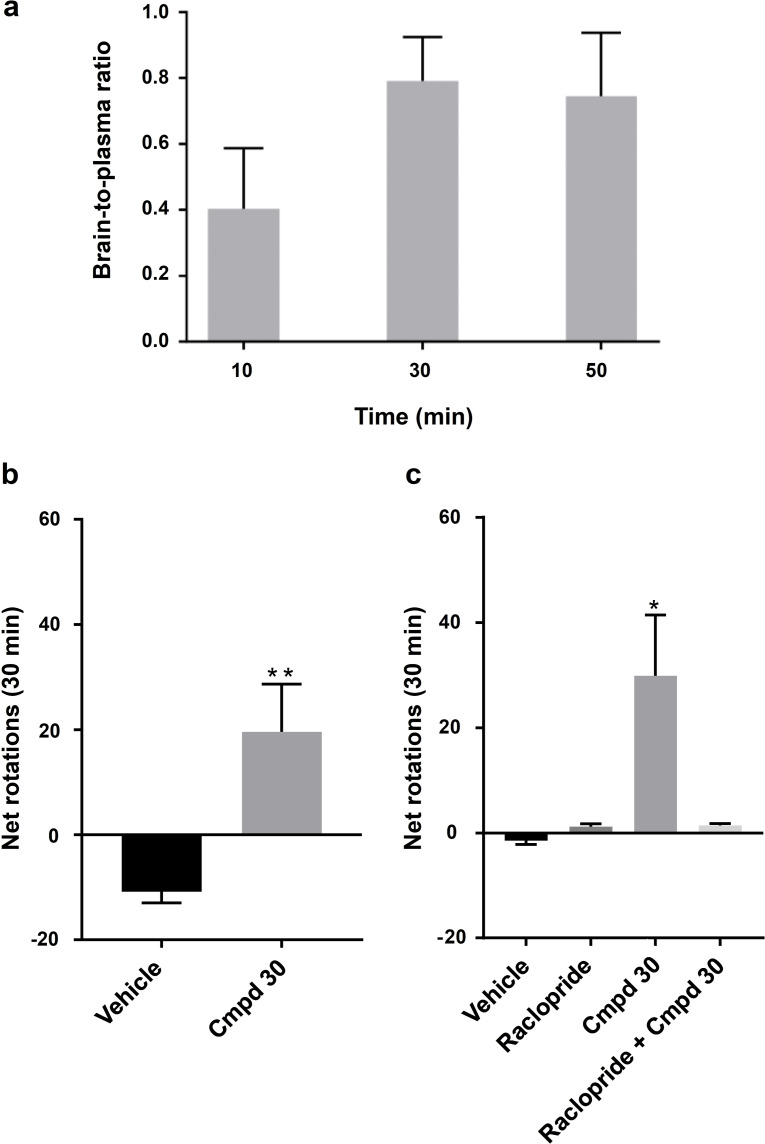
Blood‐brain barrier penetration and evaluation in rodent model of Parkinsonism. a) Brain‐to‐plasma ratio of compound **30** determined in rats (24 mg kg^−1^). Data represent mean±SD. b) Bar graph of the number of net rotations (contralateral‐ipsilateral rotation count, 30 min) induced by DMSO (17 %)/saline (*n*=6) or compound **30** (*n*=7, 24 mg kg^−1^, IP) dissolved in DMSO (17 %)/saline. **P<0.01 accordingly to Mann‐Whitney test. c) Bar graph of the number of net rotations (30 min) induced by DMSO (17 %)/saline (*n*=6), raclopride (*n*=7, 2 mg kg^−1^, IP), compound **30** (*n*=7, 24 mg kg^−1^, IP) or the combination of raclopride and compound **30** (*n*=7) dissolved in DMSO (17 %)/saline. *P<0.05 accordingly to One‐way ANOVA followed by Tukey's multiple comparisons test. Data represent mean±SEM.

The permeability of compound **30** across a Caco‐2 cell monolayer was first determined to assess its likelihood to cross the blood–brain barrier. As **30** displayed medium to high permeability (P_app_ AB: 3.8×10^−6^ cm s^−1^) and a moderate efflux ratio (ER: 8.0), its brain exposure was determined in rats. Intraperitoneal (IP) administration of **30** (24 mg kg^−1^) resulted in a brain‐to‐plasma ratio that peaked at 0.79±0.13 after 30 min (Figure 4 a). For comparison, CNS active drugs such as morphine and risperidone have brain‐to‐plasma ratios of 0.69 and 0.95, respectively.^[37]^ At 30 min compound **30** reached a total concentration of close to 3 μm in the brain, that is, several fold higher than its affinity at the A_2A_AR and D_2_R (Supplementary Figure 8). Taken together these results demonstrated that **30** achieves a sufficiently high brain exposure to justify the evaluation of its activity in an animal model for Parkinson's disease.

### Evaluation in Rodent Model of Parkinsonism

To assess if the designed dual‐target ligands could elicit antiparkinsonian effects, we administrated compound **30** intraperitoneally to unilaterally 6‐OHDA lesioned rats and performed a rotation test. 6‐OHDA lesioned rats display an innate tendency to rotate ipsilaterally due to the unequal dopaminergic innervation. D_2_R agonists induce contralateral rotational behaviour in unilaterally dopamine depleted rats, and this was confirmed by administration of apomorphine (Supplementary Figure 9).^[38]^ Rats treated with the dual‐target compound **30** (24 mg kg^−1^) had a significantly higher number of contralateral rotations compared to the control group (*p*<0.01, Figure 4 b). Using an independent set of rats, we assessed if the effect was mediated through the dopamine receptors by performing the experiments in the presence of the D_2_ antagonist raclopride (Figure 4 c). Administration of compound **30** again resulted in a significant increase of contralateral rotations (*p*<0.05). This effect was reversed if compound **30** was administered in combination with the D_2_ antagonist raclopride, confirming the involvement of dopamine receptors (Figure 4 c). These experiments showed that dual‐target compound **30** is taken up from the intraperitoneal space and is able to cross the blood–brain barrier to reach its targets in the CNS, where it elicits the desired antiparkinsonian effect.

### Strategies to Design Drugs with Polypharmacological Profiles

Polypharmacology poses a major challenge for the traditional drug discovery approach, and the optimal compound design strategy depends on the nature of the targets. In favorable cases, the targets of interest recognize similar compounds and multi‐target activity can be found among previously identified single‐target ligands or by identifying common pharmacophoric features.^[39]^ For example, Besnard et al. combined a ligand‐based method with machine learning to optimize an acetylcholineesterase inhibitor scaffold for activity at both D_2_R and acetylcholinesterase.^[40]^ Similarly, Keiser et al. used chemical similarity methods to identify polypharmacology of approved drugs.^[41]^ However, ligand‐based approaches are generally limited to targets with overlapping pharmacophore features. For disparate targets, one possible strategy is to design bivalent compounds, which consist of two single‐target ligands connected by a linker. Although these are useful as chemical probes to study GPCR dimers,[^25^, ^42^, ^43^] this approach often leads to compounds with high molecular weight that are unlikely to possess drug‐like properties and cross the blood–brain barrier. Structure‐based modelling provides an additional route to design drugs with multi‐target profiles. Crystal structures of GPCRs have revealed druggable secondary binding pockets, which can be targeted either by allosteric or bitopic compounds. Bitopic (also termed dualsteric) ligands that extend into secondary binding pockets have primarily been used to attain subtype selectivity and biased signalling.[^25^, ^26^, ^44^] Here, we demonstrate that such compounds can also be used to design polypharmacology. Bitopic compounds hence provide a general approach to tune selectivity, efficacy, and multi‐target activity to achieve maximal therapeutic effect and minimal side effects. As the presence of secondary pockets appears to be a general property of class A GPCRs (Supplementary Figure 10), the same strategy can be applied to many targets in this large family of therapeutically relevant proteins.

## Conclusion

Although the importance of polypharmacology in treatment of complex diseases is well‐established,[^3^, ^4^, ^5^, ^6^, ^8^, ^9^, ^10^, ^45^] the pharmaceutical industry has stayed clear of multi‐target drugs because it has been considered to be too challenging. Here, we present the first example of structure‐guided design of dual‐target activity at disparate GPCR targets. We show that it is possible to rapidly develop compounds with complex polypharmacology that display in vivo activity even for targets located in the CNS. We expect that the rapidly increasing access to structural and bioactivity data for GPCRs^[46]^ will make it possible to apply the same approach to design drugs with interaction profiles tailored for treatment of other complex diseases.

## Conflict of interest

The authors declare no conflict of interest.

## Supporting information

As a service to our authors and readers, this journal provides supporting information supplied by the authors. Such materials are peer reviewed and may be re‐organized for online delivery, but are not copy‐edited or typeset. Technical support issues arising from supporting information (other than missing files) should be addressed to the authors.

Supporting Information
