# Supplementary material for: Structure‐Guided Design of G‐Protein‐Coupled Receptor Polypharmacology
Source: Angew Chem Int Ed Engl. 2021 Jul 16;60(33):18022–30. doi: 10.1002/anie.202101478 (PMC8456950; doi:10.1002/anie.202101478)
Supplement: Supplementary file 1 — Supporting Information [file ANIE-60-18022-s001.pdf]

Angewandte Chemie
Eine Zeitschrift der Gesellschaft Deutscher Chemiker

Supporting Information

Structure-Guided Design of G-Protein-Coupled Receptor Polypharmacology

*Stefanie Kampen[+], Duc Duy Vo[+], Xiaoqun Zhang, Nicolas Panel, Yunting Yang, Mariama Jaiteh, Pierre Matricon, Per Svenningsson, Jose Brea, Maria Isabel Loza, Jan Kihlberg, and Jens Carlsson**

anie_202101478_sm_miscellaneous_information.pdf

Supplementary Information:

Structure-Guided Design of G-Protein-Coupled Receptor Polypharmacology

Stefanie Kampen[1], Duc Duy Vo[1], Xiaoqun Zhang[2], Nicolas Panel[1], Yunting Yang[2], Mariama Jaiteh[1], Pierre Matricon[1], Per Svenningsson[2], Jose Brea[3], Maria Isabel Loza[3], Jan Kihlberg[4], and Jens Carlsson[1,*]

[1]Science for Life Laboratory, Department of Cell and Molecular Biology, Uppsala University, SE-751 24 Uppsala, Sweden.

[2]Department of Clinical Neuroscience, Karolinska Institute, SE-171 77 Stockholm, Sweden

[3]USEF Screening Platform-BioFarma Research Group, Centre for Research in Molecular Medicine and Chronic Diseases, University of Santiago de Compostela, 15706 Santiago de Compostela, Spain.

[4]Department of Chemistry-BMC, Uppsala University, SE-75123 Uppsala, Sweden.

Table of Contents

Page

Author contributions S3

Methods

Computational Methods
Virtual chemical library S4
Homology modelling S5
Molecular docking S5
Molecular dynamics simulations S6

Experimental Methods
General synthetic procedures S8
Binding assays S9
Functional assays S12
Cell permeability S13
Blood-brain distribution S14
Evaluation of compound **30** in rodent model of Parkinsonism S15

Supplementary Figures
Fig. 1. Predicted binding mode of N-methyl-2-aminoindane in D_2R and $A_{2A}AR$. S17
Fig. 2. Distribution of molecular weight and cLogD in the virtual library. S18
Fig. 3. Binding assays for compounds **2**, **20** and **30**. S19
Fig. 4. Design of compound **2** using a retrosynthesis approach. S20
Fig. 5. Comparison of predicted binding modes of compound **2** and Tozadenant. S21
Fig. 6. MD-refined binding mode of compound **2**. S22
Fig. 7. Functional assays of compound **30**. S23
Fig. 8. Brain concentration of compound **30**. S24
Fig. 9. Evaluation of control (apomorphine) in rat model of Parkinsonism. S25
Fig. 10. Primary and secondary binding pockets in GPCRs. S26
Fig. 11. Sequence alignment used to create the D_2R homology model. S27

Supplementary Tables
Table 1. Distribution of ligand charge for the $A_{2A}AR$ and D_2R. S28
Table 2. Binding data for the predicted dual-target ligands. S29
Table 3. Binding data for analogs of compound **2**. S30
Table 4. Calculated compound properties relevant for blood-brain barrier penetration. S31
Table 5. Binding data for analogs of compound **2** with modified linker length. S32
Table 6. Affinities of selected compounds at the A_1AR, D_3R, D_4R, and H_1R. S33

Supplementary Schemes
Scheme 1. Synthesis of compounds **2**, **5-7** and **8**. S34
Scheme 2. Synthesis of compounds **3**, **4** and **9-11**. S35

Supplementary Results: Chemistry
NMR spectra of synthesized compounds. S36

Supplementary References S97

Author contributions

S.K. generated virtual libraries and performed the molecular docking calculations under the supervision of J.C. M.J. generated a homology model of the D_2R. N.P and P.M. performed MD simulations. D.D.V. performed compound synthesis under the supervision of J.K. The binding and functional assays were performed by the USEF screening platform under the supervision of M.I.L and J.B. X.Z. and Y.Y. evaluated compounds in rodent models under the supervision of P.S. S.K, D.D.V., J.K., and J.C wrote the manuscript with contributions from the other authors.

Methods

Computational Methods

Virtual chemical library. A focused compound library was created by connecting the N-methyl-2-aminoindane scaffold to a second building block via either an N-pentanamide or N-butylamine linker. Building blocks compatible with either amide coupling or Buchwald-Hartwig amination were identified from two sources using substructure searches and chemical property filtering with OECHEM 2.1.3 and OMEGA/FILTER 3.1.2.2 (OpenEye Scientific Software, Santa Fe, NM; http://www.eyesopen.com). The first set of building blocks were based on 3461 $A_{2A}AR$ ligands (K_i < 10 μM) obtained from the ChEMBL database[1]. Virtual retrosynthesis of $A_{2A}AR$ ligands containing amine and amide bonds into building blocks compatible with the two selected reactions was performed using Reactor (Version 17.8, ChemAxon, https://www.chemaxon.com). A second set of building blocks were obtained from the ZINC15 database[2] (Purchasability: "In stock", Reactivity: "Standard", MW < 350 Da) by identifying compounds with suitable functional groups. Reactor was used to create the virtual library based the two sets of building blocks. Duplicate compounds and building blocks with more than one reactive functional groups were removed. Compounds that were based on $A_{2A}AR$ ligands with MW < 420 Da were used in the docking screens without any additional filtering. For the virtual library based on commercial building blocks, compounds with ≤ 3 hydrogen bond donors, ≤ 7 hydrogen bond acceptors, and 23-27 heavy atoms were included, and compounds with undesirable moieties were removed. In the hit optimization step, additional virtual libraries were created by identifying relevant building blocks in commercial chemical libraries. All virtual libraries were prepared for docking with DOCK3.6 using the ZINC database protocol[3], which considered relevant tautomeric/protonated forms (pH 6-8) and up to 600 pre-generated

conformations using OMEGA[4]. For analogs of compound **2**, multiple binding poses per ligand were inspected and conformational sampling was increased to include thousands of conformations. Compounds selected for synthesis and experimental evaluation were also analysed with the FAF-Drugs4 server (https://fafdrugs4.rpbs.univ-paris-diderot.fr/)[5] to identify potential pan-assay interference compounds (PAINS) and none of the active compounds contained such chemotypes.

Homology Modelling. Homology models of the D_2R were built using MODELLER (version 9.14)[6] based on two templates. The crystal structure of the D_3R (PDB code: 3PBL[7]) was used to model all loop segments and the transmembrane region except for TM5, which was based on the structure of the active β_2 adrenergic receptor (3SN6[8]). The sequence alignment is shown in Supplementary Fig. 11. Additional alpha helix restraints were added to the extracellular tip of TM5 (residues Pro187$^{5.36}$, Ala188$^{5.37}$, Phe189$^{5.38}$, and Val190$^{5.39}$) and on side chain rotamers (Asp114$^{3.32}$, Ile184^{EL2}, Ser193$^{5.42}$, Ser194$^{5.43}$, and Ser197$^{5.46}$) to mimic the active β_2 adrenergic receptor. Disulfide bridges were introduced in the extracellular loops (residues 399-401 and 107-182). Molecular docking of 36 D_2R agonists and property-matched decoys[9] to a set of 1000 homology models was used to select a binding site structure that showed high enrichment of ligands.[10]

Molecular docking. All molecular docking calculations were carried out using DOCK3.6 (http://dock.compbio.ucsf.edu/DOCK3.6/).[11] Docking to the $A_{2A}AR$ was performed using a crystal structure (PDB code: 3PWH[12]). Docking to the D_2R binding site was based on a homology model. The crystal structure was prepared by removing non-protein atoms. Mutated residues in the binding site of the $A_{2A}AR$ (A54$^{2.52}$L, T88$^{3.36}$A and S277$^{7.42}$A) were modified by adding the side chains that correspond to the wild type sequence, and unresolved

side chains were added manually using PyMol (Version 1.4.1, Schrödinger, LLC.). The side chains of Arg, Lys, Glu and Asp residues were modelled as charged. Histidine residues were protonated based on visual inspection of the local hydrogen bonding networks. In the case of the A_{2A}AR, residues His230$^{6.32}$, His278$^{7.43}$ and His264^{ECL3} were protonated at the Nδ position, and His75$^{3.23}$ and His250$^{6.52}$ at the Nε position. Histidine side chains in the D_2R structure were protonated at the Nδ position. The binding sites were defined based on the co-crystallized ligands. Sampling of the docked compounds was determined by 45 matching spheres with a matching tolerance of 1.5 Å, bin sizes of 0.4 Å, and overlap of 0.3 Å. The DOCK3.6 scoring function estimates the binding energy as the sum of the van der Waals and electrostatic interaction energy, corrected for ligand desolvation.[11] These energy terms were obtained from pre-calculated grids, which were based on an AMBER force field[13] and prepared using tools available in DOCK3.6. The electrostatic potential grids of the A_{2A}AR and D_2R homology model were modified by increasing the dipole moment of side chains (A_{2A}AR: Asn253$^{6.55}$; D_2R: Ser193$^{5.42}$, Ser194$^{5.43}$ and Ser197$^{5.46}$), as described previously.[14] The best scoring conformation of each docked compound was subjected to 100 steps of rigid-body energy minimization.

Molecular dynamics simulations. All MD simulations were performed based on the atomic coordinates of the A_{2A}AR crystal structure in complex with tozadenant (PDB code: 5OLO)[15]. The crystal structure was prepared by removing non-protein atoms and the BRIL insertion, and engineered mutations were reverted. The receptor was placed in a pre-equilibrated 1-palmitoyl-2-oleoyl-phosphatidylcholine (POPC) lipid bilayer and prepared for simulations in GROMACS[16]. The system was equilibrated for 40 ns at 310 K and atmospheric pressure using the OPLS 2005 all atom force field[17], Berger lipids[18], and the TIP3P water model[19]. The receptor structure was held rigid using tight positional restraints on protein heavy atoms

whereas the membrane and water molecules were allowed to relax. Each receptor-ligand complex was then prepared based on a snapshot of the equilibrated system. The binding modes of the compounds were modelled based on the docking poses. The MD simulations of the complexes were performed with the program Q[20] using the same force field[21]. The simulations were performed under spherical boundary conditions with a sphere radius of 21 Å centered on the ligand. The OPLSAA_2005 force field from the hetgrp_ffgen program (Schrödinger, LLC, New York, NY, 2017) was used to parameterize compounds. Atoms outside the sphere were excluded from non-bonded interactions. Ionizable residues close to the sphere edge were set to their neutral form and atoms within 3 Å of the sphere edge were restrained to their initial coordinates. Water molecules at the sphere border were subject to radial and polarization restraints according to the surface-constrained all atom solvent (SCAAS) model[22]. Solvent bonds and angles were constrained using the SHAKE algorithm[23]. A cutoff of 10 Å was used for non-bonded interactions except for ligand atoms, for which no cutoff was applied. Long-range electrostatic interactions were treated using the local reaction field approximation[24]. Protonation states of ionizable residues Asp, Glu, Arg, and Lys in the binding site were set to the most probable in aqueous solution at pH 7. His264^{ECL3} and His278$^{7.43}$ were protonated at the Nδ position whereas His75$^{3.23}$, His155^{ECL2} and His250$^{6.52}$ were protonated at the ε position. In all simulations, a time step of 1 fs was used and non-bonded pair lists were updated every 25 steps. The receptor-ligand complexes were first equilibrated for 430 ps. During the equilibration, harmonic positional restraints on solute heavy atoms were gradually released and the system was heated to the target temperature. This was followed by unrestrained simulations of 10 ns. Three independent simulations were performed in each case. Simulations were performed for compounds **20**, **30** and **37** based on molecular docking calculations. For compound **2**, the aminoindane moiety was rotated to enable a hydrogen bond to Glu169^{EL2}.

Experimental methods

General synthetic procedures. All reagents were purchased from Fluorochem, Sigma-Aldrich, Enamine, and Chemtronica. For solvents, DCM, methanol, DMF, and acetonitrile (99.9%) were purchased from VWR International AB, whereas THF was purchased from Sigma-Aldrich. Reagents and solvents were used as such without further purification. All reactions involving air or moisture-sensitive reagents or intermediates were performed under a nitrogen atmosphere. Mainly LCMS was used for monitoring reactions using an Agilent 1100 series HPLC having a C18 Atlantis T3 column (3.0 × 50 mm, 5 μm). Acetonitrile–water (flow rate 0.75 mL/min over 6 min) was used as mobile phase and a Waters micromass Z_Q (model code: MM1) mass spectrometer with electrospray ionization mode used for detection of molecular ions. TLC silica gel 60 F_{254} plates from Merck were also used for monitoring reactions and particularly during purification of compounds. Visualization of the developed TLC was performed using UV light (254 nm) and staining with ninhydrin stain or anisaldehyde stain. After workup, organic phases were dried over Na_2SO_4/$MgSO_4$ and filtered before being concentrated under reduced pressure. Silica gel (Matrex, 60 Å, 35–70 μm, Grace Amicon) was used for purification of intermediate compounds with flash column chromatography. ^1H and ^{13}C NMR spectra for synthesized compounds were recorded at 298 K on an Agilent Technologies 400 MR spectrometer at 400 MHz or 100 MHz, or on Bruker Avance Neo spectrometers at 500/600 MHz or 125/150 MHz. Chemical shifts are reported in parts per million (ppm, δ) referenced to the residual ^1H resonance of the solvent ($(CD_3)_2CO$, δ 2.05; $CDCl_3$, δ 7.26; CD_3OD δ 3.31; DMSO-d_6 δ 2.50). Splitting patterns are designated as follows: s (singlet), d (doublet), t (triplet) and m (multiplet), br (broad). Coupling constants (J values) are listed in hertz (Hz). Preparative reversed-phase HPLC was performed on a Kromasil C8 column (250 × 21.2 mm, 5 μm) on a Gilson HPLC equipped

with Gilson 322 pump, UV/Visible-156 detector and 202 collector using acetonitrile-water gradients as eluents with a flow rate of 15 mL/min and detection at 210 or 254 nm. Unless otherwise stated, all the tested compounds were purified by HPLC and high resolution ^{1}H NMR (400-600 MHz) and LCMS were used to assess the purity of the compounds (95%+).

Binding assays. Human $A_{2A}AR$ competition binding experiments were carried out in multiscreen GF/C 96-well plates (Millipore, Madrid, Spain) pretreated with binding buffer (50 mM Tris-HCl, 1 mM EDTA, 10 mM $MgCl_2$, 2 U/ml adenosine deaminase, pH=7.4). Each well contained 5 µg of membranes from a Hela-$A_{2A}AR$ cell line (protein concentration of 2058 µg/ml), 3 nM [^{3}H]-ZM241385 (60 Ci/mmol, 1 mCi/ml, ARC-ITISA 0884), and the compound studied. Non-specific binding was determined in the presence of 50 µM NECA (Sigma E2387). The reaction mixture (200 µl/well) was incubated at 25 °C for 30 min, after which it was filtered and washed four times with 250 µl wash buffer (50 mM Tris-HCl, 1 mM EDTA, 10 mM $MgCl_2$, pH=7.4) before measuring in a microplate beta scintillation counter (Microbeta Trilux, PerkinElmer, Madrid, Spain). Three independent experiments were carried out to calculate K_i values (n=3).

Human D_2R competition binding experiments were carried out in polypropylene 96-well plates. Each well contained 10 µg of membranes from a CHO-D_2R #S20 cell line (protein concentration of 3241 µg/ml), 0.4 nM [^{3}H]-spiperone (80.2 Ci/mmol, 1 mCi/ml, Perkin Elmer NET1187001MC), and the compound studied. Non-specific binding was determined in the presence of 10 µM sulpiride (Sigma S112). The reaction mixture (250 µl/well) was incubated at 25°C for 120 min, after which 200 µL was transferred to a GF/C 96-well plate (Millipore, Madrid, Spain) pretreated with 0.5% of PEI and treated with binding buffer (50 mM Tris-HCl, 1 mM EDTA, 5 mM $MgCl_2$, 5 mM KCl, 120 mM NaCl, pH=7.4). The

samples were filtered and washed four times with 250 µl wash buffer (50 mM Tris-HCl, 0.9% NaCl, pH=7.4) before measuring in a microplate beta scintillation counter (Microbeta Trilux, PerkinElmer, Madrid, Spain). Three independent experiments were carried out to calculate K_i values (n=3).

Human A_1AR competition binding experiments were carried out in multiscreen GF/C 96-well plates (Millipore, Madrid, Spain) pretreated with assay buffer (20 mM Hepes, 100 mM NaCl, 10 mM $MgCl_2$, 2 U/ml adenosine deaminase, pH=7.4). Each well contained 5 µg of membranes from a CHO-A_1AR cell line, 1 nM [^{3}H]-DPCPX (137 Ci/mmol, 1 mCi/Ml, Perkin Elmer NET974001MC), and the compound studied. Non-specific binding was determined in the presence of R-PIA 10 µM (Sigma P4532). The reaction mixture (200 µl/well) was incubated at 25°C for 60 min. The samples were then filtered and washed four times with 250 µl wash buffer (20 mM Hepes, 100 mM NaCl, 10 mM $MgCl_2$, pH=7.4) before measuring in a microplate beta scintillation counter (Microbeta Trilux, PerkinElmer, Madrid, Spain). Two independent experiments were carried out to calculate K_i values (n=2).

D_3R competition binding experiments were carried out in polypropylene 96-well plates. Each well contained 2 µg of membranes from a D_3R cell line (Perkin Elmer, ES-173M400UA, protein concentration=1000 µg/ml), 1 nM [^{3}H]-spiperone (68 Ci/mmol, 1 mCi/ml, Perkin Elmer NET1187001MC), and the compound studied. Non-specific binding was determined in the presence of 1 µM haloperidol (Sigma H1512). The reaction mixture (250 µl/well) was incubated at 25 °C for 60 min, after which 200 µL was transferred to GF/C 96-well plates (Millipore, Madrid, Spain) pretreated with 0.5% PEI and treated with binding buffer (50 mM Tris-HCl, 5 mM $MgCl_2$, pH=7.4). The samples were filtered and washed four times with 250 µl wash buffer (50 mM Tris-HCl, pH=7.4) before measuring in a microplate beta scintillation

counter (Microbeta Trilux, PerkinElmer, Madrid, Spain). Two independent experiments were carried out to calculate K_i values (n=2).

D_4R competition binding experiments were carried out in polypropylene 96-well plates. Each well contained 35 µg of membranes from a CHO-D_4R cell line (Perkin Elmer, RBHD42M400UA, protein concentration=14000 µg/ml), 1 nM [^{3}H]-spiperone (68 Ci/mmol, 1 mCi/ml, Perkin Elmer NET1187001MC), and the compound studied. Non-specific binding was determined in the presence of 25 µM haloperidol (Sigma H1512). The reaction mixture (250 µl/well) was incubated at 27 °C for 120 min, after which 200 µL was transferred to GF/C 96-well plates (Millipore, Madrid, Spain) pretreated with 0.5% PEI and treated with binding buffer (50 mM Tris-HCl, 1 mM EDTA, 5 mM $MgCl_2$, 5 mM KCl, 120 mM NaCl, pH=7.4). The samples were filtered and washed four times with 250 µl wash buffer (50 mM Tris-HCl, 0.9% NaCl, pH=7.4) before measuring in a microplate beta scintillation counter (Microbeta Trilux, PerkinElmer, Madrid, Spain). Two independent experiments were carried out to calculate K_i values (n=2).

H_1R competition binding experiments were carried out in polypropylene 96-well plates. Each well contained 50 µg of membranes from a CHO-H_1R cell line (protein concentration=3860 µg/ml), 4 nM [^{3}H]pyrilamine (20.1 Ci/mmol, 1 mCi/ml, Perkin Elmer NET594250UC), and the compound studied. Non-specific binding was determined in the presence of 10 µM triprolidine (T6764, Sigma Aldrich). The reaction mixture (250 µl/well) was incubated at 27 °C for 60 min, after which 200 µL was transferred to GF/B 96-well plates (Millipore, Madrid, Spain) pretreated with 0.1% Tween 20 and assay buffer (Na+ / K+ phosphate buffer pH=7.4). The samples were then filtered and washed four times with 250 µl wash buffer (Na^+/K^+ phosphate buffer, pH=7.4), after which 30 µL Universol (MP Biomedicals, Spain) was added

and measurements were performed in a microplate beta scintillation counter (Microbeta Trilux, PerkinElmer, Madrid, Spain). Two independent experiments were carried out to calculate K_i values (n=2).

Data were adjusted to non-linear fitting by using Prism V2.1 (GraphPad Inc, Chicago, USA) and K_i values were calculated by using the Cheng-Prusoff equation[25].

Functional assays. For the human A_{2A}AR, functional experiments measuring cAMP production were carried out in CHO-A_{2A} cell line. The day before the assay, the cells were seeded on a 96-well culture plate (Falcon 353072). The cells were washed with wash buffer (Dulbecco's modified eagle's medium nutrient mixture F-12 ham (Sigma D8062), 25 mM Hepes; pH=7.4). Wash buffer was then replaced by incubation buffer (Dulbecco's modified eagle's medium nutrient mixture F-12 ham (Sigma D8062), 25 mM Hepes, 20 μM Rolipram, pH=7.4). The studied compounds were added and incubated at 37 ºC for 15 min. After incubation, NECA (Sigma E2387) was added in several concentrations and incubated at 37 ºC for 15 min. After incubation, the amount of cAMP was determined using the cAMP Biotrak Enzymeimmunoassay (EIA) System Kit (GE Healthcare RPN225). Data were adjusted to non-linear fitting by using Prism V2.1 (GraphPad Inc, Chicago USA). K_B values were calculated from the IC_{50} values obtained for each compound by using the formula: $K_B=IC_{50}/(1+([NECA]/EC_{50}\ NECA))$.

Functional assays for the human D_2R were carried out in a CHO-D_2 #S20 cell line. 5,000 cells were seeded in 30 μl of Opti-MEM (Invitrogen 11058) and 500μM IBMX (Sigma 17018) on a 96-well isoplate (PerkinElmer 6005030). The studied compounds were then added and incubated for 10 min at 37 ºC with gentle stirring (150 rpm). Then, 10 μM

forskolin (Sigma 17018) was added and incubated for 5 min at 37 ºC with gentle stirring (150 rpm). Reagents (#CISBIO 62AM4PEC) were added and, after incubation for 60 min at rt with gentle stirring (90 rpm) and protected from light, homogeneous time resolved fluorescence (HTRF, λ_{Ex}: 320 nm; λ_{Em}: 620-665 nm) from each well was measured using a Tecan Infinite M1000 Pro. Data were normalized to the maximum effect of dopamine. EC_{50} and E_{max} values were obtained after fitting the data to a four-parameter logistic equation using Prism V2.1 (Graph Pad Inc, Chicago, USA).

Cell permeability. The Caco-2 cell permeability of compound **30** was determined by the Uppsala university optimization and pharmaceutical profiling platform (UDOPP) as described previously[26]. Briefly, Caco-2 cell monolayers (passage 94-105) were grown on a permeable filter support and used on day 21 after seeding. Prior to the experiment a 10 µM solution of **30** was prepared by dilution of a 10 mM stock solution in DMSO with Hank's balanced salt solution (HBSS; 7.4) and warmed to 37 °C. The Caco-2 filters were washed with pre-warmed HBSS prior to the experiment, and the solution of **30** was thereafter applied on the apical or basolateral side of the monolayer. Permeability was determined at 37 °C and at pH 7.4 in both the apical and basolateral chamber, with a stirring rate of 500 rpm. The receiver compartment was sampled at 15, 30 and 60 min. At 60 min a final sample from the donor chamber was taken in order to calculate the mass balance of the compound. The samples (100 µl) were transferred to a 96-well plate containing 100 µl methanol and Warfarin as internal standard and were sealed until LC-MS/MS analysis.

Blood-brain distribution. Measurements of blood-brain distribution in rats were performed by Admescope (Finland). The male Sprague Dawley rats (120-157 g) were housed in air-conditioned rooms (12h dark/light cycle) at 22 ˚C and a humidity of 55%. Animals were allowed to acclimatize to the site for at least five days prior to the study.

Compound **30** (24 mg/kg, formulated in 17% DMSO in saline) was administered intraperitoneally and blood samples were collected at 10, 30, and 50 min after dosing. Within 30 min following the sampling, the blood was centrifuged for plasma separation (room temperature; 10 min; 2700 G). The plasma samples were transferred into plastic tubes, frozen and stored at –20˚C until analysis. Brains were perfused under terminal isoflurane anaesthesia at 10, 30 and 50 min after dosing (n=6 per time point). For the perfusion, the heart of the rat was exposed and major veins leading to the right atrium were severed. Approximately 10-20 ml of refrigerated saline was infused into the heart via a blunt needle inserted into the left ventricle, allowing all the blood to be removed from the body via the severed veins. The brain was then immediately collected, frozen in dry ice and stored at –20˚C until analysis.

The plasma samples were prepared by mixing 30 μL of plasma sample with 90 μL of internal standard solution (100 ng/ml of Verapamil and Midazolam). The samples were mixed for 3 min (1000 rpm), centrifuged for 20 min at 4000 rpm, and then submitted to LC-MS analysis. The standard samples were prepared into rat plasma by spiking the matrix into concentrations 1 – 5000 ng/ml of the analyte, respectively, and otherwise treated identically to the samples. Quality control samples were prepared into rat plasma by spiking the matrix into concentrations of 3, 30 and 150 ng/ml of the analyte, respectively, and otherwise treated identically to the samples. Prior to bioanalysis the brains were homogenized 1 + 4 (m + v) in PBS. The brain samples were prepared by mixing 30 μL of brain homogenate with 90 μL of

internal standard solution (100 ng/ml of Verapamil and Midazolam). The samples were mixed for 3 min (1000 rpm), centrifuged for 20 min at 4000 rpm, and then submitted to LC-MS analysis. The standard samples were prepared into brain by spiking the matrix into concentrations 2-2000 ng/ml of the analyte, respectively, and otherwise treated identically to the samples. Quality control samples were prepared into brain by spiking the matrix into concentrations of 3, 30 and 300 ng/ml of the analyte, respectively, and otherwise treated identically to the samples.

Evaluation of compound 30 in rodent model of Parkinsonism. Male Sprague Dawley rats (150-200 g) were housed in air-conditioned rooms (12h dark/light cycle) at 20°C and a humidity of 53%. Experiments were performed in agreement with the European Communities Council Directive of 24 November 1986 (86/609/EEC) on the ethical use of animals and were approved by the local ethical committee (Stockholm North Ethical Committee, N105/16). Rats were anesthetized with ketamine (100 mg/kg, IP; Intervet)/xylazine (5 mg/kg, IP; Bayer, Kiel, Germany), pretreated with desipramine (25 mg/kg, IP; Sigma, St Louis, MO, USA)/pargyline (5 mg/kg, IP; Sigma), placed in a stereotaxic instrument and injected with 6-OHDA (2.5 µl of a 5mg/ml solution; Sigma) into the median forebrain bundle (MFB) of the right hemisphere (AP -2.8 mm, ML -2.0 mm and V -9.0 mm). Two weeks after the unilateral 6-OHDA lesion, rats were injected with apomorphine (1 mg/kg, IP; Sigma) and their contralateral rotations were measured to determine the degree of nigrostriatal denervation. Four weeks after surgery, rats that were divided into two group according to the number of apomorphine-induced rotations. The rats were treated either with DMSO(17%)/saline (n=6) or compound **30** (n=7, 24 mg/kg, IP) dissolved in DMSO(17%)/saline. The number of rotations was calculated automatically with Noldus EthoVision XT11.5 Software for 30 min following the drug administration. The two

groups were then compared by using a Mann-Whitney test. A second set of independent experiments using a new set of rats were carried out using the same protocol to assess the involvement of the D_2R in the observed effect. In this case, rats were divided into four groups according to the number of apomorphine-induced rotations. The rats were treated with either DMSO(17%)/saline (n=6), raclopride (n=7, 2mg/kg, IP) dissolved in DMSO(17%)/saline, compound **30** (n=7, 24 mg/kg, IP) dissolved in DMSO(17%)/saline or the combination of raclopride (30 min pretreatment) and compound **30** (n=7). The rotation test was then performed for 30 min. The four groups were then compared by using One-way ANOVA followed by Tukey's multiple comparisons test.

Supplementary Figures

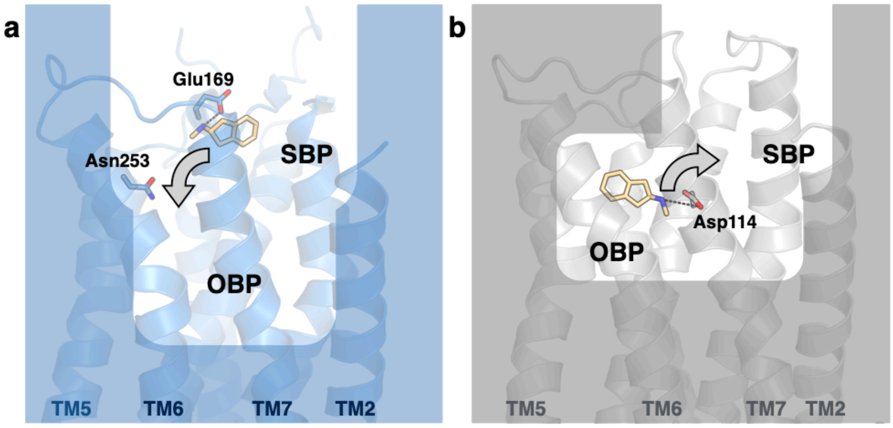

Supplementary Fig. 1. Predicted binding mode of N-methyl-2-aminoindane in the $A_{2A}AR$ and D_2R binding sites. N-methyl-2-aminoindane was docked to a secondary binding pocket (SBP) of the $A_{2A}AR$ (PDB code: 3PWH[12]) and to the orthosteric site (OBP) of the D_2R homology model. The $A_{2A}AR$ and D_2R are shown as blue and grey cartoons, respectively. Key binding site residues and ligands are shown as sticks. The grey arrows show how the compound could be elaborated to obtain a dual-target $A_{2A}AR/D_2R$ ligand.

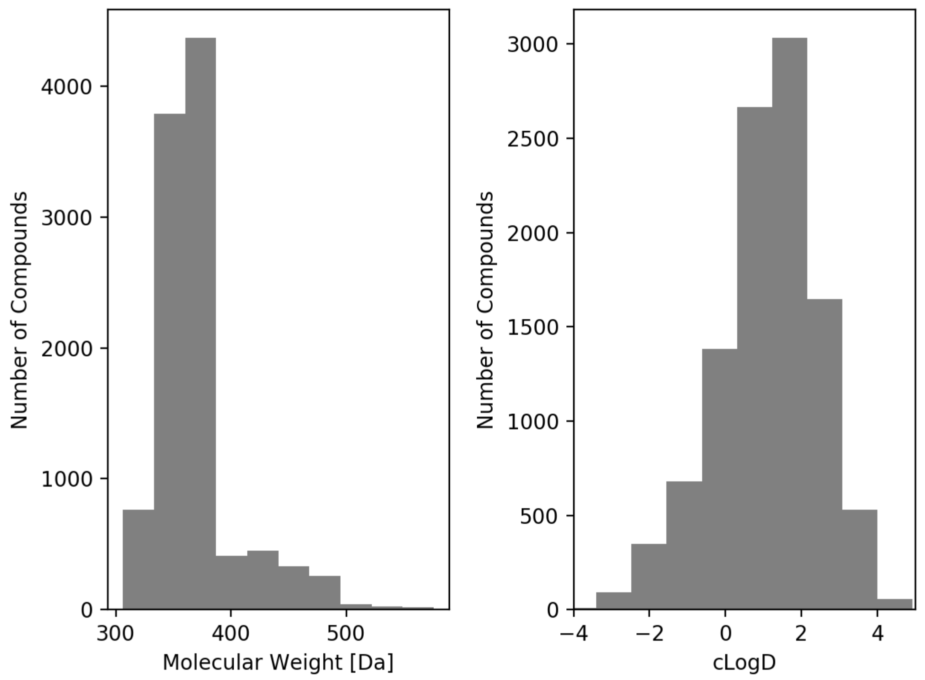

Supplementary Fig. 2. Distributions of molecular weight and cLogD (pH = 7.4) in the virtual chemical library.

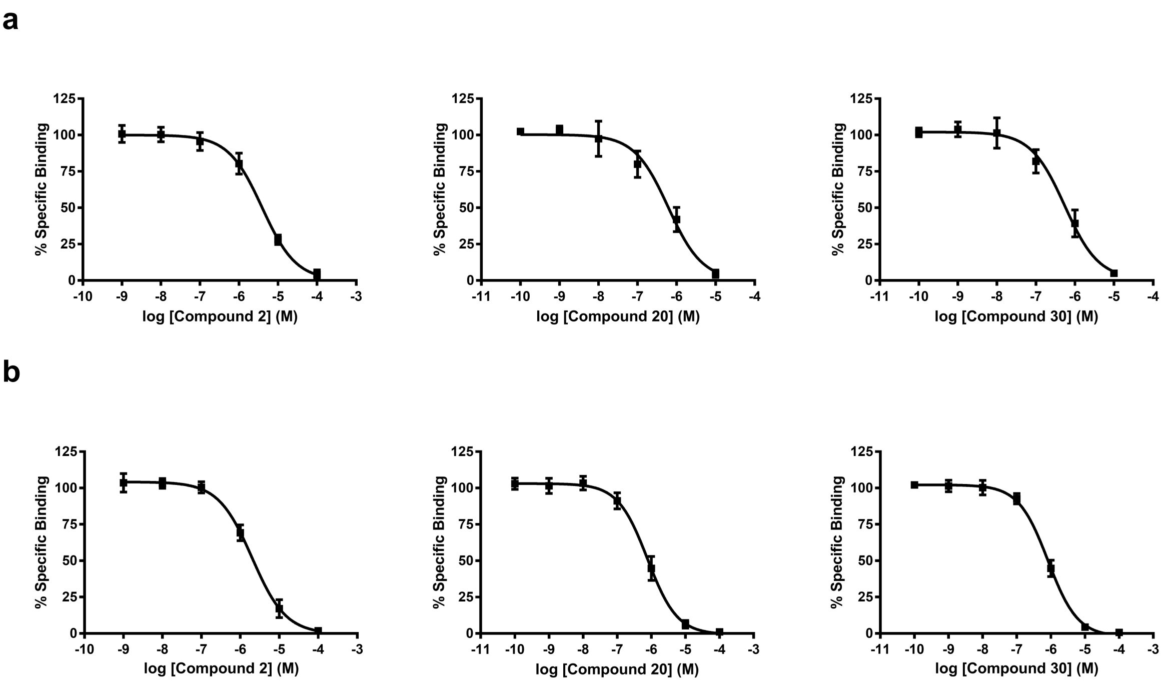

Supplementary Fig. 3. Binding assays for compounds **2**, **20** and **30** at the A_{2A}AR and D_2R. (a) Concentration response curves of compounds at the A_{2A}R labelled with [^{3}H]-ZM241385. (b) Concentration response curves of compounds at the D_2R labelled with [^{3}H]-spiperone. Points represent the mean±SD (vertical bars) of three independent experiments.

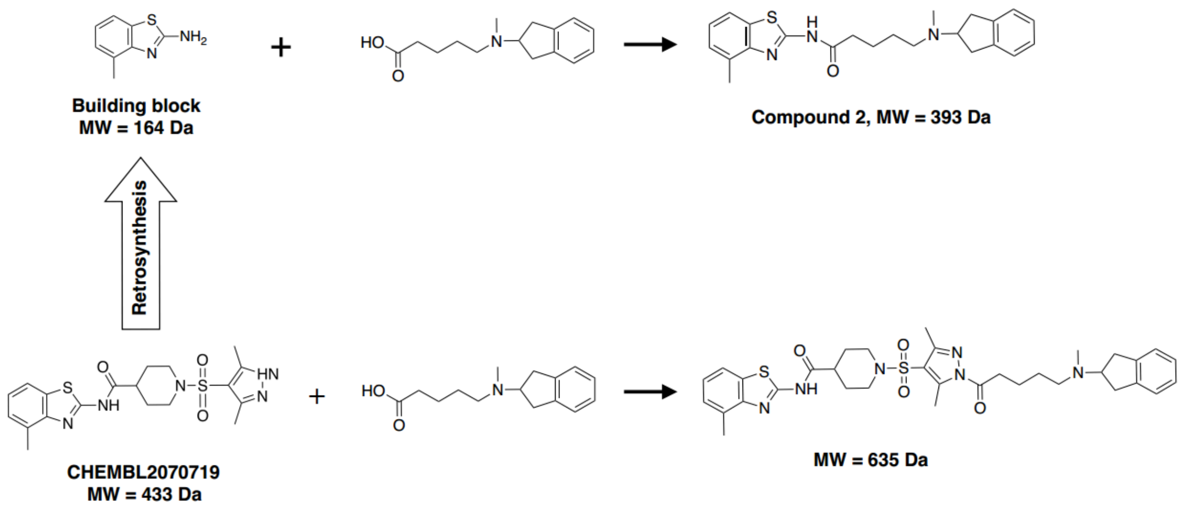

Supplementary Fig. 4. Design of compound **2** using a retrosynthesis approach. Compound **2** was designed in two steps: *In silico* retrosynthesis of $A_{2A}AR$ ligand CHEMBL2070719[27] resulted in the building block 2-amino-4-methyl-benzothiazole, which was followed by creation of compound **2** (MW = 393 Da) by amide coupling. This approach reduced the molecular weight (MW) of the compounds in the virtual library compared to directly using $A_{2A}AR$ ligands in design of dual-target compounds, *e.g.* CHEMBL2070719 (MW = 645 Da).

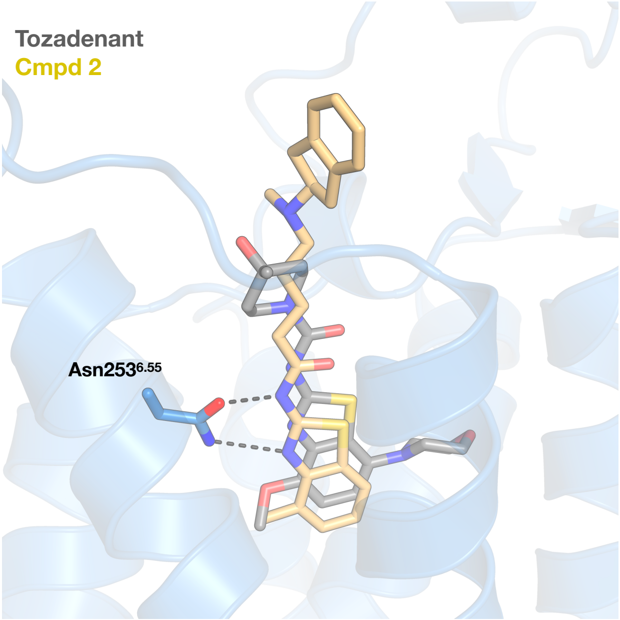

Supplementary Fig. 5. Comparison of predicted binding mode of compound **2** and Tozadenant (PDB code: 5OLO[15]). The receptor is shown as blue cartoons and key binding site residues and ligands are shown as sticks.

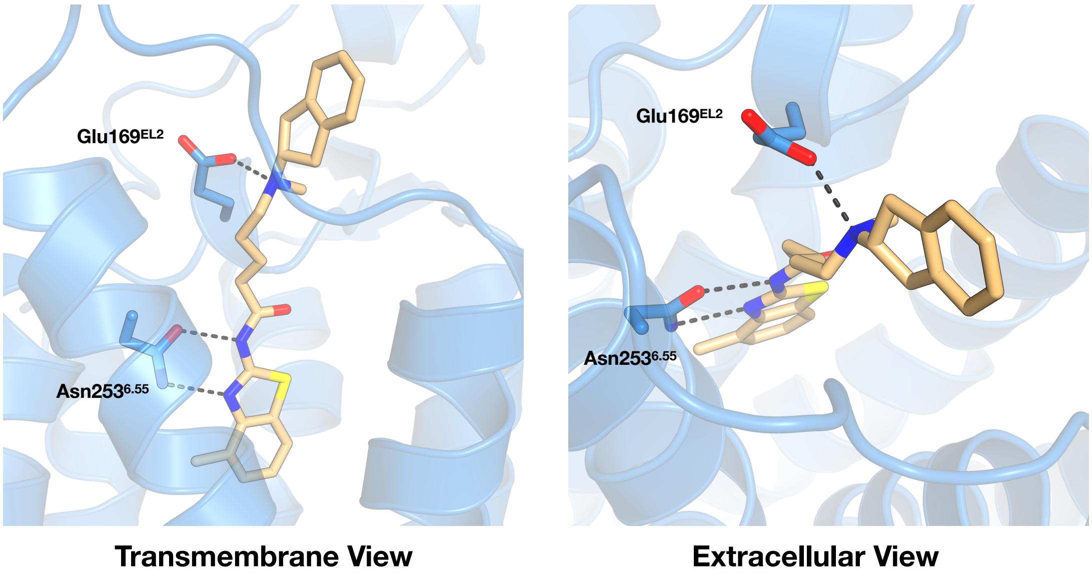

Supplementary Fig. 6. MD simulation refined binding mode of compound **2** in the $A_{2A}AR$ binding site. The receptor is shown as blue cartoons and key binding site residues and the ligand are shown as sticks.

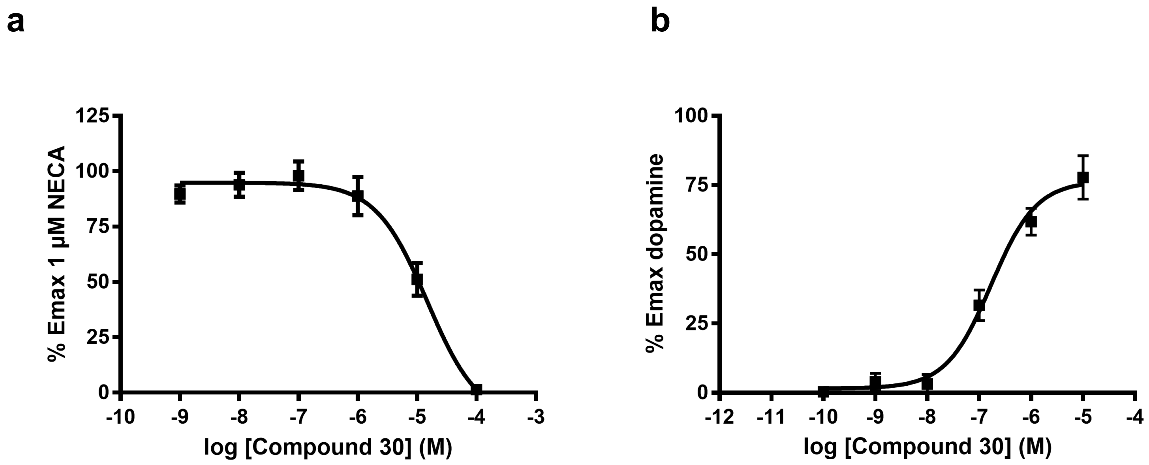

Supplementary Fig. 7. Functional assays of compound **30** at the $A_{2A}AR$ and D_2R. (a) Concentration–response curves of compound **30** based on measuring inhibition of NECA-elicited (1 μM) cAMP formation via the $A_{2A}AR$. (b) Concentration–response curves of compound **30** in functional (HTRF) assays at the D_2R. Points represent the mean ± SD (vertical bars) of three independent experiments.

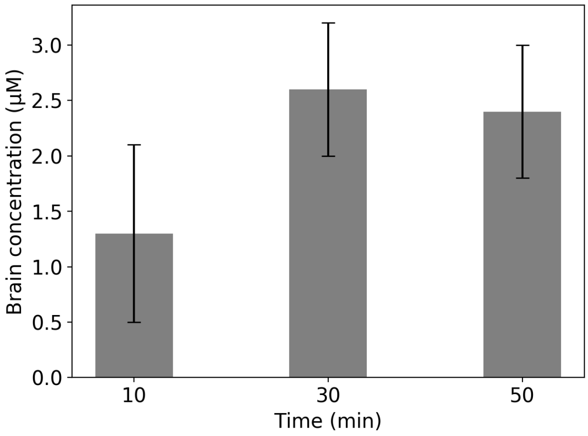

Supplementary Fig. 8. Brain concentration of compound **30** from *in vivo* pharmacokinetics experiments. Data represent mean ± SD.

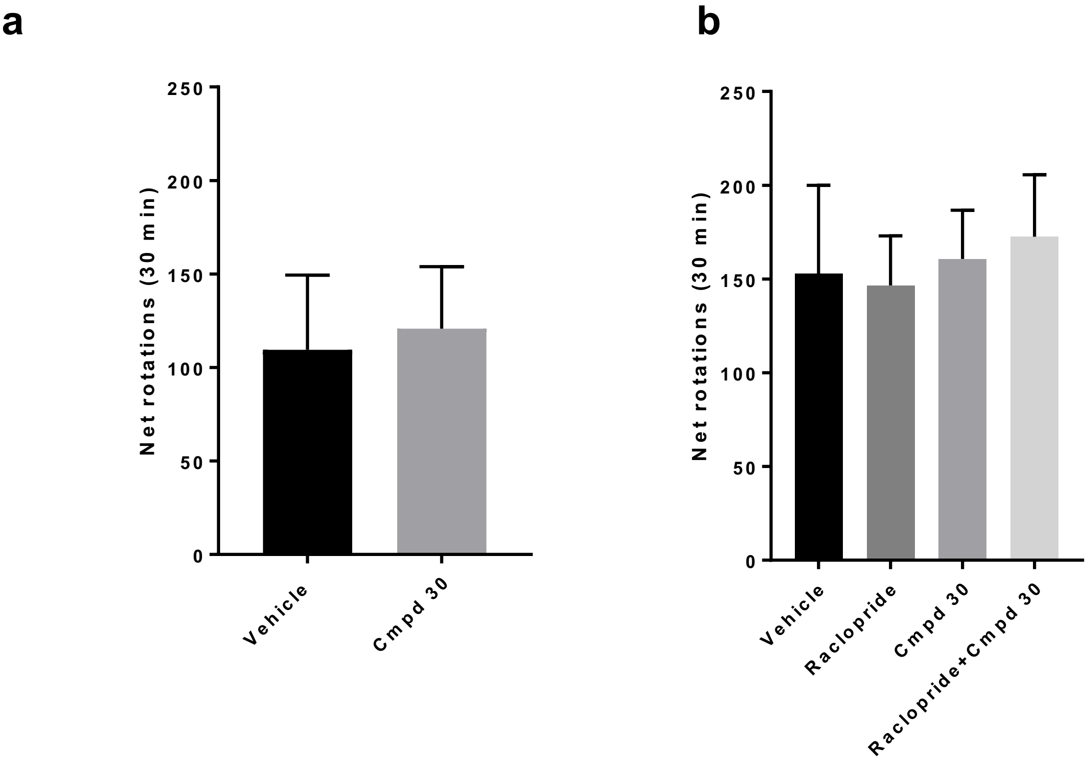

Supplementary Fig. 9. Evaluation of control (apomorphine) in rat model of Parkinsonism. (a) Bar graph showing the number of net rotations (contralateral-ipsilateral rotation count) induced by apomorphine of the rats treated with DMSO(17%)/saline (vehicle, n=6) or compound **30** (n=7, 24 mg/kg, IP) dissolved in DMSO(17%)/saline. Data represent mean ± SEM. These experiments were carried out for the rats used to generate the data in Fig. 4b. (b) Bar graph showing the number of net rotations (contralateral-ipsilateral rotation count) induced by apomorphine of the rats treated with DMSO(17%)/saline (n=6), Raclopride (n=7, 2 mg/kg, IP), compound **30** (n=7, 24 mg/kg, IP) or the combination of raclopride and compound 30 (n=7) dissolved in DMSO(17%)/saline. Data represent mean ± SEM. These experiments were carried out for the rats used to generate the data in Fig. 4c.

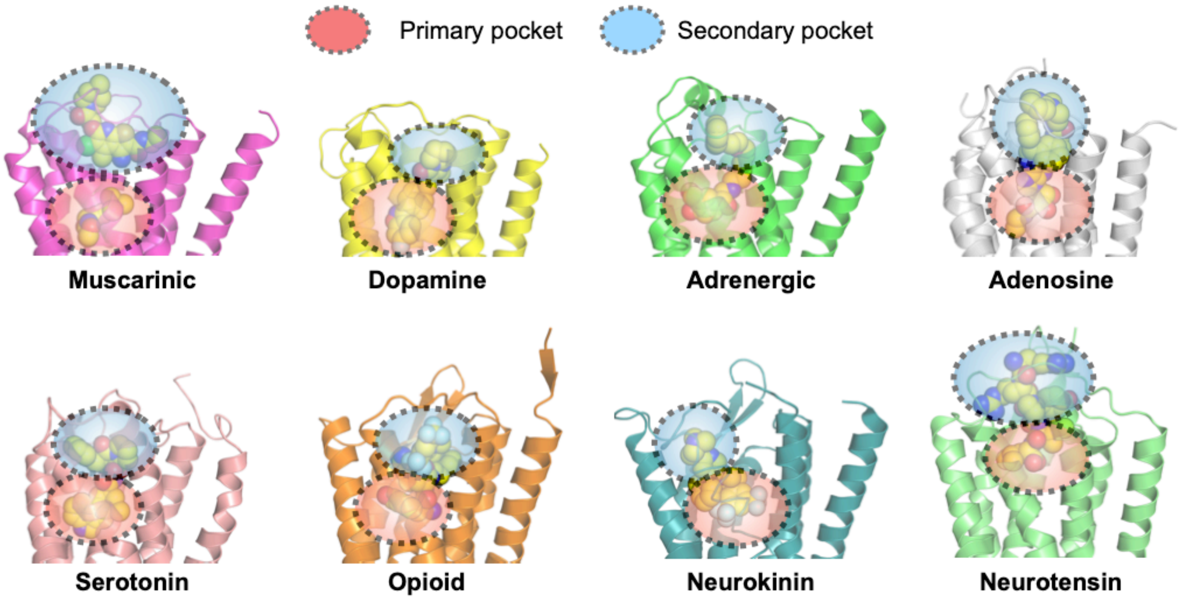

Supplementary Fig. 10. Class A GPCRs contain several distinct (primary and secondary) binding pockets that could be targeted in design of ligand polypharmacology: M_2 Muscarinic (PDB code: 4MQT), D_2 dopamine (PDB code: 6CM4), β_2 adrenergic (PDB code: 6MXT), A_{2A} adenosine (PDB code: 3QAK), 5-HT_{2C} serotonin (PDB code: 6BQG), δ Opioid (PDB code: 6PT2), Neurokinin 1 (PDB code: 6HLP), and Neurotensin 1 (PDB code: 4GRV) receptors. Receptors are shown as cartoons and co-crystallized ligands as spheres.

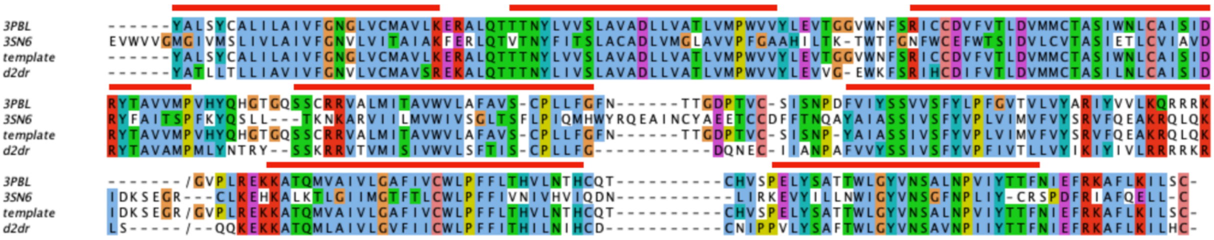

Supplementary Fig. 11. Multiple sequence alignment of the chimeric template (D_3R/β_2AR) to the D_2R sequence. The crystal structure of D_3R (PDB code: 3PBL[7]) was used to model all loop segments and the transmembrane region except for TM5 which was based on the structure of the β_2 adrenergic receptor (PDB code: 3SN6[8]). The predicted TM segments of D_2R are represented by red horizontal lines.

Supplementary Tables

Supplementary Table 1. Distribution of ligand charge for the A_{2A}AR and D_2R (pH = 7.4).

	**Charge (%)**[a]	
	**Q ≤ 0**	**Q > 0**
**A_{2A}AR**	90	10
**D_2R**	14	86

[a]A_{2A}AR and D_2R ligands (pChEMBL value ≥ 5; 4175 and 6198 compounds, respectively) were extracted from the ChEMBL database and the major protonation state at pH = 7.4 was predicted using Chemaxon's cxcalc (JChemSuite version 17.8.0, 2017, ChemAxon, www.chemaxon.com).

Supplementary Table 2. Binding data for the predicted dual-target ligands.

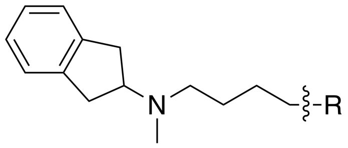

5-11

Cmpd	R	$A_{2A}AR$[a] (*% displacement at 10 μM*)	D_2R[a] (K_i, μM)
**5**[b]	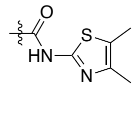	*3 ± 2%*	0.68 ± 0.05
**6**[b]	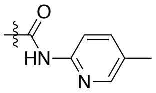	*7 ± 4%*	0.67 ± 0.06
**7**[c]	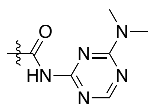	*4 ± 3%*	0.73 ± 0.07
**8**[c]	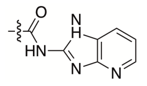	*5 ± 1%*	0.08 ± 0.01
**9**[c]	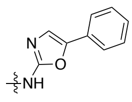	*9 ± 6%*	0.29 ± 0.03
**10**[c]	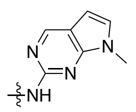	*8 ± 3%*	0.07 ± 0.07
**11**[c]	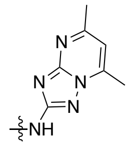	*4 ± 4%*	0.43 ± 0.05

[a] Data represent mean values ± SEM of three individual experiments each performed in duplicate.
[b] Building block for synthesis of dual-target compound identified based on known $A_{2A}AR$ ligand from ChEMBL database.
[c] Building block for synthesis of dual-target compound identified based on commercial chemical library.

Supplementary Table 3. Binding data for analogs of compound **2**.

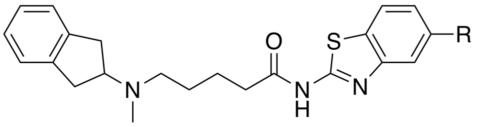

**Cmpd**	**R**	**A_{2A}AR**[a] (*% displacement at 10 μM*)
**14**	Cl	*4 ± 3%*
**15**	OCH_3	*49 ± 8%*
**16**	F	*29 ± 6%*
**17**	Br	*24 ± 3%*

[a] Data represent mean values ± SEM of three individual experiments each performed in duplicate.

Supplementary Table 4. Calculated properties relevant for blood-brain barrier penetration of designed compounds and CNS drugs. Molecular weight (MW), solvent accessible area (TPSA), number of hydrogen bond donors (#HBD), and cLogD (pH = 7.4) are shown for reference compounds and the designed compounds.

**Cmpd**	**MW (Da)**	**TPSA (Å^2)**	**#HBD**	**cLogD (pH 7.4)**
Hitchcock & Pennington[a]	< 500.0	< 90.0	< 3	2.0 – 4.0
A_{2A}AR antagonists[b]	321.1 - 503.2	80.3 - 127.0	0 - 2	1.4 - 2.4
D_2R agonists[c]	203.1 - 653.2	19.0 - 118.2	0 - 3	–1.0 - 4.6
**2**	393.2	45.2	1	3.4
**3**	411.2	56.8	2	3.0
**4**	354.2	50.3	1	1.3
**14**	413.1	45.2	1	3.5
**15**	409.2	54.4	1	3.8
**16**	397.2	45.2	1	3.4
**17**	457.1	45.2	1	3.7
**18**	409.2	54.5	1	2.8
**19**	413.1	45.2	1	3.6
**20**	457.1	45.2	1	3.7
**21**	423.2	54.5	1	3.0
**22**	471.1	45.2	1	4.0
**23**	425.2	45.2	1	3.8
**24**	435.2	45.2	1	4.7
**25**	421.2	45.2	1	4.1
**26**	437.2	54.5	1	3.5
**27**	485.1	45.2	1	4.4
**28**	471.2	54.5	1	4.0
**29**	451.2	54.5	1	3.9
**30**	467.2	63.7	1	3.3
**31**	379.2	45.2	1	2.9
**32**	443.1	45.2	1	3.2
**33**	429.1	45.2	1	3.1
**34**	381.1	54.5	1	2.2
**35**	395.2	54.5	1	2.7
**36**	415.1	54.5	1	2.8
**37**	411.2	63.7	1	2.0
**38**	395.2	54.5	1	2.4
**39**	409.2	54.5	1	2.9
**40**	425.2	63.7	1	2.2
**41**	457.1	45.2	1	3.8

[a] Recommended properties by Hitchcock and Pennington[28]. A property that is within the limits is colored green and red if the value is outside the recommended range.
[b] A_{2A}AR antagonists: Istradefylline, preladenant, tozadenant, vipadenant, ciforadenant[29,30].
[c] D_2R agonists: Rotigotine, aripiprazole, brexpiprazole, lisuride, cabergoline, terguride, roxindole, sumanirole, bromocriptine, apomorphine, pergolide, piribedil, quinpirole, pramipexole, quinelorane, benzquinamide,vilazodone[30].

Supplementary Table 5. Binding data for analogs of compound **2** with modified linker length.

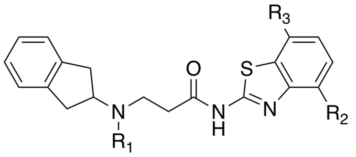

34-41

Cmpd	Structure			Binding affinity[a]	
	**R_1**	**R_2**	**R_3**	**$A_{2A}AR$ (K_i, μM)**	**D_2R (K_i, μM)**
**34**	CH_3	OCH_3	H	1.0 ± 0.1	2.4 ± 0.3
**35**	CH_3	OCH_3	CH_3	0.84 ± 0.09	1.9 ± 0.3
**36**	CH_3	OCH_3	Cl	0.77 ± 0.10	2.7 ± 0.6
**38**	CH_2CH_3	OCH_3	H	2.2 ± 0.3	0.90 ± 0.08
**41**	$CH_2CH_2CH_3$	Br	H	2.1 ± 0.5[b]	1.7 ± 0.3[b]

[a] Data represent mean values ± SEM of three individual experiments each performed in duplicate.
[b] Full curve not obtained due to poor solubility of compound.

Supplementary Table 6. Binding affinities of selected compounds at the A_1AR, D_3R, D_4R, and H_1R.

Cmpd	Binding affinity (µM) or *% displacement at 10 µM*[a]			
	**A_1AR**	**D_3R**	**D_4R**	**H_1R**
**18**	*18 ± 3%*	0.53 ± 0.1	7.6 ± 1.8	0.88 ± 0.1
**20**	2.3 ± 0.4	0.23 ± 0.03	2.9 ± 1.1	0.73 ± 0.14
**22**	2.2 ± 0.3	0.021 ± 0.006	*19 ± 1%*	*12 ± 3%*
**26**	*22 ± 1%*	0.021 ± 0.002	0.70 ± 0.08	1.8 ± 0.5
**27**	*37 ± 4%*	0.027 ± 0.004	*27 ± 4%*	*40 ± 1%*
**30**	*35 ± 1%*	0.056 ± 0.007	*19 ± 4%*	*40 ± 3%*

[a] Data represent mean values ± SEM of two individual experiments each performed in duplicate.

Supplementary Schemes

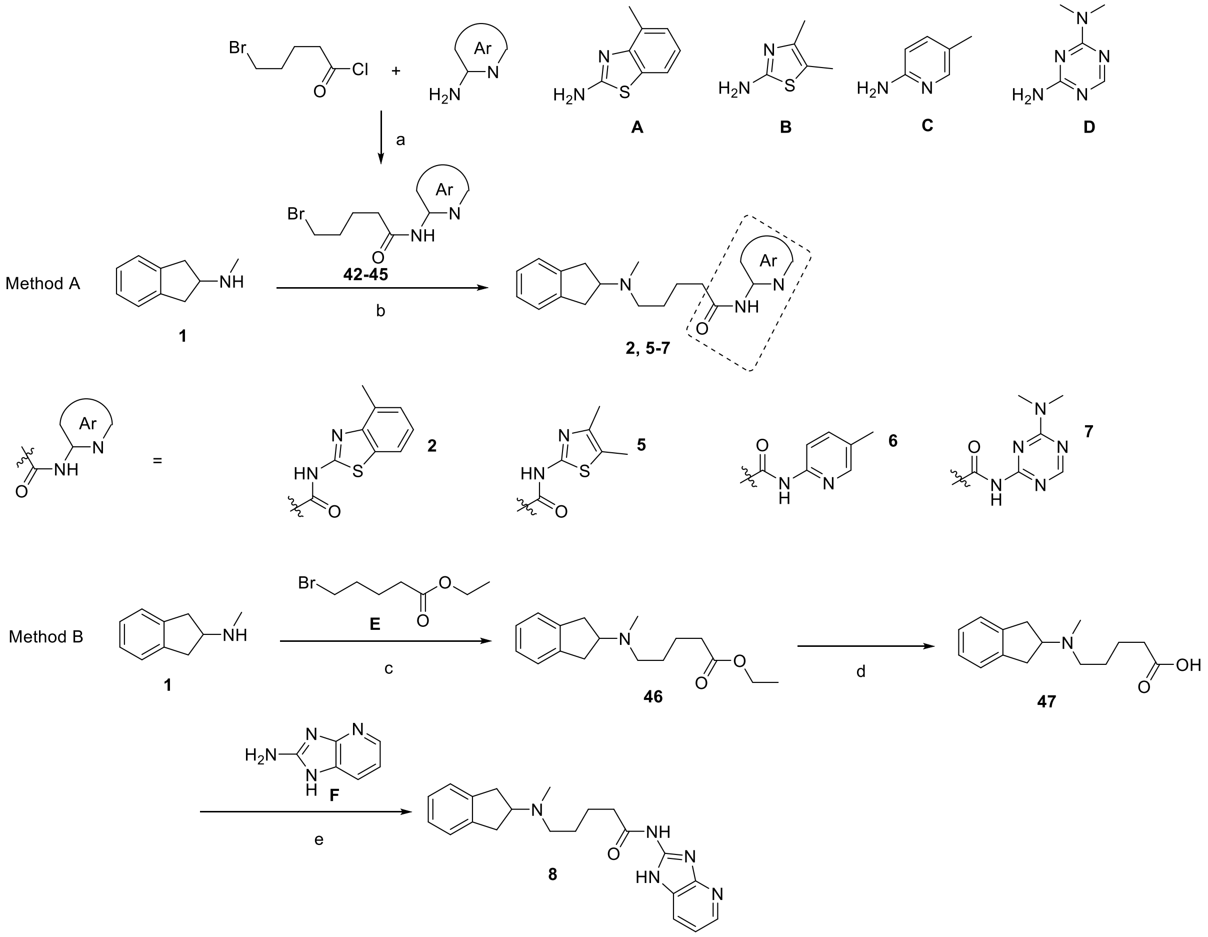

Supplementary Scheme S1. Synthesis of compounds **2**, **5-7** and **8**.

Reagents and conditions: (a) **A-D**, DCM, Et_3N, rt, 59-61%; (b) K_2CO_3, DMF, rt, overnight, 6-65% (hplc); (c) K_2CO_3, DMF, rt, overnight, 70%; (d) KOH, MeOH, 85%; (e) HATU, DIEA, DMF/DCM, overnight, 22% (hplc).

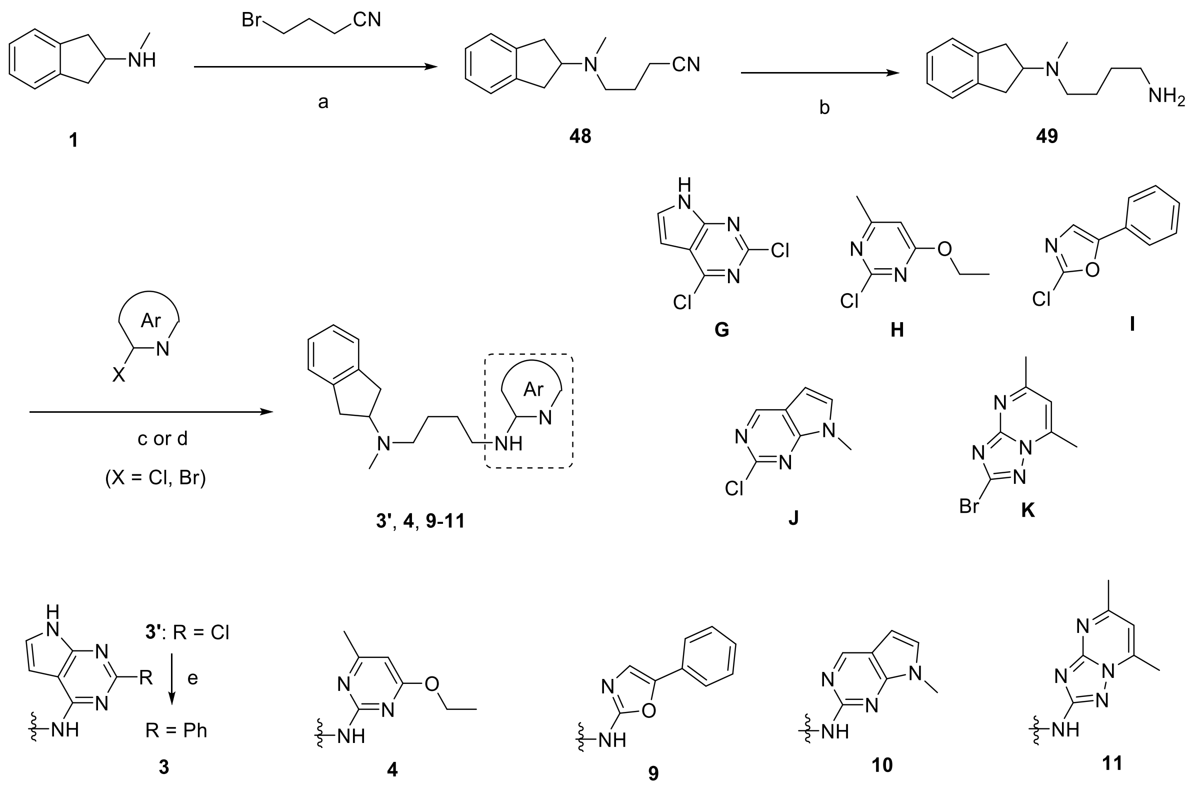

Supplementary Scheme S2. Synthesis of compounds **3**, **4** and **9-11**.

Reagents and conditions: (a) K_2CO_3, CH_3CN, rt, overnight, 24%; (b) $LiAlH_4$, Et_2O, 1 h, 82% (hplc); (c) **G**-**I**, K_2CO_3, CH_3CN, 70-160 °C, 1-4 h, 40-51% (hplc); (d) **J** or **K**, CuI, 1,10-phenantroline, K_2CO_3, DMF, 120 °C, 48 h, 2-28% (hplc); (e) phenyl boronic acid, $Pd(PPh_3)_4$, K_2CO_3, dioxane/H_2O, 100 °C, overnight, 20% (hplc, over 2 steps).

NMR spectra of synthesized compounds

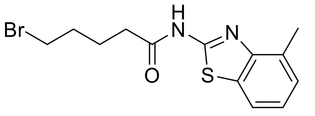

42. 5-bromo-N-(7-methylbenzo[d]thiazol-2-yl)pentanamide.
To a mixture of 4-methyl-1,3-benzothiazol-2-amine (164 mg, 1 mmol, 1 equiv.) and Et_3N (0.167 mL, 1.2 equiv.) in dry DCM (3 mL), was added 4-bromopentanoic acid chloride (0.147 mL, 1.1 mmol, 1 eq.) at 0 °C. The mixture was stirred at room temperature for 1 h. LCMS showed major desired product. After concentration, the crude was purified by silica gel colum chrommatography using dichloromethane:methanol 99:1 to afford a white solid which was used for the next step. Yield: 200 mg (61%). LCMS (ESI+): calculated for $C_{13}H_{16}BrN_2OS$ $(M+H)^+$: 327.0; found 327.1.
^1H NMR (400 MHz, $CDCl_3$) δ 7.68-7.64 (m, 1H), 7.27-7.20 (m, 2H), 3.40 (t, *J* = 6.3 Hz, 2H), 2.48 (t, *J* = 7.2 Hz, 2H), 2.63 (s, 3H), 1.97-1.84 (m, 4H).

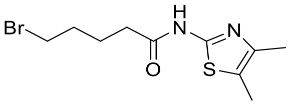

43. 5-bromo-N-(4,5-dimethylthiazol-2-yl)pentanamide.
To a mixture of 4,5-dimethylthiazol-2-amine (128 mg, 1 mmol, 1 equiv.) and Et_3N (0.167 mL, 1.2 equiv.) in dry DCM (3 mL), was added 4-bromopentanoic acid chloride (0.147 mL, 1.1 mmol, 1 eq.) at 0 °C. The mixture was stirred at room temperature for 1 h. LCMS showed major desired product. After concentration, the crude was purified by silica gel colum chrommatography using dichloromethane:methanol 99:1 to afford a white solid which was used for the next step. Yield: 173 mg (59%). LCMS (ESI+): calculated for $C_{10}H_{16}BrN_2OS$ $(M+H)^+$: 291.0; found 291.1.
^1H NMR (400 MHz, $CDCl_3$) δ 3.40 (t, *J* = 6.3 Hz, 2H), 2.44 (t, *J* = 7.2 Hz, 2H), 2.28 (d, *J* = 0.7 Hz, 3H), 2.22 (d, *J* = 0.7 Hz, 3H), 1.99-1.79 (m, 4H).

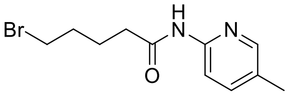

44. 5-bromo-N-(5-methylpyridin-2-yl)pentanamide.
To a mixture of 5-methylpyridin-2-amine (108 mg, 1 mmol, 1 equiv.) and Et_3N (0.167 mL, 1.2 equiv.) in dry DCM (3 mL), was added 4-bromopentanoic acid chloride (0.147 mL, 1.1 mmol, 1 eq.) at 0 °C. The mixture was stirred at room temperature for 1 h. LCMS showed major desired product. After concentration, the crude was purified by silica gel colum chrommatography using dichloromethane:methanol 99:1 to afford a white solid which was used for the next step. Yield: 167 mg (61%). LCMS (ESI+): calculated for $C_{11}H_{16}BrN_2O$ $(M+H)^+$: 271.0; found 271.1.
^1H NMR (400 MHz, $CDCl_3$) δ 8.21 (br s, 1H), 8.10 (d, *J* = 8.5 Hz, 1H), 8.07 (d, *J* = 2.0 Hz, 1H), 7.53 (dd, *J* = 8.5, 2.0 Hz, 1H), 3.43 (t, *J* = 6.3 Hz, 2H), 2.42 (t, *J* = 7.2 Hz, 2H), 2.29 (s, 3H), 1.99-1.83 (m, 4H).

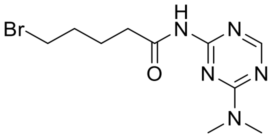

45. 5-bromo-N-(4-(dimethylamino)-1,3,5-triazin-2-yl)pentanamide.

To a mixture of N2,N2-dimethyl-1,3,5-triazine-2,4-diamine (139 mg, 1 mmol, 1 equiv.) and Et_3N (0.167 mL, 1.2 equiv.) in dry DCM (3 mL), was added 4-bromopentanoic acid chloride (0.147 mL, 1.1 mmol, 1 eq.) at 0 °C. The mixture was stirred at room temperature for 1 h. LCMS showed major desired product. The mixture was washed with 0.1 M NaOH (3 mL) solution, H_2O (3 mL) and brine (3 mL) then concentrated to give a crude (247 mg) that was used as it is for the next step without purification. LCMS (ESI+): calculated for $C_{10}H_{17}BrN_5O$ $(M+H)^+$: 302.1; found 302.1.

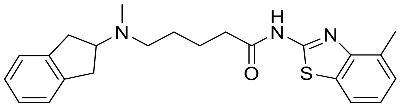

2. 5-((2,3-dihydro-1H-inden-2-yl)(methyl)amino)-N-(4-methylbenzo[d]thiazol-2-yl)pentanamide.
To a mixture of N-Me-2-indane-amine (20 mg, 0.14 mmol, 1 eq.), 5-bromo-N-(4-methyl-1,3-benzothiazol-2-yl)pentanamide (49 mg, 1.1 eq.), K_2CO_3 (38.6 mg, 2 eq.), NaI (21 mg, 1 eq.) in DMF (0.5 mL) was stirred at rt for overnight. LCMS showed product. The solid was filtered off. Purification using hplc 0-60% ACN in H_2O (+ 0.1%TFA) for 30min afforded desired product as TFA salt (4 mg, 6% yield). LCMS (ESI+): calculated for $C_{23}H_{28}N_3OS$ $(M+H)^+$: 390.2; found 390.3.
^{1}H NMR (500 MHz, CD_3OD) δ 7.67-7.65 (m, 1H), 7.26-7.16 (m, 6H), 4.26-4.19 (m, 1H), 3.52-3.16 (m, 6H), 2.88 (s, 3H), 2.64 (t, *J* = 6.5 Hz, 2H), 2.60 (s, 3H), 1.96-1.79 (m, 4H).
^{13}C NMR (125 MHz, CD_3OD) δ 173.4, 158.2, 149.2, 140.0, 133.2, 131.9, 128.8, 127.8, 125.6, 124.9, 119.7, 67.1, 55.6, 38.4, 36.2, 35.7, 35.4, 24.8, 22.8, 18.1.

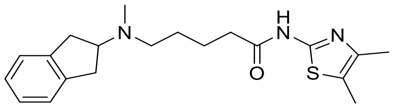

5. 5-((2,3-dihydro-1H-inden-2-yl)(methyl)amino)-N-(4,5-dimethylthiazol-2-yl)pentanamide.
To a mixture of N-Me-2-indane-amine (20 mg, 0.14 mmol, 1 eq.), 5-bromo-N-(4,5-dimethylthiazol-2-yl)pentanamide (43.5 mg, 1.1 eq.), K_2CO_3 (38.6 mg, 2 eq.), NaI (21 mg, 1 eq.) in DMF (0.5 mL) was stirred at rt for overnight. LCMS showed desired product. The solid was filtered off. Purification using hplc 0-60% ACN in H_2O (+ 0.1%TFA) for 30 min afforded desired product as TFA salt (10 mg, 15% yield). LCMS (ESI+): calculated for $C_{20}H_{28}N_3OS$ $(M+H)^+$: 358.2; found 358.3.
^{1}H NMR (400 MHz, $CDCl_3$) δ 12.27 (br s, 1H), 7.24-7.19 (m, 4H), 4.18-4.08 (m, 1H), 3.38 (d, *J* = 7.1 Hz, 2H), 3.32 (d, *J* = 7.1 Hz, 2H), 3.31-3.21 (m, 1H), 3.07-2.95 (m, 1H), 2.73 (s, 3H), 2.67 (t, *J* = 6.6 Hz, 2H), 2.34 (s, 3H), 2.32 (s, 3H), 1.99-1.76 (m, 4H).
^{13}C NMR (100 MHz, $CDCl_3$) δ 171.2, 159.2, 138.6, 138.3, 132.4, 127.8, 127.7, 124.5, 124.4, 120.0, 65.2, 54.0, 36.9, 34.9, 34.8, 33.7, 23.1, 21.8, 11.5, 10.7.

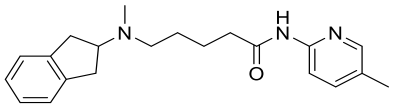

6. 5-((2,3-dihydro-1H-inden-2-yl)(methyl)amino)-N-(5-methylpyridin-2-yl)pentanamide.
To a mixture of N-Me-2-indane-amine (20 mg, 0.14 mmol, 1 eq.), 5-bromo-N-(5-methyl-2-pyridyl)pentanamide (40 mg, 1.1 eq.), K_2CO_3 (38.6 mg, 2 eq.), NaI (21 mg, 1 eq.) in DMF (0.5 mL) was stirred at rt for overnight. LCMS showed major product. The solid was filtered off. Purification using hplc 0-60% ACN in H_2O (+ 0.1%TFA) for 30 min afforded desired product as TFA salt (41 mg, 65% yield). LCMS (ESI+): calculated for $C_{21}H_{28}N_3O$ $(M+H)^+$: 338.2; found 338.3.

^{1}H NMR (400 MHz, $CDCl_3$) δ 12.81 (br s, 1H), 11.61 (br s, 1H), 8.43 (d, *J* = 8.9 Hz, 1H), 8.03-7.98 (m, 2H), 7.23-7.17 (m, 4H), 4.14-4.07 (m, 1H), 3.40-3.20 (m, 5H), 3.09-3.03 (m, 1H), 2.73 (s, 3H), 2.62 (t, *J* = 6.6 Hz, 2H), 2.41 (s, 3H), 1.94-1.72 (m, 4H).
^{13}C NMR (100 MHz, $CDCl_3$) δ 173.0, 147.2, 146.8, 138.5, 138.3, 136.5, 130.3, 127.7, 127.6, 124.5, 124.4, 116.9, 65.3, 54.2, 37.0, 35.6, 34.9, 33.7, 23.0, 21.8, 17.6.

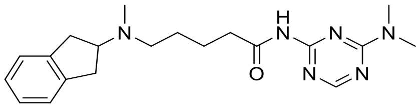

7. 5-((2,3-dihydro-1H-inden-2-yl)(methyl)amino)-N-(4-(dimethylamino)-1,3,5-triazin-2-yl)pentanamide.
To a mixture of N-Me-2-indane-amine (88 mg, 2 eq.), 5-bromo-N-(4,5-dimethylthiazol-2-yl)pentanamide (91 mg, 0.3 mmol, 1 eq.), K_2CO_3 (83 mg, 2 eq.), NaI (45 mg, 1 eq.) in DMF (0.5 mL) was stirred at rt for overnight. LCMS showed desired product. The solid was filtered off. Purification using hplc 0-50% ACN in H_2O (+ 0.1%TFA) for 30 min afforded desired product as TFA salt (22 mg, 15% yield). LCMS (ESI+): calculated for $C_{20}H_{29}N_6O$ $(M+H)^+$: 369.2; found 369.3.
^{1}H NMR (400 MHz, $CDCl_3/CD_3OD$) δ 8.40 (s, 1H), 7.22-7.12 (m, 4H), 4.11-4.03 (m, 1H), 3.36-3.18 (m, 5H), 3.29 (s, 3H), 3.24 (s, 3H), 3.05-2.93 (m, 1H), 2.69 (s, 3H), 2.65 (t, *J* = 6.7 Hz, 2H), 1.86-1.70 (m, 4H).
^{13}C NMR (100 MHz, $CDCl_3/CD_3OD$) δ 174.3, 173.4, 162.3, 157.8, 157.7, 155.3, 155.2, 154.5, 138.4, 127.7, 127.6, 124.4, 65.1, 64.9, 54.0, 53.9, 37.3, 37.2, 37.0, 36.9, 36.7, 35.8, 32.8, 23.1, 23.0, 21.7, 21.1.

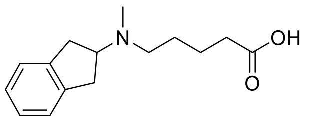

47. 5-((2,3-dihydro-1H-inden-2-yl)(methyl)amino)pentanoic acid.

Step 1
To a mixture of N-Me-2-indane-amine (2.0 g, 13.6 mmol, 1 eq.), ethyl 5-bromopentanoate (3.2 mL, 1.5 eq.), K_2CO_3 (3.79 g, 2 eq.), in ACN (20 mL) was stirred at 60 ^{o}C for overnight. LCMS showed major desired product. The mixture was filtered. After removing of solvent, the crude was purified by silica gel column using PE:EA 7:3 + 0.5% Et_3N to afford desired product ethyl 5-((2,3-dihydro-1H-inden-2-yl)(methyl)amino)pentanoate as a yellow oil (2.6 g, 70% yield). LCMS (ESI+): calculated for $C_{17}H_{26}NO_2$ $(M+H)^+$: 276.2; found 276.3.
^{1}H NMR (400 MHz, $CDCl_3$) δ 7.22-7.10 (m, 4H), 4.13 (q, *J* = 7.1 Hz, 2H), 3.39-3.28 (m, 1H), 3.05 (dd, *J* = 15.1, 7.5 Hz, 2H), 2.89 (dd, *J* = 15.3, 8.7 Hz, 2H), 2.48 (t, *J* = 7.5 Hz, 2H), 2.34 (d, *J* = 7.2 Hz, 2H), 2.27 (s, 3H), 1.71-1.51 (m, 4H), 1.25 (t, *J* = 7.1 Hz, 3H).
^{13}C NMR (100 MHz, $CDCl_3$) δ 173.6, 141.7, 126.3, 124.4, 66.2, 60.2, 55.0, 39.4, 36.9, 34.2, 26.4, 23.0, 14.2.

Step 2
To a solution of ethyl 5-[indan-2-yl(methyl)amino]pentanoate (2.6 g, 9.4 mmol, 1 eq.) in MeOH (20 mL) was added KOH (1.06 g, 2 eq.). The mixture was heated at 60 ^{o}C for 2 h. LCMS showed complete ester hydrolysis. MeOH was removed and the mixture was diluted with ethyl acetate (50 mL), neutralized with concentrated HCl (1 mL) in an ice bath to pH = 1, dried over $MgSO_4$, filtered and concentrated to give a crude. Dissolve the crude with small amount of ACN and 10 times H_2O and freezed dry overnight to eliminate all the trace of

alcohol that can disturb its use in amide coupling. Yield: 2 g, 85%. This material is used as such for the next step without purification. LCMS (ESI+): calculated for $C_{15}H_{22}NO_2$ $(M+H)^+$: 248.2; found 248.2.
^{1}H NMR (400 MHz, $CDCl_3$) δ 7.31-7.20 (m, 4H), 3.99-3.89 (m, 1H), 3.39-3.20 (m, 4H), 2.99-2.92 (m, 2H), 2.57 (s, 3H), 2.30 (t, J = 6.5 Hz, 2H), 1.86-1.56 (m, 4H).
^{13}C NMR (100 MHz, $CDCl_3$) δ 178.0, 139.4, 127.3, 124.4, 64.9, 54.5, 36.6, 35.3, 34.5, 24.1, 22.9.

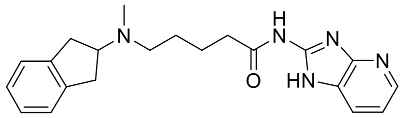

8. 5-((2,3-dihydro-1H-inden-2-yl)(methyl)amino)-N-(1H-imidazo[4,5-b]pyridin-2-yl)pentanamide.
To a mixture of 5-[indan-2-yl(methyl)amino]pentanoic acid (51 mg, 0.2 mmol, 1 eq.), HATU (157 mg, 2 eq.), DIEA (72 µL, 2 eq.) in 3/1 mixture of DCM/DMF (v/v, 4 mL) was stirred for 5 min at rt. Then, 3H-imidazo[4,5-b]pyridin-2-amine (27.7 mg, 1 eq.) was added and the reaction was stirred at rt for overnight. LCMS showed no starting material left. After removal of DCM and filtering, the crude was purified by hplc using 0-60% of ACN in H_2O (+ 0.1% TFA) for 30 min to give desired product as yellow TFA salt solid (21 mg, 22% yield). LCMS (ESI+): calculated for $C_{21}H_{26}N_5O$ $(M+H)^+$: 364.2; found 364.3.
^{1}H NMR (400 MHz, $CDCl_3/CD_3OD$) δ 8.37 (d, J = 5.0 Hz, 1H), 8.08 (d, J = 7.9 Hz, 1H), 7.37 (dd, J = 7.9, 5.0 Hz, 1H), 7.23-7.12 (m, 4H), 4.16-4.06 (m, 1H), 3.40-3.19 (m, 5H), 3.11-2.96 (m, 1H), 2.73 (s, 3H), 2.66 (t, J = 6.7 Hz, 2H), 2.00-1.70 (m, 4H).
^{13}C NMR (100 MHz, $CDCl_3/CD_3OD$) δ 172.9, 149.8, 145.9, 138.6, 138.4, 127.6, 126.4, 124.4, 124.0, 118.4, 65.1, 54.0, 36.8, 35.2, 23.1, 21.4.

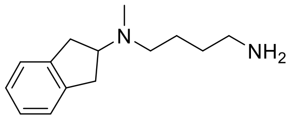

49. N1-(2,3-dihydro-1H-inden-2-yl)-N1-methylbutane-1,4-diamine.

Step 1
To a mixture of N-Me-2-indane-amine (2.5 g, 13.6 mmol, 1 eq.), 4-bromobutyronitrile (2 mL, 1.5 eq.), K_2CO_3 (3.79 g, 2 eq.), in ACN (20 mL) was stirred at 60 °C for overnight. LCMS showed product. The mixture was filtered. After removing of solvent, the crude was purified by silica gel column using PE:EA 7:3 + 0.5% Et_3N to afford desired product 4-[indan-2-yl(methyl)amino]butanenitrile as a yellow oil (700 mg, 24% yield). LCMS (ESI+): calculated for $C_{14}H_{19}N_2$ $(M+H)^+$: 215.2; found 215.2.
^{1}H NMR (400 MHz, $CDCl_3$) δ 7.23-7.09 (m, 4H), 3.40-3.31 (m, 1H), 3.06 (dd, J = 15.3, 7.5 Hz, 2H), 2.88 (dd, J = 15.3, 8.5 Hz, 2H), 2.56 (t, J = 6.7 Hz, 2H), 2.45 (t, J = 7.1 Hz, 2H), 2.24 (s, 3H), 1.90-1.77 (m, 2H).
^{13}C NMR (100 MHz, $CDCl_3$) δ 141.6, 126.4, 124.4, 119.8, 66.3, 53.0, 39.2, 36.9, 23.2, 14.8.

Step 2
To a suspension of $LiAlH_4$ (372 mg, 3 eq.) in Et_2O (25 mL) at 0 °C was added a solution of 4-[indan-2-yl(methyl)amino]butanenitrile (700 mg, 3.3 mmol, 1 eq.) in Et_2O (6 mL). The reaction was stirred at rt for 1 h. LCMS indicated complete reaction. The reaction was cooled down again to 0 °C, diluted with Et_2O (25 mL) and quenched with sat. $NaHCO_3$ (3 mL) and $MgSO_4$ was added. The mixture was then filtered on celite/$MgSO_4$/celite and washed with DCM (40 mL), EA (40 mL). The organic phase was dried over $MgSO_4$ and filtered and

concentrated to give desired product as a yellow oil (585 mg, 82%). This material is used as such for the next step without purification. LCMS (ESI+): calculated for $C_{14}H_{23}N_2$ $(M+H)^+$: 219.2; found 219.3.
^{1}H NMR (400 MHz, $CDCl_3$) δ 7.22-7.08 (m, 4H), 3.39-3.28 (m, 1H), 3.02 (dd, *J* = 15.0, 7.5 Hz, 2H), 2.88 (dd, *J* = 15.4, 8.7 Hz, 2H), 2.73 (t, *J* = 6.7 Hz, 2H), 2.46 (t, *J* = 7.1 Hz, 2H), 2.27 (s, 3H), 1.59-1.43 (m, 4H).
^{13}C NMR (100 MHz, $CDCl_3$) δ 141.7, 126.3, 124.4, 66.2, 55.3, 42.0, 39.4, 36.9, 31.6, 24.4.

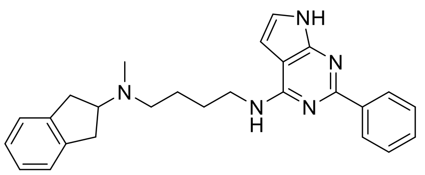

3. N1-(2,3-dihydro-1H-inden-2-yl)-N1-methyl-N4-(2-phenyl-7H-pyrrolo[2,3-d]pyrimidin-4-yl)butane-1,4-diamine.
To a mixture of N'-indan-2-yl-N'-methyl-butane-1,4-diamine (36 mg, 1.1 eq.), 2,4-dichloro-7H-pyrrolo[2,3-d]pyrimidine (28 mg, 0.15 mmol, 1 eq.), K_2CO_3 (42 mg, 2 eq.) in ACN (2 mL) was stirred at 160 °C for 1h. After cooling down and filtered and removing of solvent, the crude was used as such for the next step of Suzuki coupling without purification. LCMS showed full conversion. The mixture of crude N-(2-chloro-7H-pyrrolo[2,3-d]pyrimidin-4-yl)-N'-indan-2-yl-N'-methyl-butane-1,4-diamine (0.15 mmol, 1 eq.), phenyl boronic acid (37 mg, 2 eq.), $Pd(PPh_3)_4$ (8.7 mg, 0.05 eq.), K_2CO_3 (42 mg, 2 eq.) in 2/1 mixture of dioxane/H_2O (6 mL) was heated at 100 °C for overnight. LCMS showed major desired product. The crude was filtered and purified by hplc using 0-60% ACN in H_2O (+ 0.1% TFA) for 30 min to afford desired product as yellow TFA salt material (16 mg, 20% yield, 2 steps). LCMS (ESI+): calculated for $C_{26}H_{30}N_5$ $(M+H)^+$: 412.2; found 412.3.
^{1}H NMR (400 MHz, $CDCl_3$) δ 13.73 (br s, 1H), 12.14 (br s, 1H), 11.79 (br s, 1H), 8.30-8.05 (m, 2H), 7.57-7.40 (m, 2H), 7.24-7.11 (m, 4H), 6.88-6.82 (m, 1H), 7.08-7.04 (m, 1H), 6.78-6.70 (m, 1H), 4.08-3.98 (m, 1H), 3.93-3.60 (m, 2H), 3.32-3.06 (m, 5H), 3.01-2.86 (m, 1H), 2.70 (s, 3H), 2.11-1.73 (m, 4H).
^{13}C NMR (100 MHz, $CDCl_3$) δ 156.8, 156.1, 152.2, 140.4, 138.2, 132.4, 131.5, 129.5, 129.1, 128.1, 127.8, 124.5, 124.4, 123.3, 120.2, 115.3, 106.6, 102.6, 100.5, 65.6, 53.8, 39.8, 36.9, 34.8, 33.8, 25.4, 21.6.

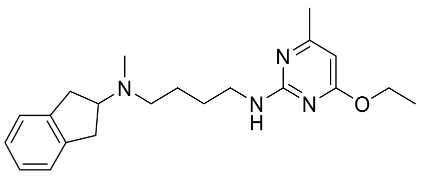

4. N1-(2,3-dihydro-1H-inden-2-yl)-N4-(4-ethoxy-6-methylpyrimidin-2-yl)-N1-methylbutane-1,4-diamine.
To a mixture of N'-indan-2-yl-N'-methyl-butane-1,4-diamine (17.5 mg, 1.1 eq.), 2-chloro-4-ethoxy-6-methyl-pyrimidine (12.5 mg, 0.07 mmol, 1 eq.), K_2CO_3 (20 mg, 2 eq.) in DMF (1 mL) was stirred at 160 °C for 1h. After cooling down and filtered, the crude was purified by hplc using 0-60% ACN in H_2O (+ 0.1 % TFA) for 30 min to afford desired product as yellow TFA salt solid (13 mg, 40% yield). LCMS (ESI+): calculated for $C_{21}H_{31}N_4O$ $(M+H)^+$: 355.2; found 355.3.
^{1}H NMR (400 MHz, $CDCl_3$) δ 12.08 (br s, 1H), 10.32 (br s, 1H), 7.24-7.18 (m, 4H), 5.93 (s, 1H), 4.46 (q, *J* = 7.1 Hz, 2H), 4.19-4.11 (m, 1H), 3.58-3.03 (m, 8H), 2.72 (s, 3H), 2.39 (s, 3H), 2.00-1.65 (m, 4H), 1.41 (t, *J* = 7.1 Hz, 3H).

^{13}C NMR (100 MHz, $CDCl_3$) δ 172.0, 157.8, 156.0, 138.7, 138.5, 127.7, 127.6, 124.5, 124.4, 97.3, 65.0, 64.5, 53.8, 40.4, 36.4, 34.9, 33.5, 25.4, 21.3, 18.7, 13.9.

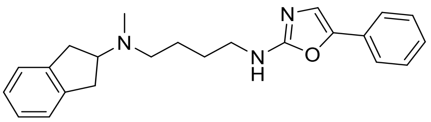

9. N1-(2,3-dihydro-1H-inden-2-yl)-N1-methyl-N4-(5-phenyloxazol-2-yl)butane-1,4-diamine. To a mixture of N'-indan-2-yl-N'-methyl-butane-1,4-diamine (35 mg, 1 eq.), 2-chloro-5-phenyloxazole (26 mg, 0.14 mmol, 1 eq.), K_2CO_3 (42 mg, 4 eq.) in DMF (1 mL) was stirred at 70 °C for 4h. LCMS showed major desired product. After cooling down and filtered, the crude was purified by hplc using 0-60% ACN in H_2O (+ 0.1 % TFA) for 30 min to afford desired product as TFA salt (34 mg, 51% yield). LCMS (ESI+): calculated for $C_{23}H_{28}N_3O$ $(M+H)^+$: 362.2; found 362.3.
^1H NMR (400 MHz, $CDCl_3$) δ 12.64 (br s, 1H), 11.02 (br s, 1H), 7.55-7.36 (m, 5H), 7.22-7.16 (m, 4H), 7.07 (s, 1H), 4.20-4.11 (m, 1H), 3.64-2.98 (m, 8H), 2.74 (s, 3H), 2.10-1.71 (m, 4H).
^{13}C NMR (100 MHz, $CDCl_3$) δ 157.8, 145.5, 138.5, 138.6, 129.6, 129.2, 127.7, 125.1, 124.4, 123.6, 109.6, 65.0, 53.6, 41.9, 36.5, 34.9, 33.6, 25.9, 21.1.

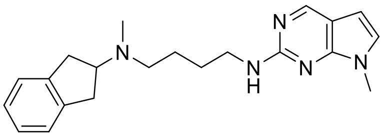

10. N1-(2,3-dihydro-1H-inden-2-yl)-N1-methyl-N4-(7-methyl-7H-pyrrolo[2,3-d]pyrimidin-2-yl)butane-1,4-diamine.
To a mixture of N'-indan-2-yl-N'-methyl-butane-1,4-diamine (17.5 mg, 1.1 eq.), 2-chloro-7-methyl-pyrrolo[2,3-d]pyrimidine (12.5 mg, 0.07 mmol, 1 eq.), CuI (5.7 mg, 0.4 eq.), 1,10-phenanthroline(5.4 mg, 0.4 eq.), K_2CO_3 (20 mg, 2 eq.) in DMF (1 mL) was stirred at 120 °C for 48h. LCMS showed major product. After cooling down and filtered, the crude was purified by hplc using 0-60% ACN in H_2O (+ 0.1 % TFA) to afford desired product as yellow TFA salt solid (9 mg, 28% yield). LCMS (ESI+): calculated for $C_{21}H_{28}N_5$ $(M+H)^+$: 350.2; found 350.3.
^1H NMR (400 MHz, $CDCl_3/CD_3OD$) δ 8.24 (s, 1H), 7.21-7.14 (m, 4H), 7.03 (d, J = 3.8 Hz, 1H), 6.46 (d, J = 3.8 Hz, 1H), 4.16-4.05 (m, 1H), 3.69 (s, 3H), 3.58-3.45 (m, 2H), 3.31-3.03 (m, 6H), 2.70 (s, 3H), 1.95-1.65 (m, 4H).
^{13}C NMR (100 MHz, $CDCl_3/CD_3OD$) δ 154.6, 151.2, 138.5, 138.1, 132.3, 127.6, 124.4, 110.9, 102.0, 64.9, 53.8, 40.2, 36.4, 31.0, 25.4, 21.1.

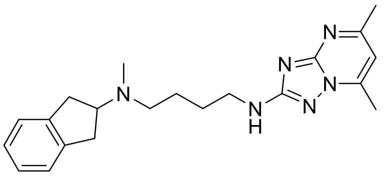

11. N1-(2,3-dihydro-1H-inden-2-yl)-N4-(5,7-dimethyl-[1,2,4]triazolo[1,5-a]pyrimidin-2-yl)-N1-methylbutane-1,4-diamine.
To a mixture of N'-indan-2-yl-N'-methyl-butane-1,4-diamine (17.5 mg, 2.2 eq.), 2-bromo-5,7-dimethyl-[1,2,4]triazolo[1,5-a]pyrimidine (8.5 mg, 0.04 mmol, 1 eq.), CuI (2.8 mg, 0.4 eq.), 1,10-phenanthroline (2.7 mg, 0.4 eq.), K_2CO_3 (10 mg, 2 eq.) in DMF (1 mL) was stirred at 120 °C for 48h. LCMS showed trace of product. After cooling down and filtered, the crude was purified by hplc using 0-60% ACN in H_2O (+ 0.1 % TFA) for 30 min to afford desired

product as yellow TFA salt solid (1 mg). LCMS (ESI+): calculated for $C_{21}H_{29}N_6$ $(M+H)^+$: 365.2; found 365.3.
^1H NMR (400 MHz, $CDCl_3/CD_3OD$) δ 7.22-7.15 (m, 4H), 6.77 (s, 1H), 4.14-4.04 (m, 1H), 3.45 (t, *J* = 6.2 Hz, 2H), 3.31-3.01 (m, 6H), 2.70 (s, 3H), 2.68 (s, 3H), 2.57 (s, 3H), 2.10-1.71 (m, 4H).

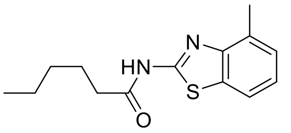

12. N-(4-methylbenzo[d]thiazol-2-yl)hexanamide.
To a mixture of hexanoic acid (37.5 µL, 1.5 eq.), HATU (106 mg, 1.4 eq.), DIEA (70 µL, 2 eq.) in DCM (3 mL) was stirred for 5 min at rt. Then, 4-methyl-1,3-benzothiazol-2-amine (32.8 mg, 0.2 mmol, 1 eq.) was added and the reaction was stirred at rt for overnight. After removing of DCM, the crude was purified by hplc using 0-100% ACN in H_2O (+ 0.1 % TFA) for 30 min to afford desired product as white solid (40 mg, 76% yield). LCMS (ESI+): calculated for $C_{14}H_{19}N_2OS$ $(M+H)^+$: 263.1; found 263.2.
^1H NMR (400 MHz, $CDCl_3$) δ 9.64 (br s, 1H), 7.66 (dd, *J* = 7.4, 1.3 Hz, 1H), 7.26-7.18 (m, 2H), 2.64 (s, 3H), 2.42 (t, *J* = 7.6 Hz, 2H), 1.75-1.65 (m, 2H), 1.35-1.25 (m, 4H), 0.89-0.85 (m, 3H).
^{13}C NMR (100 MHz, $CDCl_3$) δ 171.3, 157.2, 147.0, 131.8, 130.4, 127.0, 123.9, 118.9, 36.5, 31.2, 24.7, 22.2, 18.1, 13.8.

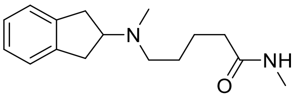

13. 5-((2,3-dihydro-1H-inden-2-yl)(methyl)amino)-N-methylpentanamide.
To a mixture of N-methyl-2-indane-amine (130 mg, 0.883 mmol, 1 eq.), 5-bromo-N-methyl-pentanamide (150 mg, 1 eq.), K_2CO_3 (123 mg, 1 eq.), KI (147 mg, 1 eq.) in ACN (2 mL) was stirred at rt for overnight. LCMS showed desired product. The solid was filtered off. Purification using hplc 0-40% ACN in H2O (+ 0.1% TFA) for 30 min to afford desired product as TFA salt (10 mg, 3% yield). LCMS (ESI+): calculated for $C_{16}H_{25}N_2O$ $(M+H)^+$: 261.2; found 261.3.
^1H NMR (400 MHz, $CDCl_3$) δ 7.26-7.21 (m, 4H), 6.72 (br s, 1H), 4.18-4.06 (m, 1H), 3.46-3.18 (m, 5H), 3.02-2.91 (m, 1H), 2.80 (d, *J* = 4.1 Hz, 3H), 2.72 (d, *J* = 2.6 Hz, 3H), 2.32 (t, *J* = 6.7 Hz, 2H), 1.87-1.69 (m, 4H).
^{13}C NMR (100 MHz, $CDCl_3$) δ 174.2, 138.3, 138.1, 127.9, 127.8, 124.5, 124.4, 65.4, 54.0, 36.9, 34.9, 34.7, 33.7, 26.5, 23.5, 22.3.

Synthetic procedures for analogs of compound **2**: Compounds **14-33** and **41**

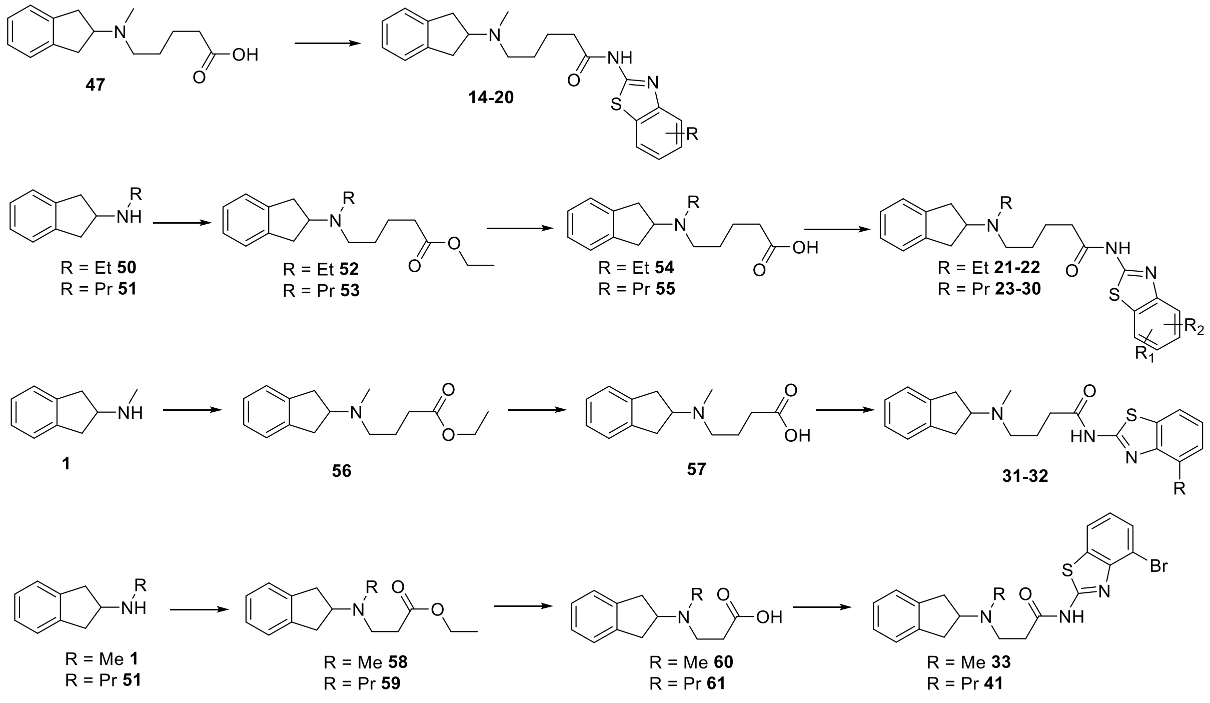

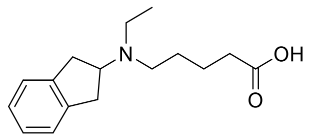

54. 5-((2,3-dihydro-1H-inden-2-yl)(ethyl)amino)pentanoic acid.

Step 1

To a mixture of N-Et-2-indane-amine (322 mg, 2 mmol, 1 eq.), ethyl 5-bromopentanoate (0.48 mL, 1.5 eq.), K_2CO_3 (552 mg, 2 eq.), in ACN (5 mL) was stirred at 60 °C for overnight. LCMS showed major desired product. The mixture was filtered. After removing of solvent, the crude was purified by silica gel column using PE:EA 7:3 + 0.5% Et_3N to afford desired product ethyl 5-((2,3-dihydro-1H-inden-2-yl)(ethyl)amino)pentanoate as a yellow oil (364 mg, 63% yield). LCMS (ESI+): calculated for $C_{18}H_{28}NO_2$ $(M+H)^+$: 290.2; found 290.3.

Step 2

To a solution of ethyl 5-[indan-2-yl(ethyl)amino]pentanoate (364 mg, 1.25 mmol, 1 eq.) in (5 mL) was added KOH (140 mg, 2 eq.). The mixture was heated at 60 °C for 2 h. LCMS showed complete ester hydrolysis. MeOH was removed and the mixture was diluted with ethyl acetate (20 mL), neutralized with concentrated HCl (1 mL) in an ice bath to pH = 1, dried over $MgSO_4$, filtered and concentrated to give a crude. Purification using hplc 0-40% ACN in H_2O (+ 0.1% TFA) for 30 min to afford desired product as TFA salt (400 mg, 85% yield). LCMS (ESI+): calculated for $C_{16}H_{24}NO_2$ $(M+H)^+$: 262.2; found 262.3.
^{1}H NMR (400 MHz, $CDCl_3$) δ 7.24-7.15 (m, 4H), 4.34-4.26 (m, 1H), 3.47-3.15 (m, 8H), 2.41 (t, J = 7.0 Hz, 2H), 1.93-1.61 (m, 4H), 1.37 (t, J = 7.3 Hz, 3H).

^{13}C NMR (100 MHz, $CDCl_3$) δ 176.7, 139.9, 128.7, 125.6, 64.5, 51.5, 47.5, 35.9, 33.9, 24.5, 22.9, 9.2.

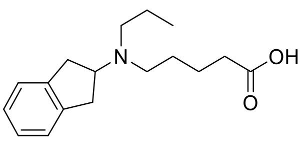

55. 5-((2,3-dihydro-1H-inden-2-yl)(propyl)amino)pentanoic acid.

Step 1
To a mixture of N-Pr-2-indane-amine (600 mg, 3.4 mmol, 1 eq.), ethyl 5-bromopentanoate (0.82 mL, 1.5 eq.), K_2CO_3 (946 mg, 2 eq.), NaI (509 mg, 1 eq.) in DMF (2 mL) was stirred at 50 °C for overnight. LCMS showed major desired product. The mixture was diluted by DCM (30 mL), washed with H_2O (15 mL x 2), and brine (10 mL). Purification with silica gel column chromatography using PE:EA 7:3 + 0.5% Et_3N afforded desired product ethyl 5-[indan-2-yl(propyl)amino]pentanoate as a yellow oil (550 mg, 53% yield). LCMS (ESI+): calculated for $C_{18}H_{28}NO_2$ $(M+H)^+$: 304.2; found 304.3.
^1H NMR (400 MHz, $CDCl_3$) δ 7.20-7.10 (m, 4H), 4.13 (q, J = 7.1 Hz, 2H), 3.70-3.59 (m, 1H), 3.01 (dd, *J* = 15.2, 7.7 Hz, 2H), 2.88 (dd, *J* = 15.2, 8.8 Hz, 2H), 2.57-2.47 (m, 4H), 2.32 (d, *J* = 7.2 Hz, 2H), 2.27 (s, 3H), 1.68-1.42 (m, 6H), 1.25 (t, *J* = 7.1 Hz, 3H), 0.88 (t, *J* = 7.3 Hz, 3H).
^{13}C NMR (100 MHz, $CDCl_3$) δ 173.6, 141.9, 126.2, 124.4, 63.1, 60.2, 53.4, 50.9, 36.6, 34.3, 26.6, 23.0, 20.2, 14.2, 12.0.

Step 2
To a solution of ethyl 5-[indan-2-yl(propyl)amino]pentanoate (540 mg, 1.78 mmol, 1 eq.) in MeOH (5 mL) was added KOH (199 mg, 2 eq.). The mixture was heated at 60 °C for 2 h. LCMS showed complete ester hydrolysis. MeOH was removed and the mixture was diluted with ethyl acetate (20 mL), neutralized with concentrated HCl (1 mL) in an ice bath to pH = 1, dried over $MgSO_4$, filtered and concentrated to give a crude. Purification using hplc 0-40% ACN in H_2O (+ 0.1% TFA) for 30 min to afford desired product as TFA salt (500 mg, 72% yield). LCMS (ESI+): calculated for $C_{17}H_{26}NO_2$ $(M+H)^+$: 276.2; found 276.3.
^1H NMR (400 MHz, CD_3OD) δ 7.29-7.20 (m, 4H), 4.35-4.24 (m, 1H), 3.51-3.12 (m, 8H), 2.41 (t, *J* = 7.0 Hz, 2H), 1.90-1.63 (m, 6H), 1.04 (t, *J* = 7.3 Hz, 3H).
^{13}C NMR (100 MHz, CD_3OD) δ 176.7, 139.9, 128.7, 125.6, 65.0, 54.1, 52.3, 35.9, 33.9, 24.4, 22.9, 18.6, 11.2.

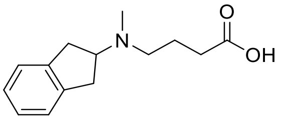

57. 4-((2,3-dihydro-1H-inden-2-yl)(methyl)amino)butanoic acid.

Step 1
To a mixture of N-Me-2-indane-amine (500 mg, 3.4 mmol, 1 eq.), ethyl 4-bromobutanoate (0.73 mL, 1.5 eq.), K_2CO_3 (946 mg, 2 eq.) in ACN (10 mL) was stirred at 60 °C for overnight. LCMS showed major desired product. After filtering, removing of solvent, the crude was purified by silica gel column using PE:EA 7:3 + 0.5% Et_3N to afford desired product ethyl 4-[indan-2-yl(methyl)amino]butanoate as yellow oil (500 mg, 56% yield). LCMS (ESI+): calculated for $C_{16}H_{24}NO_2$ $(M+H)^+$: 262.2; found 262.3.

^{1}H NMR (400 MHz, $CDCl_3$) δ 7.19-7.10 (m, 4H), 4.13 (q, *J* = 7.1 Hz, 2H), 3.40-3.32 (m, 1H), 3.05 (dd, *J* = 15.1, 7.5 Hz, 2H), 2.88 (dd, *J* = 15.3, 8.7 Hz, 2H), 2.50 (t, *J* = 7.1 Hz, 2H), 2.36 (t, *J* = 7.3 Hz, 2H), 2.28 (s, 3H), 1.88-1.80 (m, 2H), 1.23 (t, *J* = 7.1 Hz, 3H).
^{13}C NMR (100 MHz, $CDCl_3$) δ 173.6, 141.7, 126.3, 124.4, 66.2, 60.3, 54.4, 39.4, 36.9, 32.2, 22.2, 14.2.

Step 2
To a solution of ethyl 4-[indan-2-yl(methyl)amino]butanoate (500 mg, 1.91 mmol, 1 eq.) in MeOH (5 mL) was added KOH (214 mg, 2 eq.). The mixture was heated at 60 °C for 2 h. LCMS showed complete ester hydrolysis. MeOH was removed and the mixture was diluted with ethyl acetate (50 mL), neutralized with concentrated HCl (1 mL) in an ice bath to pH = 1, dried over $MgSO_4$, filtered and concentrated to give a crude. Dissolve the crude with small amount of ACN and 10 times H_2O and freezed dry overnight to eliminate all the trace of alcohol that can disturb its use in amide coupling. Yield: 0.4 g, 90%. This material is used as such for the next step without purification. LCMS (ESI+): calculated for $C_{14}H_{20}NO_2$ $(M+H)^+$: 234.2; found 234.2.
^{1}H NMR (400 MHz, CD_3OD) δ 7.31-7.18 (m, 4H), 4.30-4.18 (m, 1H), 3.46 (dd, *J* = 16.2, 8.1 Hz, 2H), 3.24 (dd, *J* = 16.0, 7.5 Hz, 2H), 2.89 (s, 3H), 2.49 (t, *J* = 6.8 Hz, 2H), 2.11-2.01 (m, 2H).
^{13}C NMR (100 MHz, CD_3OD) δ 176.2, 140.0, 128.7, 125.6, 67.0, 55.3, 38.5, 35.9, 31.6, 20.6.

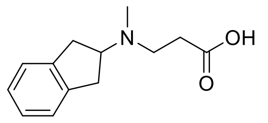

60. 3-((2,3-dihydro-1H-inden-2-yl)(methyl)amino)propanoic acid.

Step 1
To a mixture of N-Me-2-indane-amine (500 mg, 3.4 mmol, 1 eq.), ethyl but-3-enoate (0.44 mL, 1.05 eq.), in EtOH (10 mL) was stirred at reflux for overnight. LCMS showed major desired product. After removing of solvent, the crude was purified by silica gel column using PE:EA 1:1 + 0.5% Et_3N to afford desired product ethyl 3-[indan-2-yl(methyl)amino]propanoate as yellow oil. Yield: 550 mg, 65%. LCMS (ESI+): calculated for $C_{15}H_{22}NO_2$ $(M+H)^+$: 248.2; found 248.2.
^{1}H NMR (400 MHz, $CDCl_3$) δ 7.22-7.07 (m, 4H), 4.15 (q, *J* = 7.1 Hz, 2H), 3.43-3.32 (m, 1H), 3.08 (dd, *J* = 15.4, 7.5 Hz, 2H), 2.93 (dd, *J* = 15.1, 8.5 Hz, 2H), 2.85 (t, *J* = 7.1 Hz, 2H), 2.54 (t, *J* = 7.1 Hz, 2H), 2.31 (s, 3H), 1.27 (t, *J* = 7.1 Hz, 3H).
^{13}C NMR (100 MHz, $CDCl_3$) δ 172.5, 141.5, 126.4, 124.4, 66.0, 60.5, 50.6, 39.4, 36.9, 32.2, 14.2.

Step 2
To a solution of ethyl 3-[indan-2-yl(methyl)amino]propanoate (550 mg, 2.23 mmol, 1 eq.) in MeOH (5 mL) was added KOH (250 mg, 2 eq.). The mixture was heated at 60 °C for 2 h. LCMS showed complete ester hydrolysis. MeOH was removed and the mixture was diluted with ethyl acetate (50 mL), neutralized with concentrated HCl (0.5 mL) in an ice bath to pH = 1, dried over $MgSO_4$, filtered and concentrated to give a crude. Dissolve the crude with small amount of ACN and 10 times H_2O and freezed dry overnight to eliminate all the trace of alcohol that can disturb its use in amide coupling. Yield: 0.45 g, 92%. This material is used as such for the next step without purification. LCMS (ESI+): calculated for $C_{13}H_{18}NO_2$ $(M+H)^+$: 220.1; found 220.2.

^{1}H NMR (400 MHz, CD_3OD) δ 7.32-7.18 (m, 4H), 4.31-4.23 (m, 1H), 3.70-3.31 (m, 6H), 2.92 (t, *J* = 6.6 Hz, 2H), 2.89 (s, 3H).
^{13}C NMR (100 MHz, CD_3OD) δ 173.4, 140.1, 128.7, 125.6, 67.7, 51.6, 38.9, 35.8, 29.6.

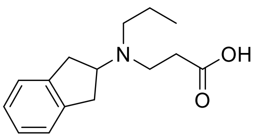

61. 3-((2,3-dihydro-1H-inden-2-yl)(propyl)amino)propanoic acid.

Step 1
To a mixture of N-Me-2-indane-amine (175 mg, 1 mmol, 1 eq.), ethyl but-3-enoate (0.32 mL, 3 eq.), in EtOH (3 mL) was stirred at reflux for 48 h. LCMS showed major desired product. After removing of solvent, the crude was purified by silica gel column using PE:EA 7:3 + 0.5% Et_3N to afford desired product ethyl 3-[indan-2-yl(propyl)amino]propanoate as yellow oil. Yield: 165 mg, 60%. LCMS (ESI+): calculated for $C_{17}H_{26}NO_2$ $(M+H)^+$: 276.2; found 276.3.

Step 2
To a solution of ethyl 3-[indan-2-yl(propyl)amino]propanoate (165 mg, 0.6 mmol, 1 eq.) in MeOH (5 mL) was added KOH (67 mg, 2 eq.). The mixture was heated at 60 °C for 2 h. LCMS showed complete ester hydrolysis. MeOH was removed and the mixture was diluted with ethyl acetate (20 mL), neutralized with concentrated HCl in an ice bath to pH = 1, dried over $MgSO_4$, filtered and concentrated to give a crude. Dissolve the crude with small amount of ACN and 10 times H_2O and freezed dry overnight to eliminate all the trace of alcohol that can disturb its use in amide coupling. Yield: 140 mg, 94%. This material is used as such for the next step without purification. LCMS (ESI+): calculated for $C_{15}H_{22}NO_2$ $(M+H)^+$: 248.2; found 248.2.

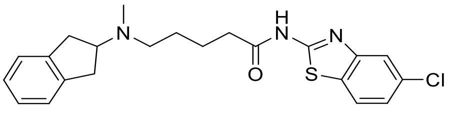

14. N-(5-chlorobenzo[d]thiazol-2-yl)-5-((2,3-dihydro-1H-inden-2-yl)(methyl)amino)pentanamide.
To a mixture of 5-[indan-2-yl(methyl)amino]pentanoic acid (50 mg, 0.2 mmol, 1 eq.), HATU (152 mg, 2 eq.), DIEA (70 µL, 2 eq.) in 3/1 mixture of DCM/DMF (v/v, 4 mL) was stirred for 5 min at rt. Then, 5-chloro-1,3-benzothiazol-2-amine (37 mg, 1 eq.) was added and the reaction was stirred at rt for 48 h. LCMS showed > 50% conversion. After removal of DCM and filtering, the crude was purified by hplc using 0-70% of ACN in H_2O (+ 0.1 % TFA) for 30 min to give desired product as yellow TFA salt solid (15 mg, 14% yield). LCMS (ESI+): calculated for $C_{22}H_{25}ClN_3O_2S$ $(M+H)^+$: 414.1; found 414.2.
^{1}H NMR (400 MHz, $CDCl_3/CD_3OD$) δ 7.70 (d, *J* = 1.5 Hz, 1H), 7.66 (d, *J* = 8.5 Hz, 1H), 7.22 (dd, *J* = 8.5, 1.5 Hz, 1H), 7.21-7.12 (m, 4H), 4.11-4.01 (m, 1H), 3.41-2.88 (m, 6H), 2.69 (s, 3H), 2.56 (t, *J* = 6.3 Hz, 2H), 1.91-1.71 (m, 4H).
^{13}C NMR (100 MHz, $CDCl_3/CD_3OD$) δ 171.6, 159.6, 149.4, 138.3, 132.0, 130.1, 127.7, 124.4, 124.1, 121.9, 120.6, 65.0, 53.8, 36.8, 34.6, 23.3, 21.6.

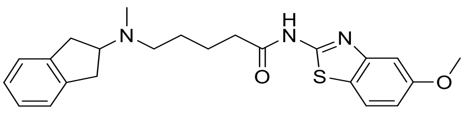

15. 5-((2,3-dihydro-1H-inden-2-yl)(methyl)amino)-N-(5-methoxybenzo[d]thiazol-2-yl)pentanamide.
To a mixture of 5-[indan-2-yl(methyl)amino]pentanoic acid (50 mg, 0.2 mmol, 1 eq.), HATU (152 mg, 2 eq.), DIEA (70 µL, 2 eq.) in 3/1 mixture of DCM/DMF (v/v, 4 mL) was stirred for 5 min at rt. Then, 5-methoxy-1,3-benzothiazol-2-amine (36 mg, 1 eq.) was added and the reaction was stirred at rt for 48 h. LCMS showed > 50% conversion. After removal of DCM and filtering, the crude was purified by hplc using 0-70% of ACN in H_2O (+ 0.1 % TFA) for 30 min to give desired product as yellow TFA salt solid (35 mg, 33% yield). LCMS (ESI+): calculated for $C_{23}H_{28}N_3O_2S$ $(M+H)^+$: 410.2; found 410.3.
^{1}H NMR (400 MHz, $CDCl_3/CD_3OD$) δ 7.62-7.55 (m, 1H), 7.23-7.09 (m, 5H), 6.95-6.88 (m, 1H), 4.10-3.98 (m, 1H), 3.80 (s, 3H), 3.32-2.86 (m, 6H), 2.67 (s, 3H), 2.55-2.53 (m, 2H), 1.84-1.67 (m, 4H).
^{13}C NMR (100 MHz, $CDCl_3/CD_3OD$) δ 171.5, 160.4, 159.4, 138.2, 127.6, 124.3, 121.8, 114.1, 102.6, 65.0, 55.5, 53.8, 36.7, 34.5, 23.1, 21.4.

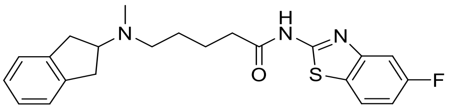

16. 5-((2,3-dihydro-1H-inden-2-yl)(methyl)amino)-N-(5-fluorobenzo[d]thiazol-2-yl)pentanamide.
To a mixture of 5-[indan-2-yl(methyl)amino]pentanoic acid (50 mg, 0.2 mmol, 1 eq.), HATU (152 mg, 2 eq.), DIEA (70 µL, 2 eq.) in 3/1 mixture of DCM/DMF (v/v, 4 mL) was stirred for 5 min at rt. Then, 5-fluoro-1,3-benzothiazol-2-amine (33.6 mg, 1 eq.) was added and the reaction was stirred at rt for 48 h. LCMS showed > 50% conversion. After removal of DCM and filtering, the crude was purified by hplc using 0-70% of ACN in H_2O (+ 0.1 % TFA) for 30 min to give desired product as yellow TFA salt solid (22 mg, 21% yield). LCMS (ESI+): calculated for $C_{12}H_{23}N_2O$ $(M+H)^+$: 414.1; found 414.2.
^{1}H NMR (400 MHz, $CDCl_3$) δ 12.72 (br s, 2H), 7.76 (dd, J = 8.8, 4.9 Hz, 1H), 7.19 (dd, J = 8.8, 6.5 Hz, 1H), 7.24-7.18 (m, 4H), 7.17 (td, J = 8.8, 2.4 Hz, 1H), 4.16-4.06 (m, 1H), 3.43-2.92 (m, 6H), 2.73 (s, 3H), 2.70 (t, J = 6.8 Hz, 2H), 2.02-1.82 (m, 4H).
^{13}C NMR (100 MHz, $CDCl_3$) δ 171.5, 163.6 (d, J = 1.6 Hz), 162.6 (d, J = 247.6 Hz), 158.9, 138.3, 127.8, 124.4, 123.2 (d, J = 9.9 Hz), 114.3 (d, J = 24.6 Hz), 105.0 (d, J = 26.2 Hz), 65.2, 53.9, 36.9, 35.1, 34.9, 33.7, 23.2, 21.6.

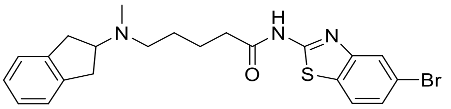

17. N-(5-bromobenzo[d]thiazol-2-yl)-5-((2,3-dihydro-1H-inden-2-yl)(methyl)amino)pentanamide.
To a mixture of 5-[indan-2-yl(methyl)amino]pentanoic acid (50 mg, 0.2 mmol, 1 eq.), HATU (152 mg, 2 eq.), DIEA (70 µL, 2 eq.) in 3/1 mixture of DCM/DMF (v/v, 4 mL) was stirred for 5 min at rt. Then, 5-bromo-1,3-benzothiazol-2-amine hydrochloride (46 mg, 1 eq.) was added and the reaction was stirred at rt for 48 h. LCMS showed > 50% conversion. After removal of DCM and filtering, the crude was purified by silica gel column chromatography using petroleum ether/ethyl acetate (3:7) with 0.5% of Et_3N to give desired product as white solid (38 mg, 41% yield). LCMS (ESI+): calculated for $C_{22}H_{25}BrN_3OS$ $(M+H)^+$: 458.1; found 458.2.
^{1}H NMR (400 MHz, $CDCl_3$) δ 7.82 (d, J = 1.7 Hz, 1H), 7.64 (d, J = 8.4 Hz, 1H), 7.38 (dd, J = 8.4, 1.7 Hz, 1H), 7.23-7.11 (m, 4H), 3.57-3.47 (m, 1H), 3.17-3.06 (m, 4H), 2.66-2.53 (m, 4H), 2.37 (s, 3H), 1.92-1.63 (m, 4H).

^{13}C NMR (100 MHz, $CDCl_3$) δ 171.9, 159.6, 149.6, 141.2, 131.0, 126.7, 126.6, 124.4, 123.7, 122.4, 119.5, 66.3, 54.5, 39.1, 36.3, 35.6, 25.2, 23.4.

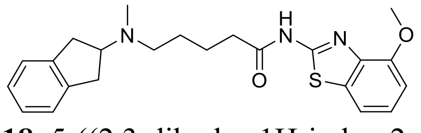

18. 5-((2,3-dihydro-1H-inden-2-yl)(methyl)amino)-N-(4-methoxybenzo[d]thiazol-2-yl)pentanamide.

To a mixture of 5-[indan-2-yl(methyl)amino]pentanoic acid (50 mg, 0.2 mmol, 1 eq.), HATU (152 mg, 2 eq.), DIEA (70 µL, 2 eq.) in 3/1 mixture of DCM/DMF (v/v, 4 mL) was stirred for 5 min at rt. Then, 4-methoxy-1,3-benzothiazol-2-amine (36 mg, 1 eq.) was added and the reaction was stirred at rt for 48 h. LCMS showed > 50% conversion. After removal of DCM and filtering, the crude was purified by hplc using 0-70% of ACN in H_2O (+ 0.1 % TFA) for 30 min to give desired product as yellow TFA salt solid (30 mg, 28% yield). LCMS (ESI+): calculated for $C_{23}H_{28}N_3O_2S$ $(M+H)^+$: 410.2; found 410.3.

^1H NMR (400 MHz, $CDCl_3$) δ 13.07 (br s, 2H), 7.39 (dd, J = 8.0, 0.9 Hz, 1H), 7.32 (t, J = 8.0, 1H), 7.23-7.17 (m, 4H), 6.94 (dd, J = 8.0, 0.9 Hz, 1H), 4.12-4.05 (m, 1H), 3.99 (s, 3H), 3.41-2.88 (m, 6H), 2.71 (s, 3H), 2.63 (t, J = 6.7 Hz, 2H), 1.96-1.74 (m, 4H).

^{13}C NMR (100 MHz, $CDCl_3$) δ 171.3, 159.1, 151.0, 138.6, 134.3, 131.4, 127.7, 125.8, 124.5, 113.5, 107.8, 64.9, 56.0, 53.8, 36.8, 34.9, 23.2, 21.7.

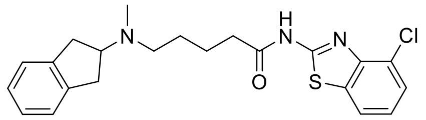

19. N-(4-chlorobenzo[d]thiazol-2-yl)-5-((2,3-dihydro-1H-inden-2-yl)(methyl)amino)pentanamide.

To a mixture of 5-[indan-2-yl(methyl)amino]pentanoic acid (50 mg, 0.2 mmol, 1 eq.), HATU (152 mg, 2 eq.), DIEA (70 µL, 2 eq.) in 3/1 mixture of DCM/DMF (v/v, 4 mL) was stirred for 5 min at rt. Then, 4-chloro-1,3-benzothiazol-2-amine (37 mg, 1 eq.) was added and the reaction was stirred at rt for 48 h. LCMS showed > 50 % conversion. After removal of DCM and filtering, the crude was purified by hplc using 0-70% of ACN in H_2O (+ 0.1 % TFA) for 30 min to give desired product as yellow TFA salt solid (30 mg, 28% yield). LCMS (ESI+): calculated for $C_{22}H_{25}ClN_3O_2S$ $(M+H)^+$: 414.1; found 414.2.

^1H NMR (400 MHz, CD_3OD) δ 7.81-7.78 (m, 1H), 7.47-7.43 (m, 1H), 7.31-7.18 (m, 5H), 4.29-4.19 (m, 1H), 3.54-3.11 (m, 6H), 2.88 (s, 3H), 2.71-2.61 (m, 2H), 2.00-1.75 (m, 4H).

^{13}C NMR (100 MHz, CD_3OD) δ 173.6, 160.1, 147.1, 139.9, 135.0, 128.8, 127.5, 126.9, 125.6, 121.2, 67.1, 55.6, 38.4, 36.2, 35.6, 35.4, 24.7, 22.8.

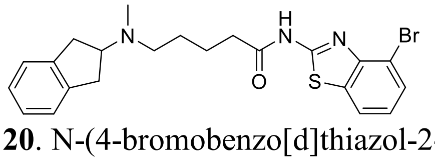

20. N-(4-bromobenzo[d]thiazol-2-yl)-5-((2,3-dihydro-1H-inden-2-yl)(methyl)amino)pentanamide.

To a mixture of 5-[indan-2-yl(methyl)amino]pentanoic acid (50 mg, 0.2 mmol, 1 eq.), HATU (152 mg, 2 eq.), DIEA (70 µL, 2 eq.) in 3/1 mixture of DCM/DMF (v/v, 4 mL) was stirred for 5 min at rt. Then, 4-bromo-1,3-benzothiazol-2-amine hydrochloride (53 mg, 1 eq.) was added and the reaction was stirred at rt for 48 h. LCMS showed > 50% conversion. After removal of DCM and filtering, the crude was purified by silica gel column chromatography using petroleum ether/ethyl acetate (3:7) with 0.5% of Et_3N to give desired product as yellow

TFA salt solid (42.6 mg, 46% yield). LCMS (ESI+): calculated for $C_{22}H_{25}BrN_3O_2S$ $(M+H)^+$: 458.1; found 458.2.

^{1}H NMR (400 MHz, CD_3OD) δ 7.82 (dd, *J* = 7.9, 0.9 Hz, 1H), 7.61 (dd, *J* = 7.9, 0.9 Hz, 1H), 7.23-7.04 (m, 5H), 4.12-4.05 (m, 1H), 3.17-3.24 (m, 4H), 2.69-2.54 (m, 4H), 2.36 (s, 3H), 1.83-1.61 (m, 4H).

^{13}C NMR (100 MHz, CD_3OD) δ 174.2, 159.6, 148.5, 142.2, 134.5, 130.6, 127.7, 125.8, 125.4, 121.8, 115.5, 67.4, 56.1, 39.7, 37.6, 36.4, 26.6, 24.0.

^{13}C NMR (125 MHz, CD_3OD) δ 173.4, 162.1, 158.4, 153.4, 139.9, 139.6, 134.6, 128.8, 126.1, 125.6, 114.4, 108.5, 64.5, 56.6, 51.5, 47.5, 35.9, 35.6, 24.5, 22.9, 9.3.

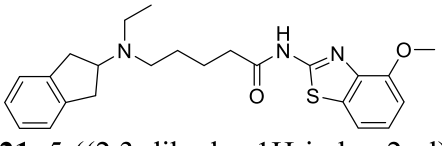

21. 5-((2,3-dihydro-1H-inden-2-yl)(ethyl)amino)-N-(4-methoxybenzo[d]thiazol-2-yl)pentanamide.

To a mixture of 5-[indan-2-yl(ethyl)amino]pentanoic acid TFA salt (37.6 mg, 0.1 mmol, 1 eq.), HATU (76 mg, 2 eq.), DIEA (51 μL, 3 eq.) in a 2/1 mixture of DCM/DMF (3 mL) was stirred for 5 min at rt. Then, 4-methoxy-1,3-benzothiazol-2-amine (18 mg, 1 eq.) was added and the reaction was stirred at rt for 48 h. After removing of DCM, the crude was purified by hplc using 0-70% ACN in H_2O (+ 0.1% TFA) for 30 min to afford desired product as white TFA salt solid (35 mg, 65% yield). LCMS (ESI+): calculated for $C_{24}H_{30}N_3O_2S$ $(M+H)^+$: 424.2; found 424.3.

^{1}H NMR (500 MHz, CD_3OD) δ 7.43 (d, *J* = 8.0 Hz, 1H), 7.27 (t, *J* = 8.0 Hz, 1H), 7.27-7.20 (m, 4H), 7.00 (d, *J* = 8.0 Hz, 1H), 4.35-4.27 (m, 1H), 4.00 (s, 3H), 3.50-3.13 (m, 8H), 2.66 (t, *J* = 6.2 Hz, 2H), 1.97-1.77 (m, 4H), 1.38 (t, *J* = 7.1 Hz, 3H).

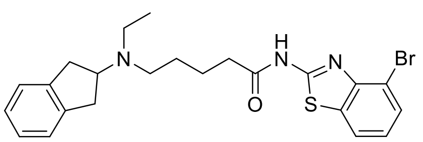

22. N-(4-bromobenzo[d]thiazol-2-yl)-5-((2,3-dihydro-1H-inden-2-yl)(ethyl)amino)pentanamide.

To a mixture of 4-[indan-2-yl(propyl)amino]pentanoic acid TFA salt (37.6 mg, 0.1 mmol, 1 eq.), HATU (76 mg, 2 eq.), DIEA (51 μL, 3 eq.) in 3/1 mixture of DCM/DMF (v/v, 4 mL) was stirred for 5 min at rt. Then, 4-bromo-1,3-benzothiazol-2-amine hydrochloride (26.5 mg, 1 eq.) was added and the reaction was stirred at rt for 48 h. After removal of DCM and filtering, the crude was purified by hplc using 0-70% of ACN in H_2O (+ 0.1% TFA) for 30 min to give desired product as white TFA salt solid (25 mg, 43% yield). LCMS (ESI+): calculated for $C_{23}H_{27}BrN_3OS$ $(M+H)^+$: 472.1; found 472.2.

^{1}H NMR (500 MHz, CD_3OD) δ 7.84 (d, *J* = 7.9 Hz, 1H), 7.63 (d, *J* = 7.9 Hz, 1H), 7.30-7.19 (m, 4H), 7.19 (t, *J* = 7.9 Hz, 1H), 4.34-4.27 (m, 1H), 3.50-3.18 (m, 8H), 2.65 (t, *J* = 6.2 Hz, 2H), 1.97-1.77 (m, 4H), 1.38 (t, *J* = 7.1 Hz, 3H).

^{13}C NMR (125 MHz, CD_3OD) δ 173.6, 159.7, 148.5, 139.8, 134.4, 130.7, 128.7, 125.9, 125.6, 121.8, 115.5, 65.0, 54.2, 52.2, 35.9, 35.6, 24.4, 22.8, 18.6, 11.2.

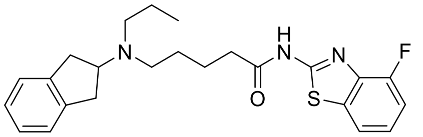

23. 5-((2,3-dihydro-1H-inden-2-yl)(propyl)amino)-N-(4-fluorobenzo[d]thiazol-2-yl)pentanamide.
To a mixture of 5-[indan-2-yl(propyl)amino]pentanoic acid (25 mg, 0.09 mmol, 1 eq.), HATU (44 mg, 2 eq.), DIEA (31.6 µL, 2 eq.) in a 2/1 mixture of DCM/DMF (3 mL) was stirred for 5 min at rt. Then, 4-fluoro-1,3-benzothiazol-2-amine (15.3 mg, 1 eq.) was added and the reaction was stirred at rt for 48 h. After removing of DCM, the crude was purified by hplc using 0-75% ACN in H_2O (+ 0.1 % TFA) to afford desired product as white TFA salt solid (7 mg, 14% yield). LCMS (ESI+): calculated for $C_{24}H_{29}FN_3OS$ $(M+H)^+$: 426.2; found 426.3.
^{1}H NMR (400 MHz, CD_3OD) δ 7.66 (dd, J = 8.0, 0.9 Hz, 1H), 7.30 (dd, J = 8.0, 0.9 Hz, 1H), 7.30-7.19 (m, 4H), 7.17 (t, J = 10.8, 8.0, 0.9 Hz, 1H), 4.37-4.26 (m, 1H), 3.51-3.12 (m, 8H), 2.66 (t, J = 6.5 Hz, 2H), 1.95-1.69 (m, 6H), 1.04 (t, J = 7.1 Hz, 3H).
^{13}C NMR (100 MHz, CD_3OD) δ 173.6, 159.9, 156.1 (d, J = 252.4 Hz), 139.9, 138.5, 136.4, 128.8, 125.7 (d, J = 6.9 Hz), 125.6, 118.2 (d, J = 4.2 Hz), 112.9 (d, J = 18.2 Hz), 65.0, 54.2, 52.2, 35.9, 35.5, 24.4, 22.8, 18.6, 11.2.

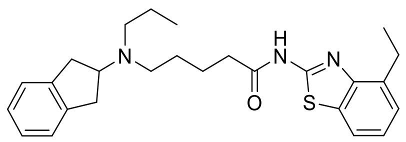

24. 5-((2,3-dihydro-1H-inden-2-yl)(propyl)amino)-N-(4-ethylbenzo[d]thiazol-2-yl)pentanamide.
To a mixture of 5-[indan-2-yl(propyl)amino]pentanoic acid (25 mg, 0.09 mmol, 1 eq.), HATU (44 mg, 2 eq.), DIEA (31.6 µL, 2 eq.) in a 2/1 mixture of DCM/DMF (3 mL) was stirred for 5 min at rt. Then, 4-ethyl-1,3-benzothiazol-2-amine (16.2 mg, 1 eq.) was added and the reaction was stirred at rt for 48 h. After removing of DCM, the crude was purified by hplc using 0-75% ACN in H_2O (+ 0.1% TFA) for 30 min to afford desired product as white TFA salt solid (10 mg, 20% yield). LCMS (ESI+): calculated for $C_{26}H_{34}N_3OS$ $(M+H)^+$: 436.2; found 436.3.
^{1}H NMR (400 MHz, CD_3OD) δ 7.69-7.63 (m, 1H), 7.31-7.16 (m, 6H), 4.37-4.25 (m, 1H), 3.51-3.11 (m, 8H), 3.04 (q, J = 7.5 Hz, 2H), 2.63 (t, J = 6.2 Hz, 2H), 1.96-1.74 (m, 6H), 1.31 (t, J = 7.5 Hz, 3H), 1.04 (t, J = 7.1 Hz, 3H).
^{13}C NMR (100 MHz, CD_3OD) δ 173.4, 158.1, 148.6, 139.9, 138.3, 133.4, 128.7, 126.2, 125.6, 125.1, 119.7, 65.0, 54.2, 52.2, 35.9, 35.6, 26.4, 24.2, 22.9, 18.6, 15.5, 11.2.

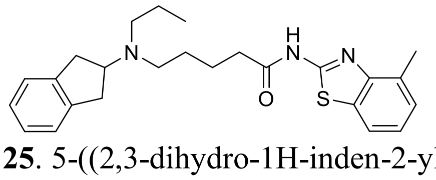

25. 5-((2,3-dihydro-1H-inden-2-yl)(propyl)amino)-N-(4-methylbenzo[d]thiazol-2-yl)pentanamide.
To a mixture of 5-[indan-2-yl(propyl)amino]pentanoic acid (10 mg, 0.036 mmol, 1 eq.), HATU (13 mg, 1.5 eq.), DIEA (12.6 uL, 2 eq.) in DCM (2 mL) was stirred for 5 min at rt. Then, 4-methyl-1,3-benzothiazol-2-amine (6 mg, 1 eq.) was added and the reaction was stirred at rt for ovn. After removing of DCM, the crude was purified by hplc using 0-70% ACN in H_2O (+ 0.1 % TFA) to afford desired product as white TFA salt solid (4 mg, 21% yield). LCMS (ESI+): calculated for $C_{25}H_{32}N_3OS$ $(M+H)^+$: 422.2; found 422.3.
^{1}H NMR (400 MHz, $CDCl_3$) δ 12.86 (br s, 1H), 7.69-7.64 (m, 1H), 7.40-7.34 (m, 2H), 7.24-7.14 (m, 4H), 4.14-4.02 (m, 1H), 3.43 (dd, J = 15.8, 8.0 Hz, 2H), 3.29 (dd, J = 15.8, 8.0 Hz, 2H), 3.18-2.95 (m, 4H), 2.74 (t, J = 6.6 Hz, 3H), 2.68 (s, 3H), 2.01-1.73 (m, 6H), 1.00 (t, J = 7.3 Hz, 3H).

^{13}C NMR (100 MHz, $CDCl_3$) δ 172.0, 138.5, 129.2, 128.3, 127.6, 125.9, 124.5, 119.5, 63.4, 52.2, 50.2, 35.2, 34.6, 22.8, 21.9, 17.8, 16.8, 11.2.

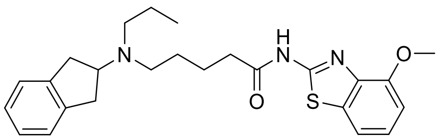

26. 5-((2,3-dihydro-1H-inden-2-yl)(propyl)amino)-N-(4-methoxybenzo[d]thiazol-2-yl)pentanamide.

To a mixture of 5-[indan-2-yl(propyl)amino]pentanoic acid (20 mg, 0.09 mmol, 1 eq.), HATU (44 mg, 2 eq.), DIEA (31.6 µL, 2 eq.) in a 2/1 mixture of DCM/DMF (3 mL) was stirred for 5 min at rt. Then, 4-methoxy-1,3-benzothiazol-2-amine (16.4 mg, 1 eq.) was added and the reaction was stirred at rt for 48 h. After removing of DCM, the crude was purified by hplc using 0-70% ACN in H_2O (+ 0.1 % TFA) to afford desired product as white TFA salt solid (19 mg, 38% yield). LCMS (ESI+): calculated for $C_{25}H_{32}N_3OS$ $(M+H)^+$: 438.2; found 438.3.

^1H NMR (400 MHz, CD_3OD) δ 7.43 (d, J = 7.9 Hz, 1H), 7.30-7.17 (m, 4H), 7.00 (d, J = 7.9 Hz, 1H), 4.36-4.25 (m, 1H), 4.00 (s, 3H), 3.50-3.11 (m, 8H), 2.67-2.61 (m, 2H), 1.96-1.68 (m, 6H), 1.04 (t, J = 7.1 Hz, 3H).

^{13}C NMR (100 MHz, CD_3OD) δ 173.4, 153.4, 139.9, 139.6, 128.7, 126.1, 125.6, 114.4, 108.5, 65.0, 56.6, 54.2, 52.2, 35.9, 35.6, 24.4, 22.9, 18.6, 11.2.

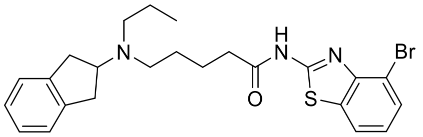

27. N-(4-bromobenzo[d]thiazol-2-yl)-5-((2,3-dihydro-1H-inden-2-yl)(propyl)amino)pentanamide.

To a mixture of 5-[indan-2-yl(propyl)amino]pentanoic acid (55 mg, 0.2 mmol, 1 eq.), HATU (152 mg, 2 eq.), DIEA (70 µL, 2 eq.) in 3/1 mixture of DCM/DMF (v/v, 4 mL) was stirred for 5 min at rt. Then, 4-bromo-1,3-benzothiazol-2-amine hydrochloride (53 mg, 1 eq.) was added and the reaction was stirred at rt for 48 h. LCMS showed > 50% conversion. After removal of DCM and filtering, the crude was purified by hplc using 0-75% of ACN in H_2O (+ 0.1% TFA) for 30min to give desired product as white TFA salt solid (20 mg, 17% yield). LCMS (ESI+): calculated for $C_{24}H_{29}BrN_3OS$ $(M+H)^+$: 486.1; found 486.2.

^1H NMR (400 MHz, CD_3OD) δ 7.83 (dd, J = 7.9, 1.0 Hz, 1H), 7.63 (dd, J = 7.9, 1.0 Hz, 1H), 7.30-7.19 (m, 4H), 7.19 (t, J = 7.9 Hz, 1H), 4.36-4.25 (m, 1H), 3.50-3.14 (m, 8H), 2.65 (t, J = 6.7 Hz, 2H), 1.95-1.70 (m, 6H), 1.04 (t, J = 7.1 Hz, 3H).

^{13}C NMR (100 MHz, CD_3OD) δ 173.6, 159.7, 148.5, 139.8, 134.4, 130.7, 128.7, 125.9, 125.6, 121.8, 115.5, 65.0, 54.2, 52.2, 35.9, 35.6, 24.4, 22.8, 18.6, 11.2.

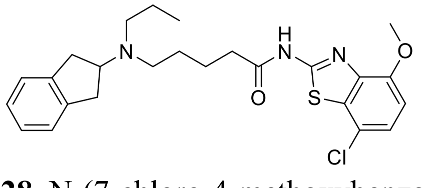

28. N-(7-chloro-4-methoxybenzo[d]thiazol-2-yl)-5-((2,3-dihydro-1H-inden-2-yl)(propyl)amino)pentanamide.

To a mixture of 5-[indan-2-yl(ethyl)amino]pentanoic acid TFA salt (39 mg, 0.1 mmol, 1 eq.), HATU (76 mg, 2 eq.), DIEA (51 µL, 3 eq.) in a 2/1 mixture of DCM/DMF (3 mL) was stirred for 5 min at rt. Then, 7-chloro-4-methoxy-1,3-benzothiazol-2-amine (21.5 mg, 1 eq.) was added and the reaction was stirred at rt for 48 h. After removing of DCM, the crude was

purified by hplc using 0-70% ACN in H_2O (+ 0.1% TFA) for 30 min to afford desired product as white TFA salt solid (35 mg, 60% yield). LCMS (ESI+): calculated for $C_{25}H_{31}ClN_3O_2S$ $(M+H)^+$: 472.2; found 472.2.
^1H NMR (500 MHz, CD_3OD) δ 7.30-7.19 (m, 4H), 7.25 (d, *J* = 8.6 Hz, 1H), 6.98 (d, *J* = 8.6 Hz, 1H), 4.35-4.27 (m, 1H), 3.99 (s, 3H), 3.45-3.15 (m, 8H), 2.66 (t, *J* = 6.5 Hz, 2H), 1.92-1.69 (m, 6H), 1.04 (t, *J* = 7.1 Hz, 3H).
^{13}C NMR (125 MHz, CD_3OD) δ 173.6, 158.6, 152.4, 140.2, 139.9, 133.8, 128.7, 125.6, 125.2, 118.7, 110.0, 65.0, 56.9, 54.2, 52.2, 35.9, 35.5, 24.4, 22.8, 18.6, 11.2.

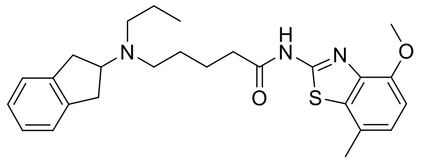

29. 5-((2,3-dihydro-1H-inden-2-yl)(propyl)amino)-N-(4-methoxy-7-methylbenzo[d]thiazol-2-yl)pentanamide.
To a mixture of 5-[indan-2-yl(ethyl)amino]pentanoic acid TFA salt (39 mg, 0.1 mmol, 1 eq.), HATU (76 mg, 2 eq.), DIEA (51 μL, 3 eq.) in a 2/1 mixture of DCM/DMF (3 mL) was stirred for 5 min at rt. Then, 4-methoxy-7-methyl-1,3-benzothiazol-2-amine (19.4 mg, 1 eq.) was added and the reaction was stirred at rt for 48 h. After removing of DCM, the crude was purified by hplc using 0-70% ACN in H_2O (+ 0.1 % TFA) to afford desired product as white TFA salt solid (35 mg, 62% yield). LCMS (ESI+): calculated for $C_{26}H_{34}N_3O_2S$ $(M+H)^+$: 452.2; found 452.3.
^1H NMR (500 MHz, CD_3OD) δ 7.26-7.19 (m, 4H), 7.07 (dd, *J* = 8.1, 0.7 Hz, 1H), 6.91 (d, *J* = 8.1 Hz, 1H), 4.34-4.27 (m, 1H), 3.97 (s, 3H), 3.48-3.15 (m, 11H), 2.44 (t, J = 6.5 Hz, 2H), 1.92-1.72 (m, 6H), 1.04 (t, *J* = 7.1 Hz, 3H).
^{13}C NMR (125 MHz, CD_3OD) δ 173.3, 158.0, 151.6, 139.9, 139.0, 134.3, 128.7, 126.0, 125.6, 124.3, 108.9, 65.0, 56.6, 54.2, 52.2, 35.9, 35.6, 24.4, 22.8, 19.9, 18.6, 11.2.

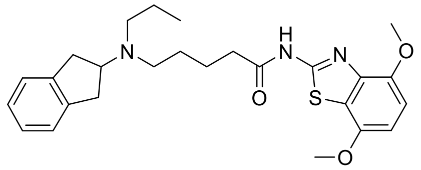

30. 5-((2,3-dihydro-1H-inden-2-yl)(propyl)amino)-N-(4,7-dimethoxybenzo[d]thiazol-2-yl)pentanamide.
To a mixture of 5-[indan-2-yl(ethyl)amino]pentanoic acid TFA salt (39 mg, 0.1 mmol, 1 eq.), HATU (76 mg, 2 eq.), DIEA (51 μL, 3 eq.) in a 2/1 mixture of DCM/DMF (3 mL) was stirred for 5 min at rt. Then, 4,7-dimethoxy-1,3-benzothiazol-2-amine (21 mg, 1 eq.) was added and the reaction was stirred at rt for 48 h. After removing of DCM, the crude was purified by hplc using 0-70% ACN in H_2O (+ 0.1% TFA) for 30 min to afford desired product as white TFA salt solid (35 mg, 60% yield). LCMS (ESI+): calculated for $C_{26}H_{34}N_3O_3S$ $(M+H)^+$: 468.2; found 468.3.
^1H NMR (500 MHz, CD_3OD) δ 7.26-7.19 (m, 4H), 6.92 (d, J = 8.7 Hz, 1H), 6.79 (d, *J* = 8.7 Hz, 1H), 4.35-4.27 (m, 1H), 3.95 (s, 3H), 3.93 (s, 3H), 3.45-3.15 (m, 8H), 2.65 (t, *J* = 6.5 Hz, 2H), 1.96-1.69 (m, 6H), 1.04 (t, *J* = 7.1 Hz, 3H).
^{13}C NMR (125 MHz, CD_3OD) δ 173.4, 159.1, 149.7, 148.0, 140.8, 139.9, 128.7, 125.6, 122.8, 109.2, 105.5, 65.0, 57.0, 56.5, 54.2, 52.2, 35.9, 35.5, 24.4, 22.8, 18.6, 11.2.

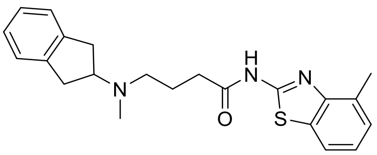

31. 4-((2,3-dihydro-1H-inden-2-yl)(methyl)amino)-N-(4-methylbenzo[d]thiazol-2-yl)butanamide.
To a mixture of 4-[indan-2-yl(methyl)amino]butanoic acid (46.7 mg, 0.2 mmol, 1 eq.), HATU (152 mg, 2 eq.), DIEA (70 µL, 2 eq.) in 3/1 mixture of DCM/DMF (v/v, 4 mL) was stirred for 5 min at rt. Then, 4-methyl-1,3-benzothiazol-2-amine (32.8 mg, 1 eq.) was added and the reaction was stirred at rt for 48 h. LCMS showed > 50% conversion. After removal of DCM and filtering, the crude was purified by hplc using 0-60% of ACN in H_2O (+ 0.1% TFA) for 30 min to give desired product as white TFA salt solid (38 mg, 38% yield). LCMS (ESI+): calculated for $C_{22}H_{26}N_3OS$ $(M+H)^+$: 380.2; found 380.2.
^{1}H NMR (400 MHz, $CDCl_3$) δ 7.68-7.62 (m, 1H), 7.37-7.30 (m, 2H), 7.25-7.17 (m, 4H), 4.19-4.10 (m, 1H), 3.43-3.14 (m, 4H), 2.81 (t, J = 6.8 Hz, 2H), 2.79 (s, 3H), 2.65 (s, 3H), 2.33-2.23 (m, 2H).
^{13}C NMR (100 MHz, $CDCl_3$) δ 170.9, 160.8, 139.9, 138.5, 128.9, 128.7, 128.1, 127.7, 125.6, 124.5, 119.3, 65.0, 53.3, 37.0, 32.8, 19.0, 17.8.

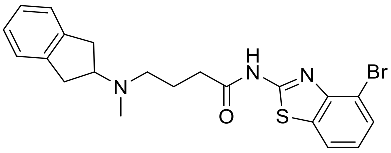

32. N-(4-bromobenzo[d]thiazol-2-yl)-4-((2,3-dihydro-1H-inden-2-yl)(methyl)amino)butanamide.
To a mixture of 4-[indan-2-yl(methyl)amino]butanoic acid (46.7 mg, 0.2 mmol, 1 eq.), HATU (152 mg, 2 eq.), DIEA (70 µL, 2 eq.) in 3/1 mixture of DCM/DMF (v/v, 4 mL) was stirred for 5 min at rt. Then, 4-bromo-1,3-benzothiazol-2-amine hydrochloride (53 mg, 1 eq.) was added and the reaction was stirred at rt for 48 h. LCMS showed > 50% conversion. After removal of DCM and filtering, the crude was purified by hplc using 0-70% of ACN in H_2O (+ 0.1 % TFA) for 30 min to give desired product as white TFA salt solid (35 mg, 31% yield). LCMS (ESI+): calculated for $C_{21}H_{23}BrN_3OS$ $(M+H)^+$: 444.1; found 444.1.
^{1}H NMR (400 MHz, CD_3OD) δ 7.84 (dd, J = 7.9, 1.0 Hz, 1H), 7.63 (dd, J = 7.9, 1.0 Hz, 1H), 7.31-7.19 (m, 4H), 7.19 (t, J = 7.9 Hz, 1H), 4.32-4.21 (m, 1H), 3.55-3.18 (m, 6H), 2.94 (s, 3H), 2.73 (t, J = 6.8 Hz, 2H), 2.33-2.10 (m, 2H).
^{13}C NMR (100 MHz, CD_3OD) δ 172.6, 156.7, 148.5, 139.9, 134.5, 130.7, 128.8, 125.9, 125.6, 121.8, 115.6, 67.0, 55.1, 49.0, 38.6, 32.8, 20.3.

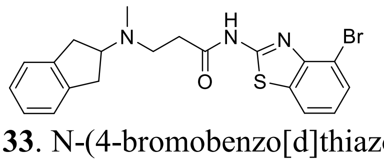

33. N-(4-bromobenzo[d]thiazol-2-yl)-3-((2,3-dihydro-1H-inden-2-yl)(methyl)amino)propanamide.
To a mixture of 3-[indan-2-yl(methyl)amino]propanoic acid (43.9 mg, 0.2 mmol, 1 eq.), HATU (152 mg, 2 eq.), DIEA (70 µL, 2 eq.) in 3/1 mixture of DCM/DMF (v/v, 4 mL) was stirred for 5 min at rt. Then, 4-bromo-1,3-benzothiazol-2-amine hydrochloride (53 mg, 1 eq.) was added and the reaction was stirred at rt for 48 h. LCMS showed > 50% conversion. After removal of DCM and filtering, the crude was purified by hplc using 0-70% of ACN in H_2O

(+ 0.1% TFA) for 30 min to give desired product as yellow TFA salt solid (5 mg, 5% yield). LCMS (ESI+): calculated for $C_{20}H_{21}BrN_3OS$ (M+H)$^+$: 430.1; found 430.1.
^{1}H NMR (400 MHz, CD_3OD) δ 7.86 (dd, *J* = 7.9, 0.9 Hz, 1H), 7.64 (dd, *J* = 7.9, 0.9 Hz, 1H), 7.30-7.21 (m, 4H), 7.21 (t, *J* = 7.9 Hz, 1H), 4.37-4.27 (m, 1H), 3.91-3.31 (m, 6H), 3.14-3.09 (m, 2H), 2.94 (s, 3H).
^{13}C NMR (100 MHz, CD_3OD) δ 170.5, 159.4, 148.5, 140.0, 134.5, 130.8, 128.8, 126.1, 125.6, 121.8, 115.7, 68.0, 51.1, 39.0, 35.8, 35.8, 30.9.

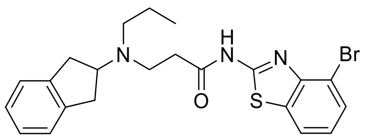

41. N-(4-bromobenzo[d]thiazol-2-yl)-3-((2,3-dihydro-1H-inden-2-yl)(propyl)amino)propanamide.
To a mixture of 4-[indan-2-yl(propyl)amino]propanoic acid TFA salt (32 mg, 0.1 mmol, 1 eq.), HATU (76 mg, 2 eq.), DIEA (51 μL, 4 eq.) in 3/1 mixture of DCM/DMF (v/v, 4 mL) was stirred for 5 min at rt. Then, 4-bromo-1,3-benzothiazol-2-amine hydrochloride (20 mg, 1 eq.) was added and the reaction was stirred at rt for 48 h. After removal of DCM and filtering, the crude was purified by hplc using 0-70% of ACN in H_2O (+ 0.1 % TFA) for 30min to give desired product as white TFA salt solid (3 mg, 5% yield). LCMS (ESI+): calculated for $C_{22}H_{25}BrN_3OS$ (M+H)$^+$: 458.1; found 458.2.
^{1}H NMR (500 MHz, CD_3OD) δ 7.86 (d, *J* = 7.9 Hz, 1H), 7.64 (d, *J* = 7.9 Hz, 1H), 7.34-7.21 (m, 4H), 7.21 (t, *J* = 7.9 Hz, 1H), 4.44-4.35 (m, 1H), 3.73-3.31 (m, 6H), 3.24 (t, *J* = 8.3 Hz, 2H), 3.12 (t, *J* = 6.5 Hz, 2H), 1.96-1.78 (m, 2H), 1.06 (t, *J* = 7.1 Hz, 3H).
^{13}C NMR (125 MHz, CD_3OD) δ 170.3, 159.4, 148.5, 139.9, 134.5, 130.8, 128.8, 126.1, 125.6, 121.8, 115.7, 65.3, 54.5, 48.0, 35.9, 31.0, 18.4, 11.2.

General synthesis for compounds **34-40**
To a mixture of 2-aminobenzothiazole derivatives (0.05 mmol), $NaHCO_3$ (1 equiv.) in THF (1 mL) at 0 °C was added 3-bromo propionyl chloride. The mixture was stirred at rt for 30 min. Solvent was evaporated to dryness and the residue was dissolved in DMF (1 mL) then N-alkyl-2-aminoindane (20 μL) and $NaHCO_3$ (1 equiv.) were added. The mixture was stirred at 50 °C for overnight. The mixture was filtered and purified by hplc using a gradient of 20 to 70% of CH_3CN in H_2O (+ 0.1 % TFA) to afford desired products as white TFA salt solid.

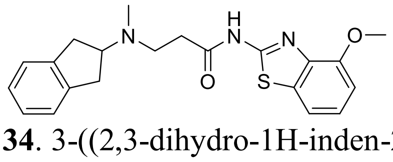

34. 3-((2,3-dihydro-1H-inden-2-yl)(methyl)amino)-N-(4-methoxybenzo[d]thiazol-2-yl)propanamide.
Yield: 8 mg (16%).
LCMS (ESI+): calculated for $C_{21}H_{24}N_3O_2S$ (M+H)$^+$: 382.2; found 382.3.
^{1}H NMR (600 MHz, CD_3OD) δ 7.44 (dd, *J* = 8.0, 0.6 Hz, 1H), 7.33-7.21 (m, 5H), 7.01 (d, *J* = 7.9 Hz, 1H), 4.34-4.29 (m, 1H), 4.00 (s, 3H), 3.88-3.42 (m, 4H), 3.38-3.31 (m, 2H), 3.14-3.10 (m, 2H), 2.94 (s, 3H). ^{13}C NMR (150 MHz, CD_3OD) δ 170.4, 158.1, 153.5, 140.0, 134.7, 128.8, 126.3, 125.6, 114.5, 108.7, 68.0, 56.6, 51.2, 39.0, 35.8, 30.9.

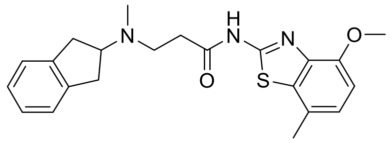

35. 3-((2,3-dihydro-1H-inden-2-yl)(methyl)amino)-N-(4-methoxy-7-methylbenzo[d]thiazol-2-yl)propanamide.
Yield: 18 mg (35%).
LCMS (ESI+): calculated for $C_{22}H_{26}N_3O_2S$ $(M+H)^+$: 396.2; found 396.3.
^{1}H NMR (600 MHz, CD_3OD) δ 7.31-7.23 (m, 4H), 7.08 (d, *J* = 8.0 Hz, 1H), 6.93 (d, *J* = 8.0 Hz, 1H), 4.34-4.29 (m, 1H), 3.97 (s, 3H), 3.95-3.42 (m, 4H), 3.38-3.34 (m, 2H), 3.14-3.12 (m, 2H), 2.94 (s, 3H), 2.45 (s, 3H).
^{13}C NMR (150 MHz, CD_3OD) δ 170.3, 157.8, 151.7, 140.0, 138.9, 134.4, 128.8, 126.2, 125.6, 124.4, 109.0, 67.9, 56.6, 51.2, 39.0, 35.8, 31.0, 19.9.

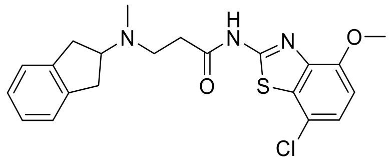

36. N-(7-chloro-4-methoxybenzo[d]thiazol-2-yl)-3-((2,3-dihydro-1H-inden-2-yl)(methyl)amino)propanamide.
Yield: 13 mg (24%).
LCMS (ESI+): calculated for $C_{21}H_{23}ClN_3O_2S$ $(M+H)^+$: 416.1; found 416.2.
^{1}H NMR (600 MHz, CD_3OD) δ 7.32-7.21 (m, 4H), 7.28 (d, *J* = 8.6 Hz, 1H), 7.01 (d, *J* = 8.6 Hz, 1H), 4.35-4.30 (m, 1H), 4.00 (s, 3H), 3.88-3.41 (m, 4H), 3.38-3.334 (m, 2H), 3.16-3.13 (m, 2H), 2.94 (s, 3H).
^{13}C NMR (150 MHz, CD_3OD) δ 170.6, 158.3, 152.5, 140.0, 133.9, 128.8, 125.6, 125.5, 118.7, 110.2, 68.0, 57.0, 51.1, 39.0, 35.8, 30.9.

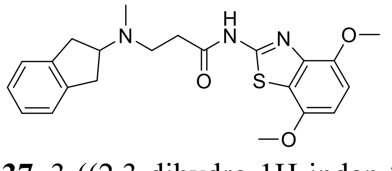

37. 3-((2,3-dihydro-1H-inden-2-yl)(methyl)amino)-N-(4,7-dimethoxybenzo[d]thiazol-2-yl)propanamide.
Yield: 21 mg (40%).
LCMS (ESI+): calculated for $C_{22}H_{26}N_3O_3S$ $(M+H)^+$: 412.2; found 412.3.
^{1}H NMR (600 MHz, CD_3OD) δ 7.32-7.21 (m, 4H), 6.93 (d, *J* = 8.6 Hz, 1H), 6.81 (d, *J* = 8.6 Hz, 1H), 4.34-4.29 (m, 1H), 3.95 (s, 3H), 3.93 (s, 3H), 3.83-3.42 (m, 4H), 3.38-3.334 (m, 2H), 3.15-3.10 (m, 2H), 2.94 (s, 3H).
^{13}C NMR (150 MHz, CD_3OD) δ 170.4, 158.8, 149.7, 148.0, 140.8, 140.0, 128.8, 125.6, 123.0, 109.4, 105.8, 68.0, 57.1, 56.6, 51.2, 39.0, 35.8, 30.9.

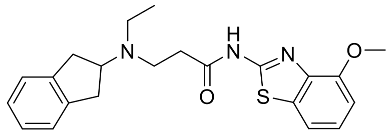

38. 3-((2,3-dihydro-1H-inden-2-yl)(ethyl)amino)-N-(4-methoxybenzo[d]thiazol-2-yl)propanamide.
Yield: 3 mg (6%).
LCMS (ESI+): calculated for $C_{22}H_{26}N_3O_2S$ $(M+H)^+$: 396.2; found 396.3.

^{1}H NMR (600 MHz, CD_3OD) δ 7.44 (dd, *J* = 8.0, 0.7 Hz, 1H), 7.31-7.22 (m, 5H), 7.01 (d, *J* = 7.9 Hz, 1H), 4.43-4.37 (m, 1H), 4.00 (s, 3H), 3.74-3.31 (m, 8H), 3.13-3.10 (m, 2H), 1.44 (t, *J* = 7.3 Hz, 3H).
^{13}C NMR (150 MHz, CD_3OD) δ 170.2, 153.5, 139.9, 134.7, 128.8, 126.3, 125.6, 114.5, 108.7, 64.8, 56.6, 47.9, 47.4, 35.9, 31.0, 9.1.

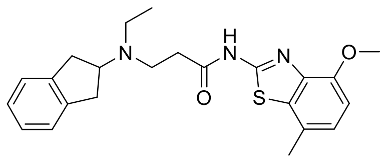

39. 3-((2,3-dihydro-1H-inden-2-yl)(ethyl)amino)-N-(4-methoxy-7-methylbenzo[d]thiazol-2-yl)propanamide.
Yield: 5 mg (9%).
LCMS (ESI+): calculated for $C_{23}H_{28}N_3O_2S$ $(M+H)^+$: 410.2; found 410.3.
^{1}H NMR (600 MHz, CD_3OD) δ 7.31-7.23 (m, 45H), 7.09 (d, *J* = 8.1 Hz, 1H), 6.93 (d, *J* = 8.1 Hz, 1H), 4.45-4.38 (m, 1H), 3.98 (s, 3H), 3.78-3.31 (m, 8H), 3.13-3.11 (m, 2H), 2.46 (s, 3H), 1.44 (t, *J* = 7.3 Hz, 3H).
^{13}C NMR (150 MHz, CD_3OD) δ 170.1, 157.8, 151.7, 140.0, 139.0, 128.8, 126.2, 125.6, 124.4, 109.0, 64.8, 56.6, 48.0, 47.4, 35.9, 31.1, 19.9, 9.1.

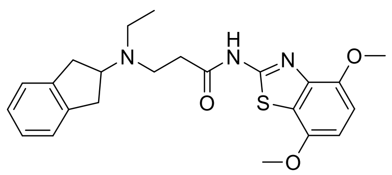

40. 3-((2,3-dihydro-1H-inden-2-yl)(ethyl)amino)-N-(4,7-dimethoxybenzo[d]thiazol-2-yl)propanamide.
Yield: 6 mg (11%).
LCMS (ESI+): calculated for $C_{23}H_{28}N_3O_3S$ $(M+H)^+$: 426.2; found 426.3.
^{1}H NMR (600 MHz, CD_3OD) δ 7.31-7.23 (m, 45H), 6.94 (d, *J* = 8.6 Hz, 1H), 6.81 (d, *J* = 8.6 Hz, 1H), 4.42-4.37 (m, 1H), 3.96 (s, 3H), 3.94 (s, 3H), 3.73-3.31 (m, 8H), 3.12-3.10 (m, 2H), 1.44 (t, *J* = 7.3 Hz, 3H).
^{13}C NMR (150 MHz, CD_3OD) δ 170.2, 158.8, 149.7, 148.0, 140.8, 139.9, 128.8, 125.6, 122.9, 109.4, 105.8, 64.8, 57.1, 56.6, 47.9, 47.4, 35.9, 31.0, 9.1.

Compound **2**, CD_3OD, 500 MHz

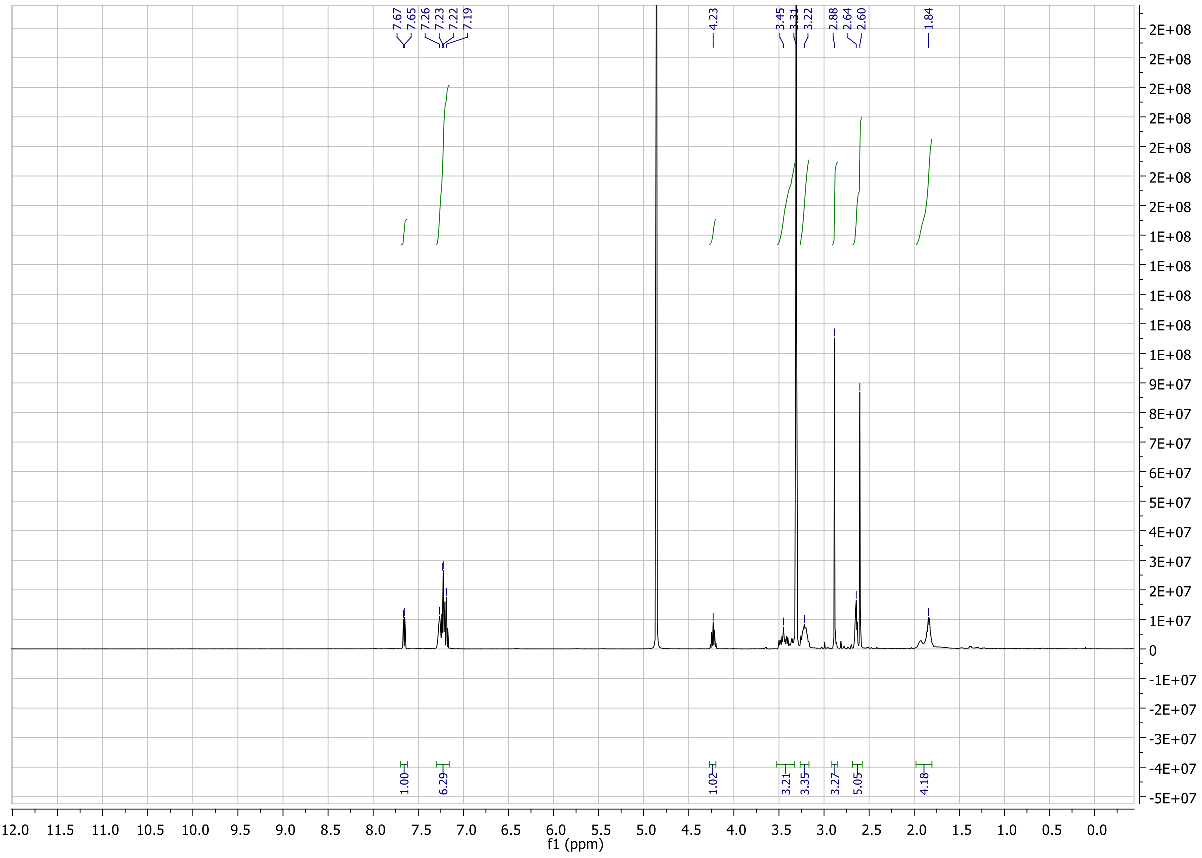

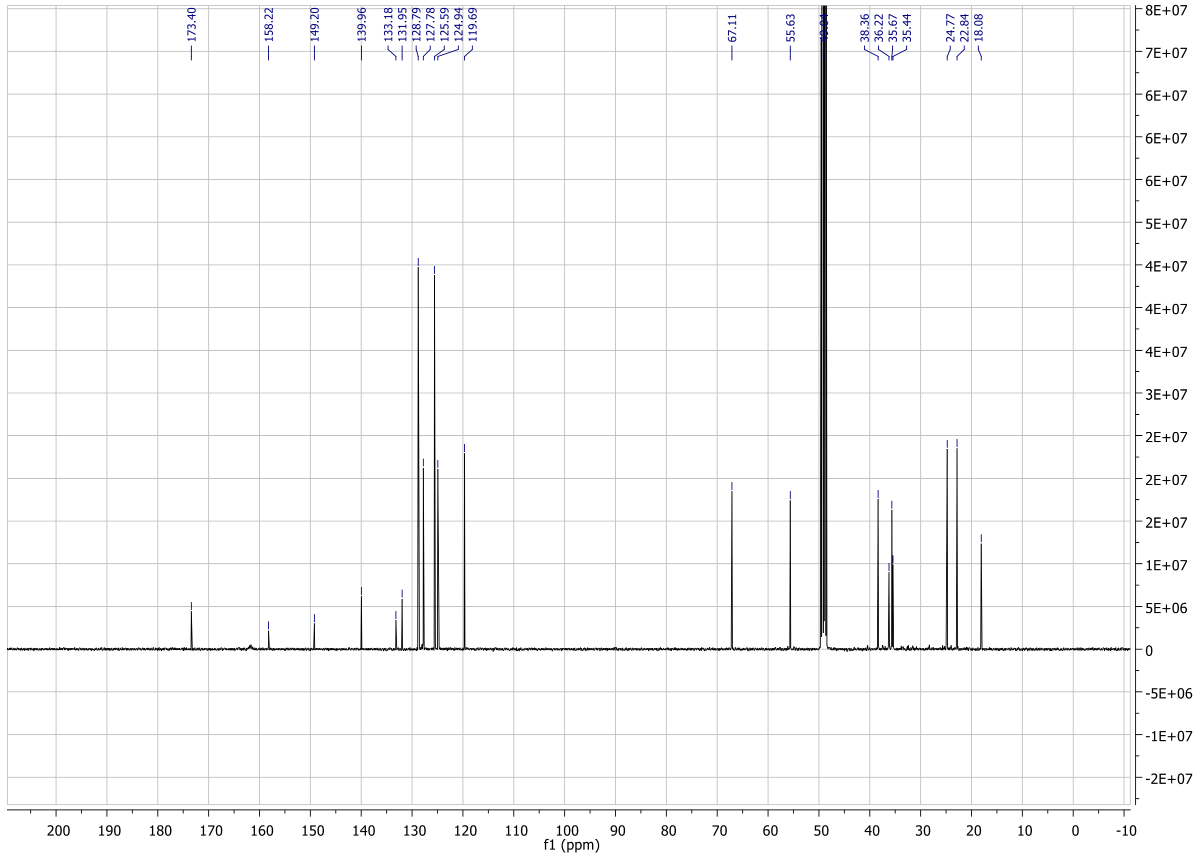

Compound **3**, $CDCl_3$, 400 MHz

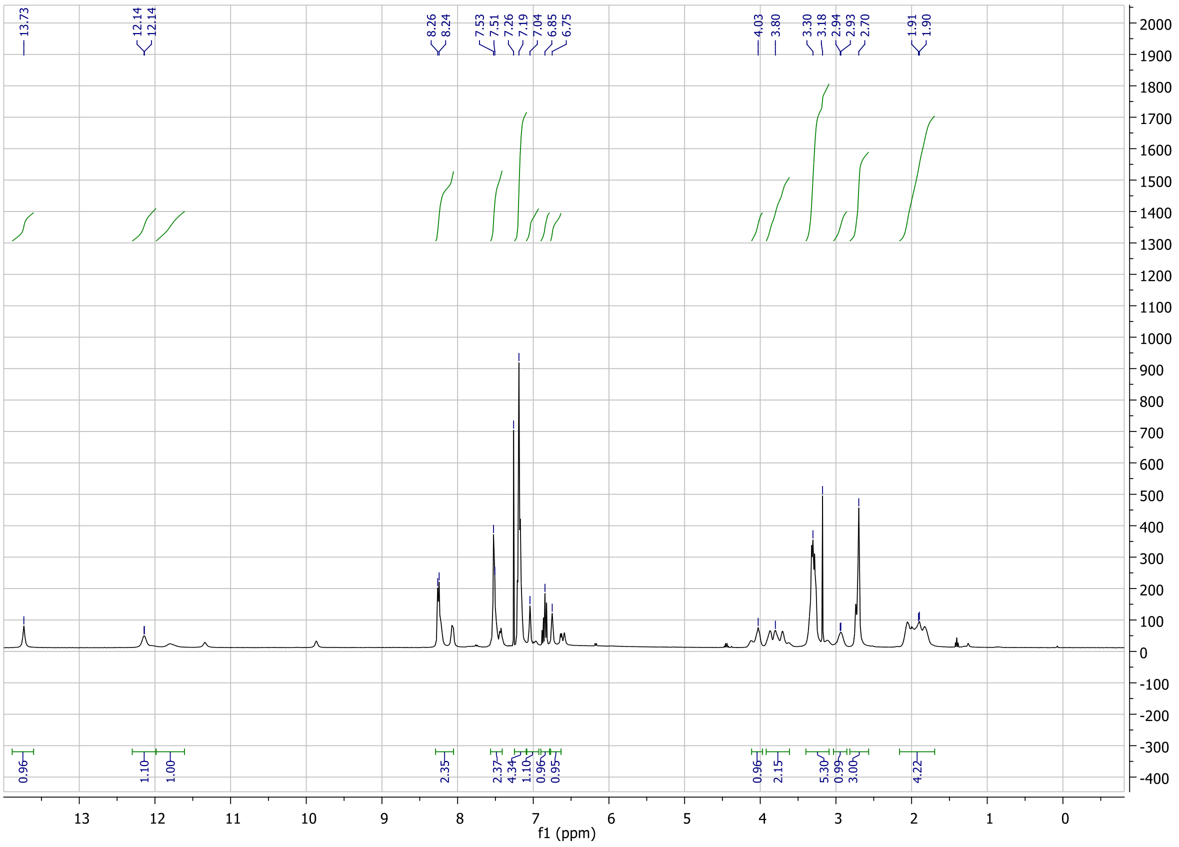

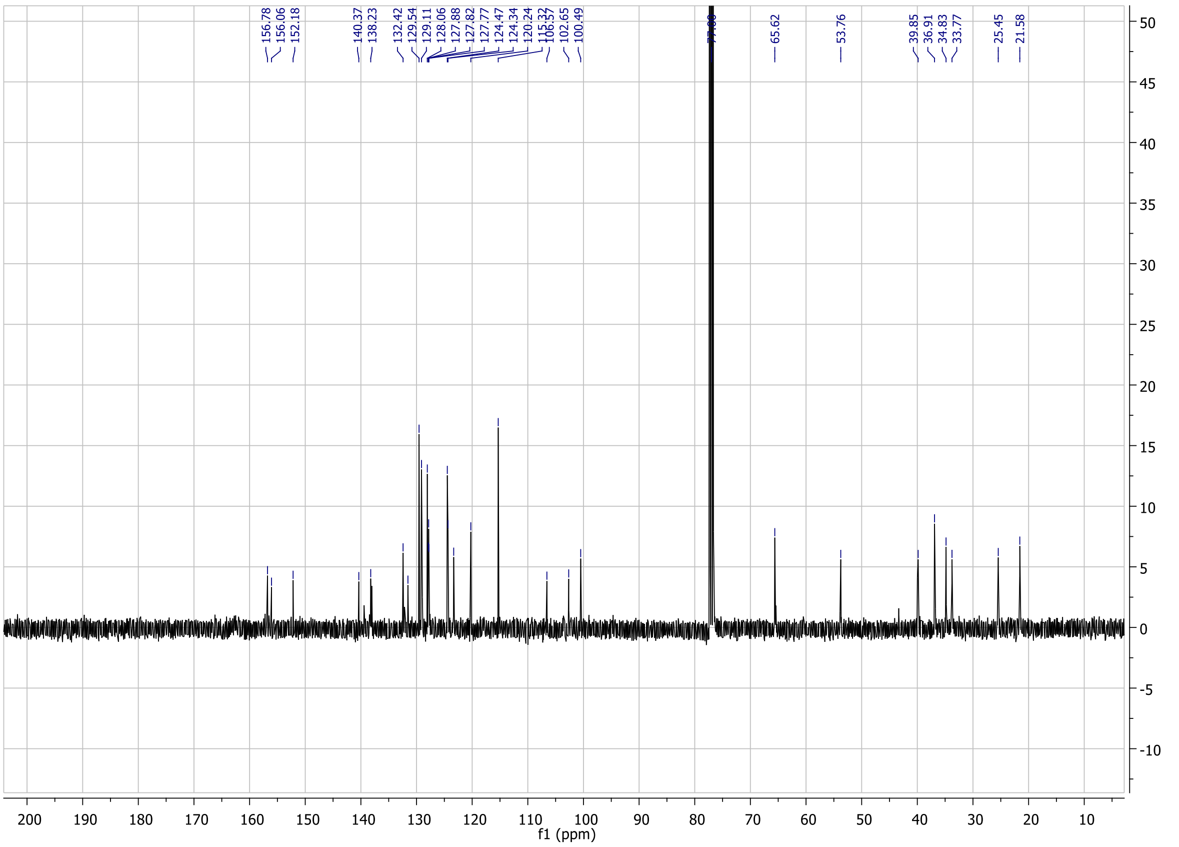

Compound **4**, $CDCl_3$, 400 MHz

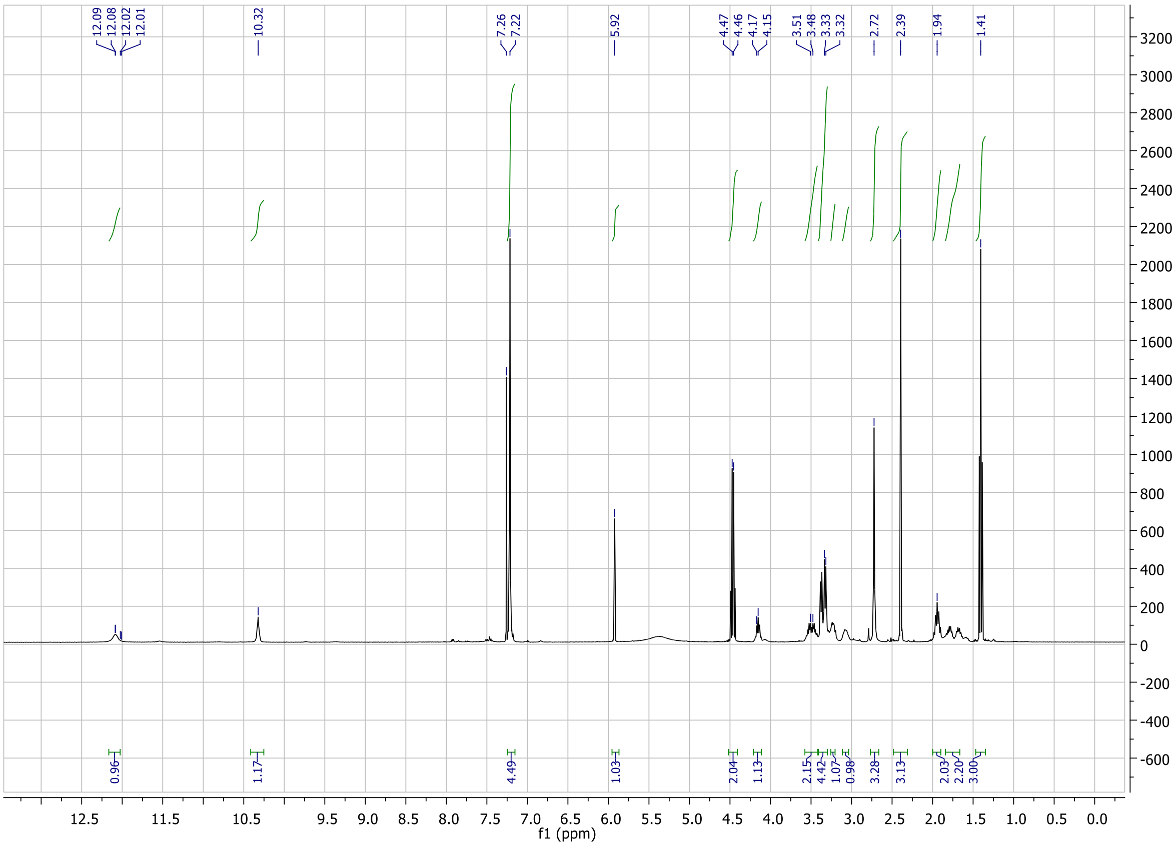

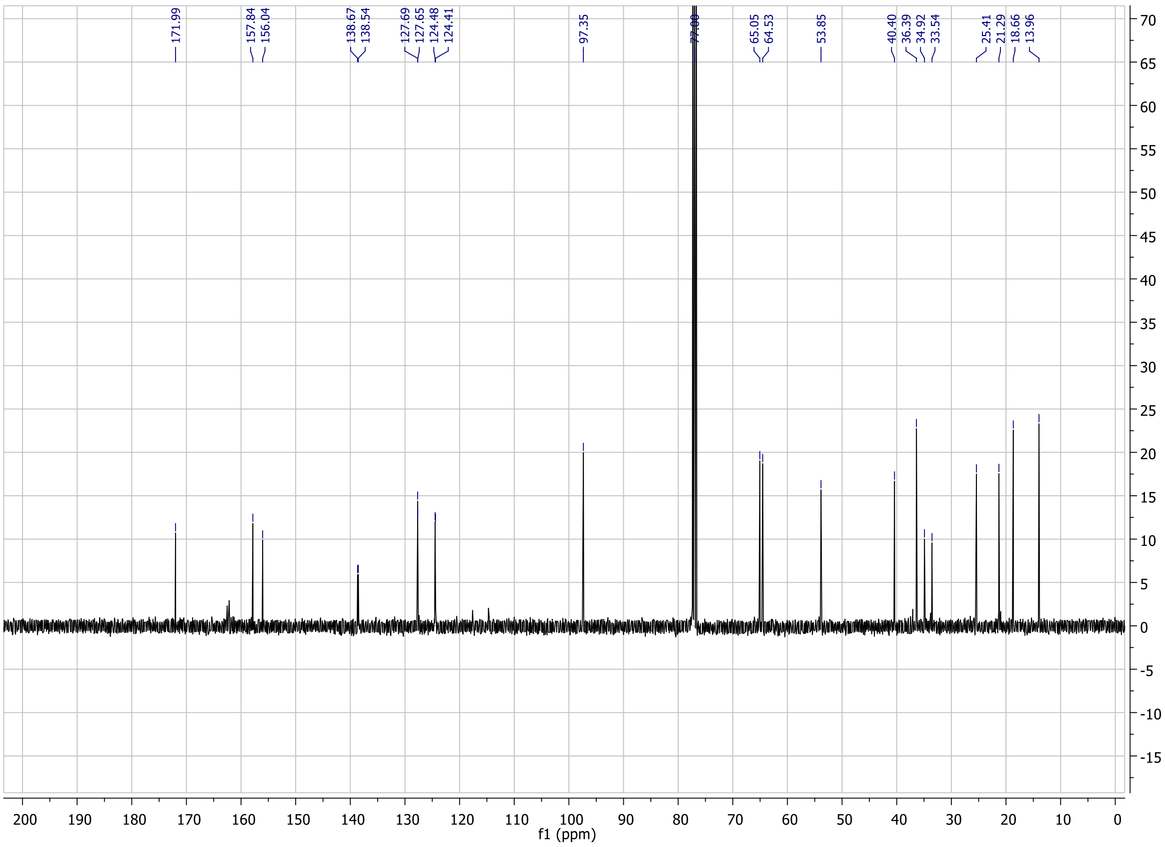

Compound **5**, $CDCl_3$, 400 MHz

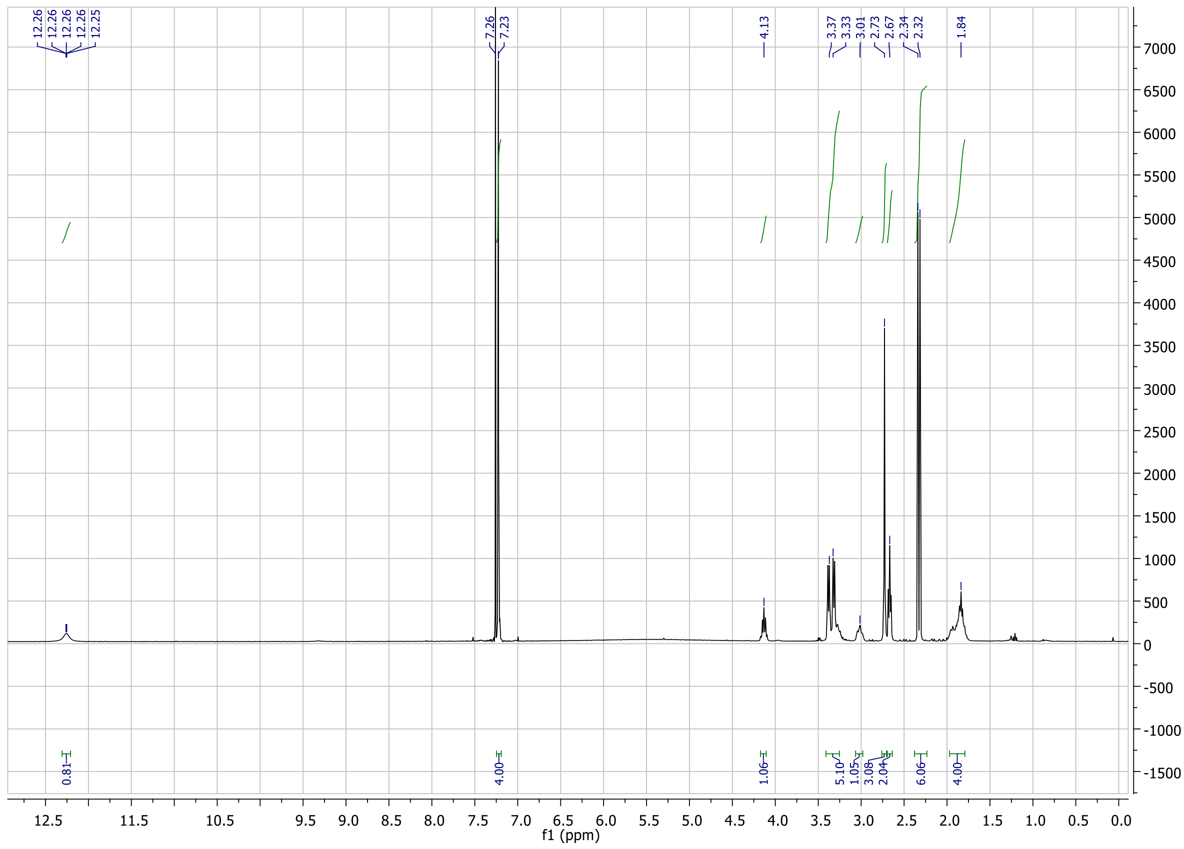

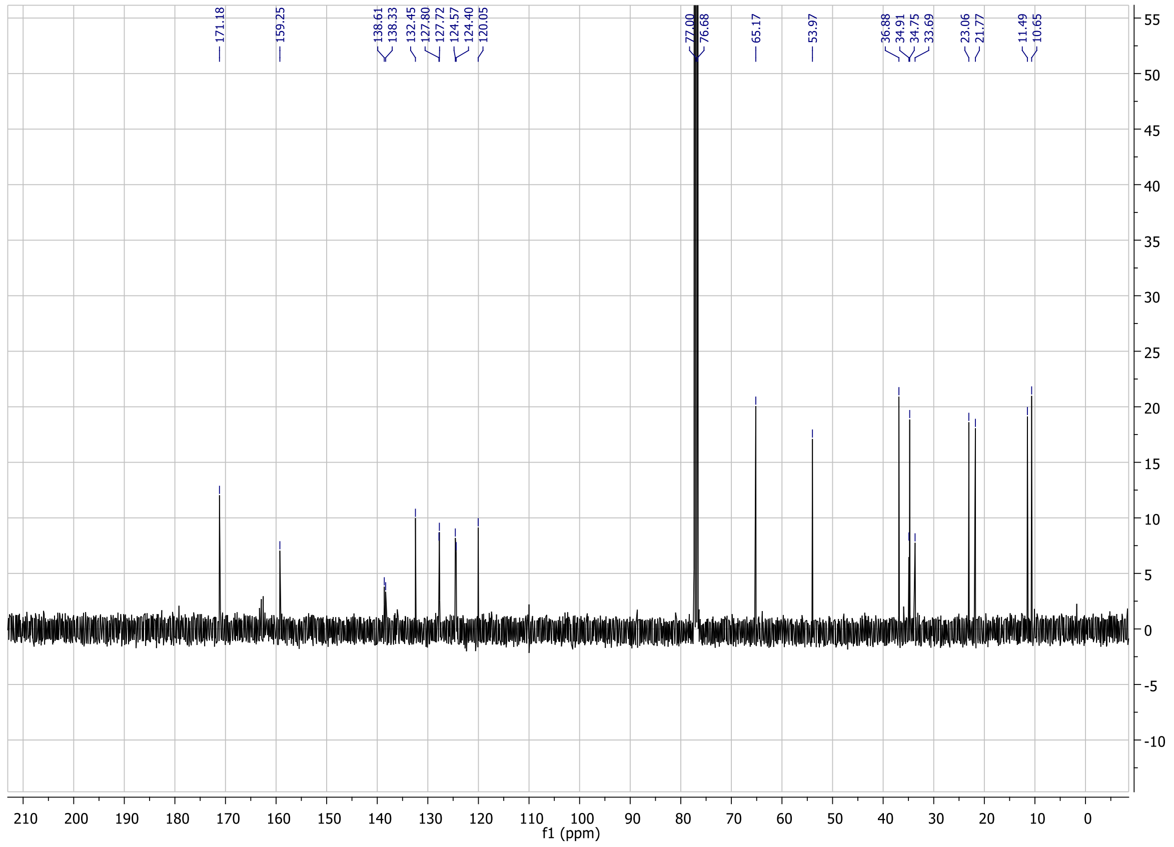

Compound **6**, 400 MHz

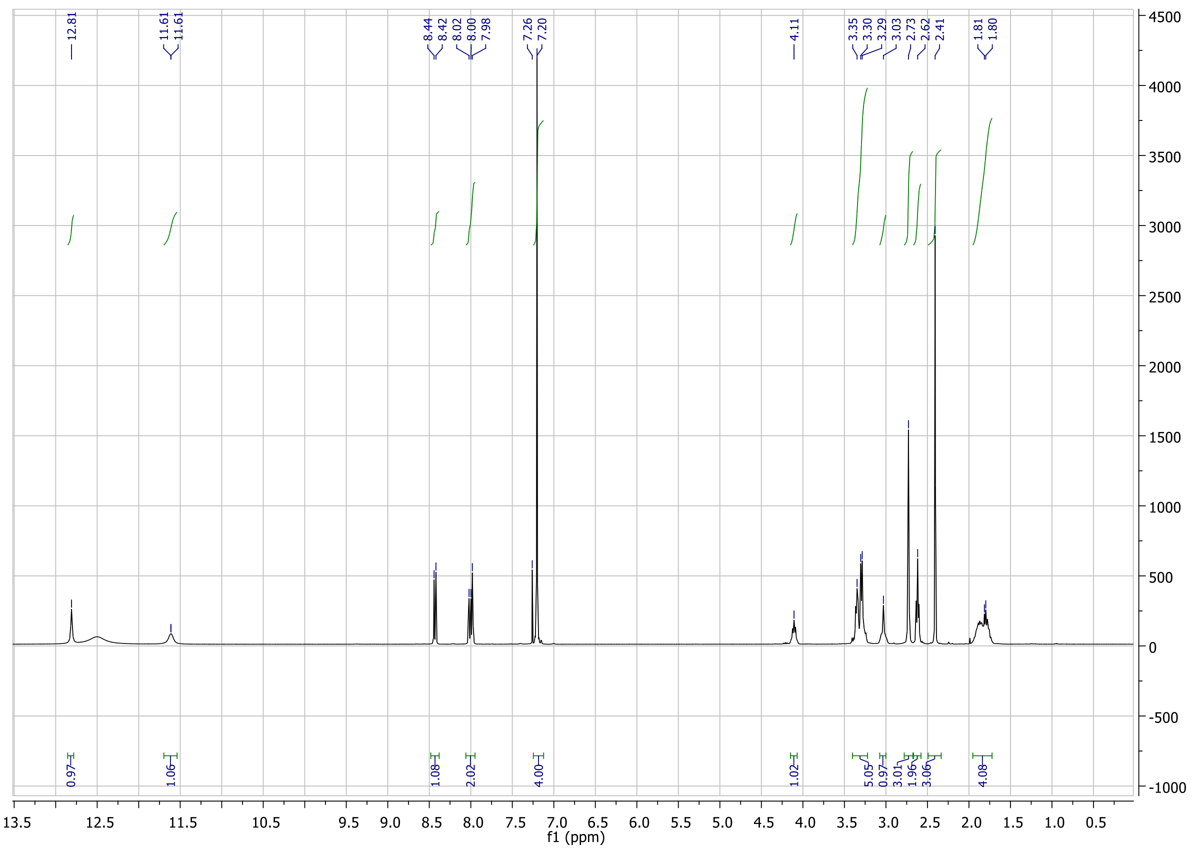

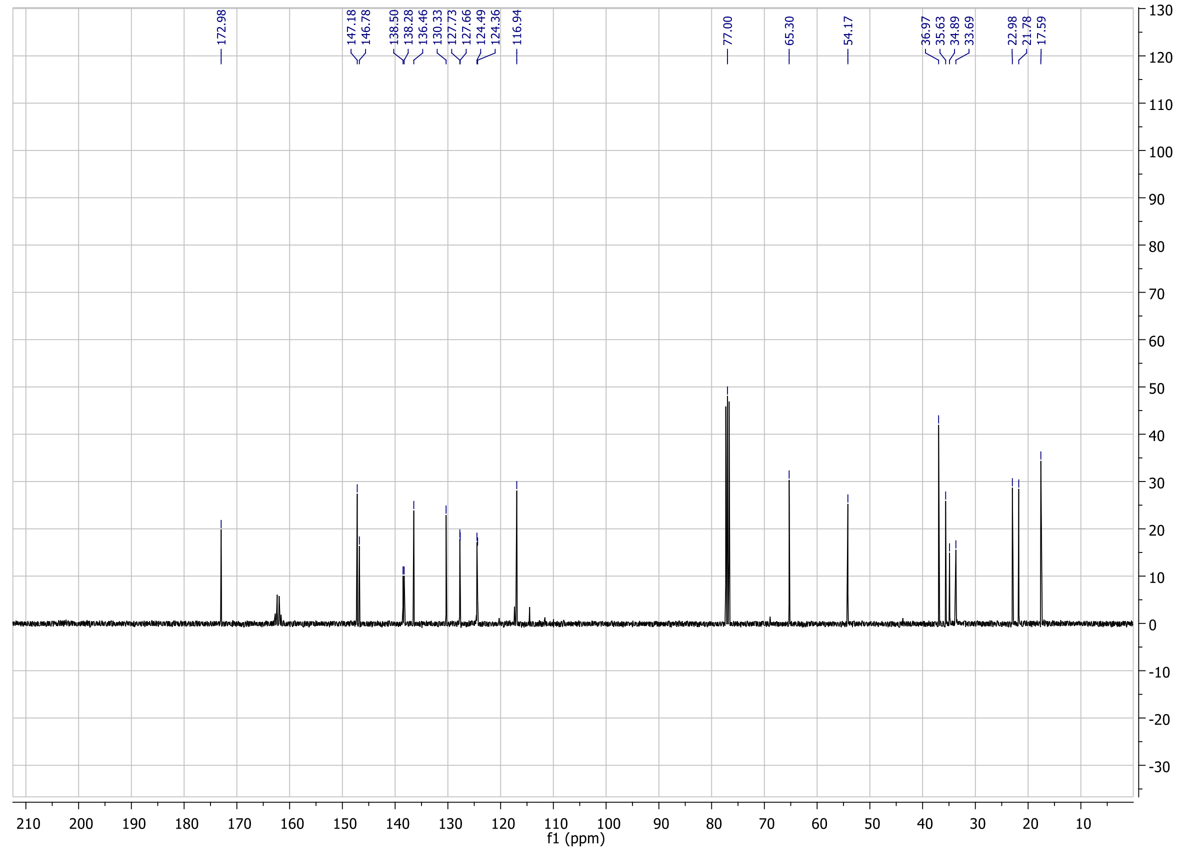

Compound **7**, $CDCl_3/CD_3OD$, 400 MHz

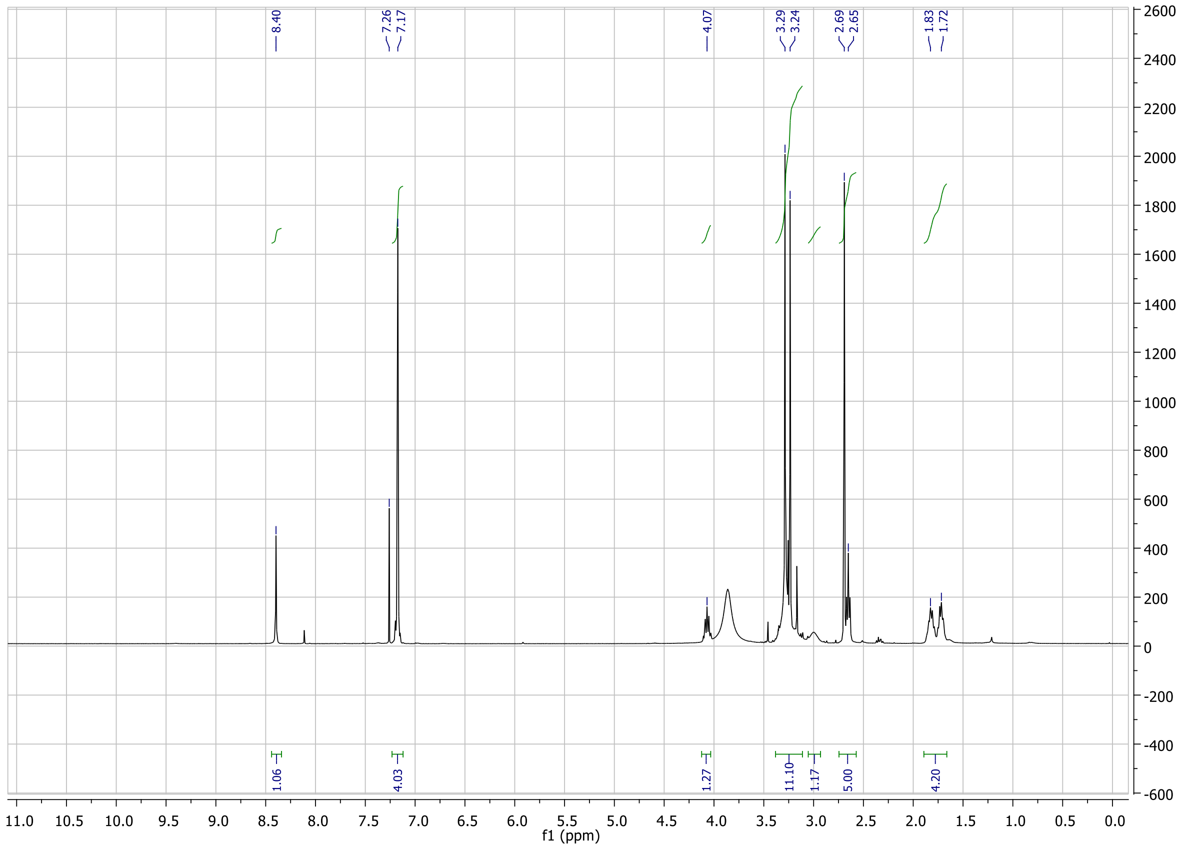

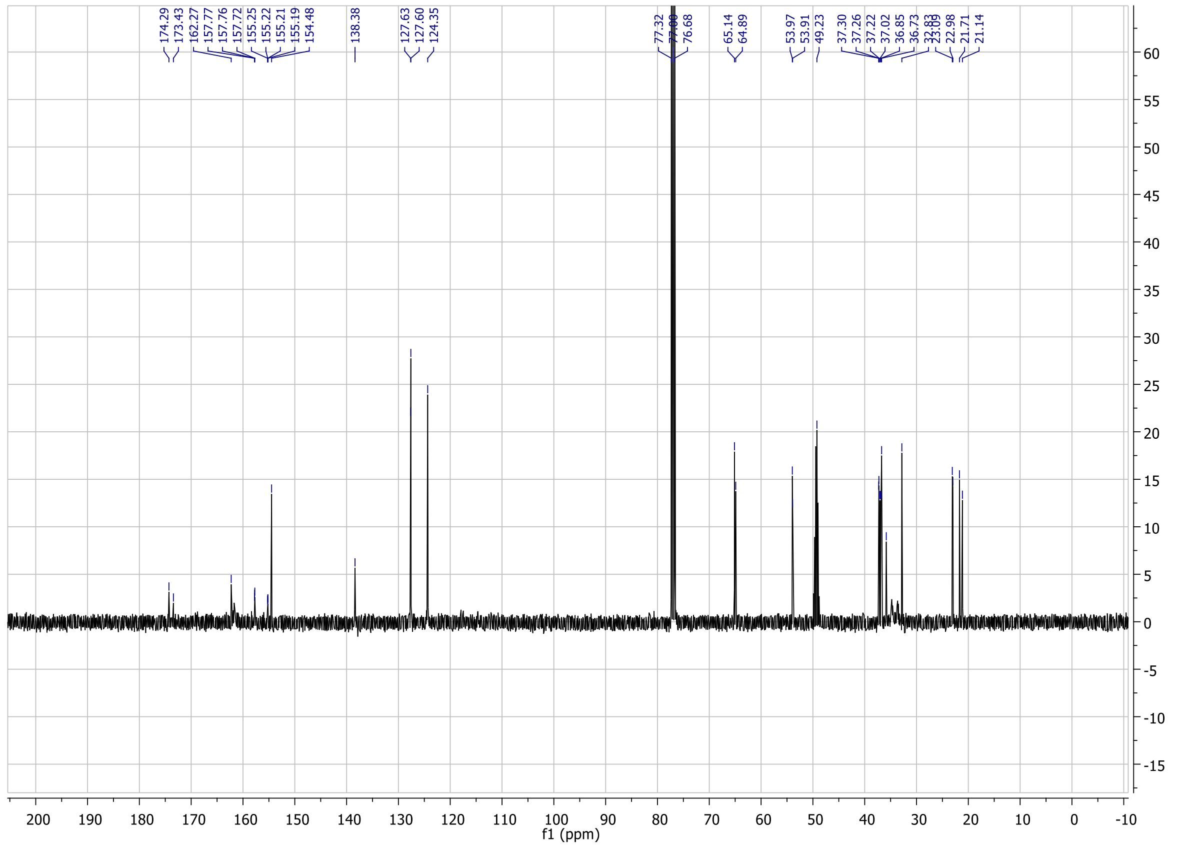

Compound **8**, $CDCl_3/CD_3OD$, 400 MHz

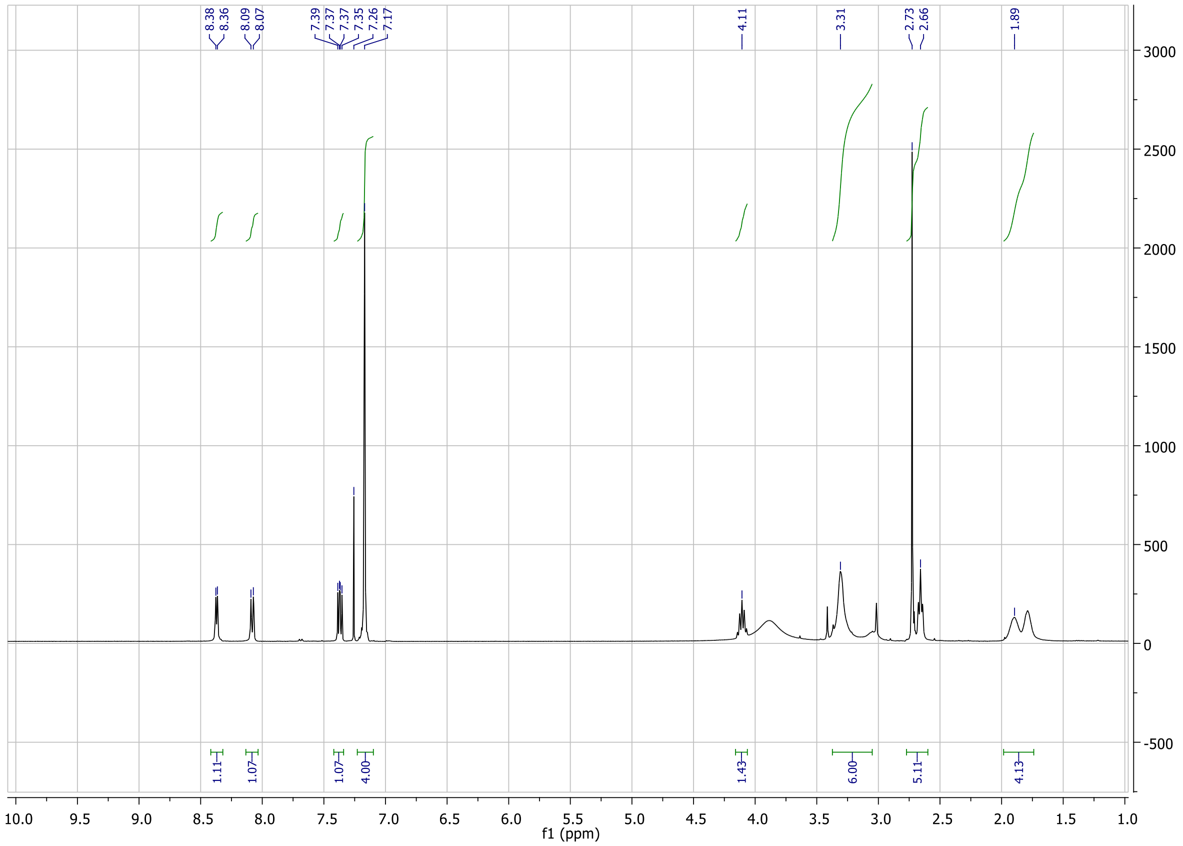

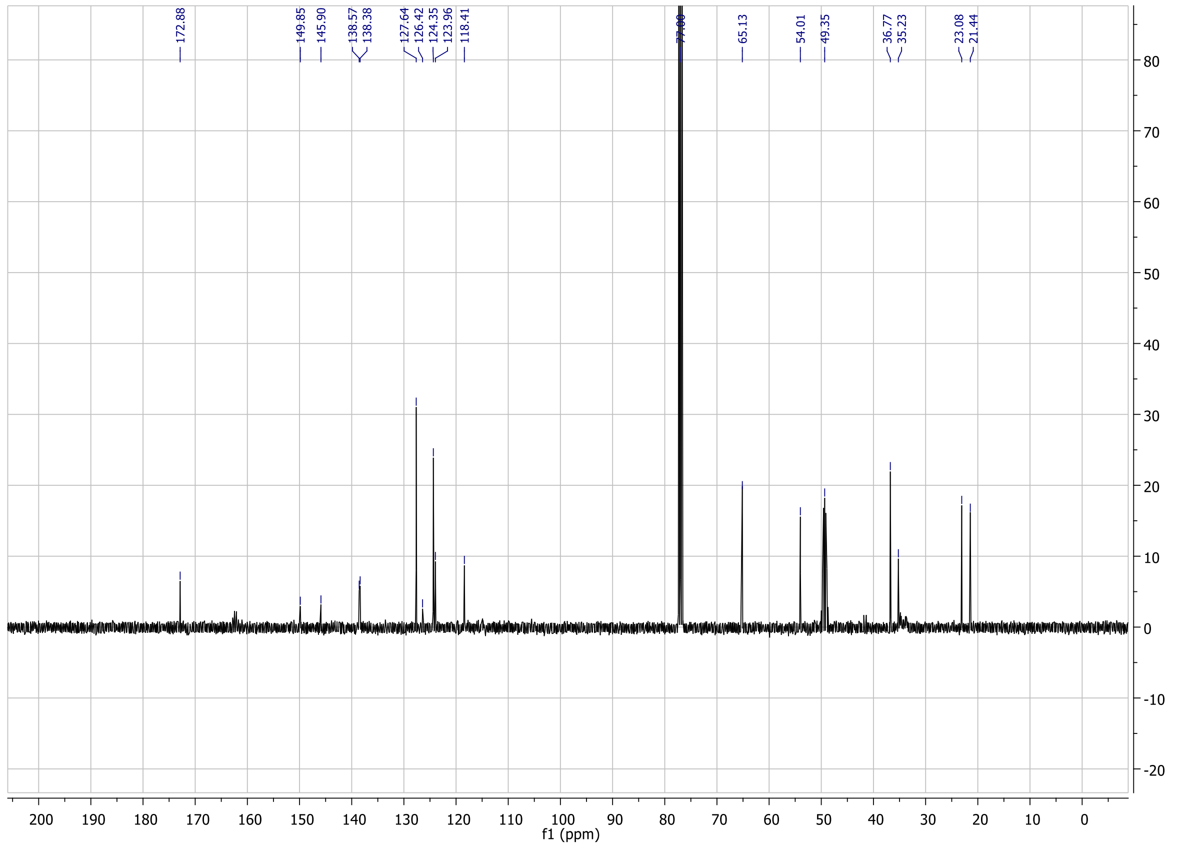

Compound **9**, $CDCl_3$, 400 MHz

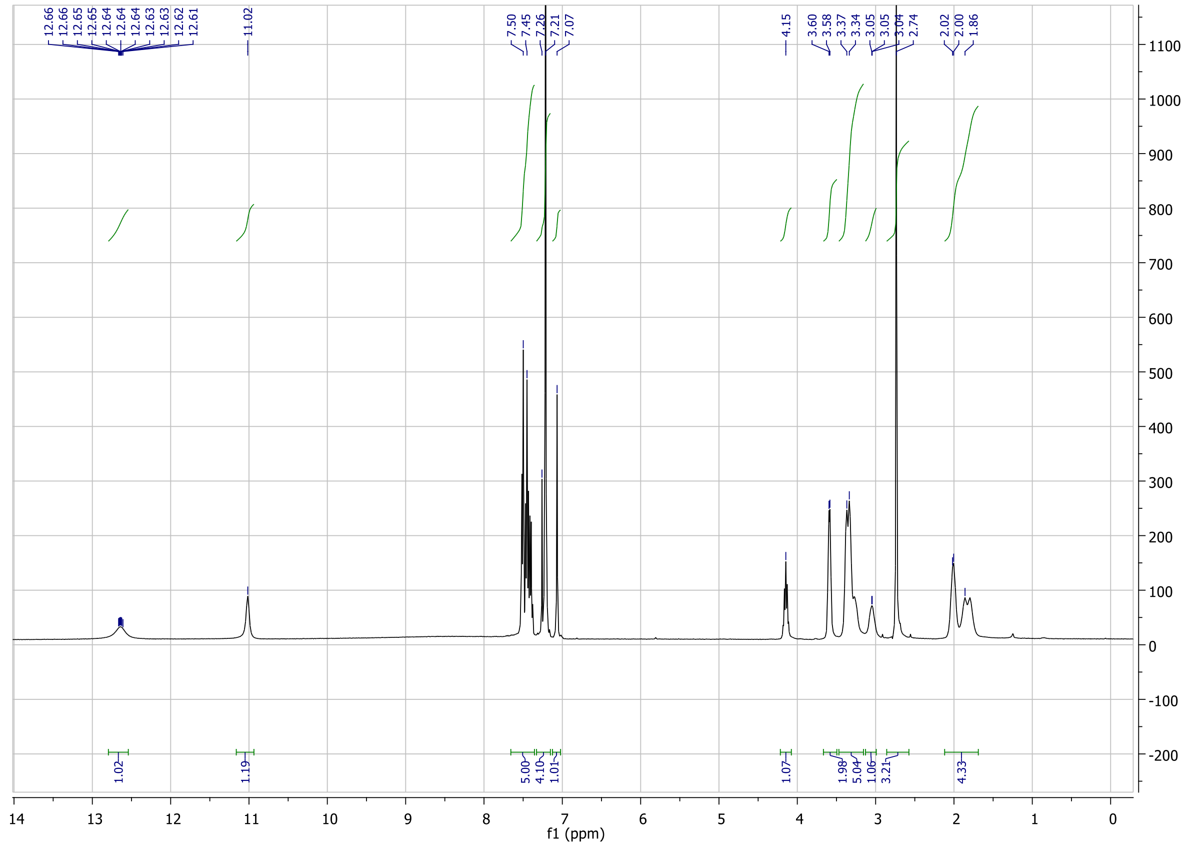

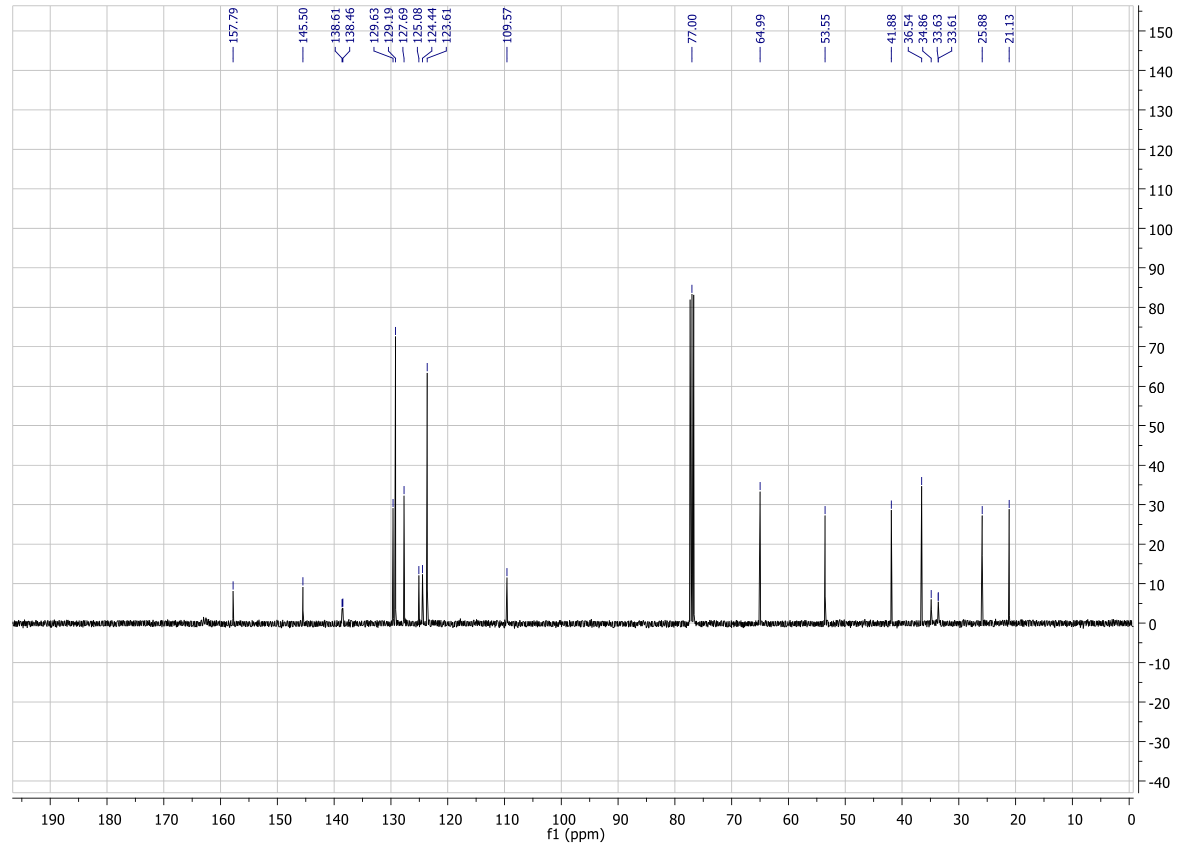

Compound **10**, $CDCl_3/CD_3OD$, 400 MHz

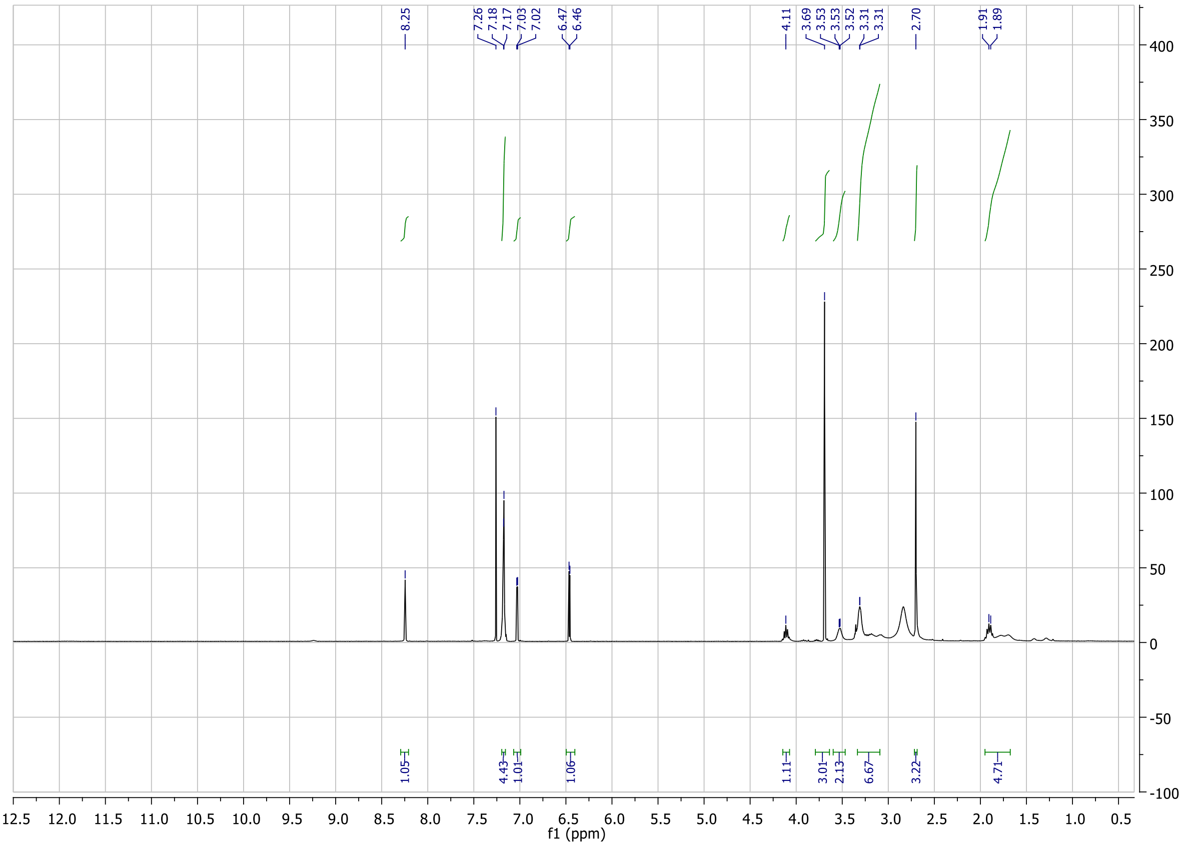

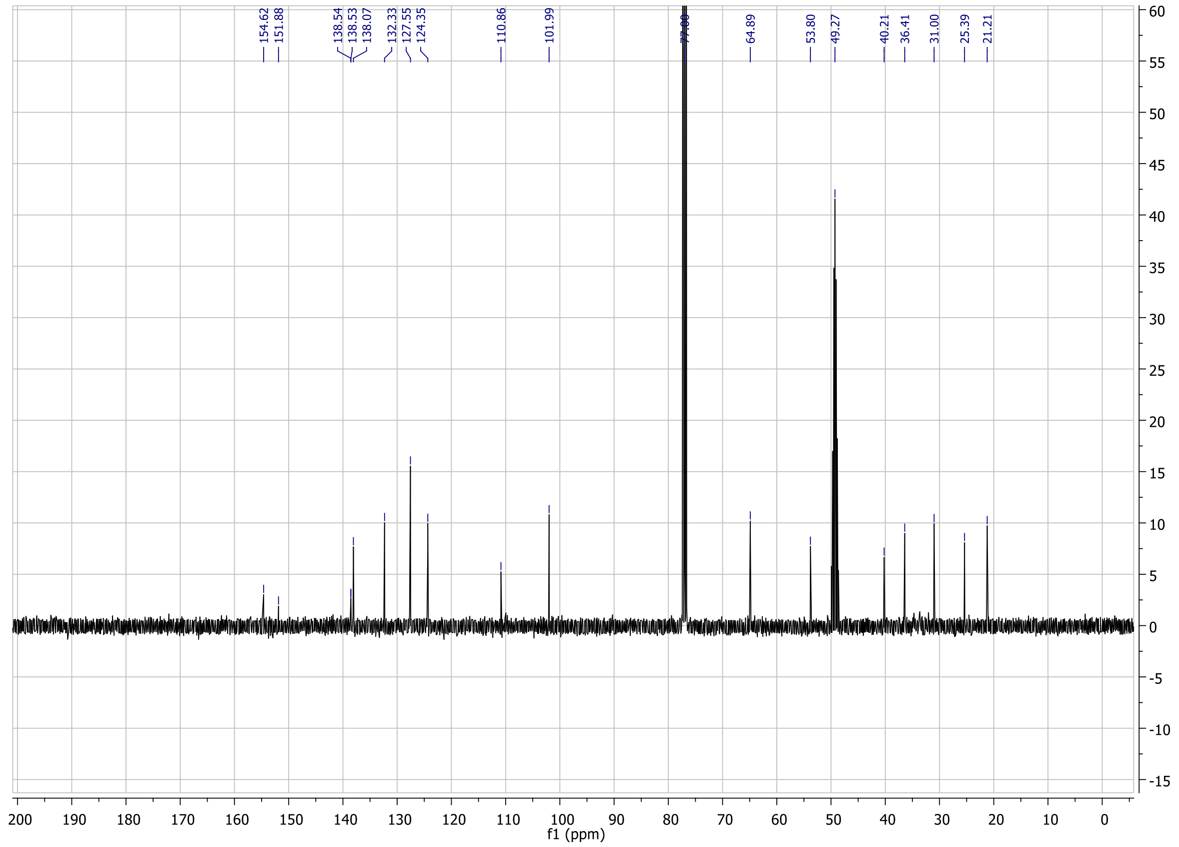

Compound **11**, $CDCl_3/CD_3OD$, 400 MHz

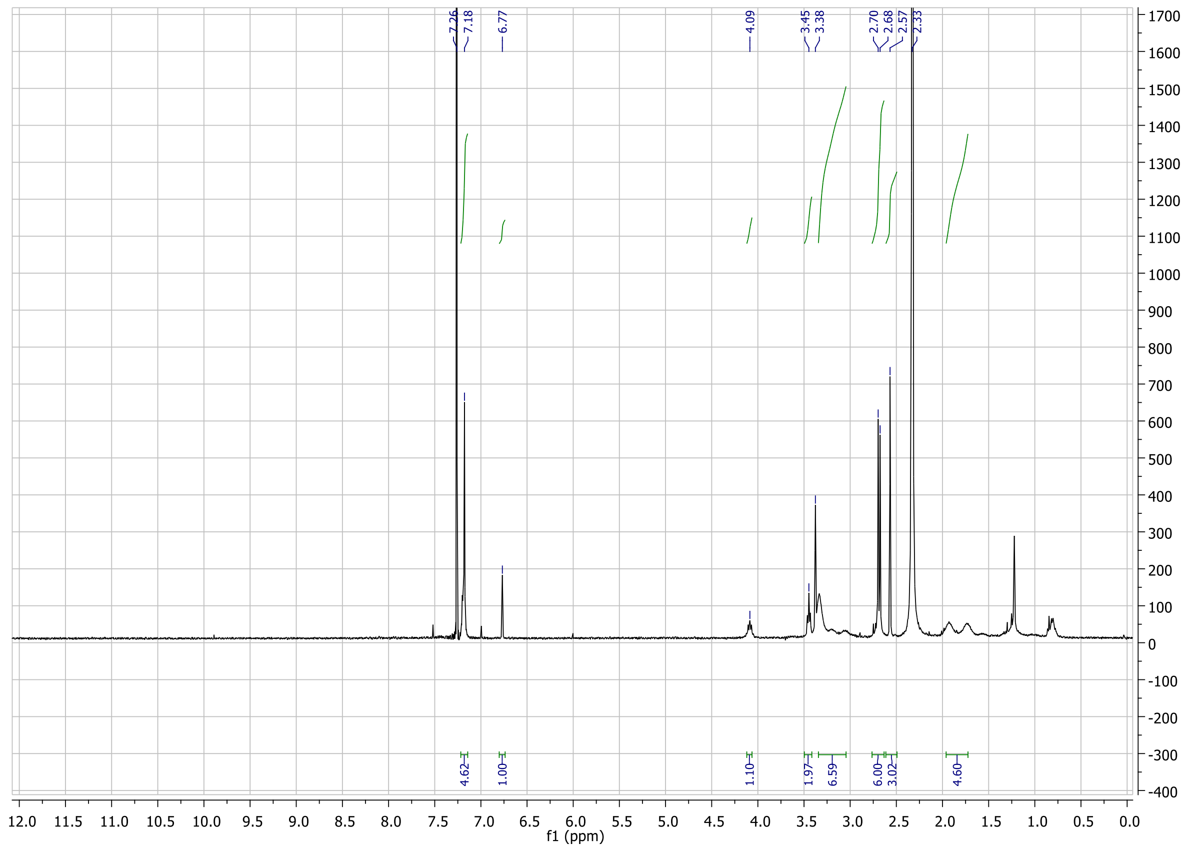

Compound **12**, $CDCl_3$, 400 MHz

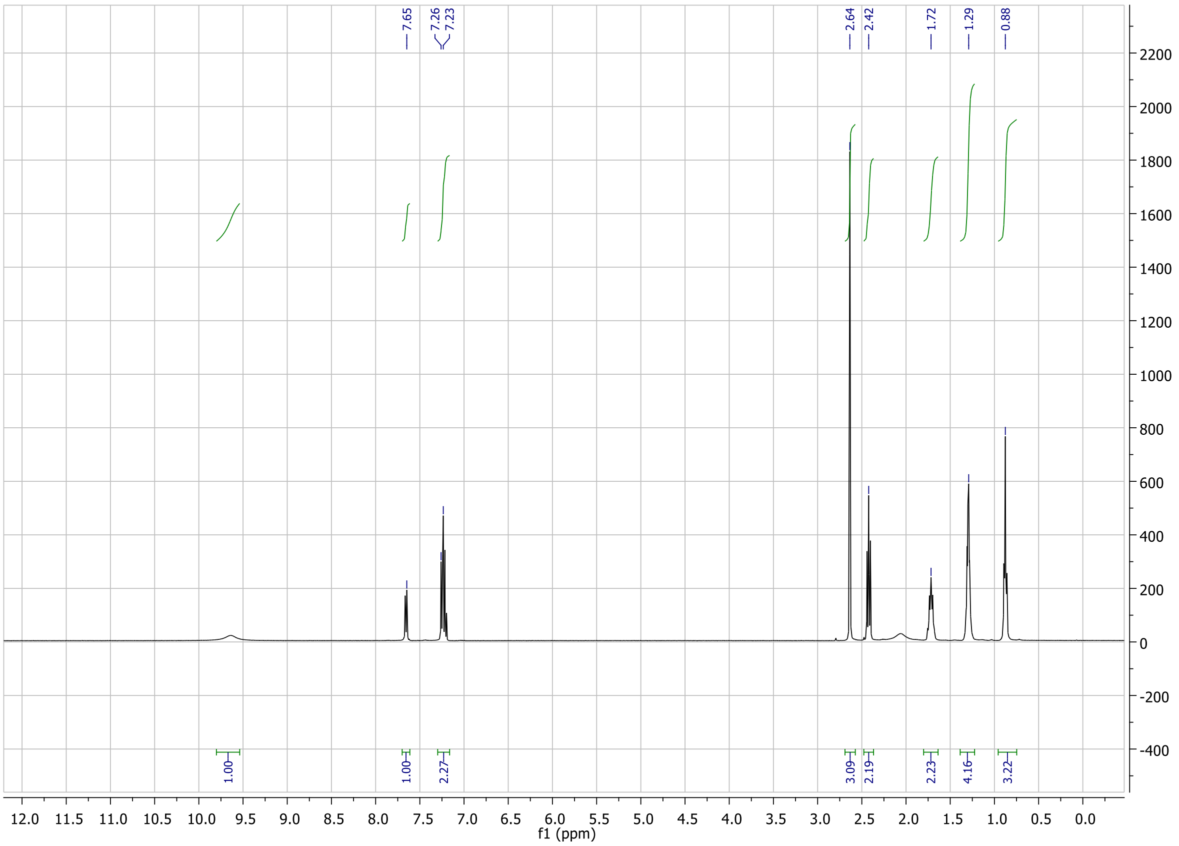

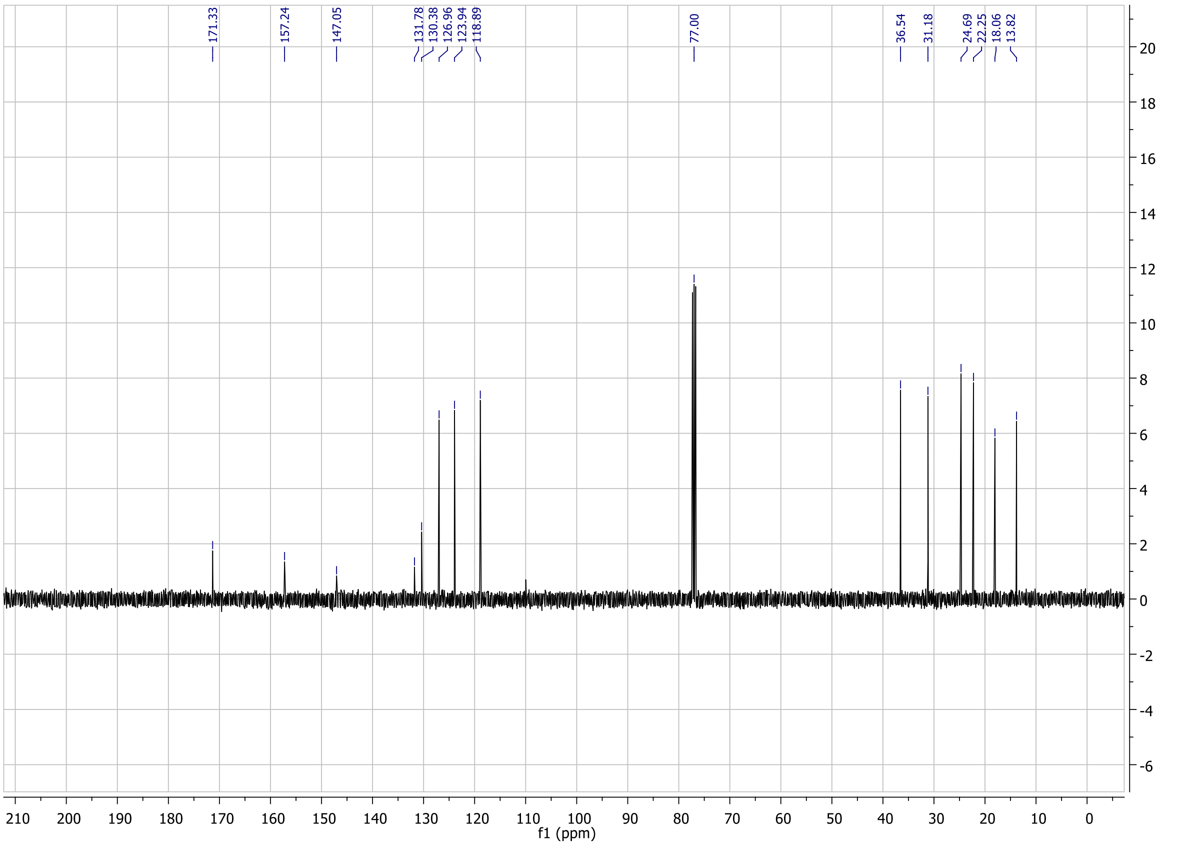

Compound **13**, $CDCl_3$, 400 MHz

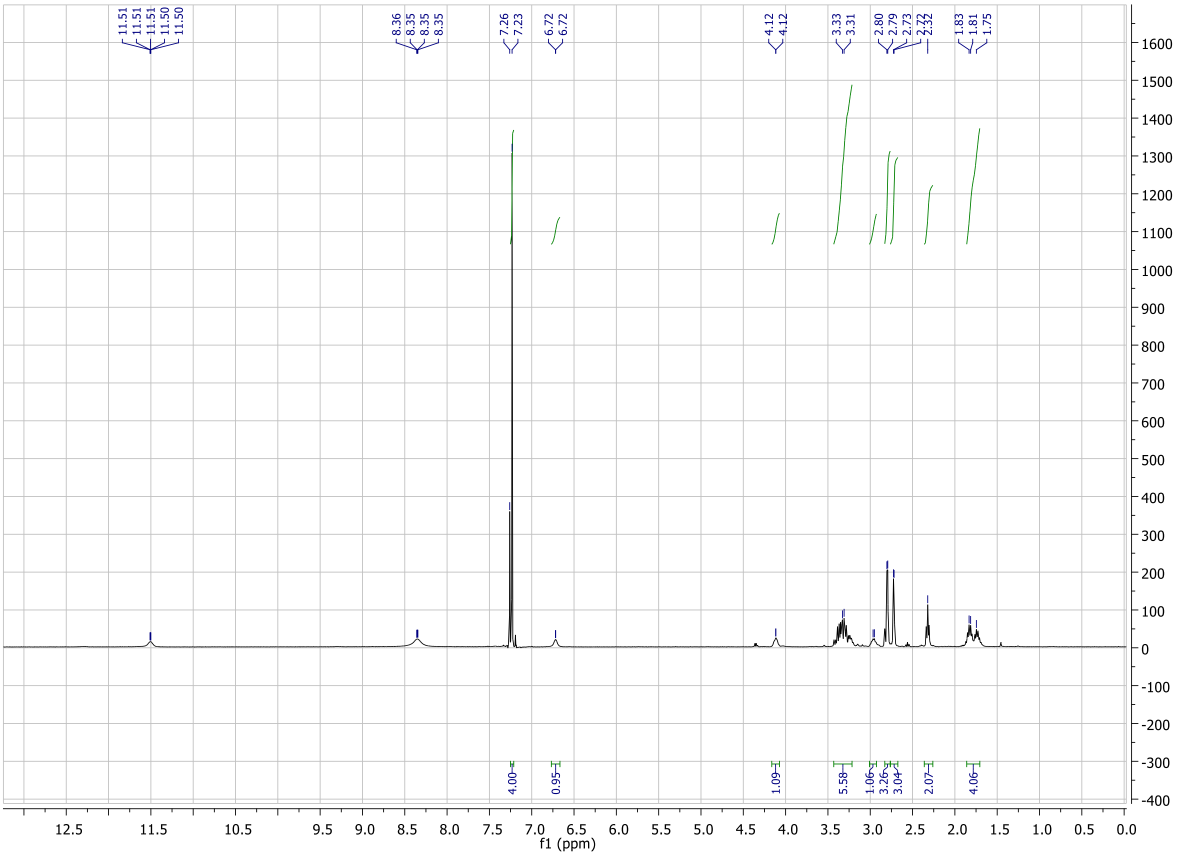

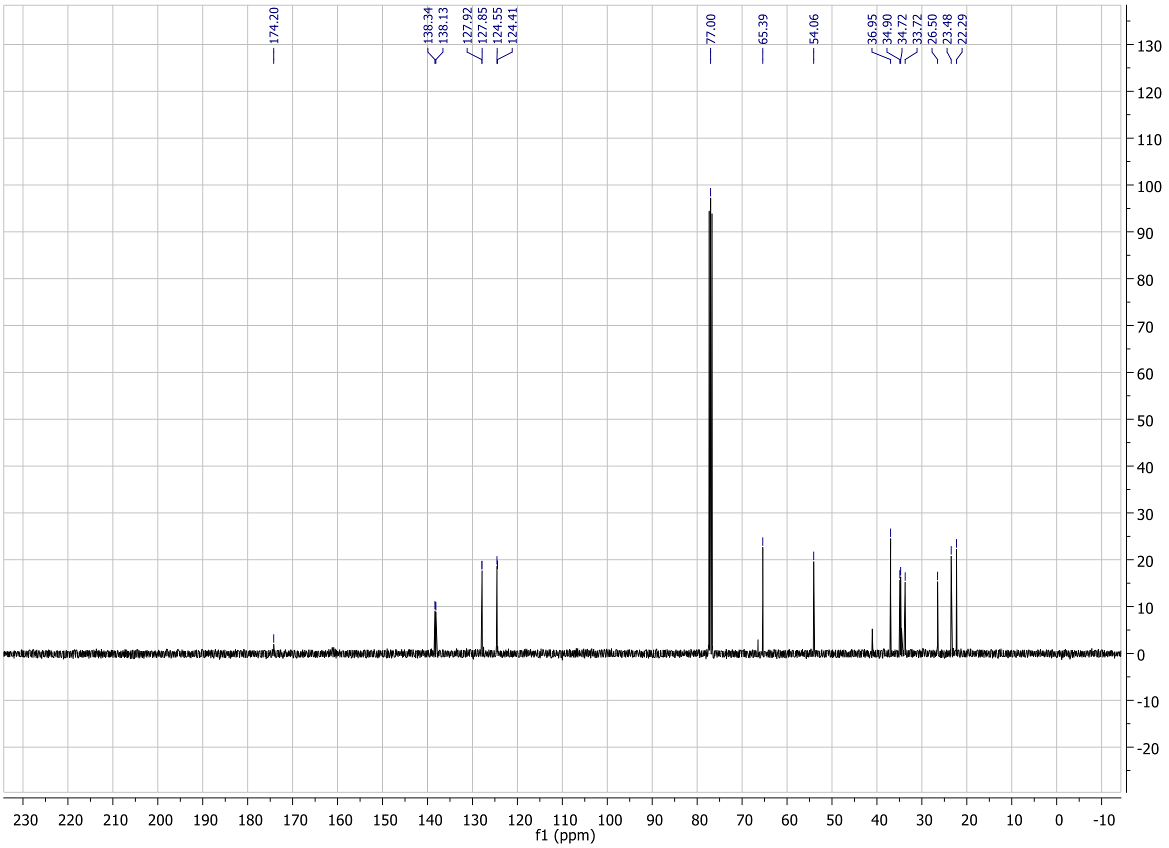

Compound **14**, $CDCl_3/CD_3OD$, 400 MHz

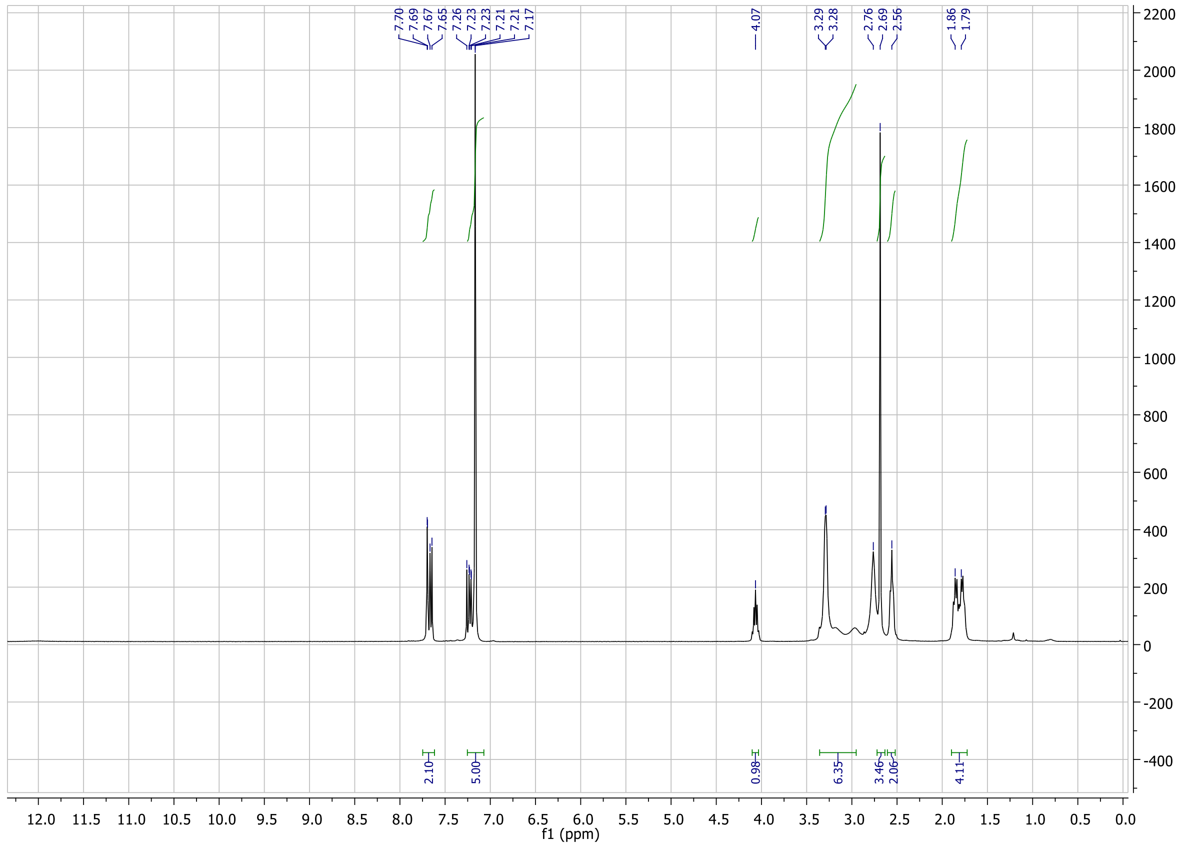

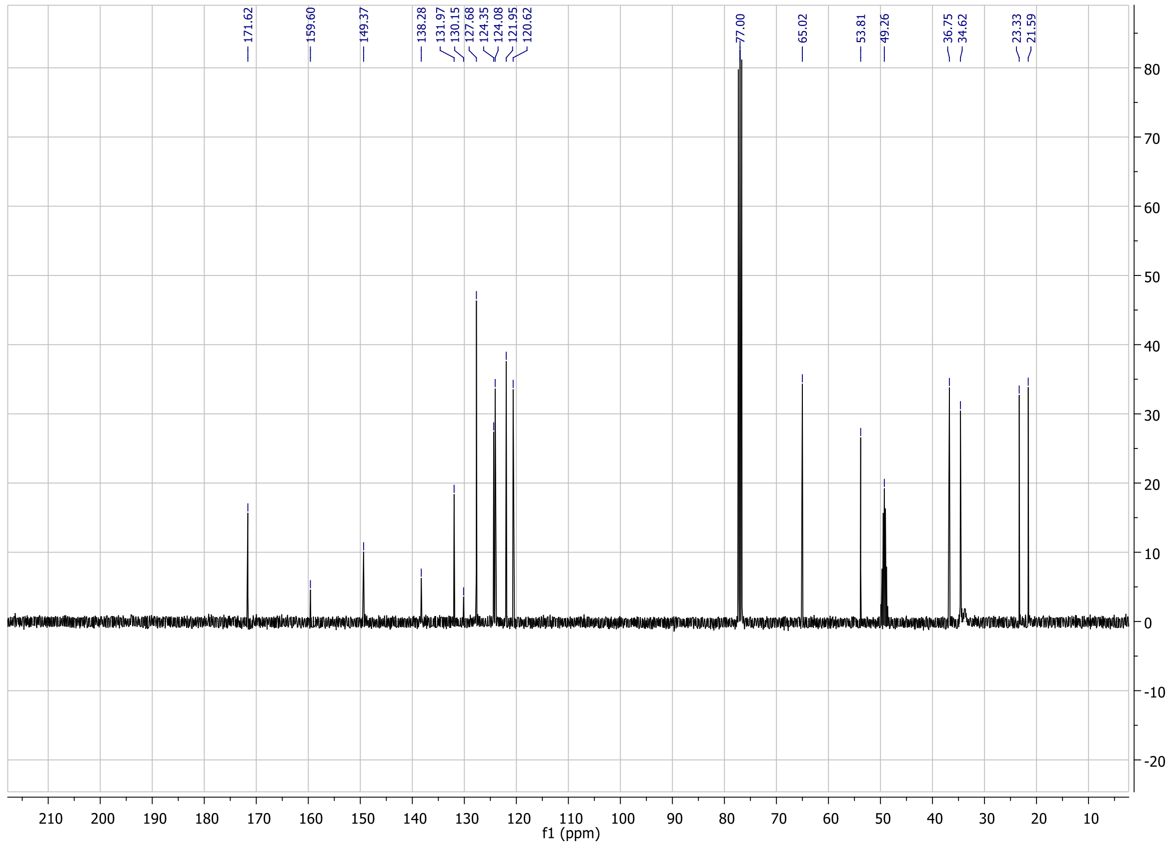

Compound **15**, $CDCl_3/CD_3OD$, 400 MHz

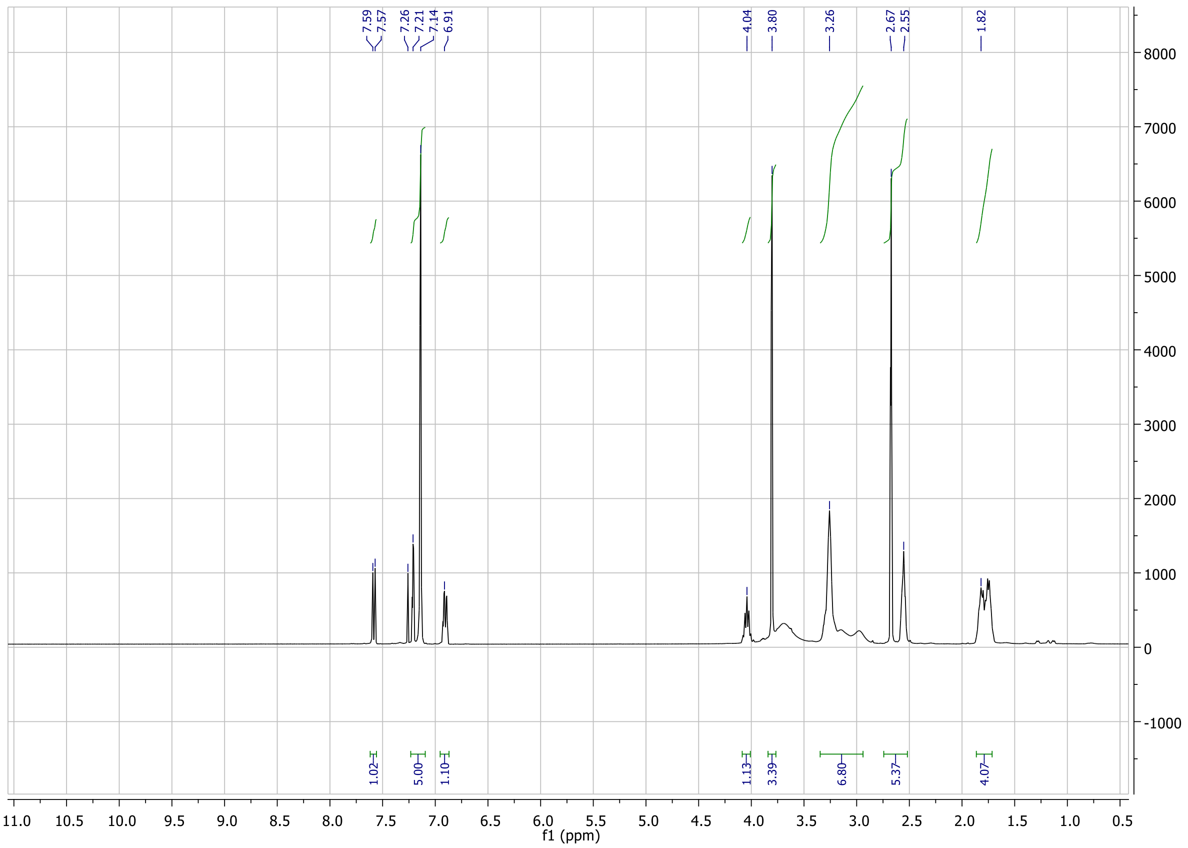

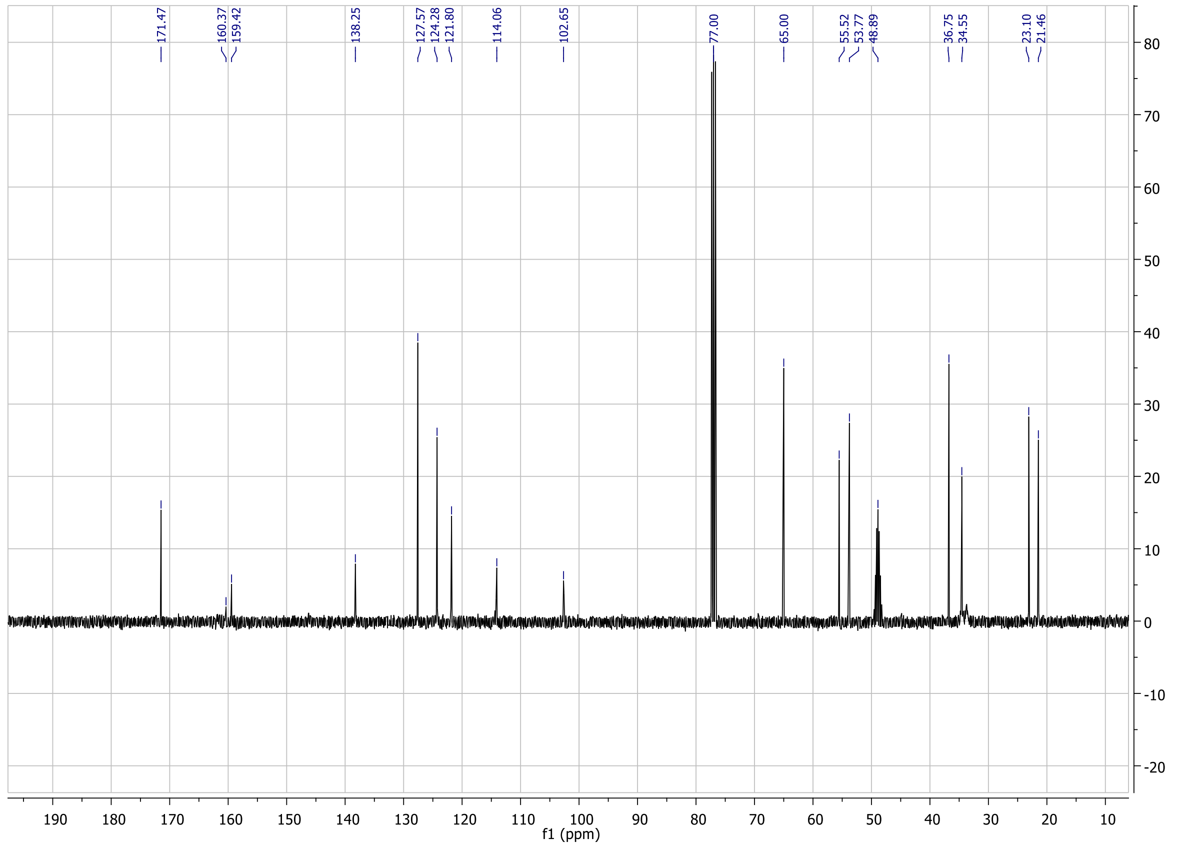

Compound **16**, $CDCl_3$, 400 MHz

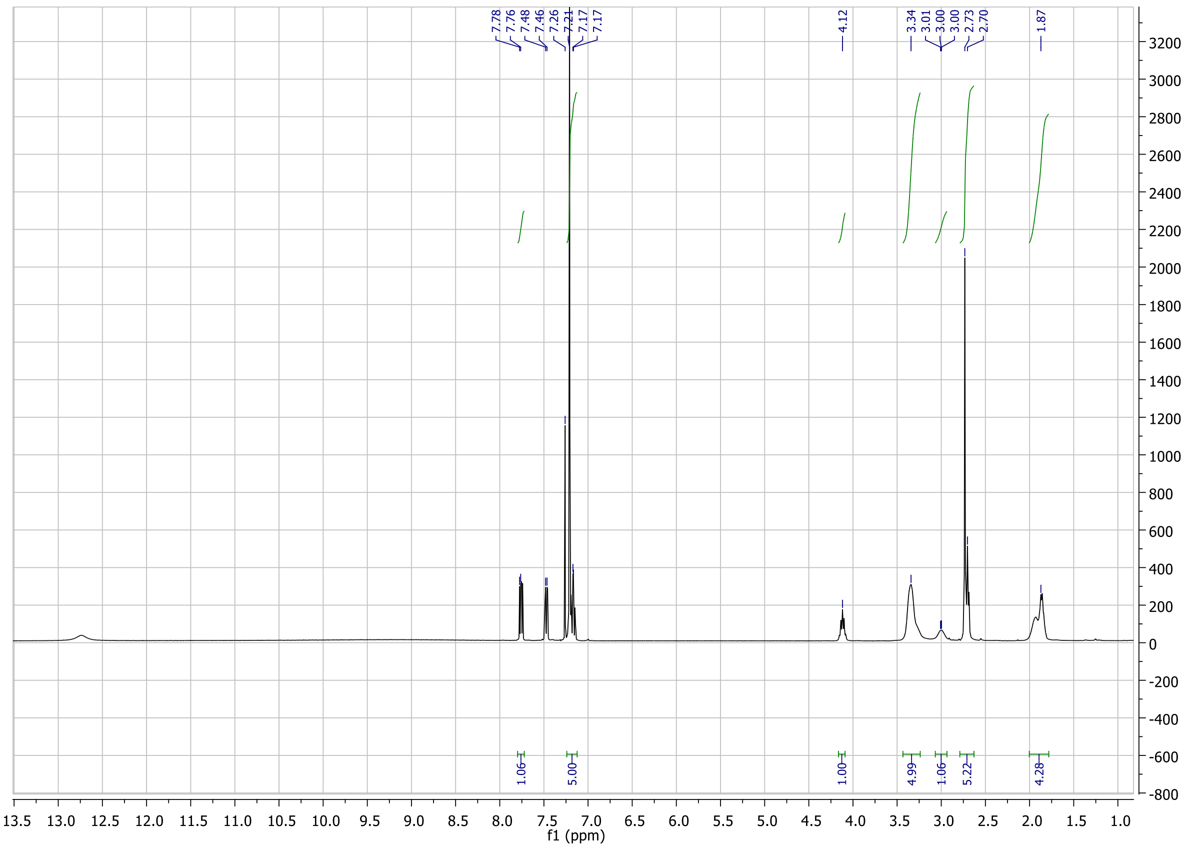

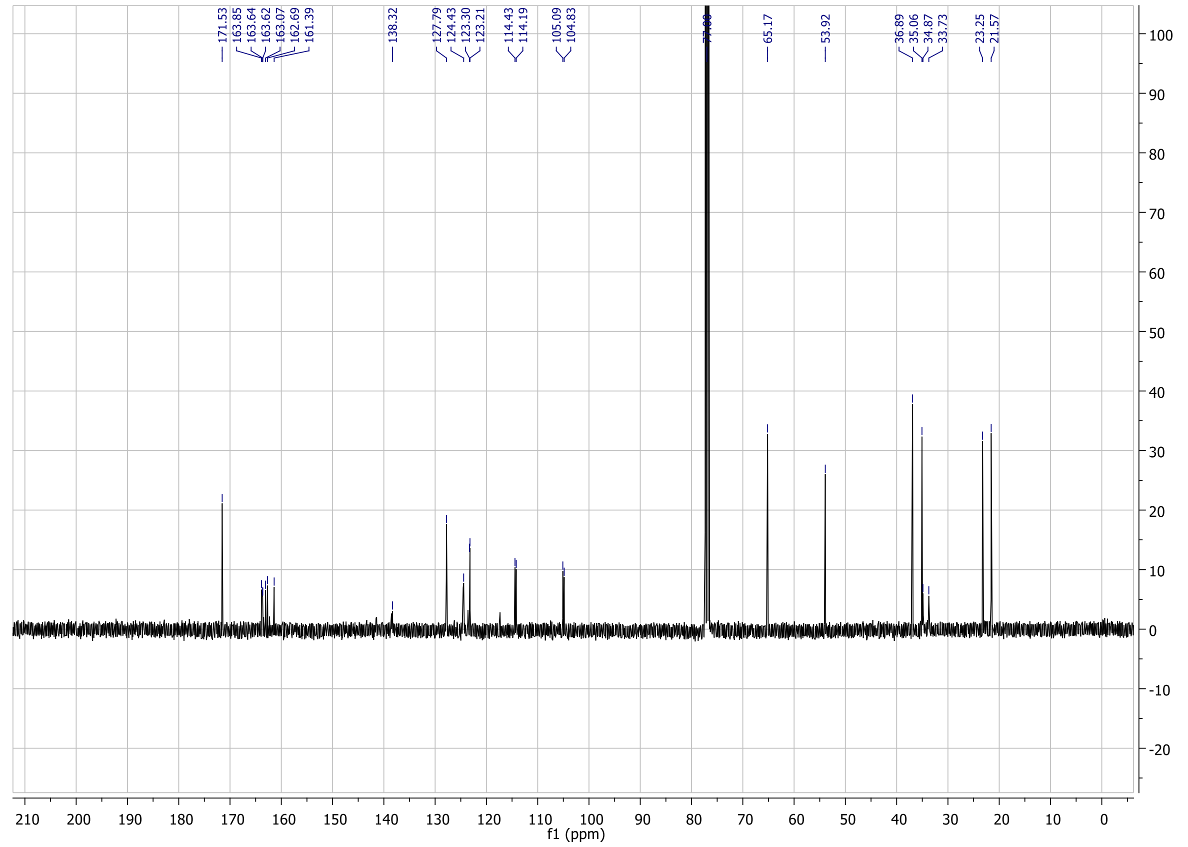

Compound **17**, $CDCl_3$, 400 MHz

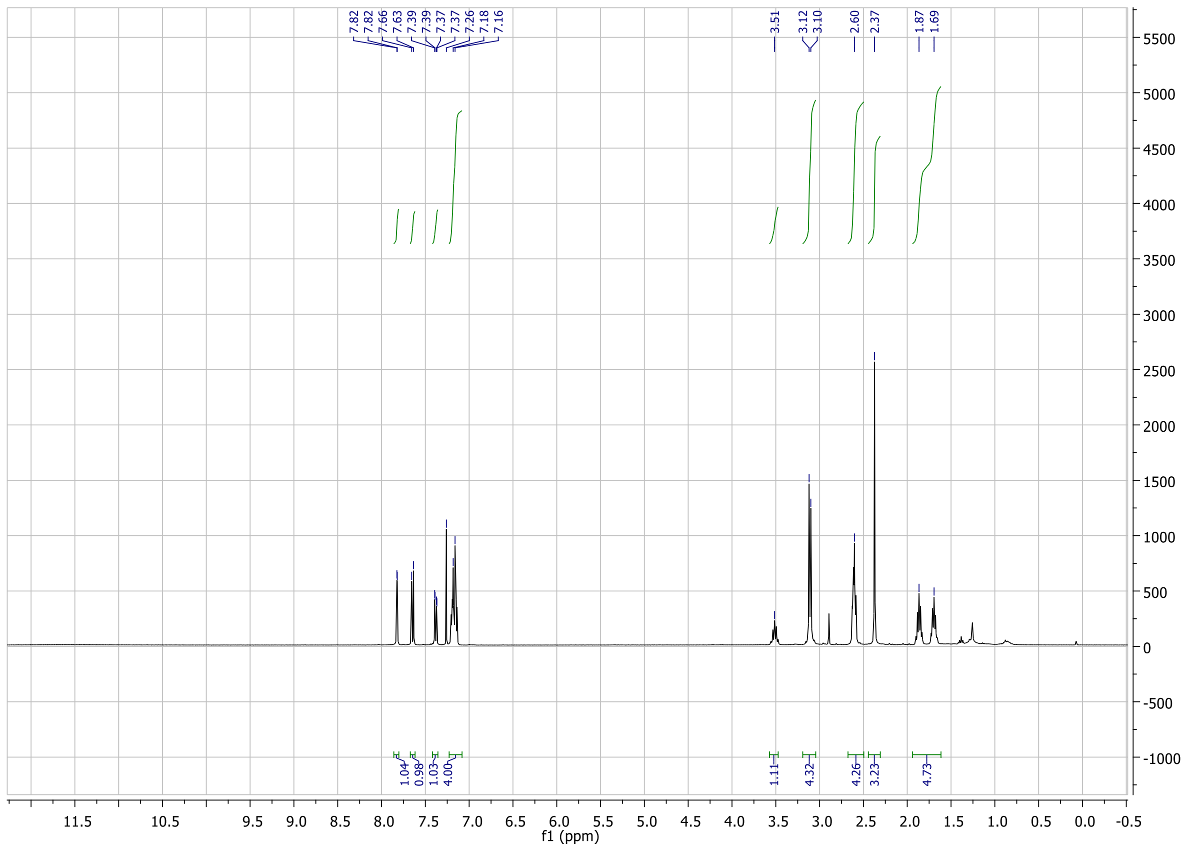

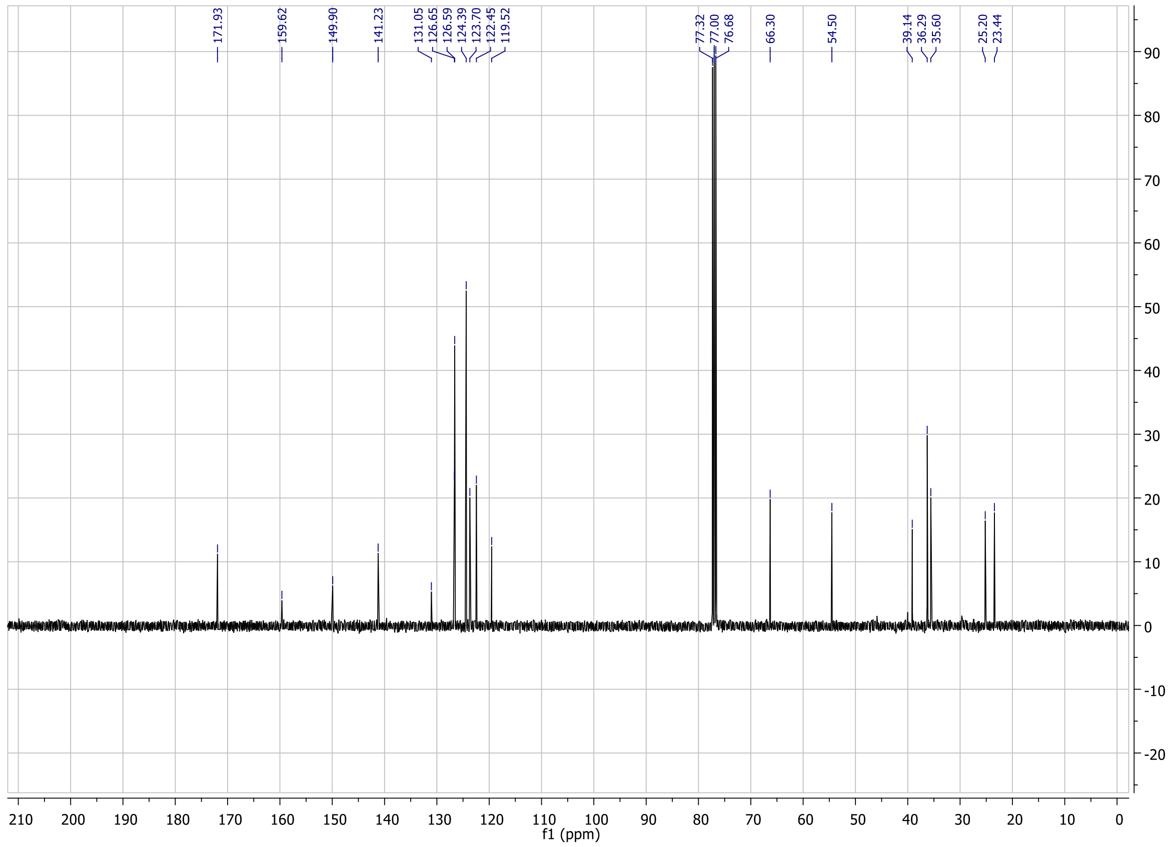

Compound **18**, $CDCl_3$, 400 MHz

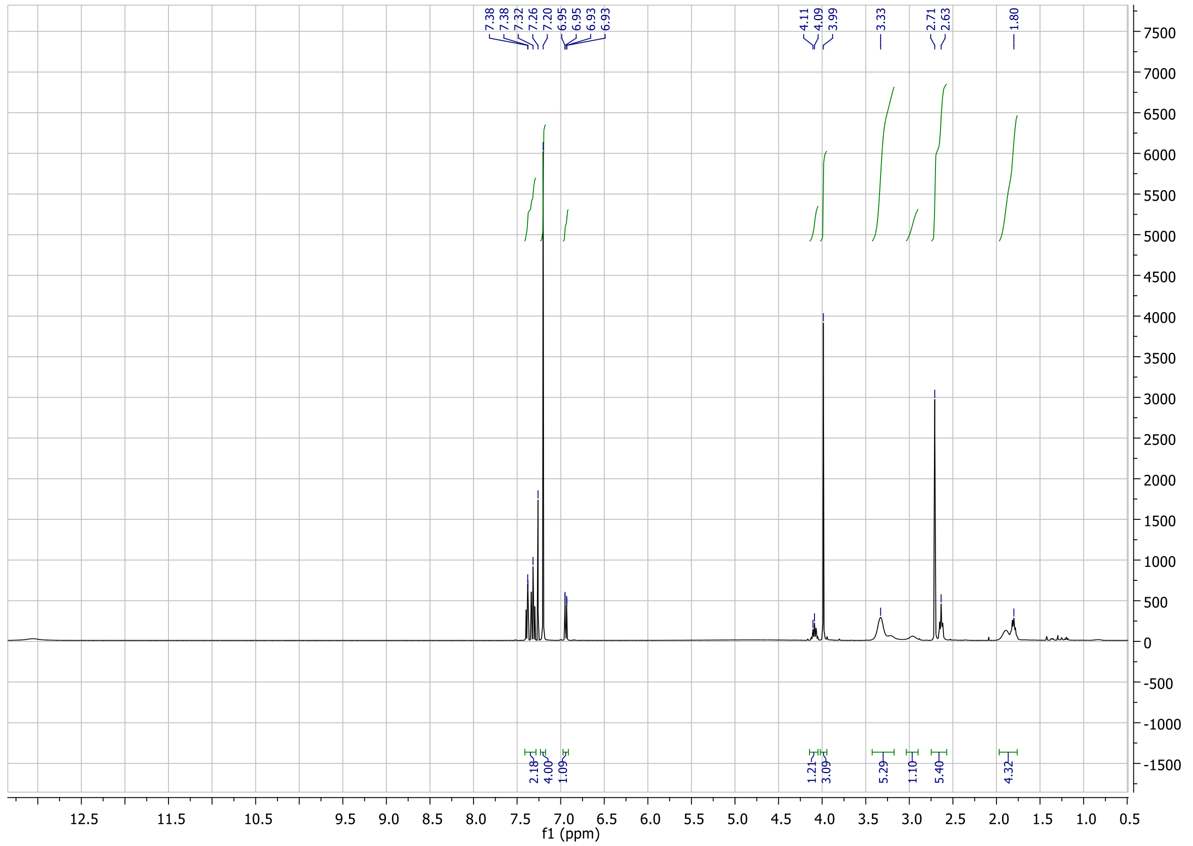

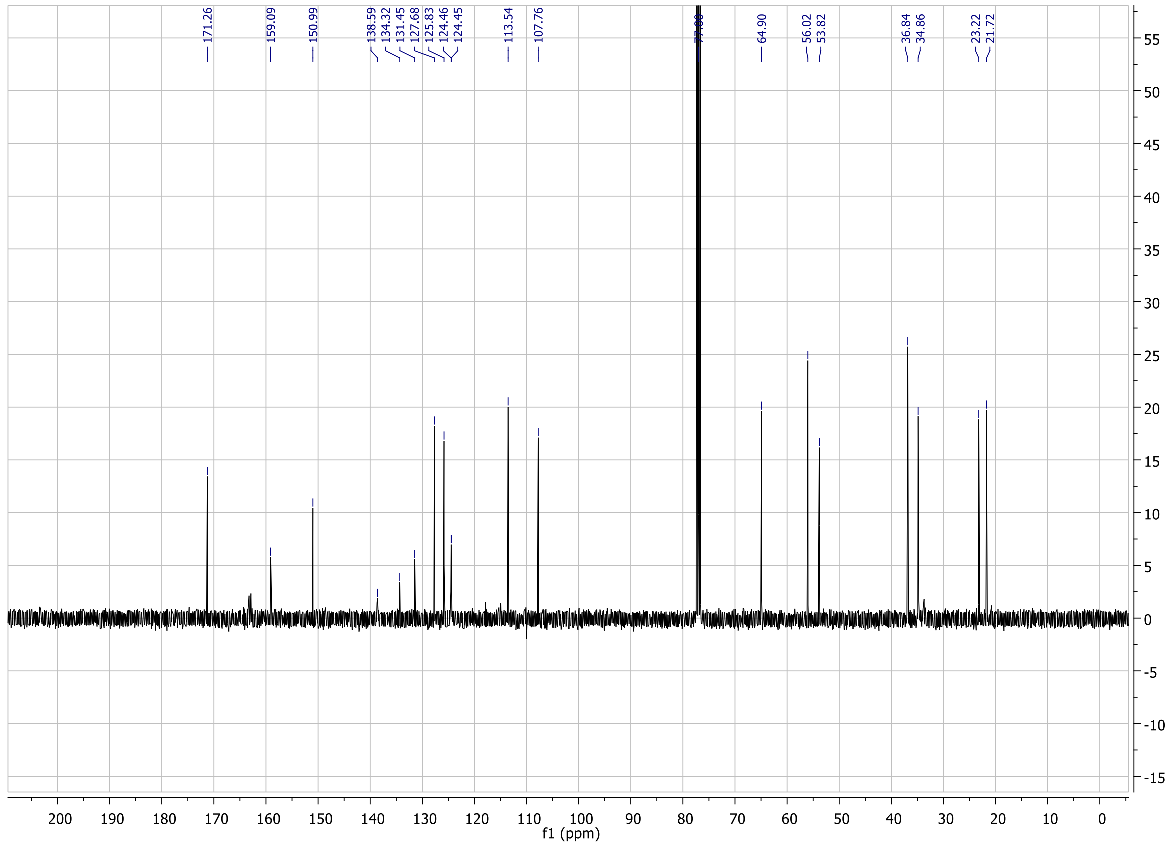

Compound **19**, CD_3OD, 400 MHz

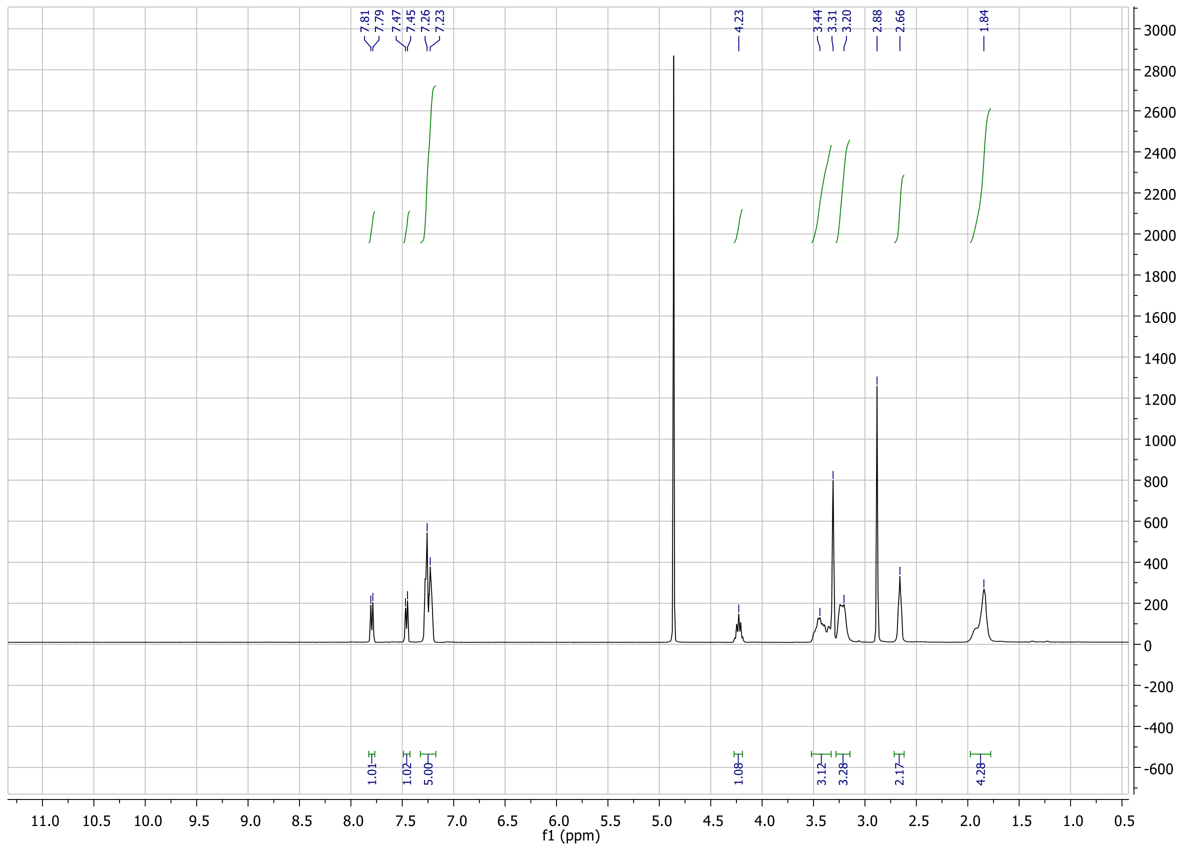

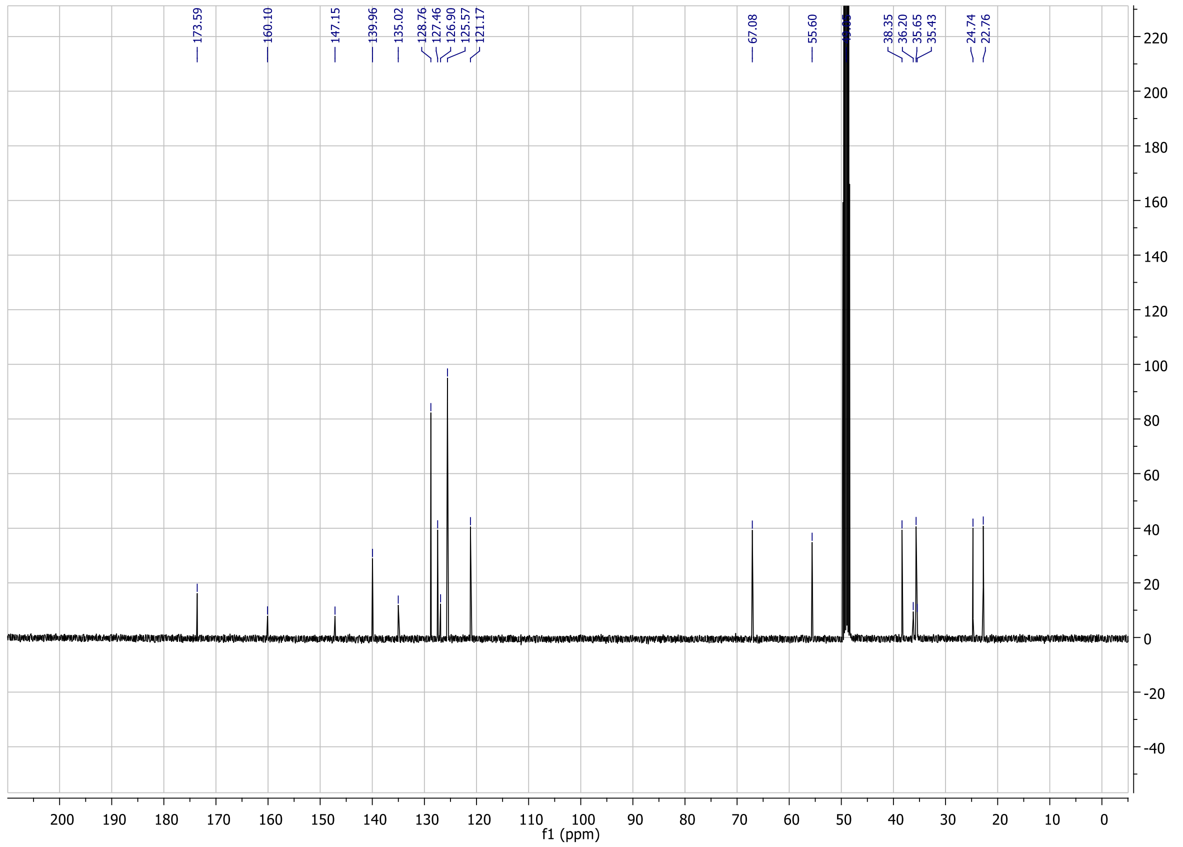

Compound **20**, CD_3OD, 400 MHz

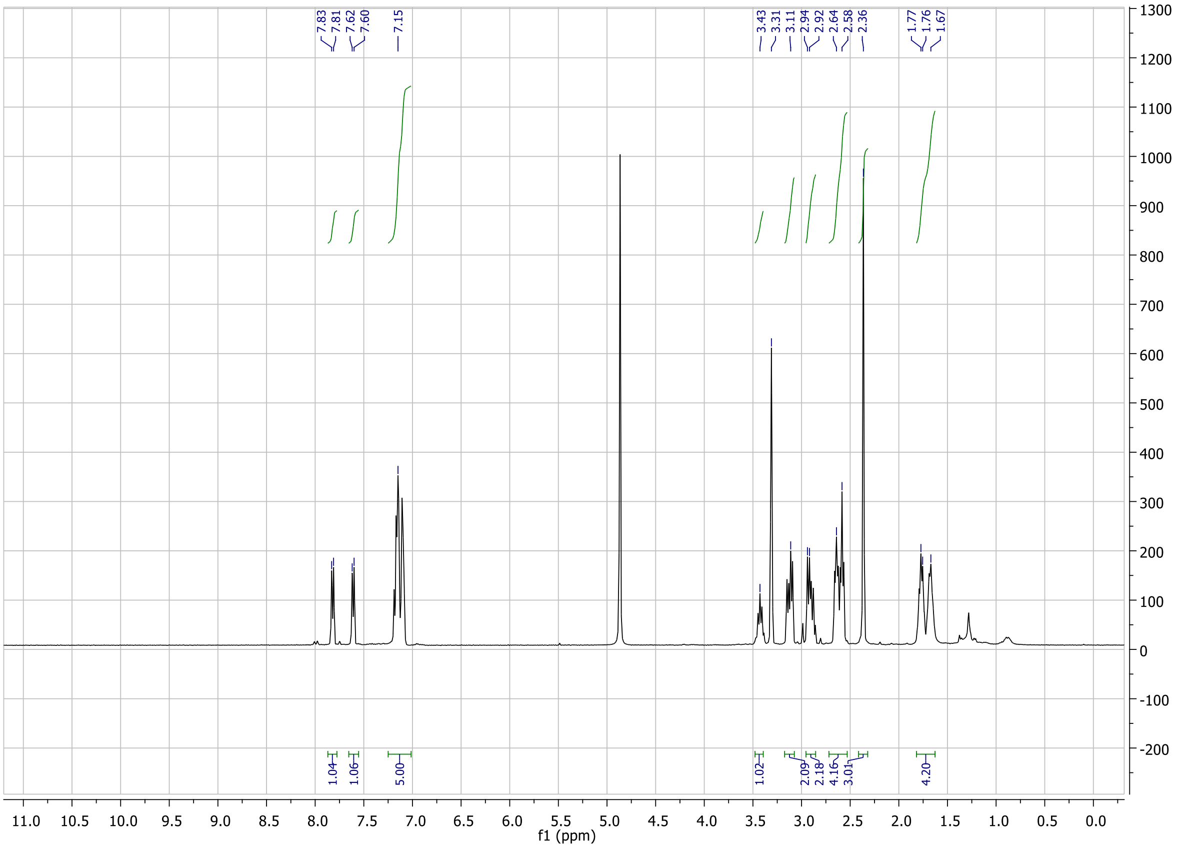

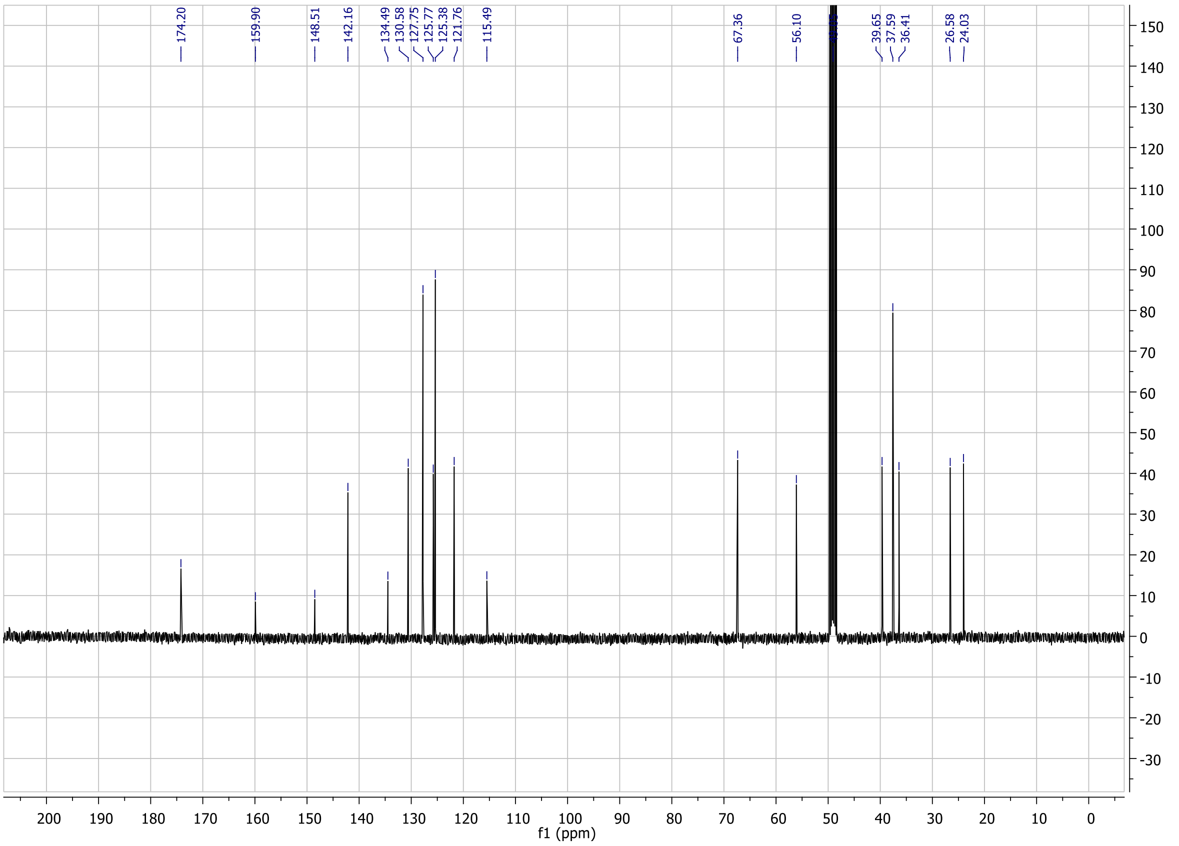

Compound **21**, CD_3OD, 500 MHz

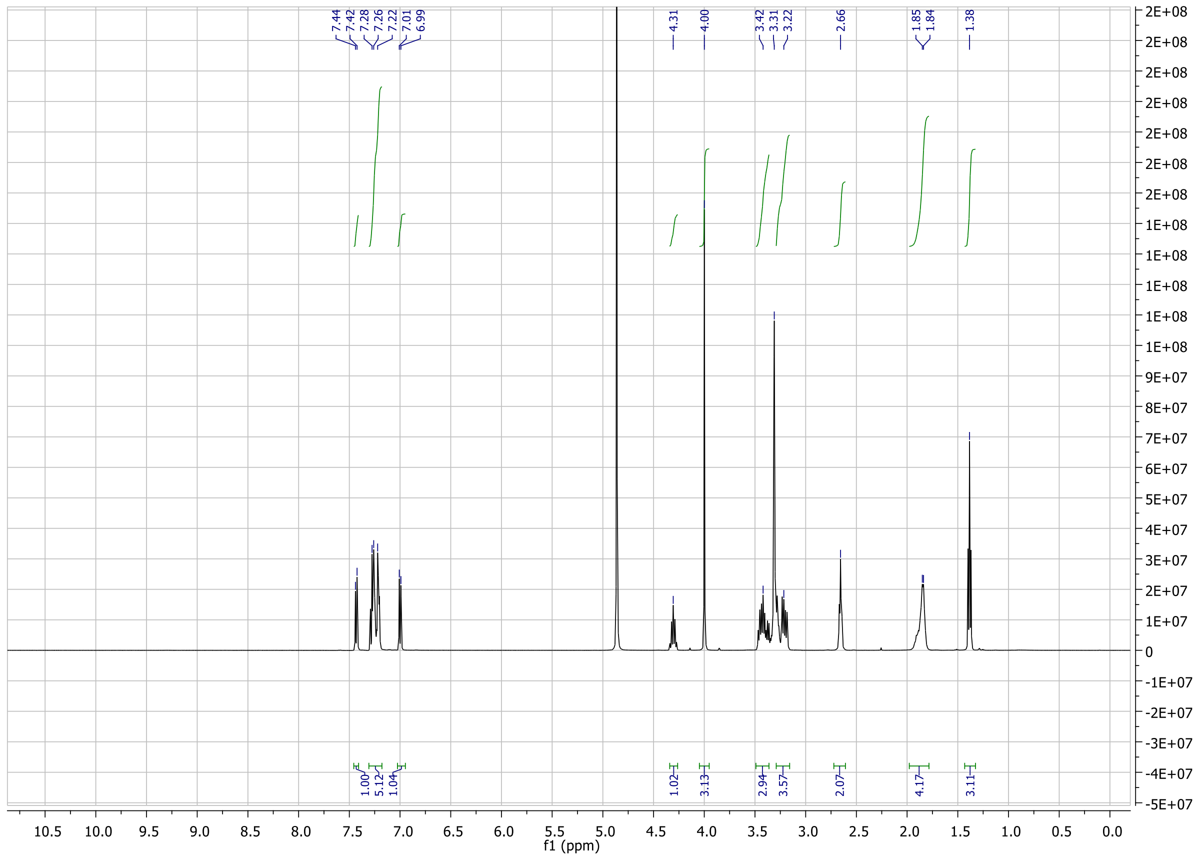

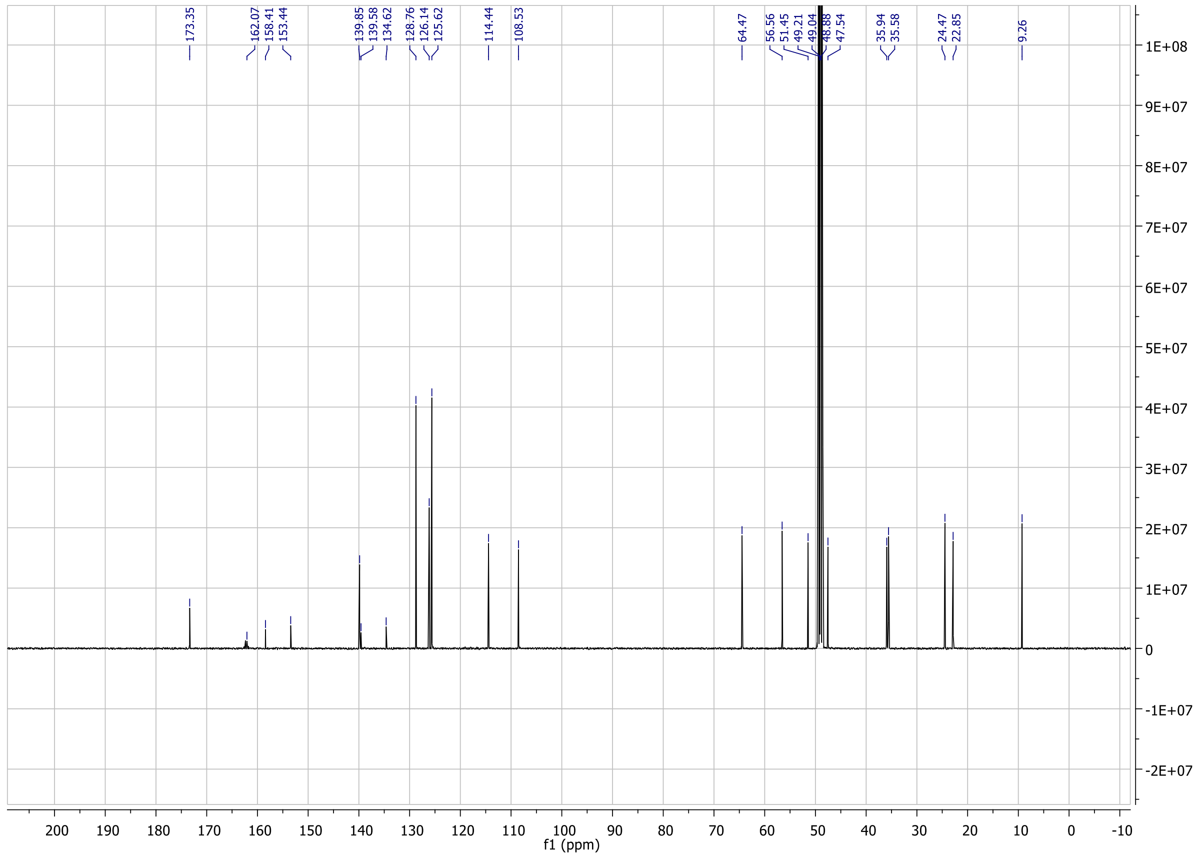

Compound **22**, CD_3OD, 500 MHz

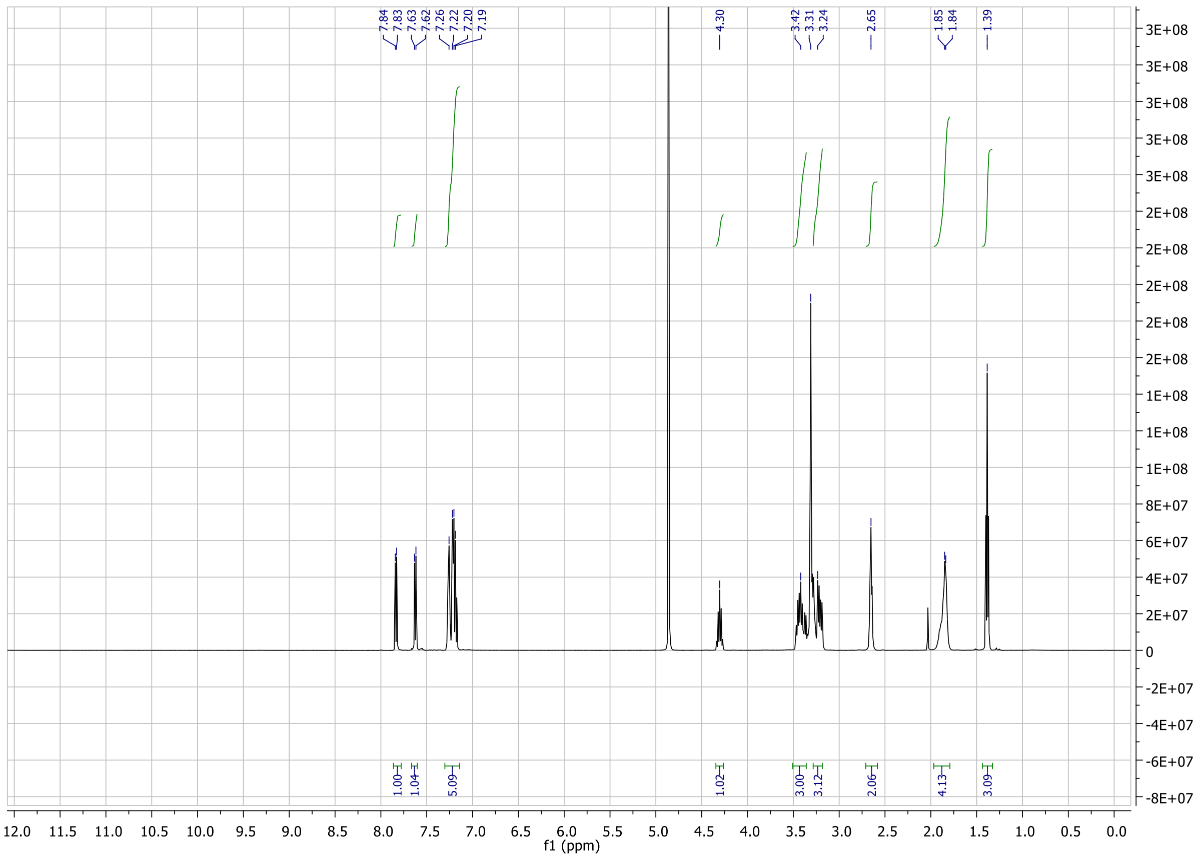

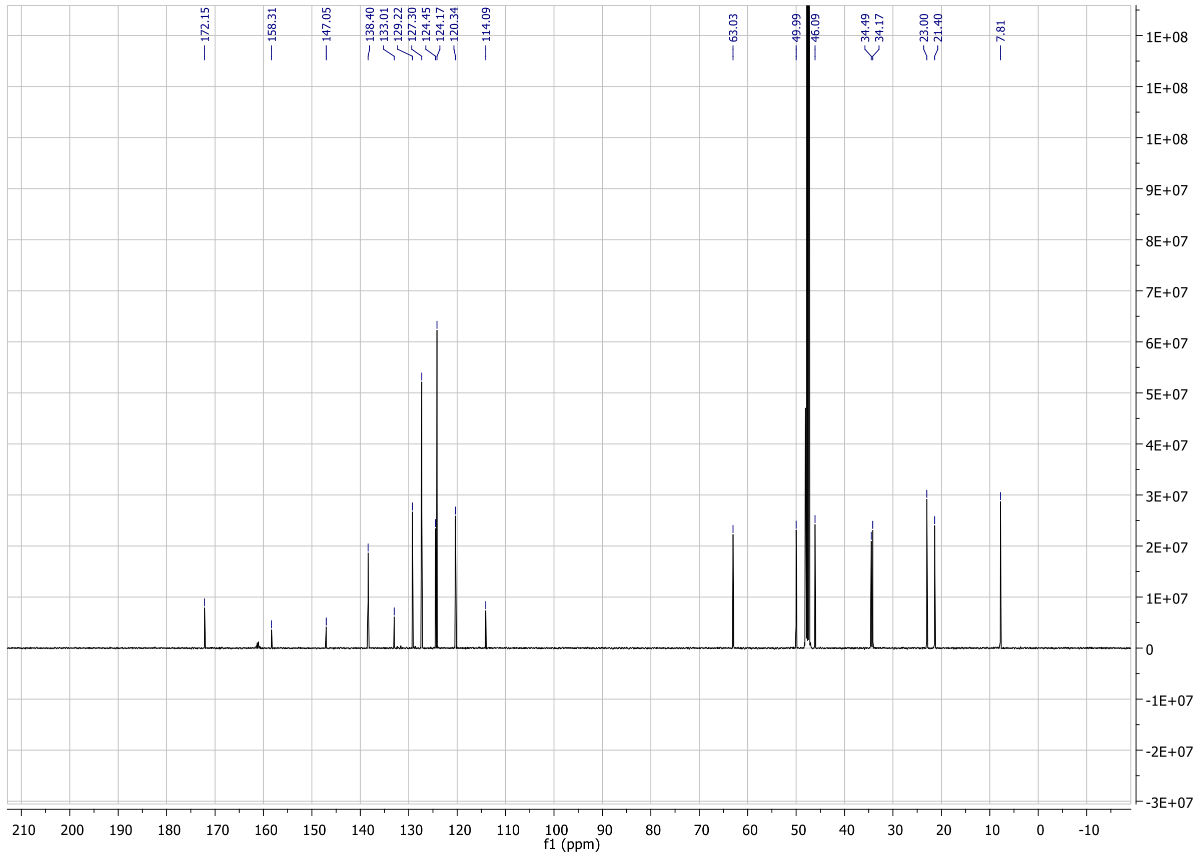

Compound **23**, CD_3OD, 400 MHz

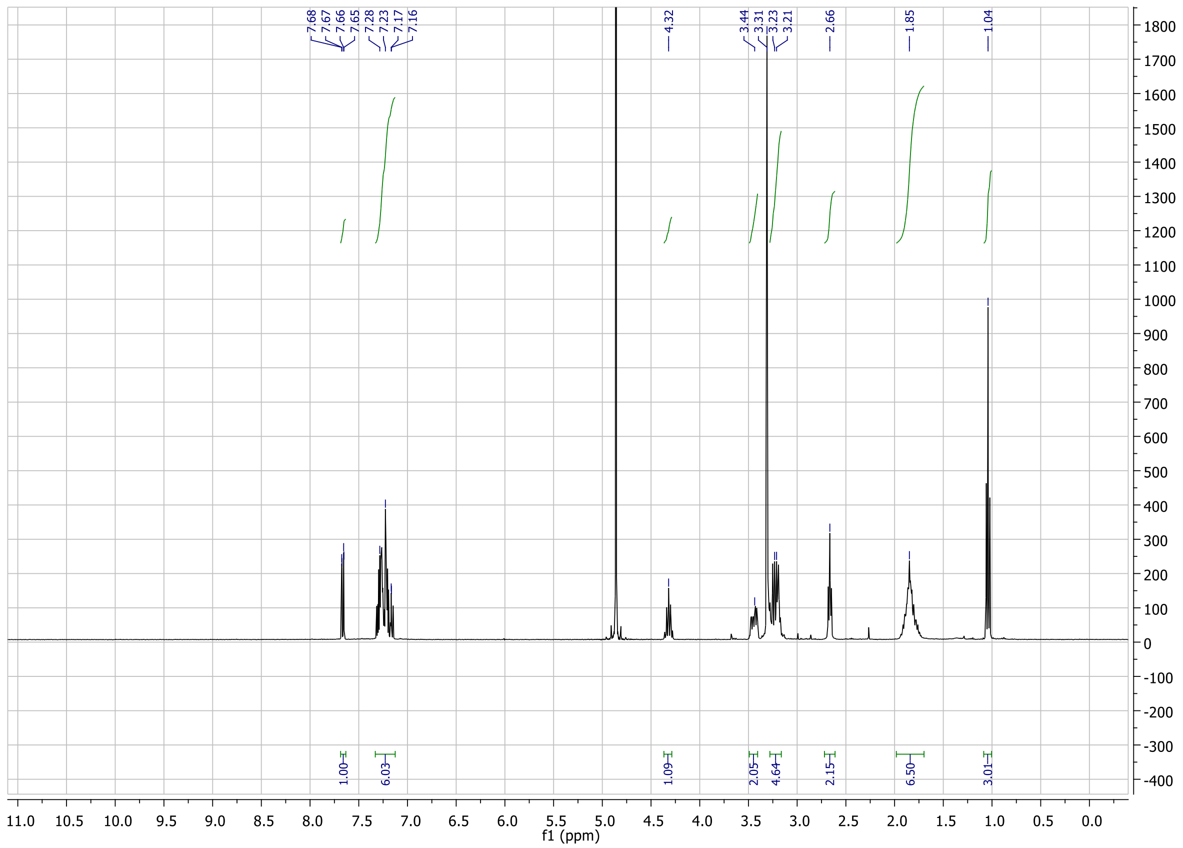

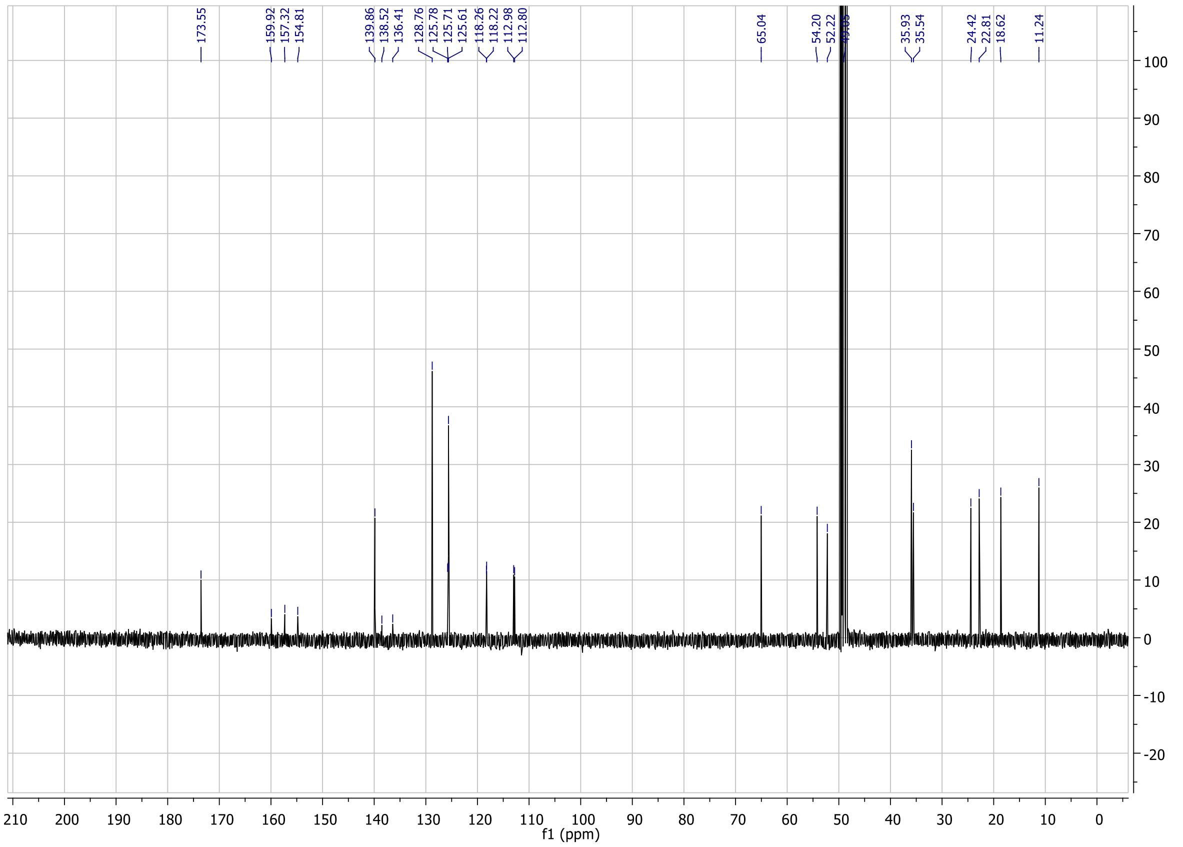

Compound **24**, CD_3OD, 400 MHz

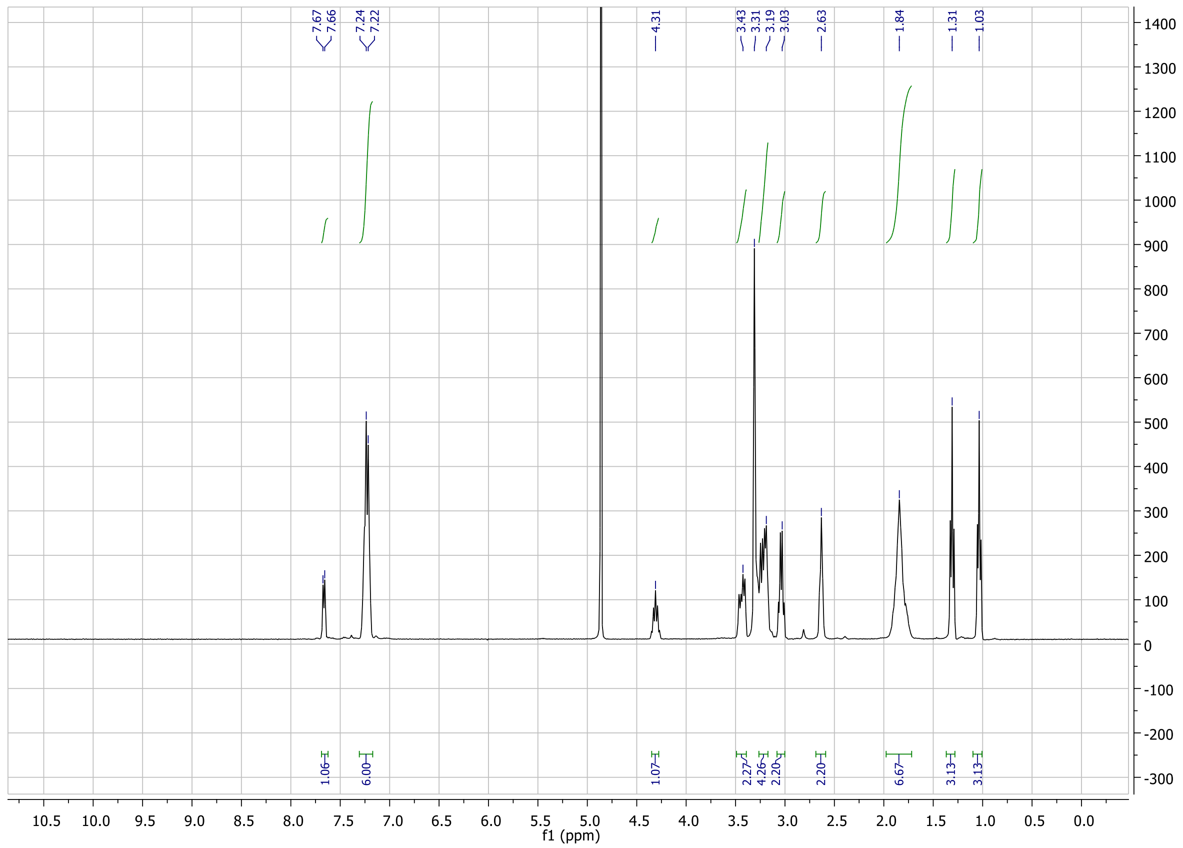

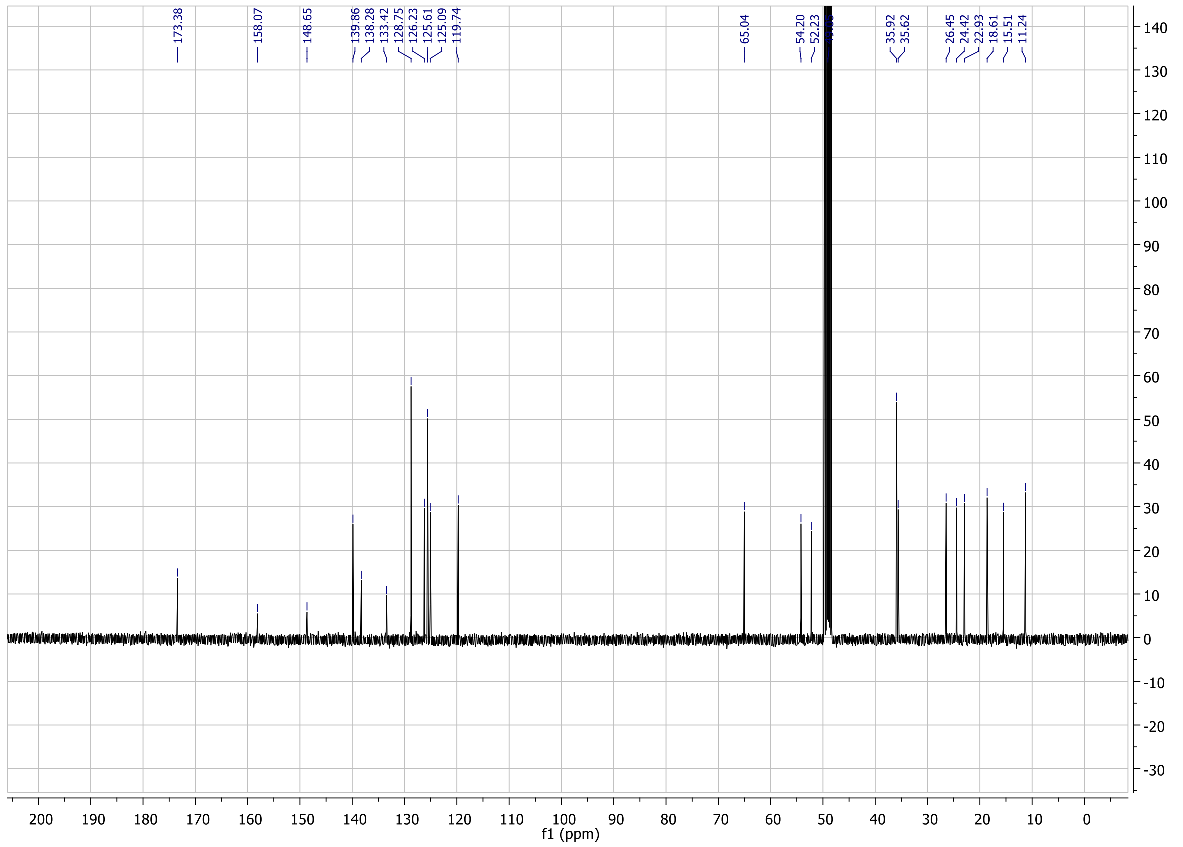

Compound **25**, $CDCl_3$, 400 MHz

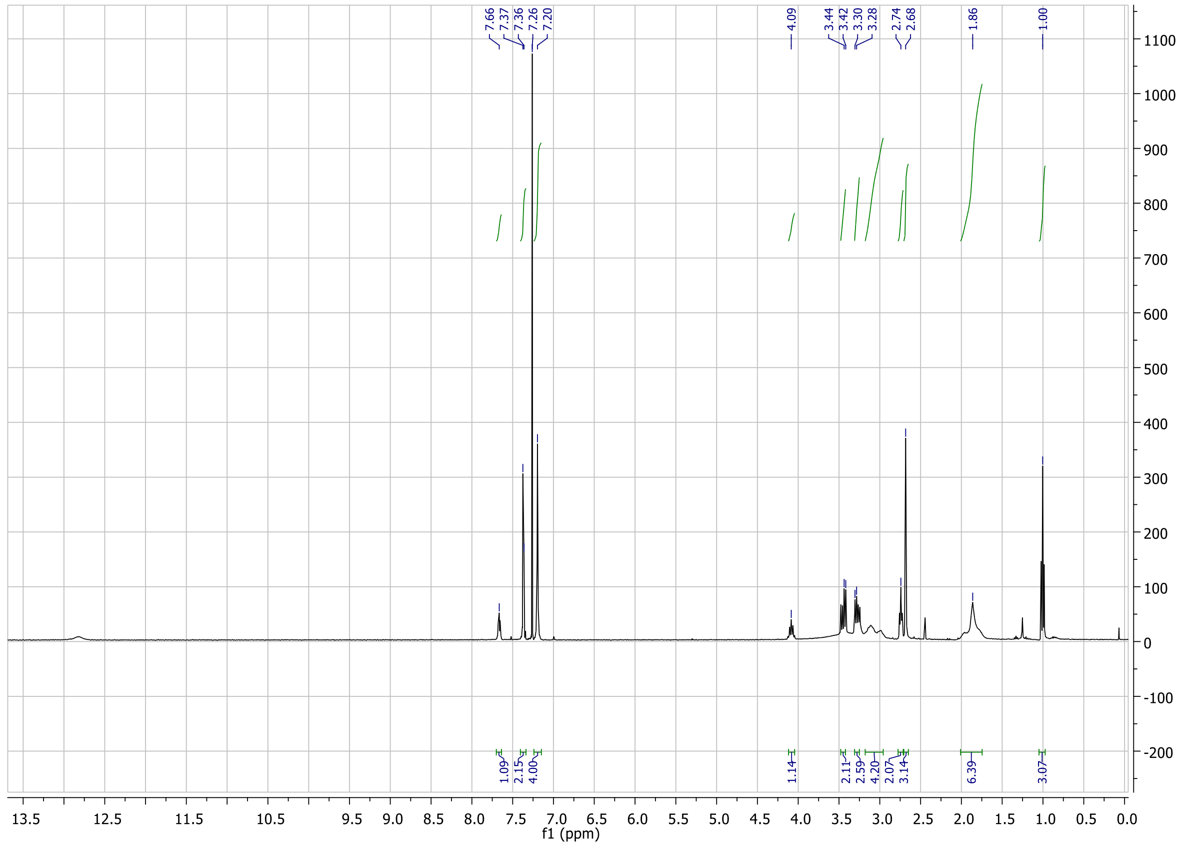

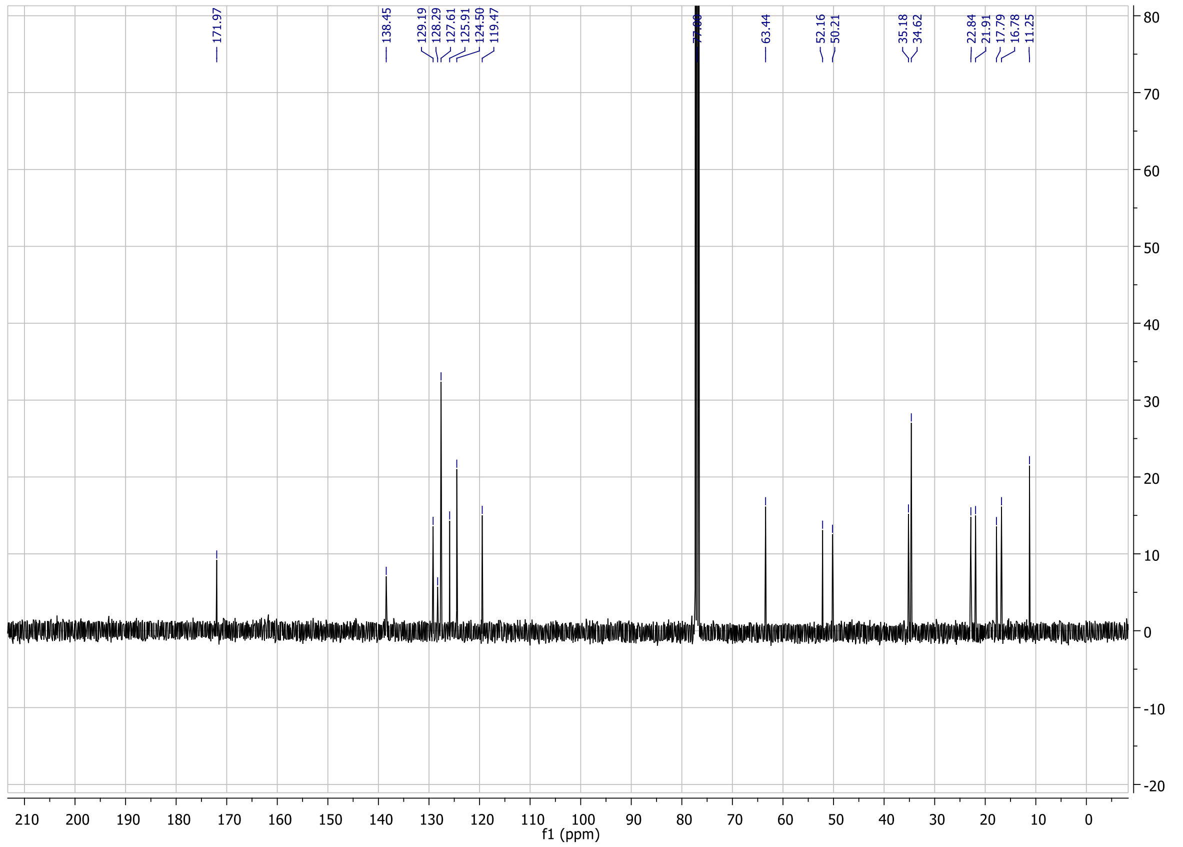

Compound **26**, CD_3OD, 400 MHz

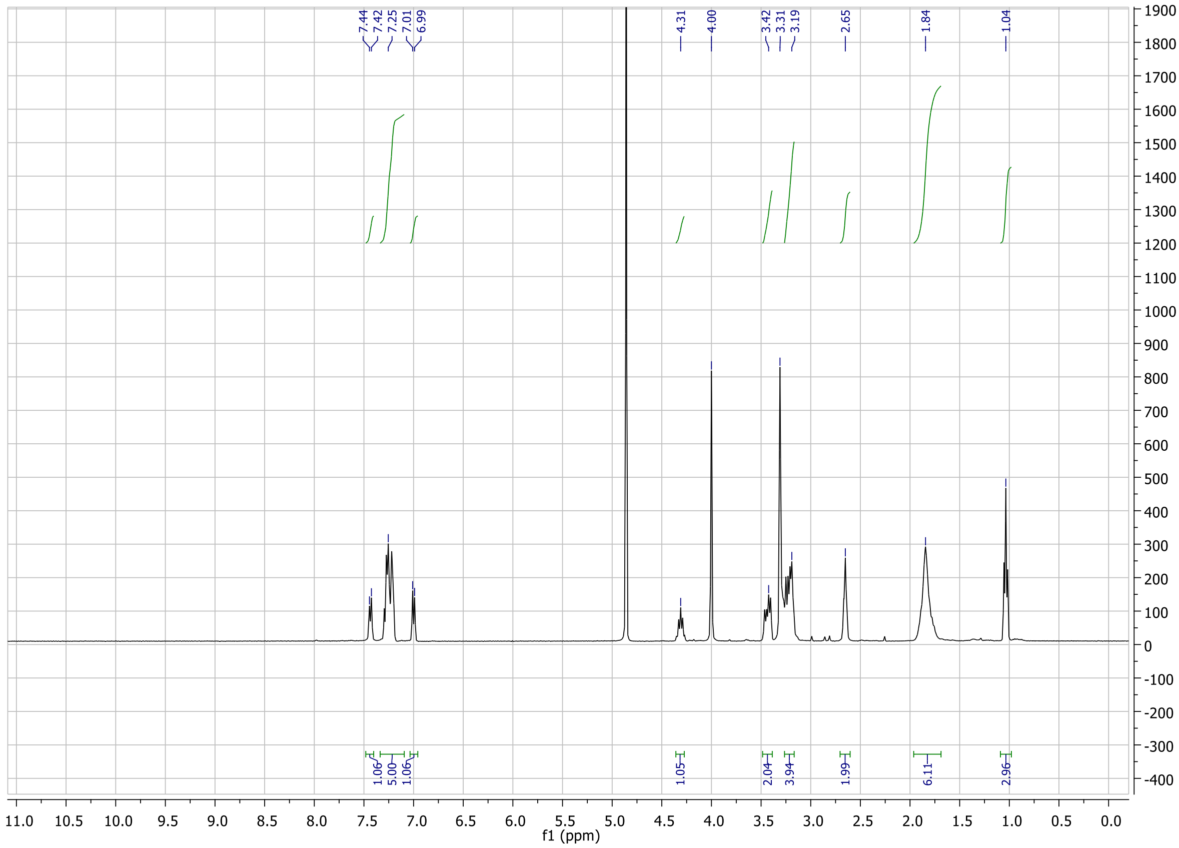

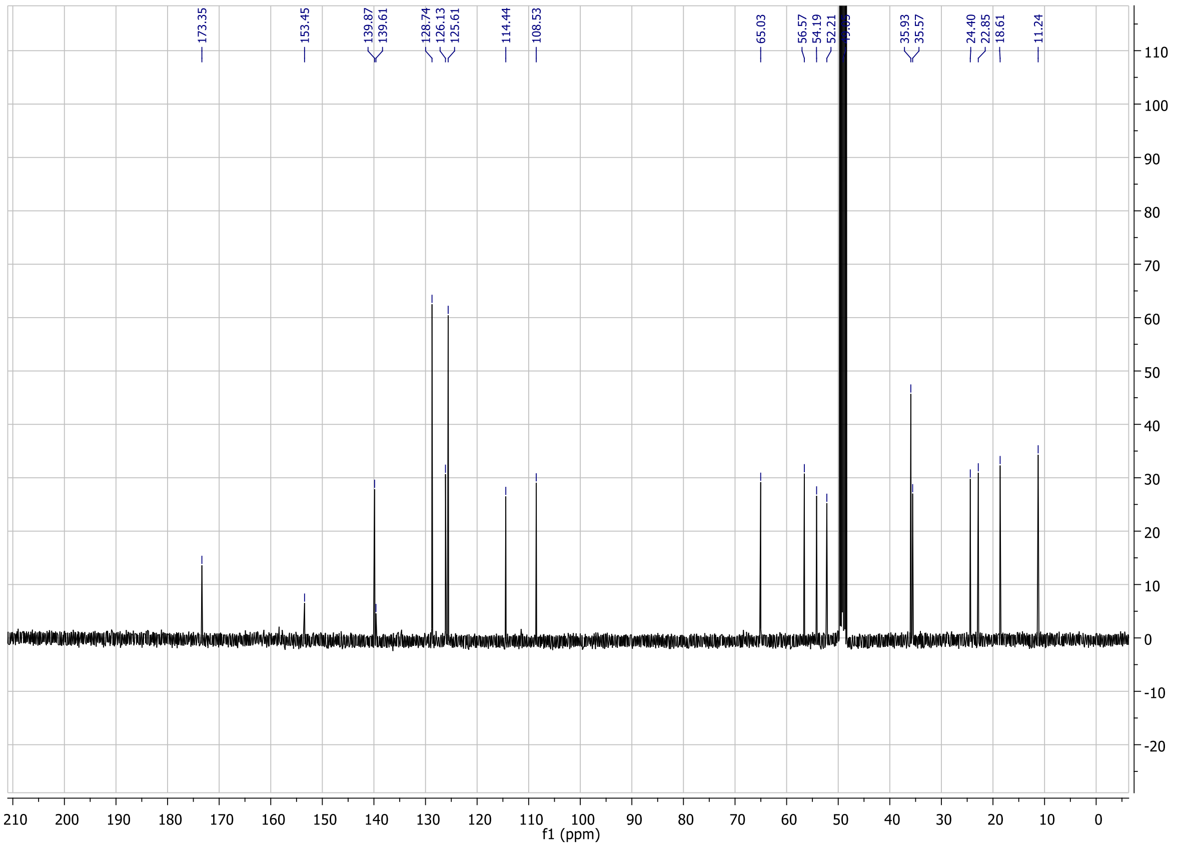

Compound **27**, CD_3OD, 400 MHz

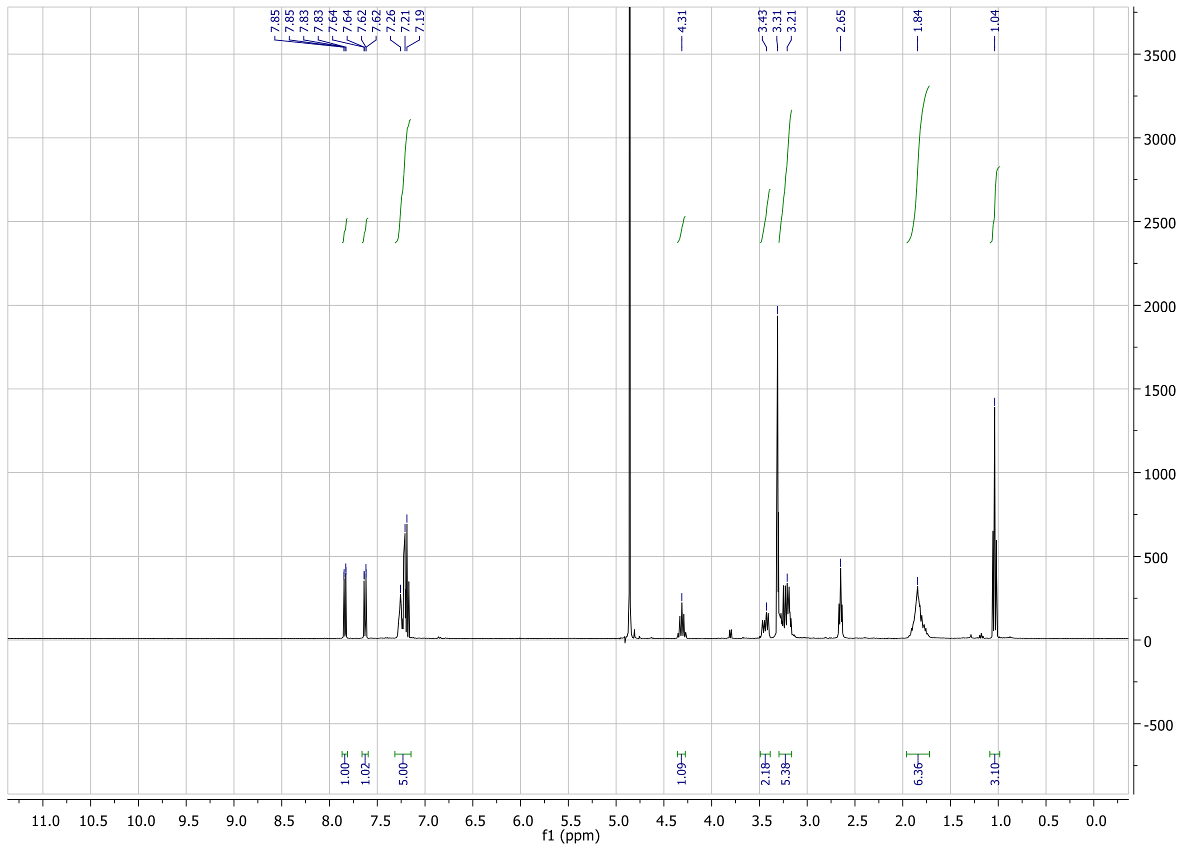

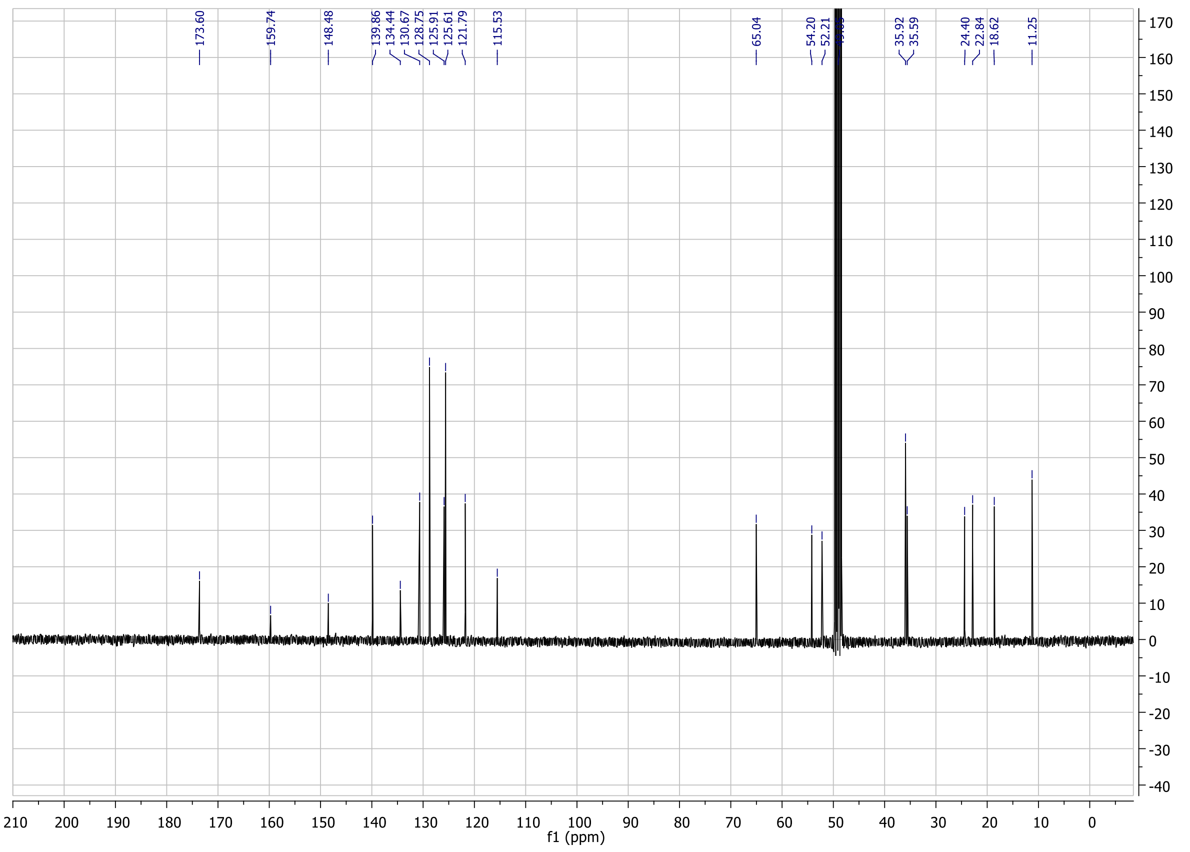

Compound **28**, CD_3OD, 500 MHz

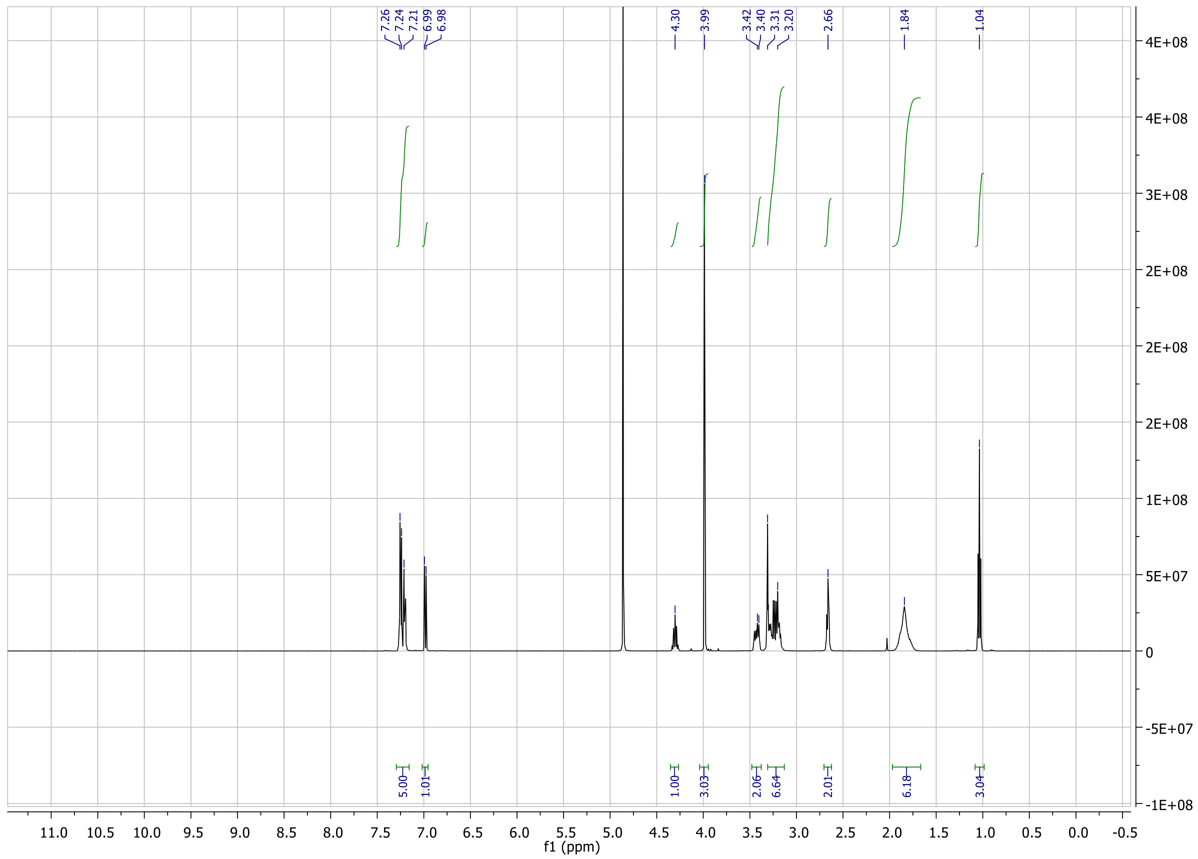

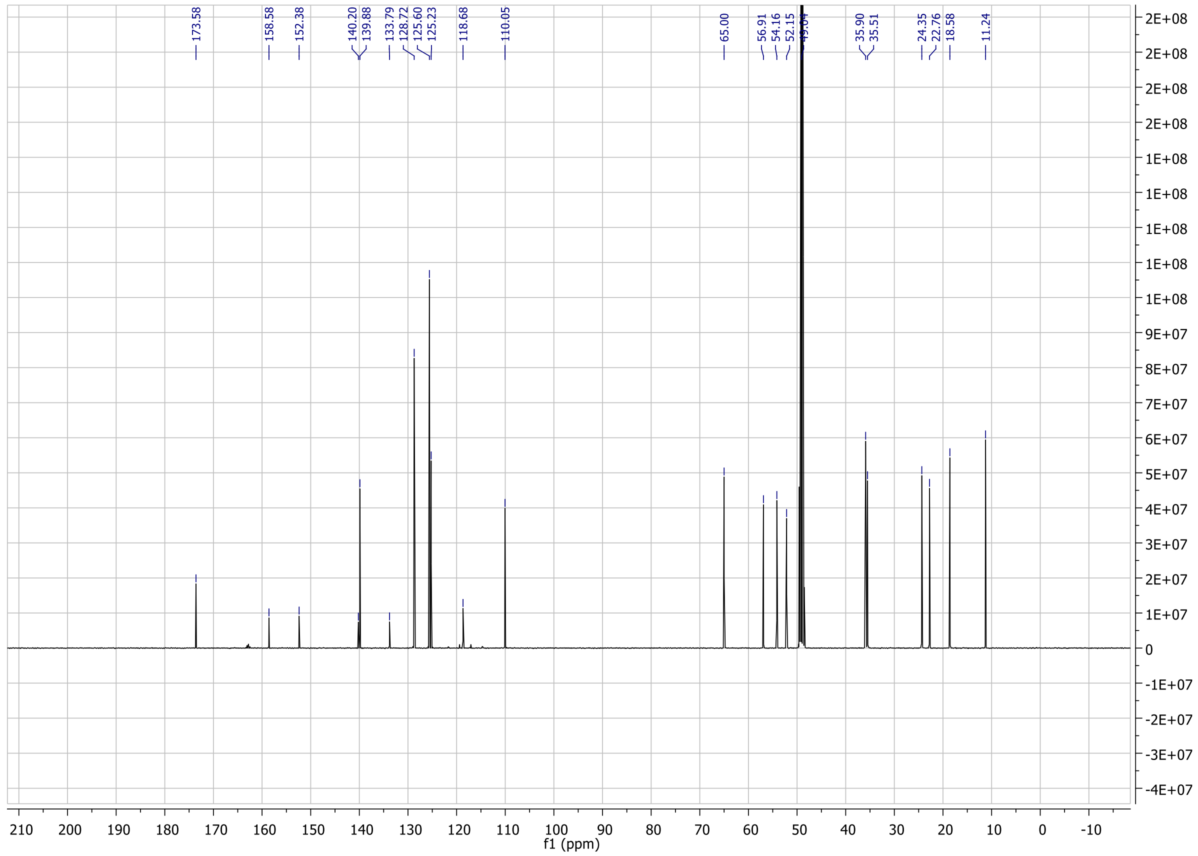

Compound **29**, CD_3OD, 500 MHz

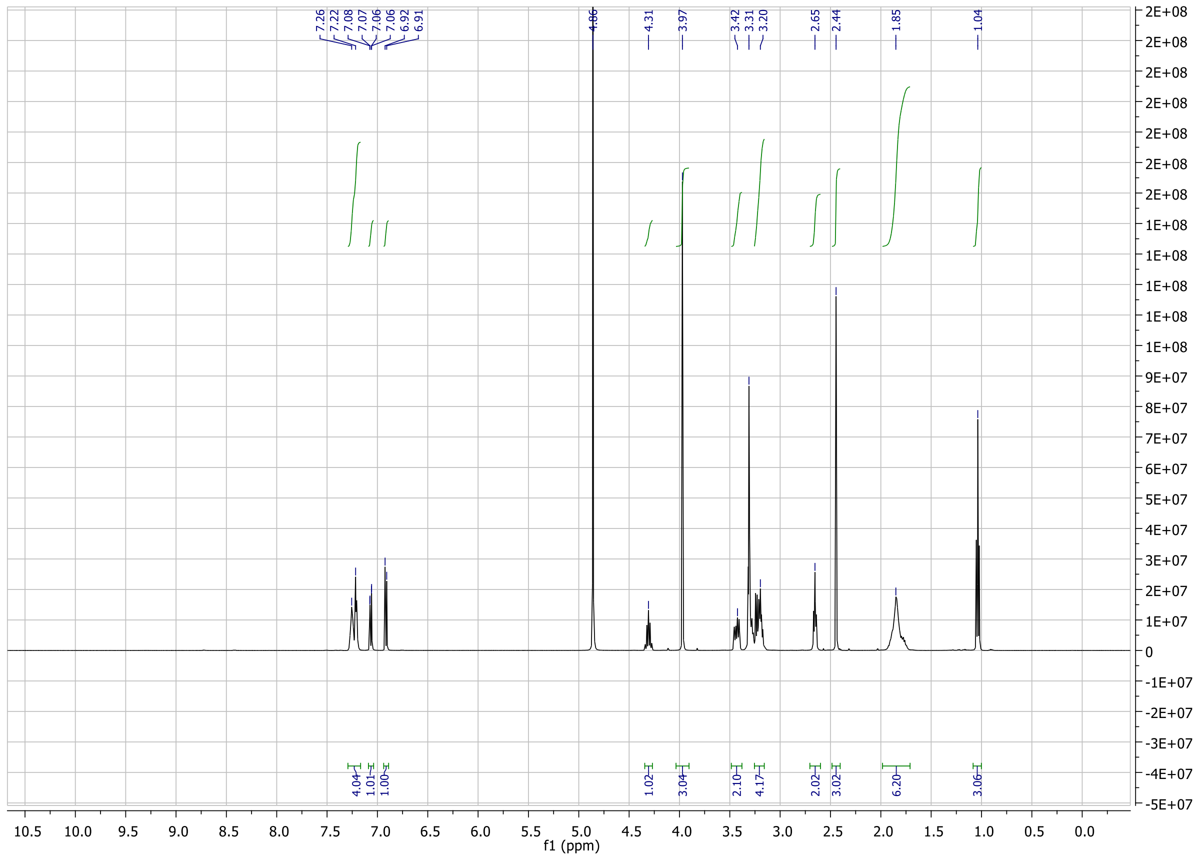

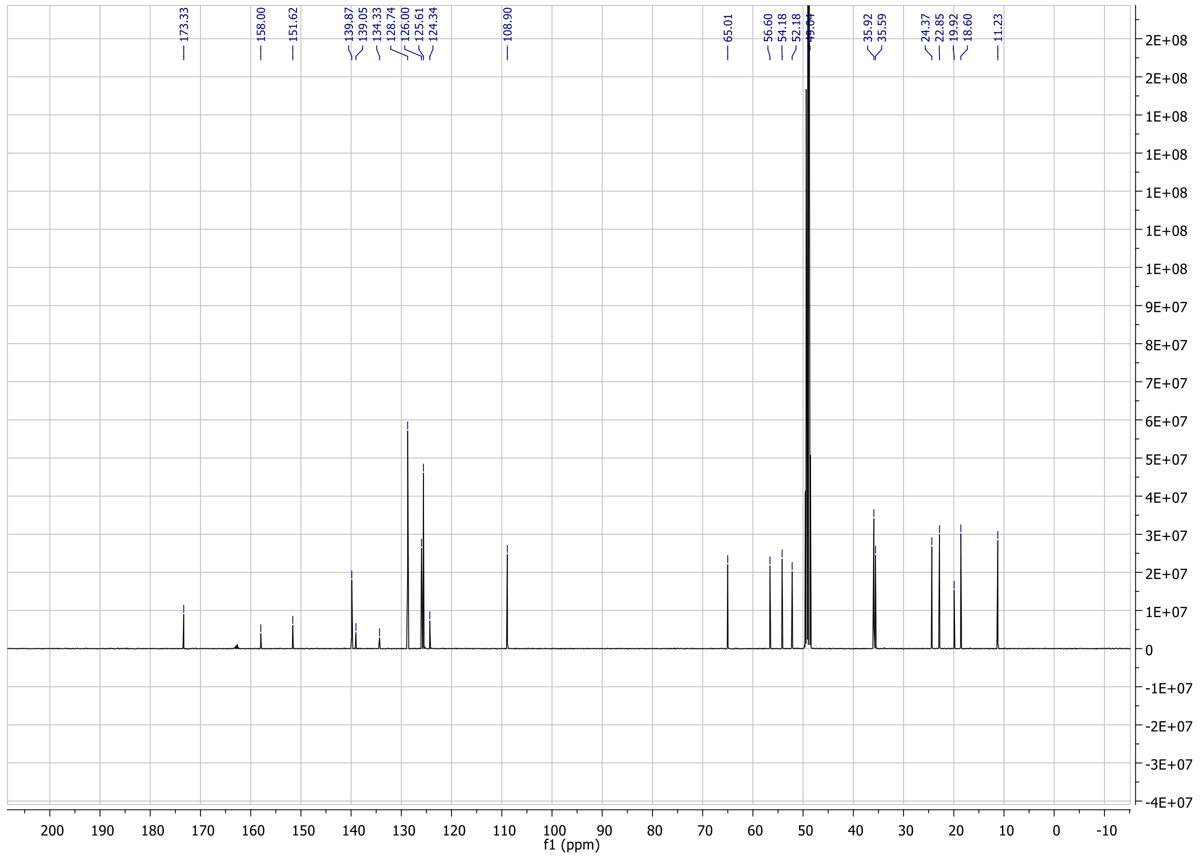

Compound **30**, CD_3OD, 500 MHz

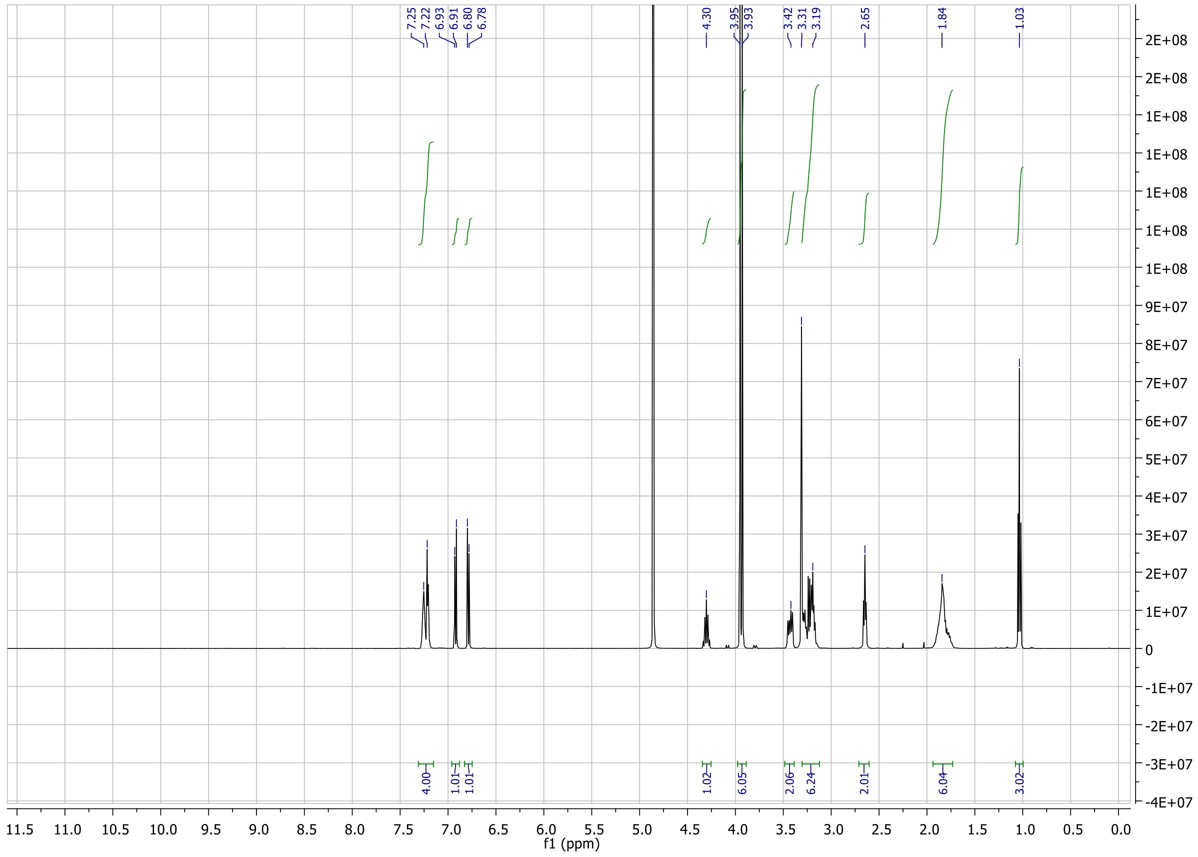

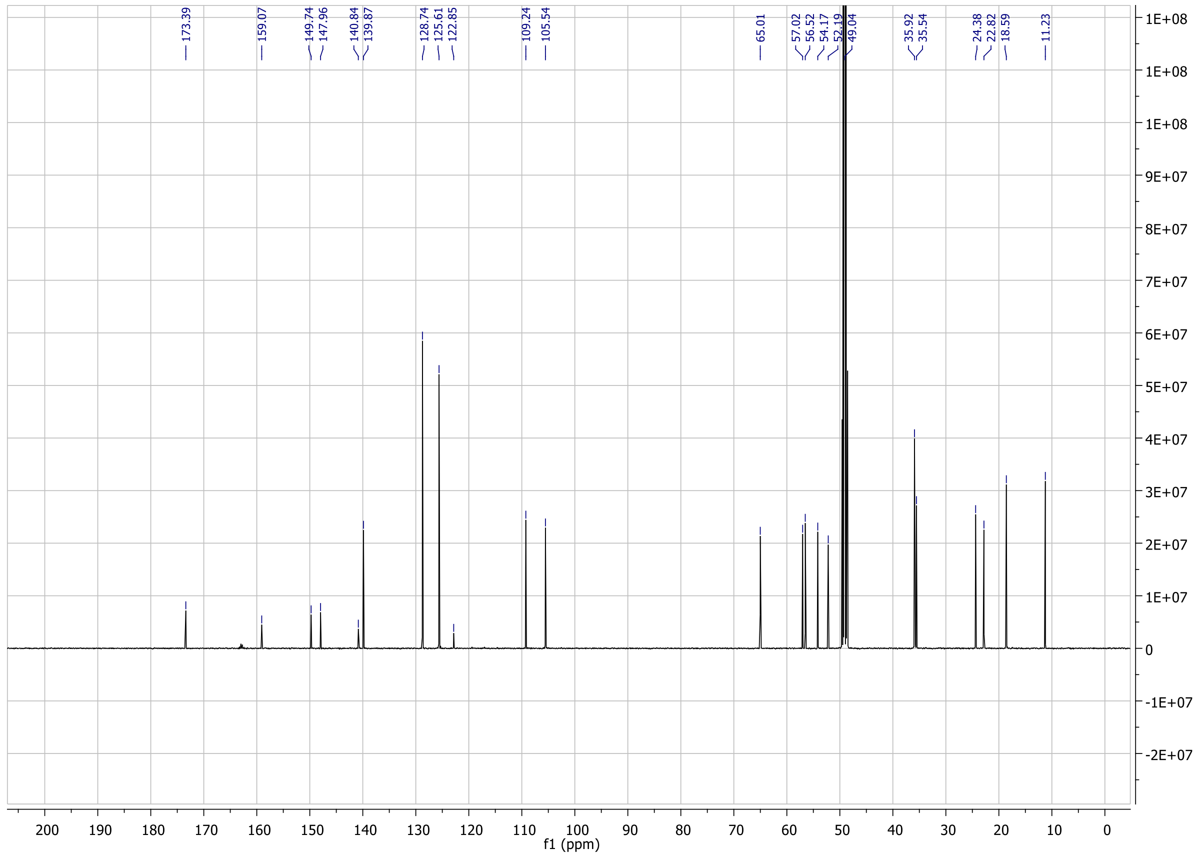

Compound **31**, $CDCl_3$, 400 MHz

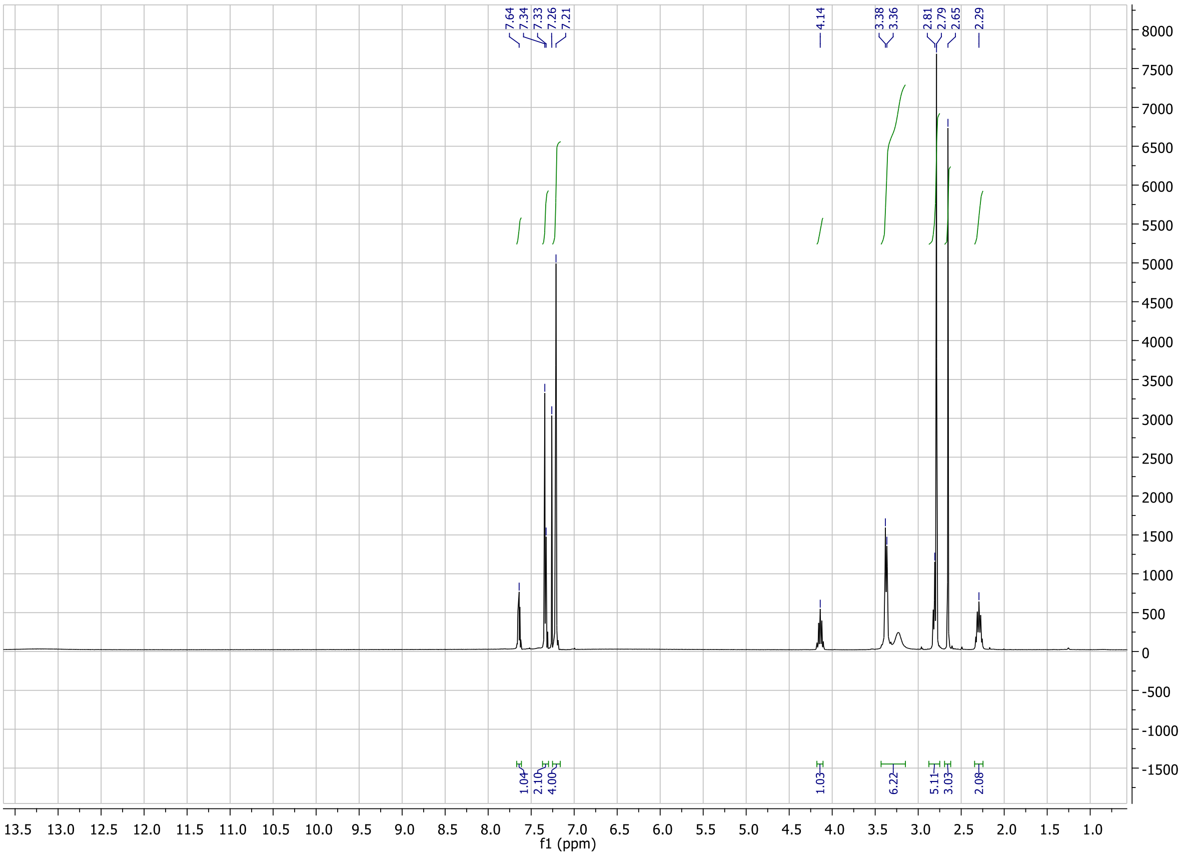

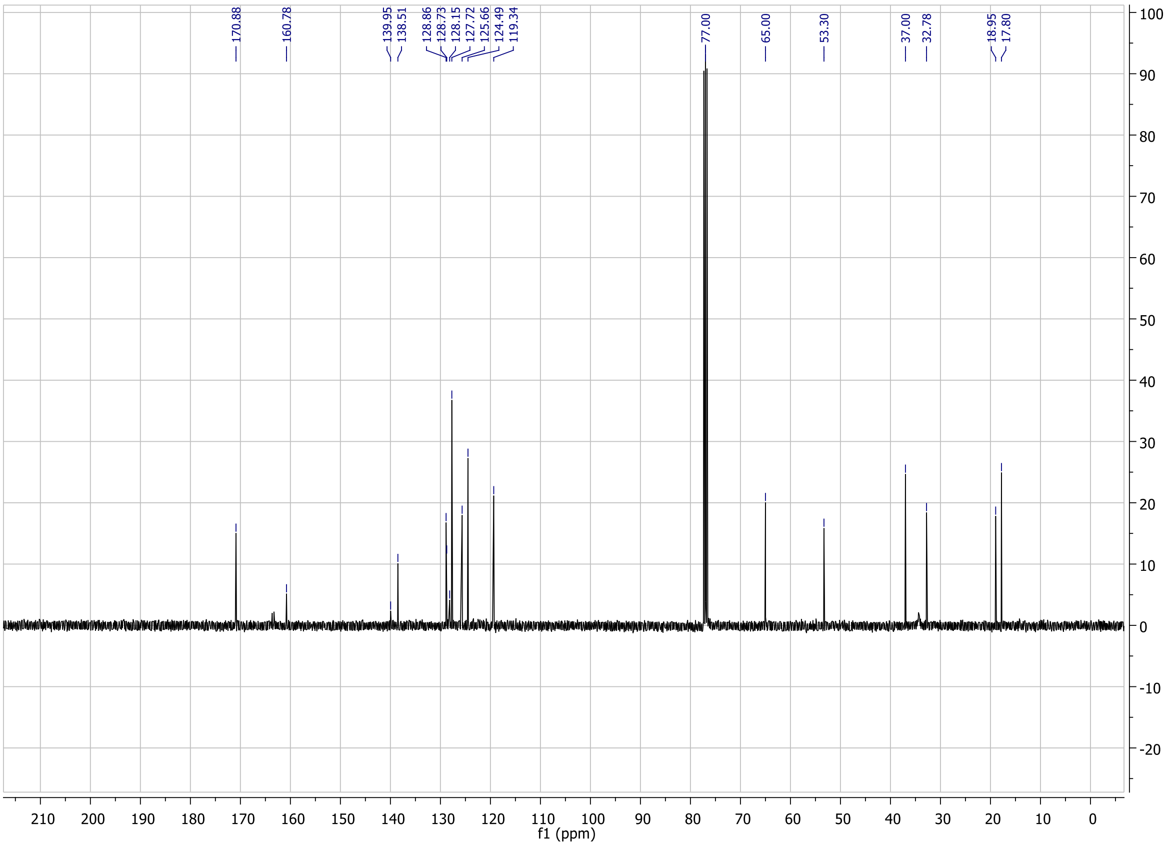

Compound **32**, CD_3OD, 400 MHz

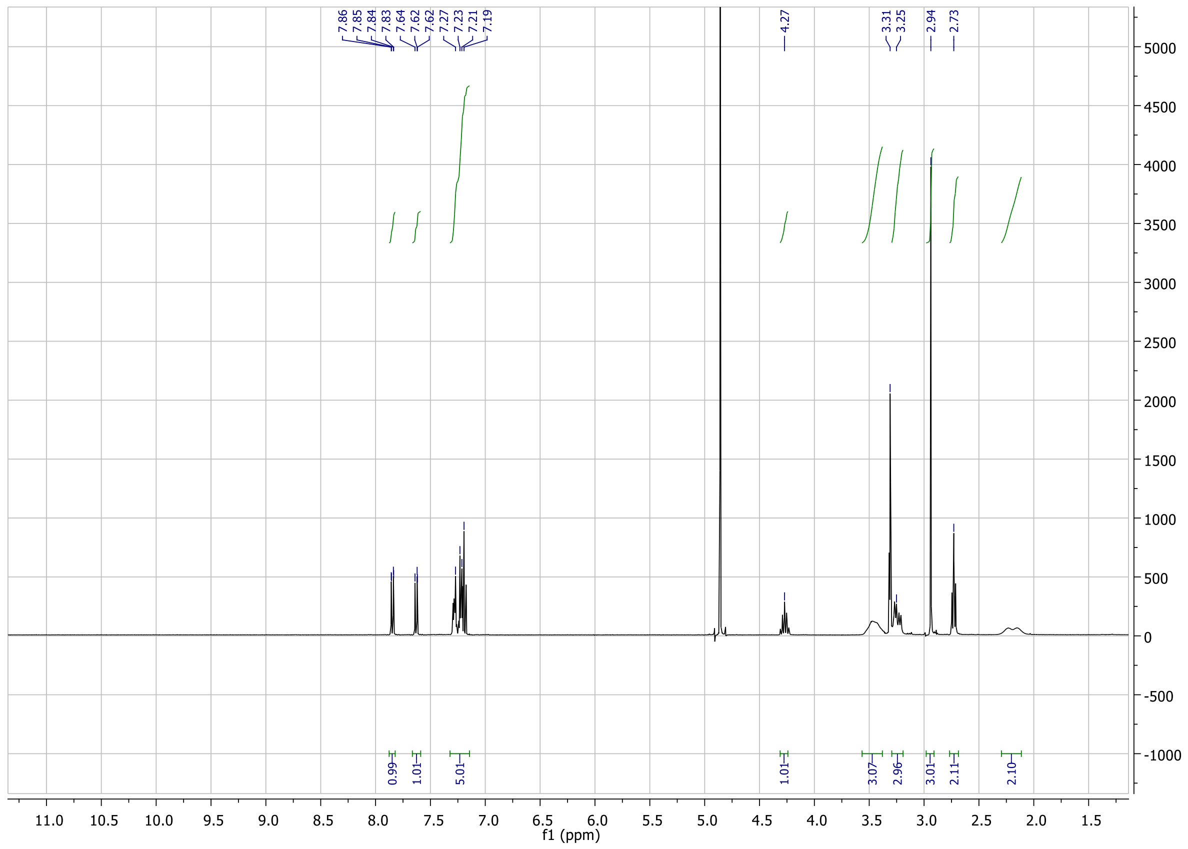

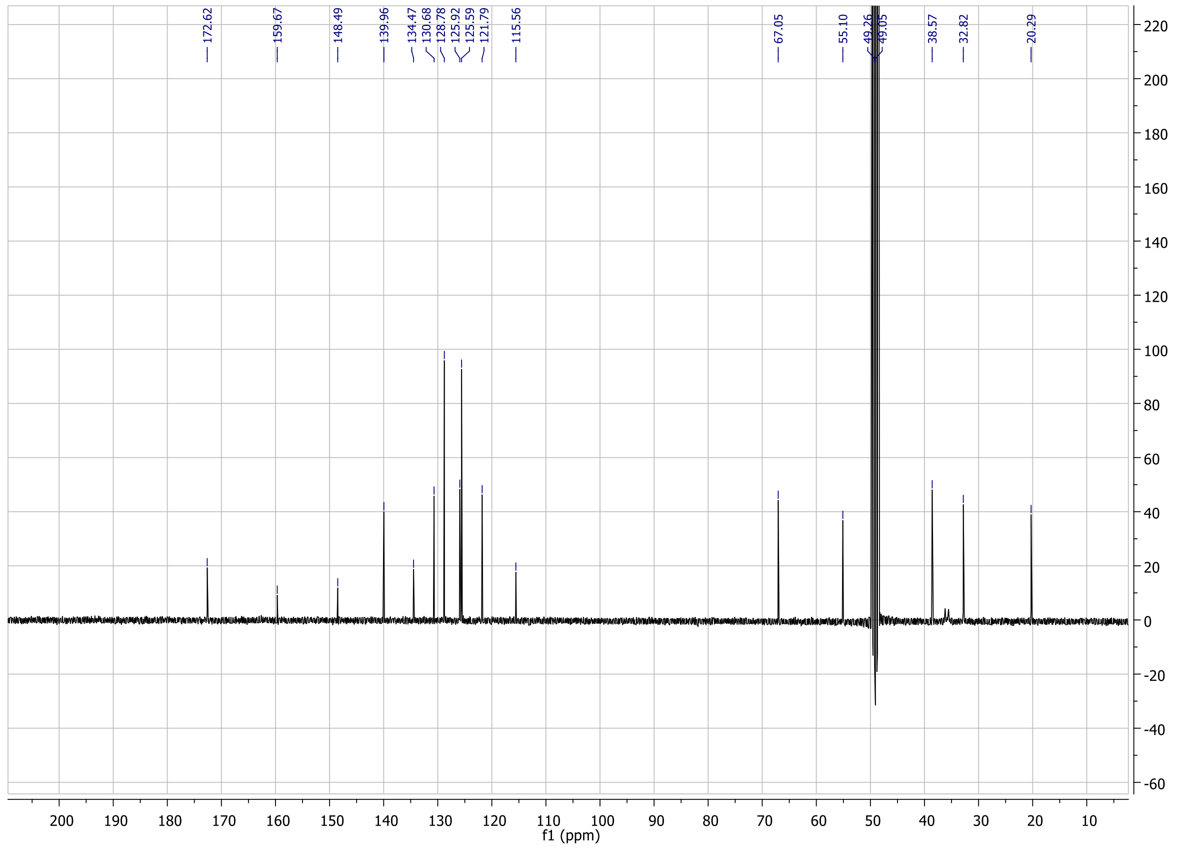

Compound **33**, CD_3OD, 400 MHz

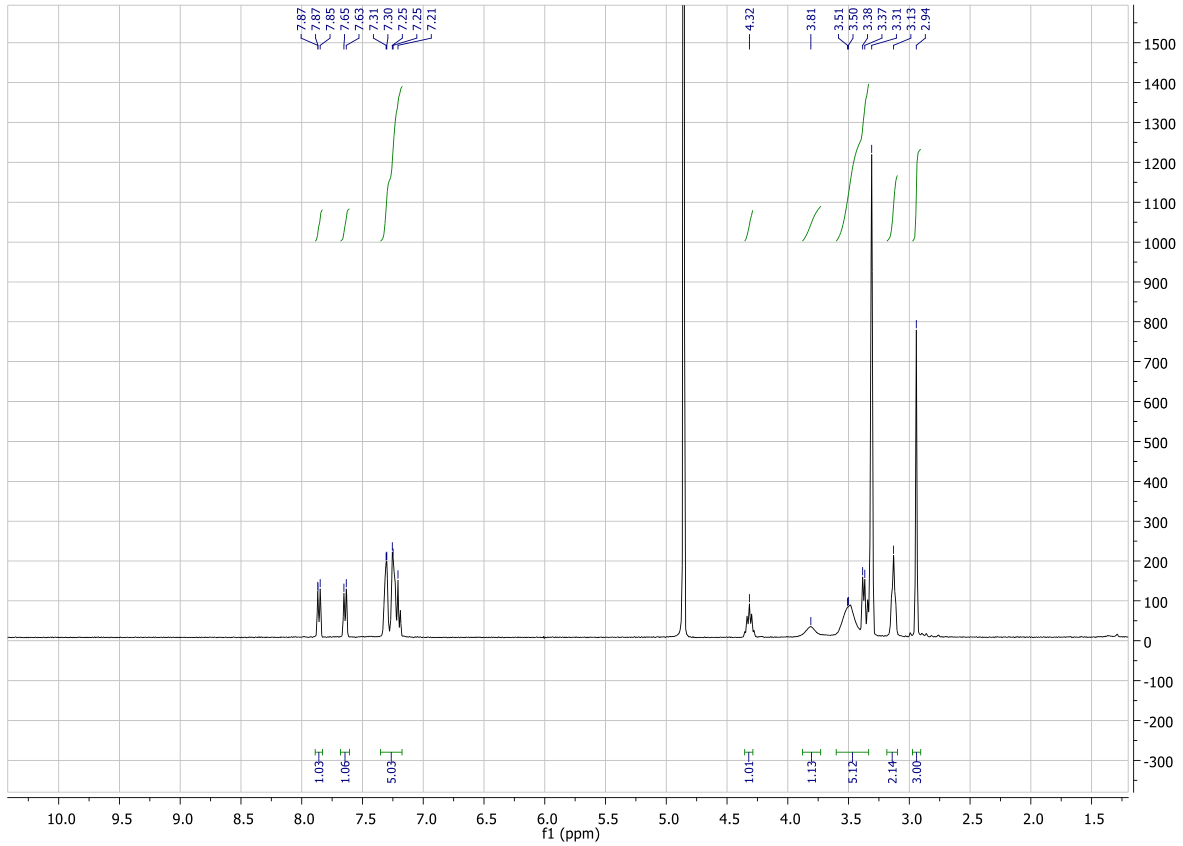

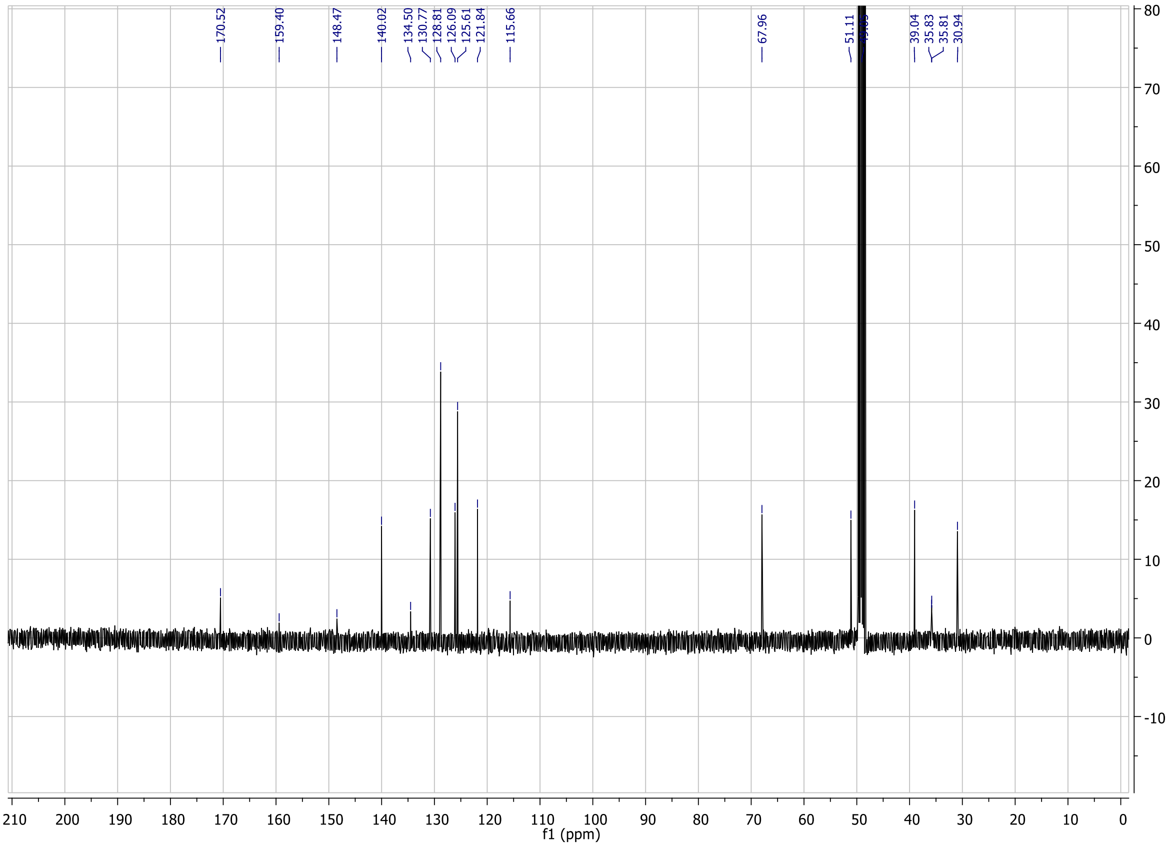

Compound **34**, CD_3OD, 6500 MHz

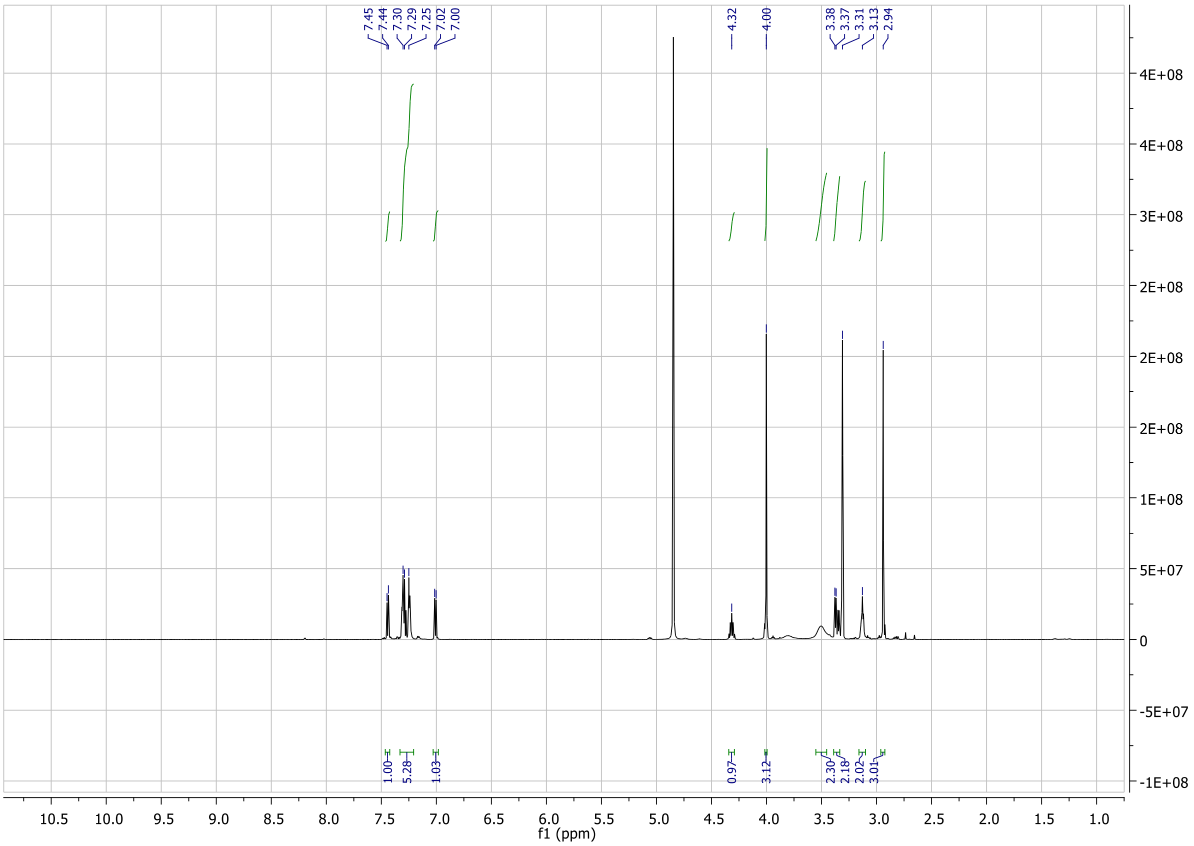

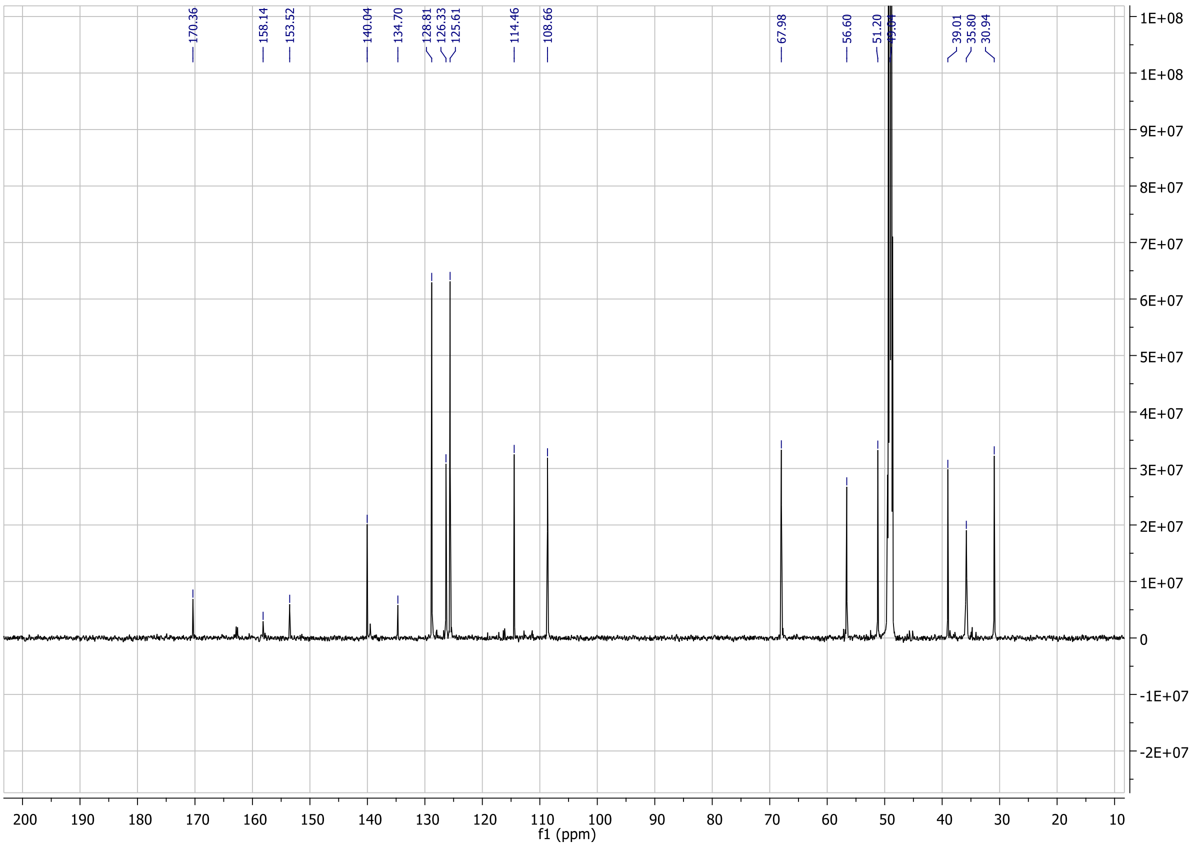

Compound **35**, CD_3OD, 600 MHz

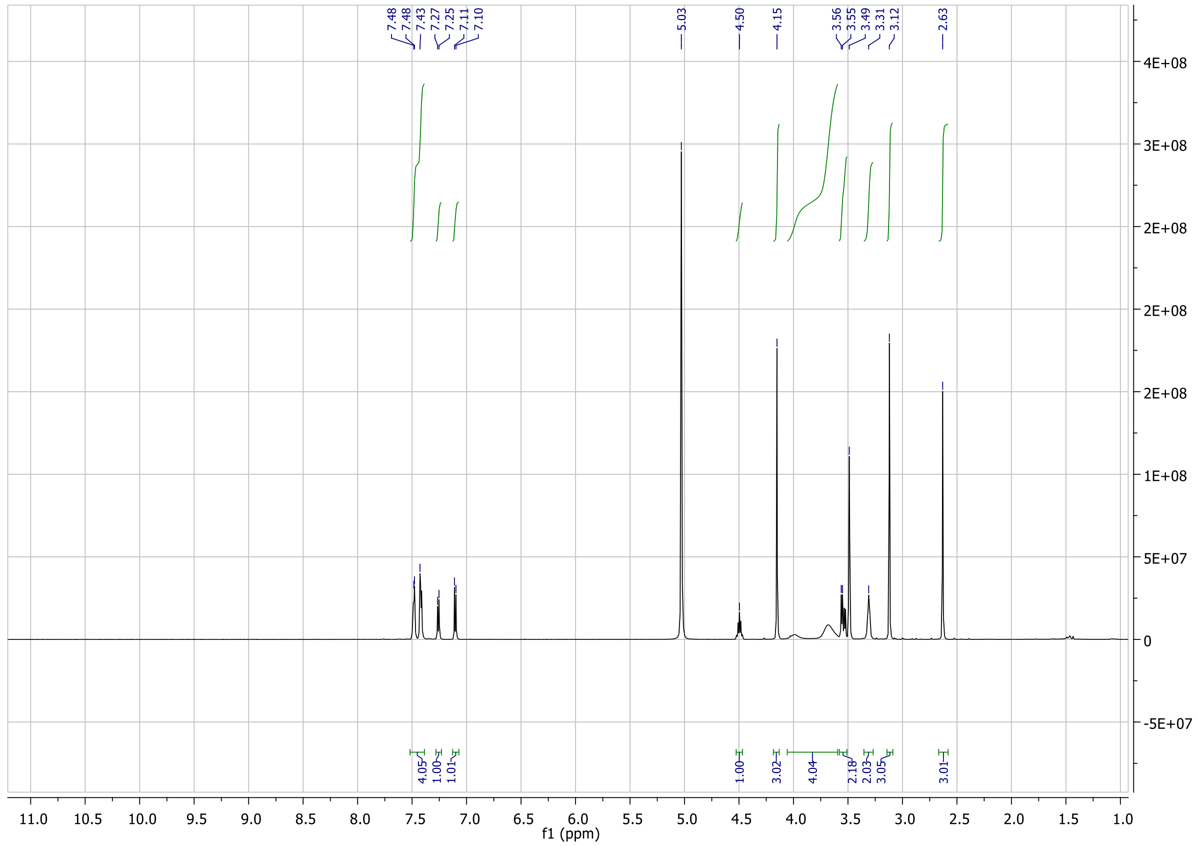

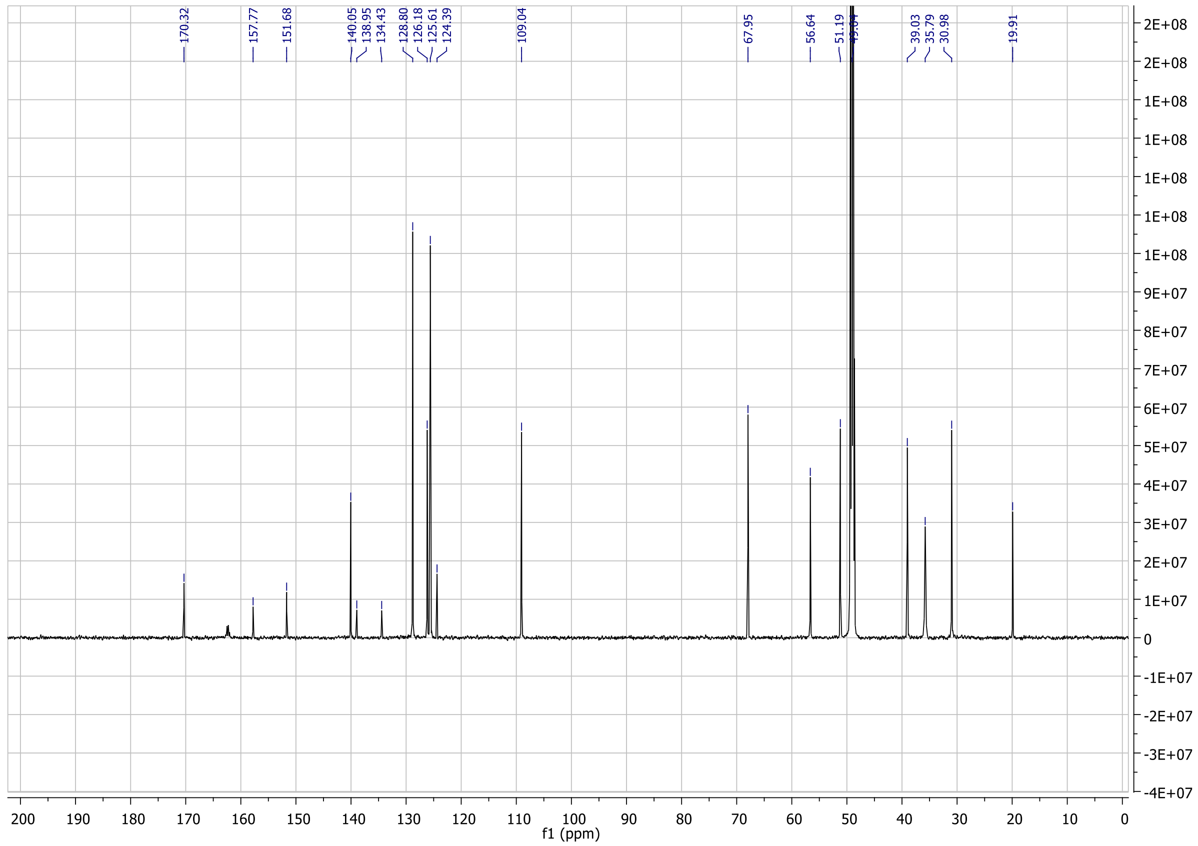

Compound **36**, CD_3OD, 600 MHz

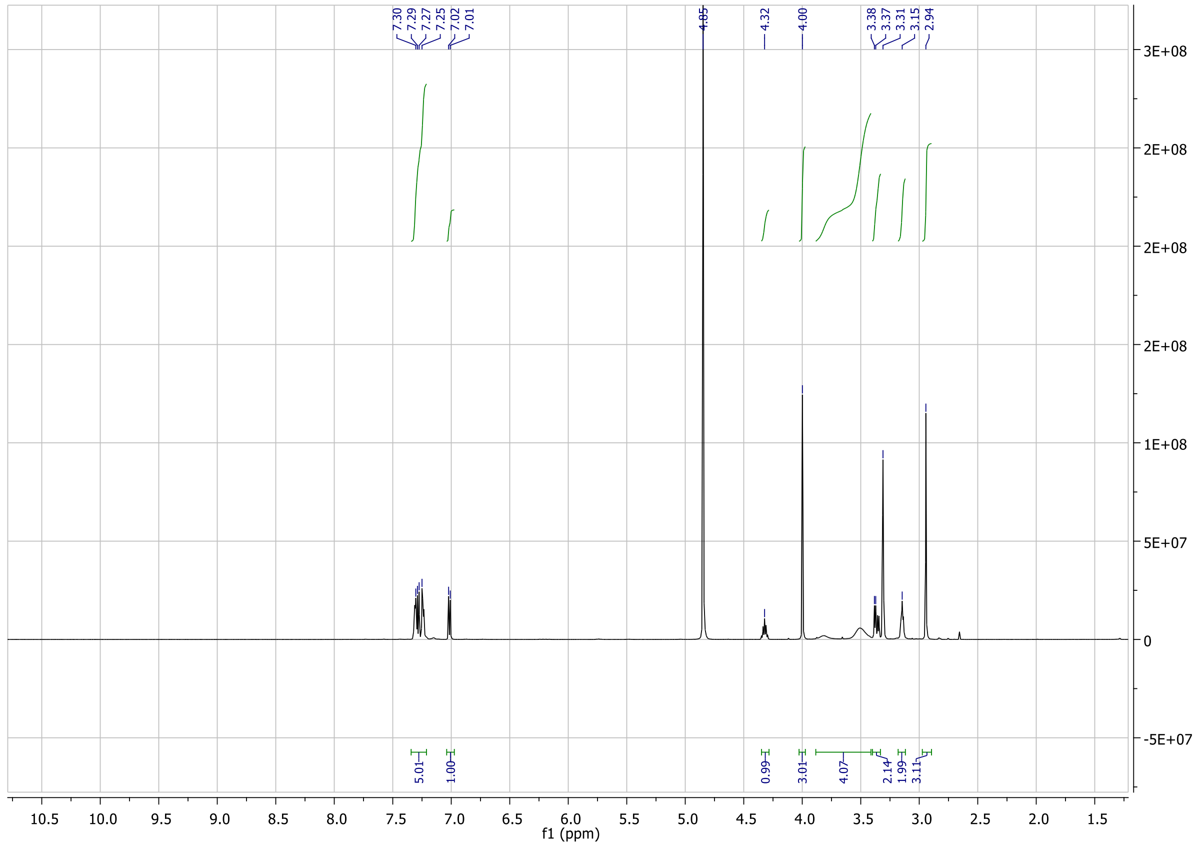

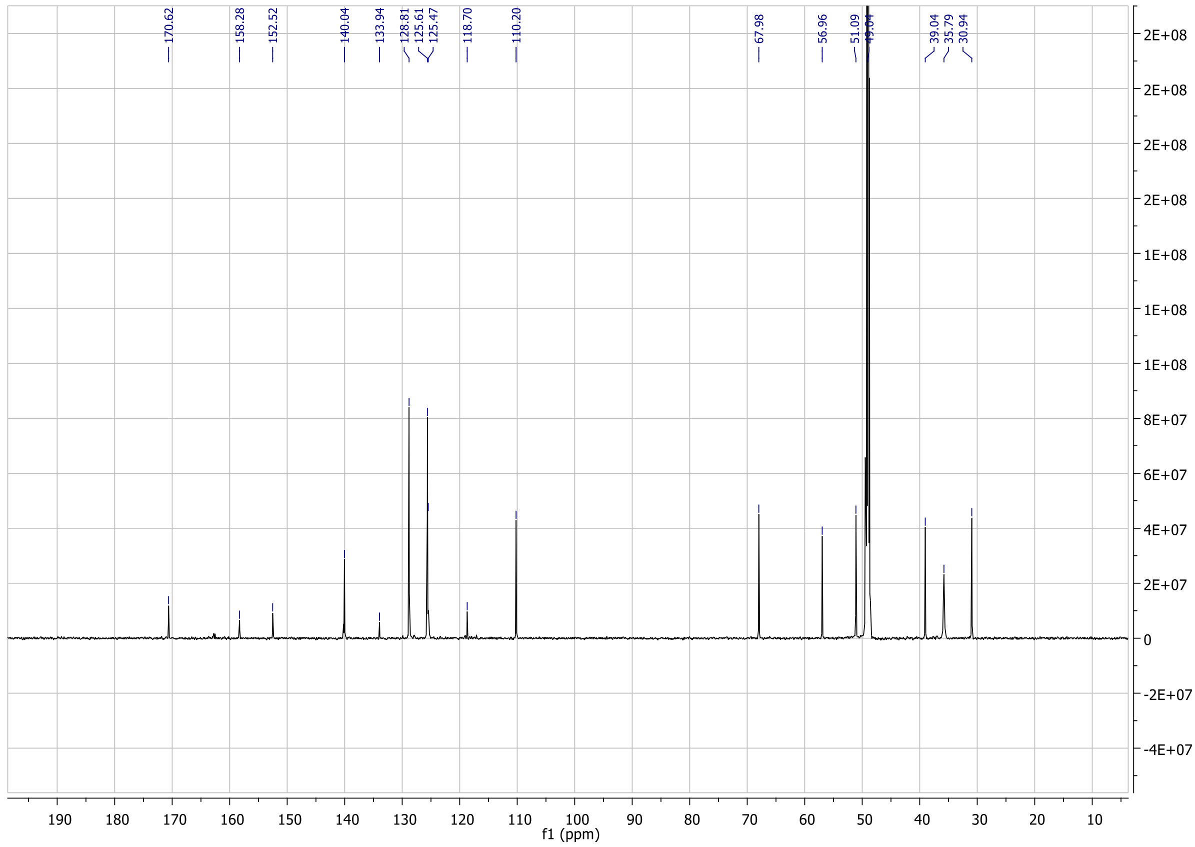

Compound **37**, CD_3OD, 600 MHz

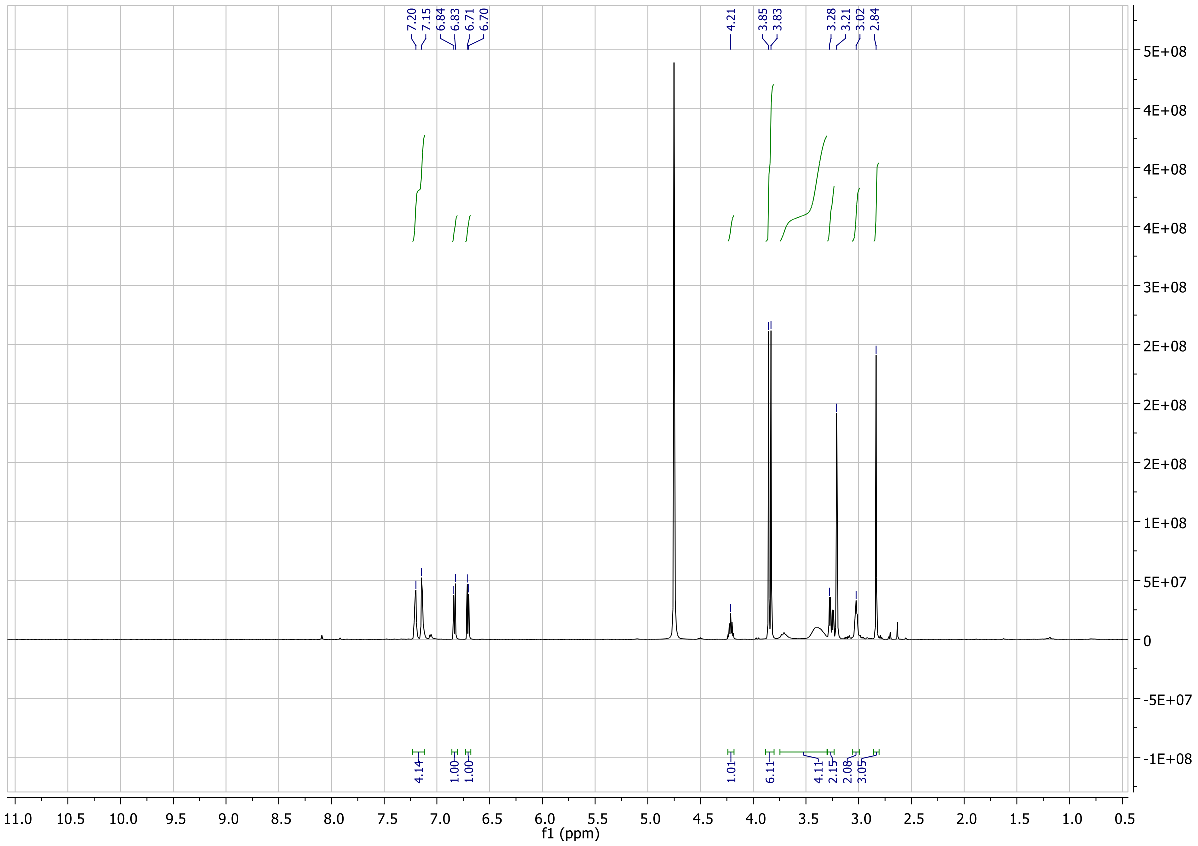

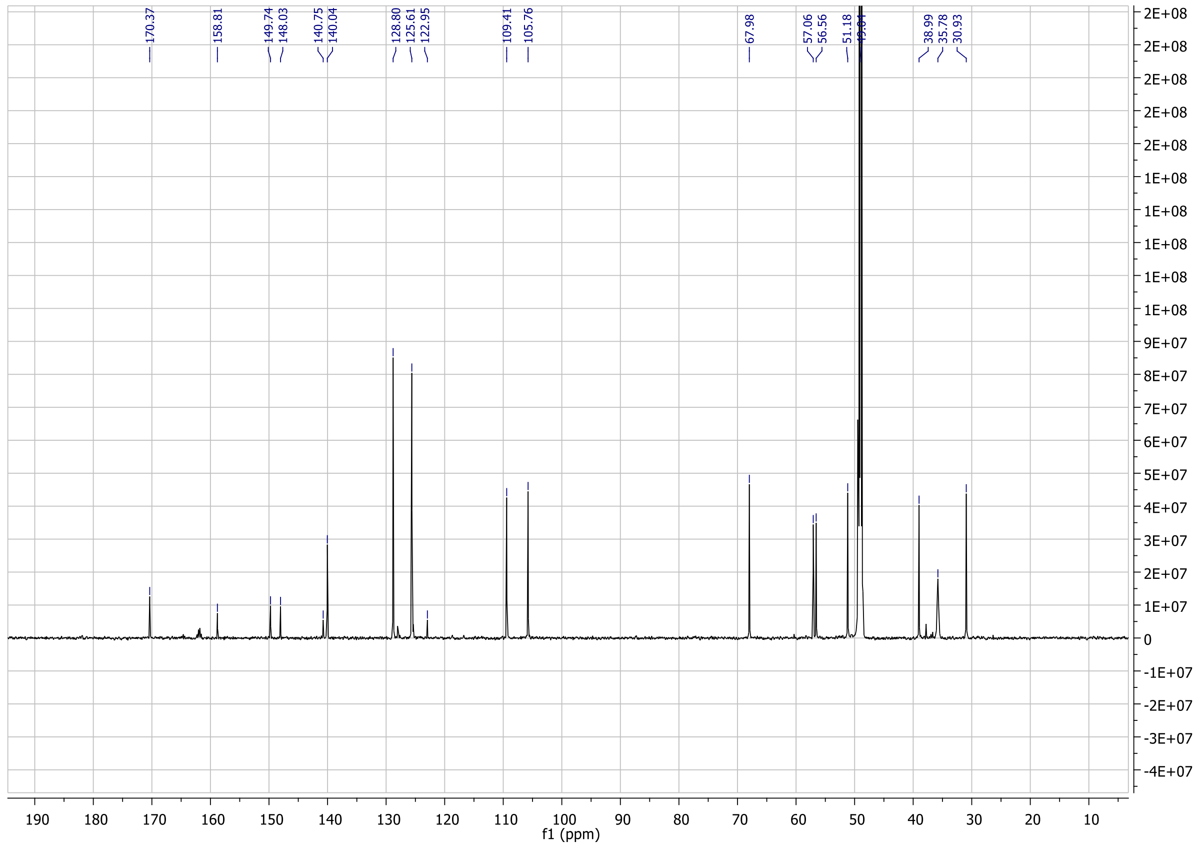

Compound **38**, CD_3OD, 600 MHz

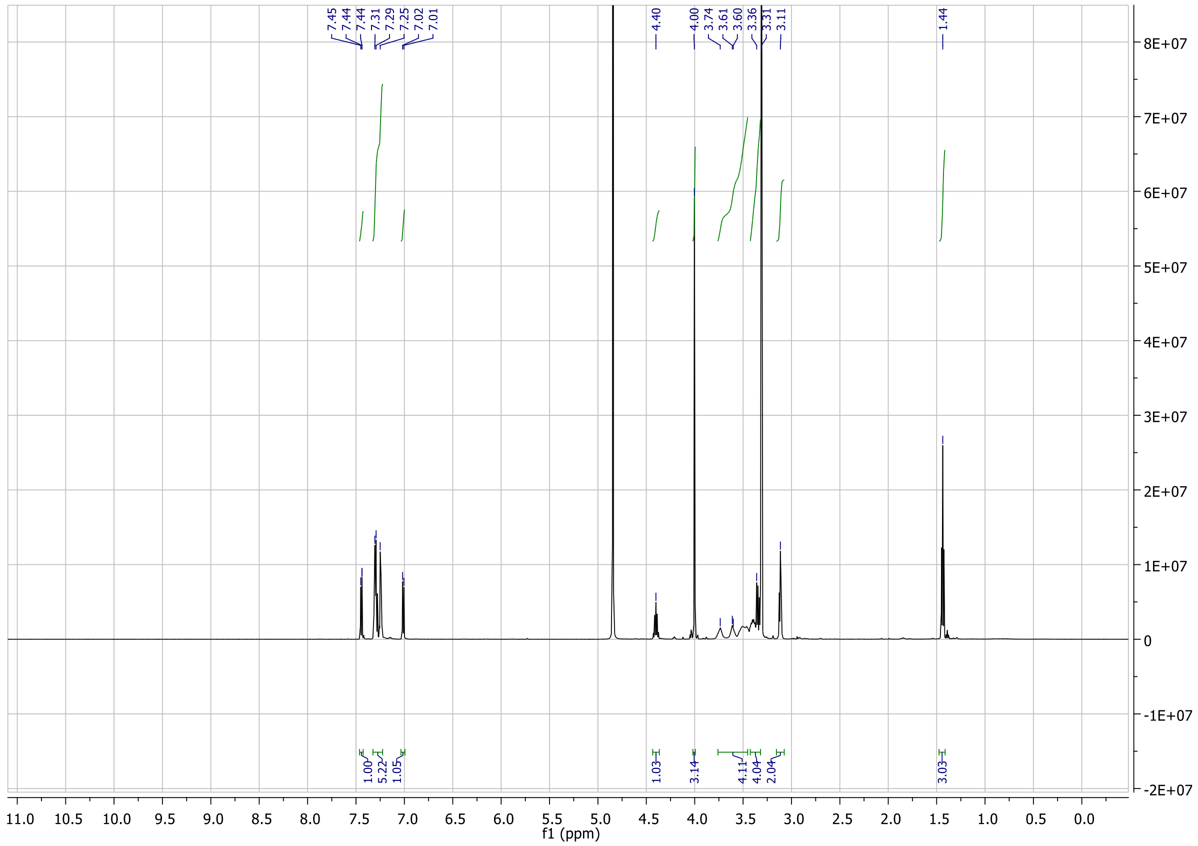

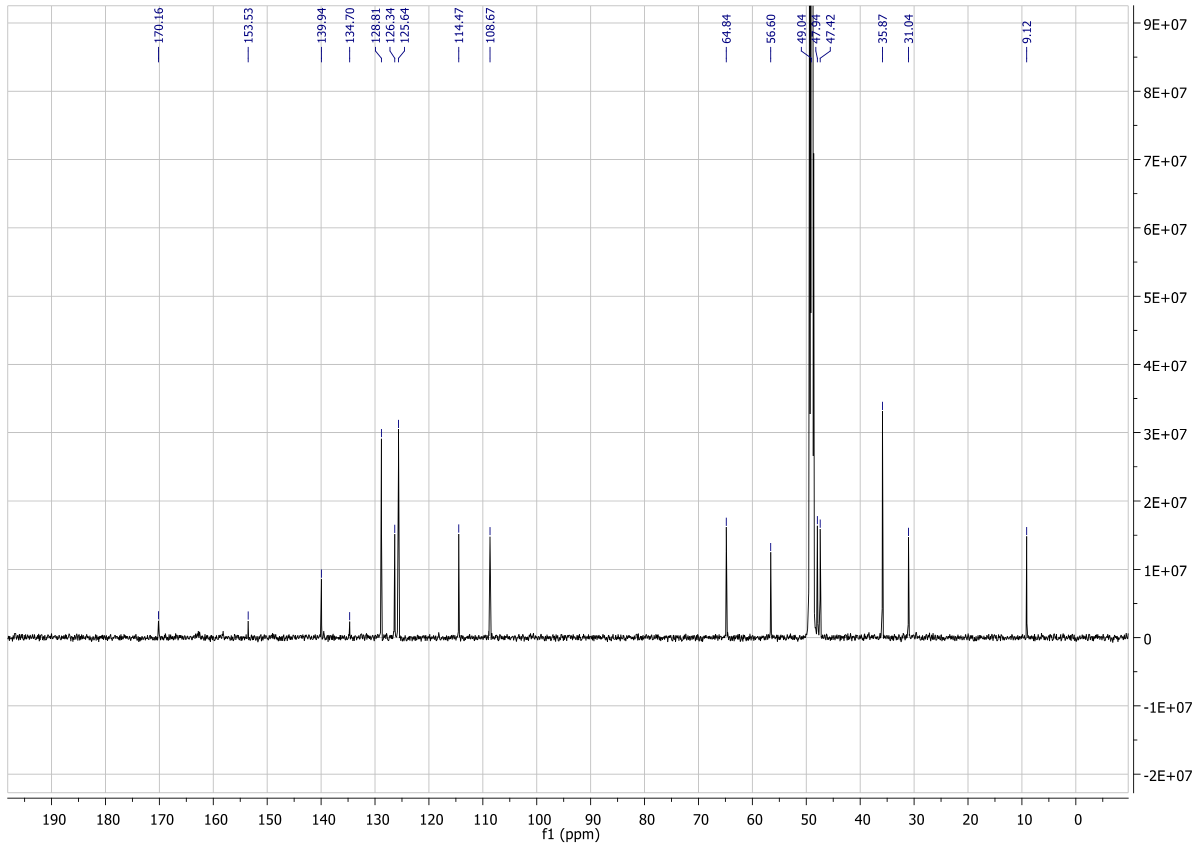

Compound **39**, CD_3OD, 600 MHz

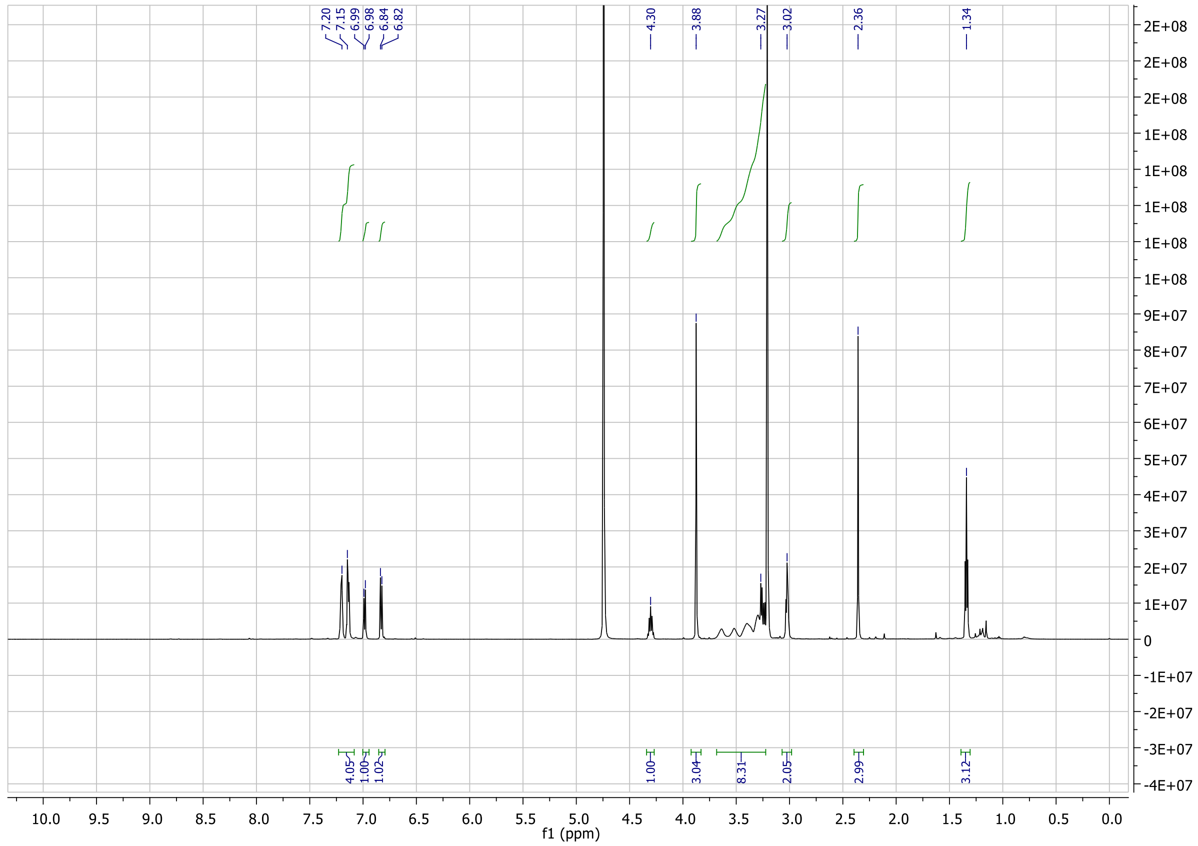

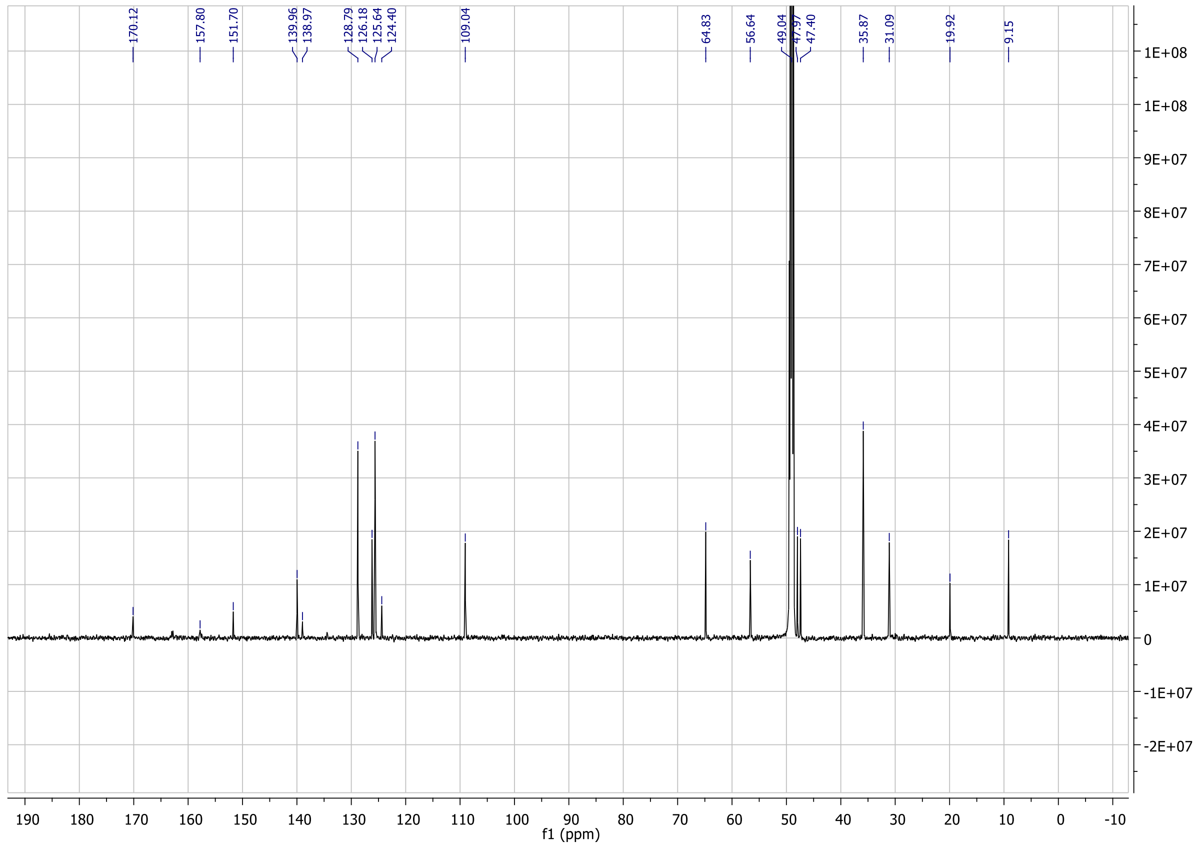

Compound **40**, CD_3OD, 600 MHz

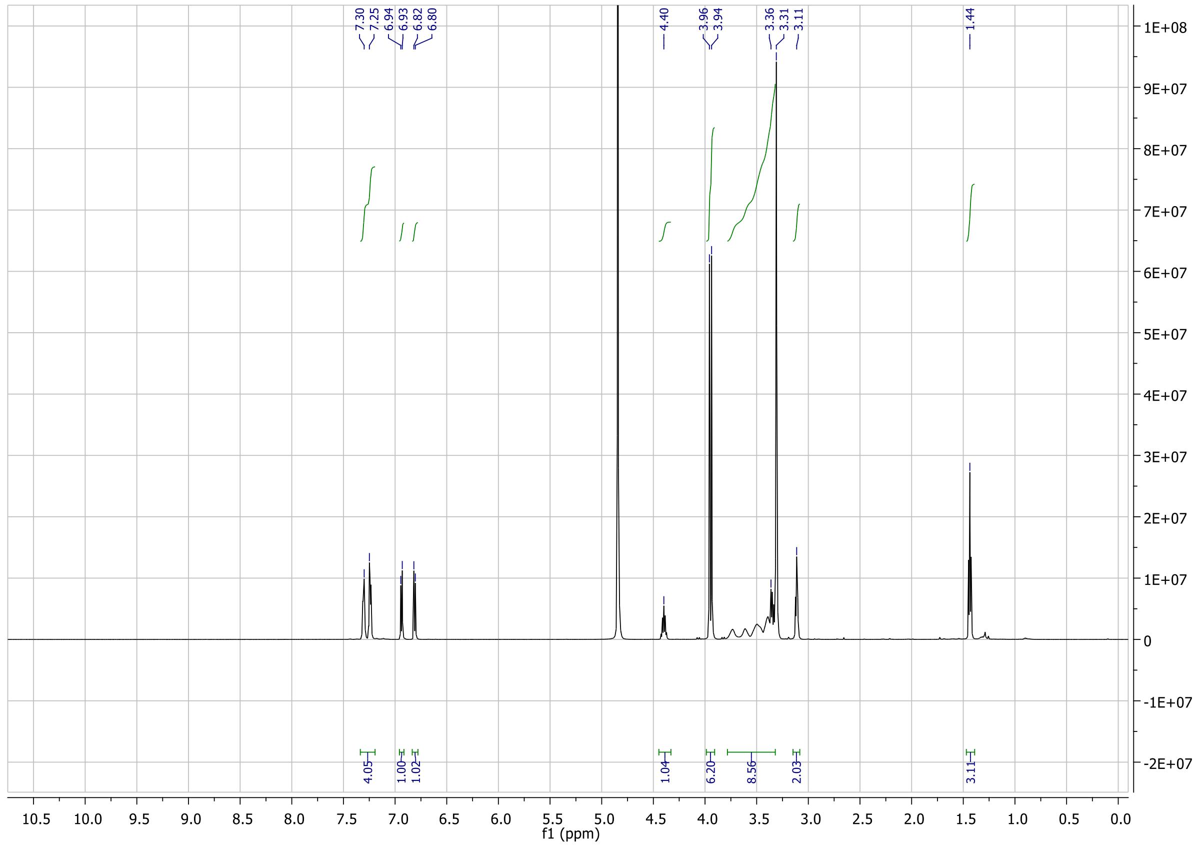

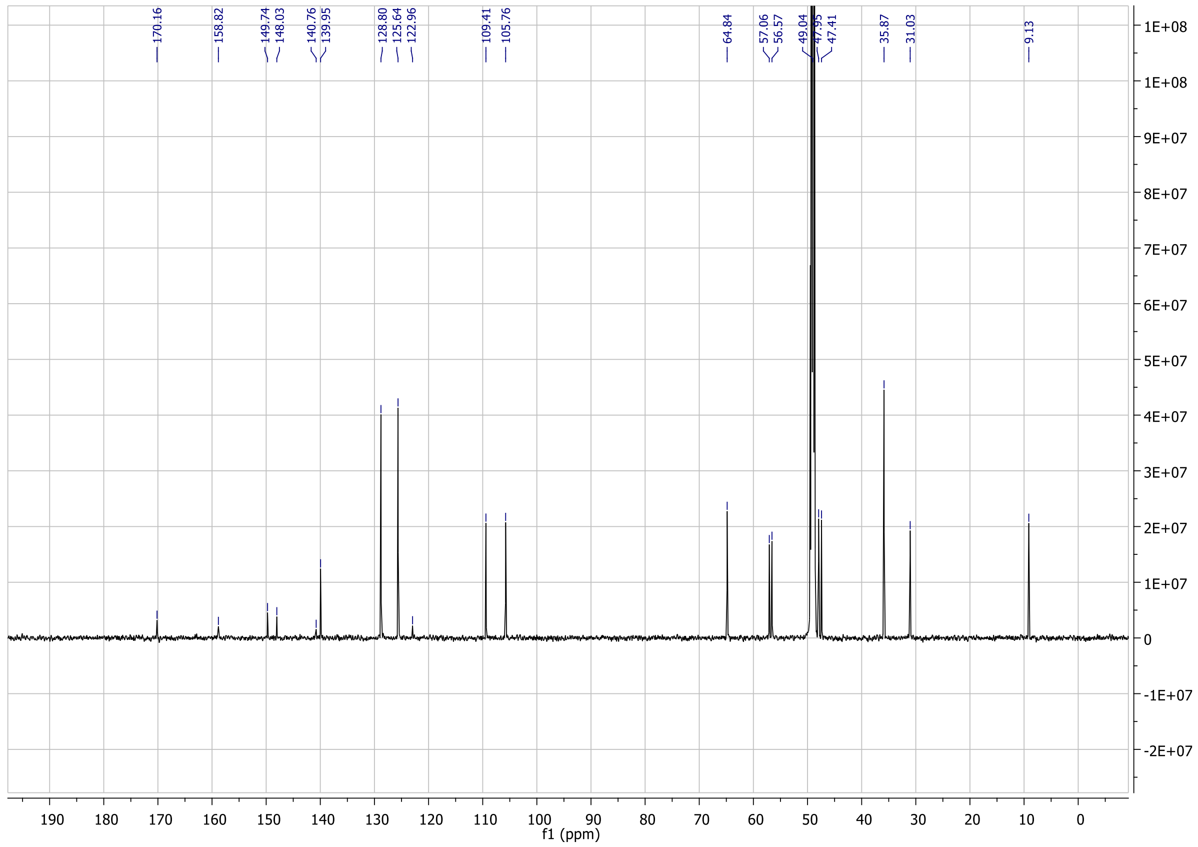

Compound **41**, CD_3OD, 500 MHz

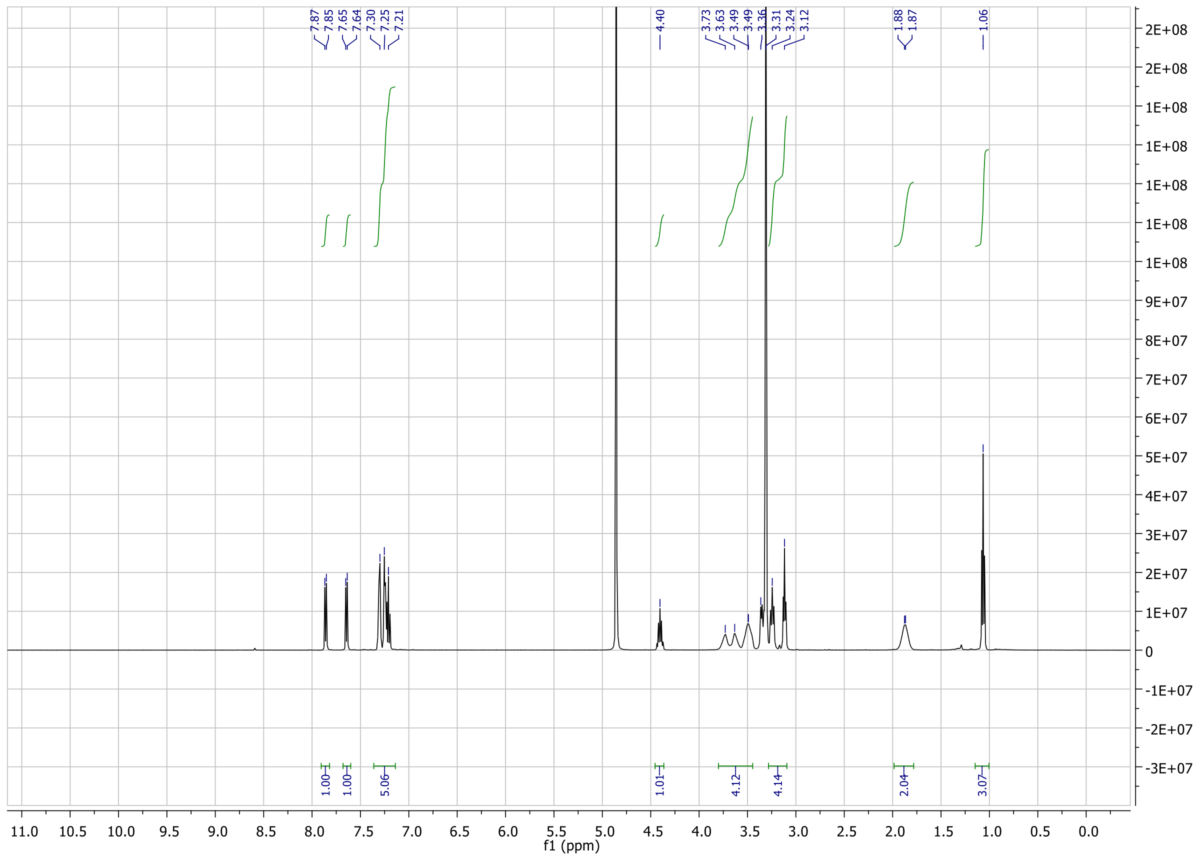

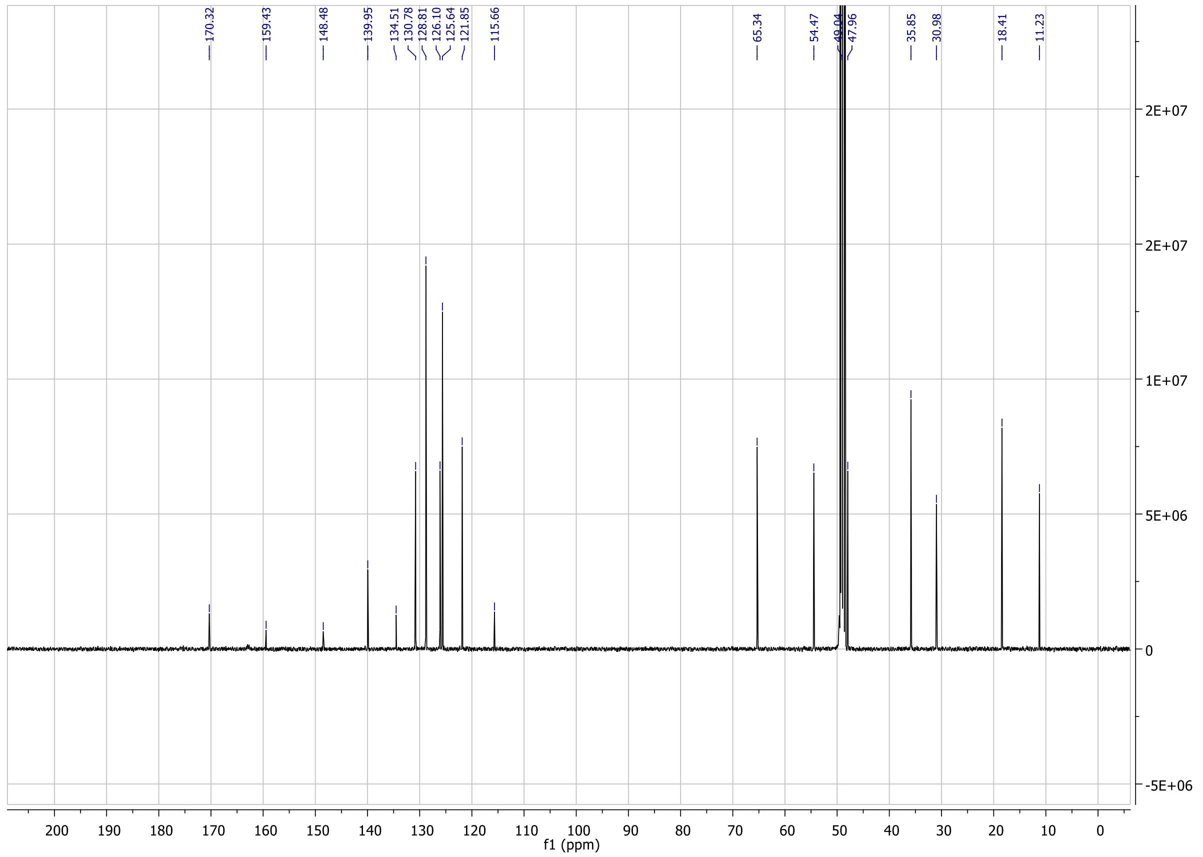

Supplementary References

[1] A. Gaulton, A. Hersey, M. L. Nowotka, A. Patricia Bento, J. Chambers, D. Mendez, P. Mutowo, F. Atkinson, L. J. Bellis, E. Cibrian-Uhalte, M. Davies, N. Dedman, A. Karlsson, M. P. Magarinos, J. P. Overington, G. Papadatos, I. Smit, A. R. Leach, *Nucleic Acids Res.* **2017**, *45*, D945–D954, DOI 10.1093/nar/gkw1074.

[2] T. Sterling, J. J. Irwin, *J. Chem. Inf. Model.* **2015**, *55*, 2324–2337, DOI 10.1021/acs.jcim.5b00559.

[3] J. J. Irwin, T. Sterling, M. M. Mysinger, E. S. Bolstad, R. G. Coleman, *J. Chem. Inf. Model.* **2012**, *52*, 1757–1768, DOI 10.1021/ci3001277.

[4] P. C. D. Hawkins, A. G. Skillman, G. L. Warren, B. A. Ellingson, M. T. Stahl, *J. Chem. Inf. Model.* **2010**, *50*, 572–584, DOI 10.1021/ci100031x.

[5] D. Lagorce, L. Bouslama, J. Becot, M. A. Miteva, B. O. Villoutreix, *Bioinformatics* **2017**, *33*, 3658–3660, DOI 10.1093/bioinformatics/btx491.

[6] A. Šali, T. L. Blundell, *J. Mol. Biol.* **1993**, *234*, 779–815, DOI 10.1006/jmbi.1993.1626.

[7] E. Y. T. Chien, W. Liu, Q. Zhao, V. Katritch, G. W. Han, M. A. Hanson, L. Shi, A. H. Newman, J. A. Javitch, V. Cherezov, R. C. Stevens, *Science (80-.).* **2010**, *330*, 1091–1095, DOI 10.1126/science.1197410.

[8] S. G. F. Rasmussen, B. T. Devree, Y. Zou, A. C. Kruse, K. Y. Chung, T. S. Kobilka, F. S. Thian, P. S. Chae, E. Pardon, D. Calinski, J. M. Mathiesen, S. T. A. Shah, J. A. Lyons, M. Caffrey, S. H. Gellman, J. Steyaert, G. Skiniotis, W. I. Weis, R. K. Sunahara, B. K. Kobilka, *Nature* **2011**, *477*, 549–557, DOI 10.1038/nature10361.

[9] M. M. Mysinger, M. Carchia, J. J. Irwin, B. K. Shoichet, *J. Med. Chem.* **2012**, *55*, 6582–6594, DOI 10.1021/jm300687e.

[10] M. Jaiteh, I. Rodríguez-Espigares, J. Selent, J. Carlsson, *PLOS Comput. Biol.* **2020**, *16*, e1007680, DOI 10.1371/journal.pcbi.1007680.

[11] M. M. Mysinger, B. K. Shoichet, *J. Chem. Inf. Model.* **2010**, *50*, 1561–1573, DOI 10.1021/ci100214a.

[12] A. S. Doré, N. Robertson, J. C. Errey, I. Ng, K. Hollenstein, B. Tehan, E. Hurrell, K. Bennett, M. Congreve, F. Magnani, C. G. Tate, M. Weir, F. H. Marshall, *Structure* **2011**, *19*, 1283–1293, DOI 10.1016/j.str.2011.06.014.

[13] S. J. Weiner, P. A. Kollman, U. C. Singh, D. A. Case, C. Ghio, G. Alagona, S. Profeta, P. Weiner, *J. Am. Chem. Soc.* **1984**, *106*, 765–784, DOI 10.1021/ja00315a051.

[14] D. Rodríguez, Z. G. Gao, S. M. Moss, K. A. Jacobson, J. Carlsson, *J. Chem. Inf. Model.* **2015**, *55*, 550–563, DOI 10.1021/ci500639g.

[15] P. Rucktooa, R. K. Y. Cheng, E. Segala, T. Geng, J. C. Errey, G. A. Brown, R. M. Cooke, F. H. Marshall, A. S. Doré, *Sci. Rep.* **2018**, *8*, DOI 10.1038/s41598-017-18570-w.

[16] H. J. C. Berendsen, D. van der Spoel, R. van Drunen, *Comput. Phys. Commun.* **1995**, *91*, 43–56, DOI 10.1016/0010-4655(95)00042-E.

[17] W. L. Jorgensen, D. S. Maxwell, J. Tirado-Rives, *J. Am. Chem. Soc.* **1996**, *118*, 11225–11236, DOI 10.1021/ja9621760.

[18] O. Berger, O. Edholm, F. Jähnig, *Biophys. J.* **1997**, *72*, 2002–2013, DOI 10.1016/S0006-3495(97)78845-3.

[19] W. L. Jorgensen, J. Chandrasekhar, J. D. Madura, R. W. Impey, M. L. Klein, *J. Chem. Phys.* **1983**, *79*, 926–935, DOI 10.1063/1.445869.

[20] J. Marelius, K. Kolmodin, I. Feierberg, J. Åqvist, *J. Mol. Graph. Model.* **1998**, *16*, 213–225, DOI 10.1016/S1093-3263(99)00012-1.

[21] M. J. Robertson, J. Tirado-Rives, W. L. Jorgensen, *J. Chem. Theory Comput.* **2015**, *11*, 3499–3509, DOI 10.1021/acs.jctc.5b00356.
[22] G. King, A. Warshel, *J. Chem. Phys.* **1989**, *91*, 3647–3661, DOI 10.1063/1.456845.
[23] J. P. Ryckaert, G. Ciccotti, H. J. C. Berendsen, *J. Comput. Phys.* **1977**, *23*, 327–341, DOI 10.1016/0021-9991(77)90098-5.
[24] F. S. Lee, A. Warshel, *J. Chem. Phys.* **1992**, *97*, 3100–3107, DOI 10.1063/1.462997.
[25] C. Yung-Chi, W. H. Prusoff, *Biochem. Pharmacol.* **1973**, *22*, 3099–3108, DOI 10.1016/0006-2952(73)90196-2.
[26] I. Hubatsch, E. G. E. Ragnarsson, P. Artursson, *Nat. Protoc.* **2007**, *2*, 2111–2119, DOI 10.1038/nprot.2007.303.
[27] M. P. A. Sanders, L. Roumen, E. Van Der Horst, J. R. Lane, H. F. Vischer, J. Van Offenbeek, H. De Vries, S. Verhoeven, K. Y. Chow, F. Verkaar, M. W. Beukers, R. McGuire, R. Leurs, A. P. Ijzerman, J. De Vlieg, I. J. P. De Esch, G. J. R. Zaman, J. P. G. Klomp, A. Bender, C. De Graaf, *J. Med. Chem.* **2012**, *55*, 5311–5325, DOI 10.1021/jm300280e.
[28] S. A. Hitchcock, L. D. Pennington, *J. Med. Chem.* **2006**, *49*, 7559–7583, DOI 10.1021/jm060642i.
[29] K. A. Jacobson, Z. Gao, P. Matricon, M. T. Eddy, J. Carlsson, *Br. J. Pharmacol.* **2020**, *n/a*, DOI 10.1111/bph.15103.
[30] P. Davies, T. G. Hales, A. A. Jensen, J. A. Peters, *IUPHAR/BPS Guid. to Pharmacol. CITE* **2019**, *2019*, DOI 10.2218/gtopdb/f83/2019.4.
